# Copper-catalysed site-selective arylation of pyrazoles

**DOI:** 10.1038/s41557-026-02148-z

**Published:** 2026-06-12

**Authors:** Minghao Wang, Xintong Hou, Stephanie A. Corio, Lori Digal, Jennifer S. Hirschi, Vy M. Dong

**Affiliations:** 1 Department of Chemistry, University of California, Irvine, Irvine, CA USA; 2 Department of Chemistry, Binghamton University, Binghamton, NY USA

**Keywords:** Synthetic chemistry methodology, Catalysis

## Abstract

A major challenge in late-stage functionalization is the selective *N*-arylation of unsymmetric pyrazoles, five-membered heterocycles with two adjacent nitrogen atoms, which are prevalent in blockbuster medicines and bioactive molecules. Traditional cross-coupling methods usually favour one type of regioisomer, limiting access to complementary structures that could accelerate drug discovery and streamline structure–activity relationship studies. Here we show that copper catalysis harnesses arynes to achieve switchable arylation of pyrazoles under mild conditions. By tuning Cu–pyrazole coordination, we direct arylation to either nitrogen atom, unlocking selectivity between two similar sites. Mechanistic studies reveal how steric and electronic effects govern regioselectivity, transforming an inherently unpredictable process into a blueprint for selective recognition of N–N linkages. This approach establishes a general strategy for controlling site selectivity in nitrogen-rich heterocycles and highlights the use of strained intermediates in late-stage diversification.

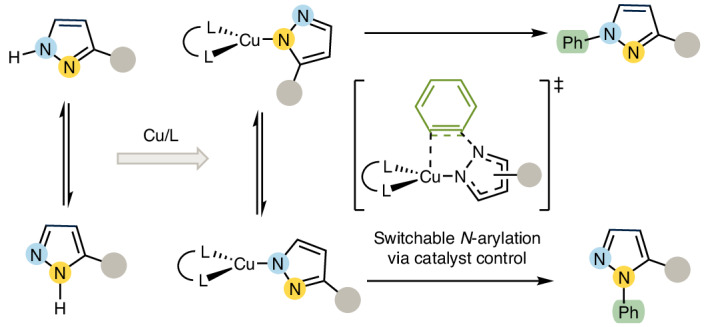

## Abstract (web summary)

*N*-aryl pyrazole is a prevalent motif among pharmaceuticals across various therapeutic areas but can be challenging to synthesize due to the high similarity of the two nitrogen atoms. Now it has been shown that the use of copper catalysis and arynes provides a switchable protocol to access both regioisomers.

## Main

**Fig. 1:**
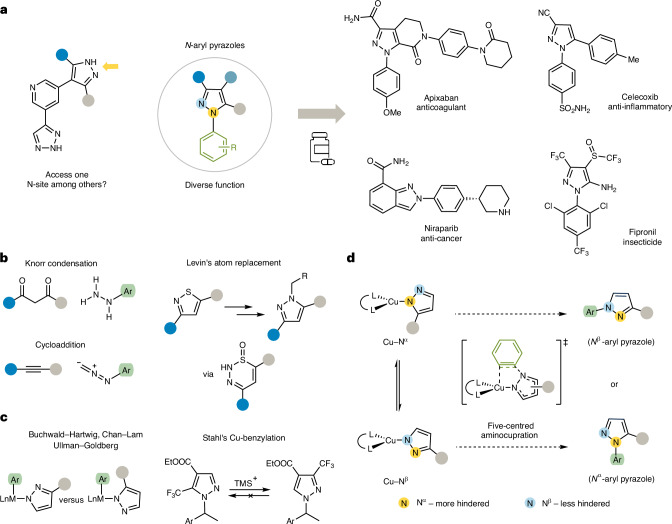
Regioselective synthesis of *N*-aryl pyrazoles. **a**, Left: Blakemore et al. pose a hypothetical molecule and the challenge of achieving regioselective nitrogen functionalization to facilitate drug discovery^3^. Right: *N*-aryl pyrazoles are key pharmacophores with two distinct nitrogen sites and are found in blockbuster drugs worth over US$19 billion (ref. ^6^). **b**, Select de novo strategies include Knorr condensation, cycloaddition and Levin’s atom replacement^8,9^. **c**, Select state-of-the-art catalytic strategies include Buchwald–Hartwig, Chan–Lam, Ullman–Goldberg and Stahl’s benzylation^13,24–27^. **d**, The design of a Cu-catalysed arylation via a five-centred aminocupration pathway to enable a switchable and chemoselective arylation of pyrazoles using arynes. Leveraging the N–N motif enables chemoselective functionalization in the presence of other amine functional groups. TMS, trimethylsilyl; Ln, ligand.

Catalysis has revolutionized the discovery of medicines by enabling the tuning of scaffolds at a late stage in a synthetic route^1,2^, yet organic synthesis remains a bottleneck in drug discovery^3^. Among the various challenges that exist, medicinal chemists seek novel ways to target specific nitrogen atoms without the need for protecting groups^4,5^ (Fig. 1a). Bearing two linked nitrogen atoms, the *N*-aryl pyrazole has emerged as a pivotal pharmacore across blockbuster drugs for cancer, cardiovascular and anti-inflammatory diseases^6,7^. Classic preparations, such as the Knorr condensation with arylhydrazines and 1,3-dipolar cycloadditions with diazoalkanes, suffer from a lack of regioselective control (Fig. 1b); a mixture of isomers is often obtained or access to only one isomer is possible depending on the substrate structure^8^. As an alternative to this textbook approach, Levin reported the regioselective synthesis of *N*-alkyl pyrazoles from isothiazoles using an atom-replacement strategy^9^. In contrast to these de novo ring constructions, there has been progress in the alkylation of pyrazoles^10–15^, including Stahl’s Cu-catalysed benzylation, where the regioselectivity is controlled by the thermodynamic difference between the products^13^. The preparation of parent N–H pyrazoles followed by catalytic, chemoselective and switchable arylation would provide a complementary yet powerful way to modify pyrazoles^16^, allowing access to regioisomeric analogues with diverse pharmaceutical properties^17^. While the Buchwald–Hartwig amination has become a cornerstone for C–N bond construction, there remains a need to replace Pd catalysts with less toxic and more sustainable Cu catalysts. Oxidative addition is challenging for Cu(I), but the invention of novel ligands^18^ and mechanistic pathways^19^, most notably single-electron paths, represent emerging solutions^20–22^. In principle, this switchable arylation could be realized via the Buchwald–Hartwig, Ullman–Goldberg and Chan–Lam paradigm, but these transformations typically favour arylation of the less-hindered nitrogen (N^β^) of the pyrazole, with isolated examples dictated by substrates^23^. This general preference for the less crowded *N*-site mirrors the innate selectivity of nucleophilic aromatic substitution (S_N_Ar), leaving the more crowded site (N^*α*^) elusive for late-stage arylation^24–27^. Inspired by this challenge, we aimed to design a catalytic arylation of heterocycles with these criteria in mind: (1) enable the selective arylation of N–N motifs in the presence of other N sites, (2) allow for the switchable arylation of the two pyrazole N sites on the basis of catalyst control rather than substrate control and (3) overcome the challenge of oxidative addition by harnessing a unique electrophile for room temperature C–N formation. To address this challenge, we turned to Cu–aryne catalysis.

While never isolated, Cu–aryne complexes have been detected in the gas phase^28^ and implicated in divergent transformations, thus suggesting their viability as catalytic intermediates^29–32^. With unsymmetric pyrazole and a Cu catalyst, we proposed an equilibrium between tautomeric Cu complexes Cu–N^β^ and Cu–N^α^, where either N^β^ or N^α^ from the pyrazole coordinates to Cu, respectively^24^ (Fig. 1c). These metallotautomers intercept a transient aryne formed in situ to channel *N*-arylation through a five-centred transition state. The design of this unique aminocupration draws insights from our laboratory’s own studies on the Cu-catalysed enantioselective addition of pyrazoles to cyclopropenes^10^. The similarity in strain energy between arynes and cyclopropenes led us to propose that Cu could trap the transient aryne and template its arylation to pyrazoles with controllable selectivity^33,34^. While increasing in availability, innovative strategies to prepare arynes continue to evolve, and these captivating species have provided important transformations and complex natural products^35,36^. However, these arynes are highly reactive and known to undergo heteroatom additions in the absence of any catalysts, thus posing an additional challenge to both regio- and chemo-control^37–39^. With this design in mind, we set out to identify Cu catalysts that allow switchable access to either *N*^α^-aryl pyrazole (**3**) or *N*^β^-aryl pyrazole (**4**), while providing both experimental and theoretical support for a unique pathway.

### Reaction development and scope of *N*^α^*-*arylation

As proof of concept, we chose 3-phenylpyrazole (**1a**) and commercial Kobayashi benzyne precursor (*o*-(trimethylsilyl)phenyl triflate; **2a**) to target the regioselective formation of *N-*phenyl pyrazole **3a** (Table 1). Among the various aryne activation strategies, Kobayashi’s mild protocol generates aryne using fluorides^40^. The control experiment in the absence of catalyst (entry 1) affords *N-*aryl pyrazoles as a 1:1 mixture of isomers in a 75% yield, a facile and non-selective background reaction. We reasoned that it would be possible to outcompete this background transformation by using the appropriate Cu and ligand combination. From surveying ligands, we identified the Xantphos ligands as the most promising. In particular, CyXantphos (**L1**) gives both high regioselectivity (16:1 N^α^:N^β^) and reactivity (89%, entry 2). To further promote Cu–pyrazolate formation, we used organic and inorganic bases and found both to benefit selectivity, with 1,1,3,3-tetramethylguanidine (TMG) providing over 20:1 N^α^:N^β^ in selectivity and 92% in yield (entry 3). Similarly, NEt_2_Xantphos (**L2**) furnishes an excellent ratio of **3a** to **4a** (>20:1), although with a lower yield (47%, entry 4). In addition, we identified Josiphos (**L3**–**L4**) to be complementary ligands to Xantphos that also afford desirable reactivity and selectivity (entry 5–6). Increasing the amount of CsF results in lower selectivity, presumably due to faster aryne formation, which participates in the non-selective background transformation. We reason that TMG also helps facilitate the rate of Cu catalysis over the background reaction, not only as a base but also as a dynamic ligand to break up Cu dimers or Cu–pyrazole resting states. In essence, matching the rate of aryne formation with Cu–aryne complexation results in a more selective arylation by outcompeting background processes.

**Table 1:**
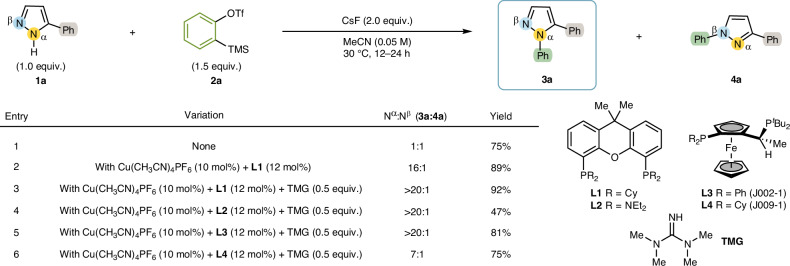
Reaction optimization of *N*^α^-selective arylation

Reactions were performed on a 0.050 mmol scale. Yield and regioselectivity (N^α^:N^β^) were determined by ^1^H NMR analysis of the reaction mixture. MeCN, acetonitrile.

**Fig. 2:**
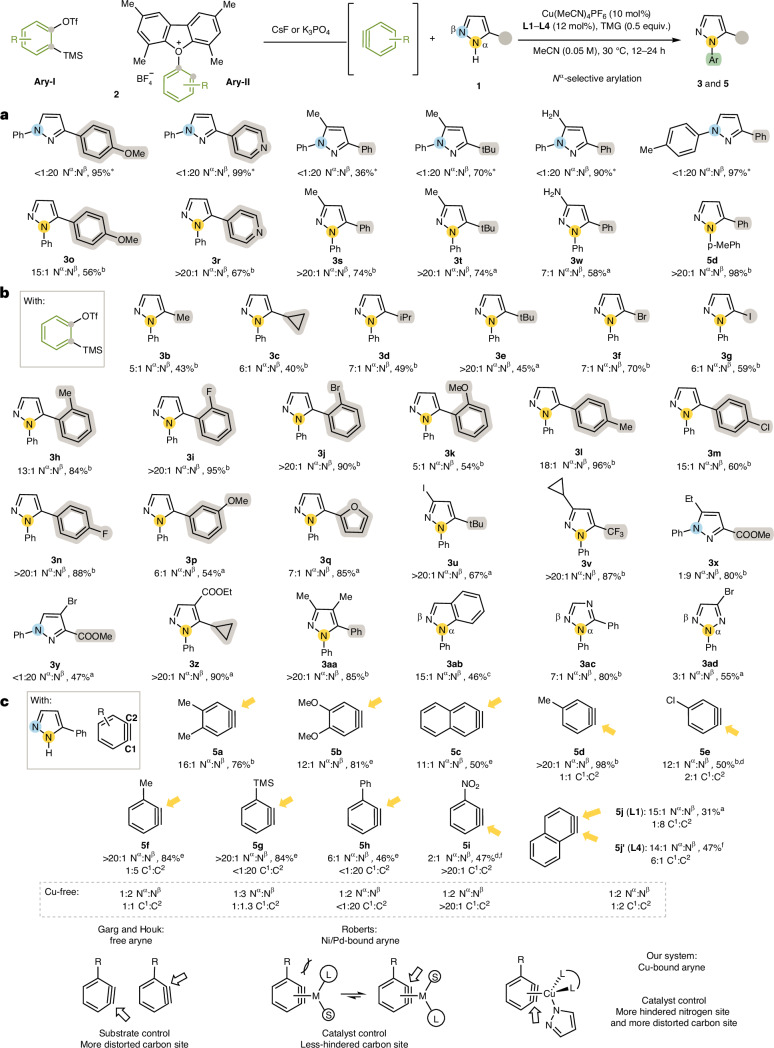
*N*^α^-arylation. **a**, Comparing our chemistry with reported cross-couplings. Asterisks indicate literature reports^24,41–43^. **b**, The scope of pyrazoles. **c**, The scope of arynes and state-of-the-art models for predicting aryne regioselectivity. Yellow arrows indicate aryne reaction site. Standard reaction conditions: **1** (0.050 mmol), **Ary-I** (0.075–0.15 mmol), Cu(CH_3_CN)_4_PF_6_ (10 mol%), **L** (12 mol%), TMG (50 mol%), CsF (2.0–4.0 equiv.), MeCN (1.0 ml), 12–24 h, 30 °C. ^a^With **L1**. ^b^With **L2**. ^c^With bis(dicyclohexylphosphino)methane ligand. ^d^With **Ary-II** (0.075 mmol), K_3_PO_4_ (2.0 equiv.) instead. ^e^With **L3**. ^f^With **L4**. Isolated yield of **3** and **5**. Regioselectivity (*N*^*α*^*:N*^*β*^) was determined from ^1^H NMR analysis of the reaction mixture. See the Supplementary Information (product characterization) for full reaction conditions for each substrate.

**Fig. 3:**
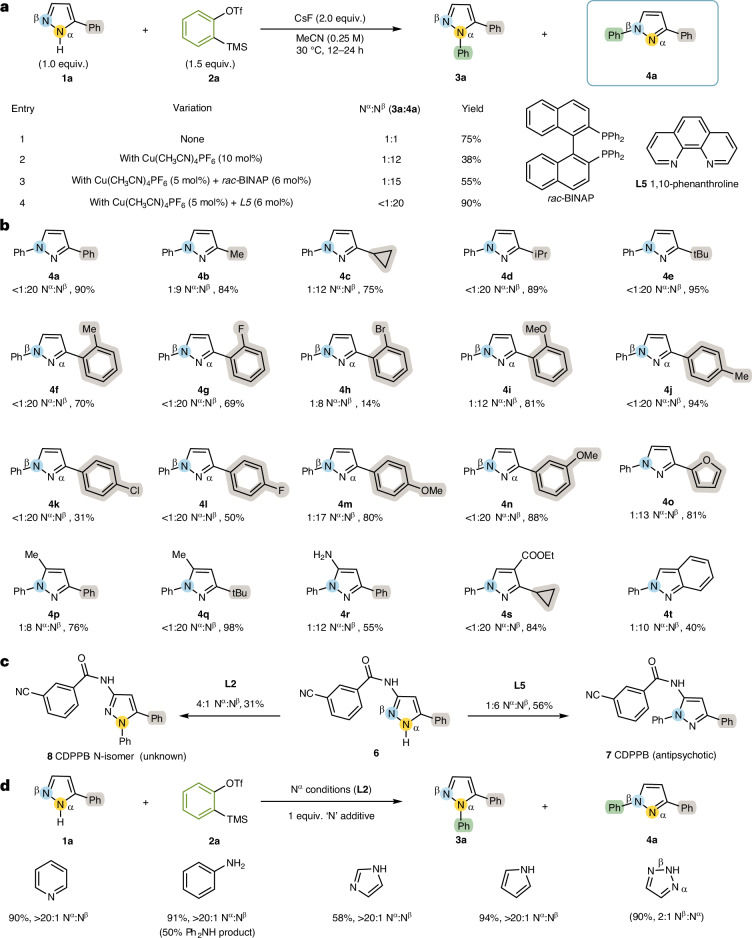
*N*^β^-arylation. **a**, Reaction optimization. **b**, The scope. Standard reaction conditions: **1** (0.10 mmol), **2a** (0.15–0.30 mmol), Cu(CH_3_CN)_4_PF_6_ (5 mol%), **L5** (6 mol%), CsF (2.0–4.0 equiv.), MeCN (0.4 ml), 12–24 h, 30 °C. Isolated yield of **4**. Regioselectivity (N^α^:N^β^) was determined from ^1^H NMR analysis of the reaction mixture. See the Supplementary Information (product characterization) for full reaction conditions for each substrate. **c**, Using regiodivergent access to synthesize the bioactive compound. **d**, The reaction compatibility test.

With the N^α^-conditions, we investigated the arylation of 28 *N-*heterocycles (**1**) with benzyne precursor **2a**. Figure 2a highlights direct comparisons between literature-reported examples and our arylation conditions. Through the use of Cu–diamine complexes, Buchwald provided seminal access to *N*^β^-aryl pyrazoles under mild conditions^24^. Subsequent studies using both Cu and Pd also favour arylation of the less-hindered nitrogen^41–43^. By stark contrast, subjecting these exact pyrazoles to Cu–aryne catalysis generates the opposite constitutional isomers (**3o**, **3r**, **3s**, **3t**, **3w** and **5d**). In addition to the reported substrates, alkyl-substituted pyrazoles were found to be tolerant (Figs. 2b and 3b–e). We observe an increase in regioselectivity as the alkyl substituent increases in size (5:1 to >20:1 N^α^:N^β^). Halogen-containing pyrazoles exhibit modest yields and regioselectivities (**3f** and **3g**). High yields (56–96%) and regioselectivities (13:1 to >20:1 N^α^:N^β^) are obtained for 3-aryl pyrazoles with substituents varying in electronics and sterics (**3h**–**3k** and **3l**–**3p**), with the exception of 2-OMe (**3k**) and 3-OMe (**3p**). Heteroaryl substituents on pyrazoles, such as furyl (**3q**) and pyridyl (**3r**), also tolerate *N*^α^*-*arylation (7:1 and >20:1 N^α^:N^β^, respectively). To study more hindered substrates, we subjected various 3,5-disubstituted pyrazoles (**3s**–**3v**) to N^α^ conditions. Both regioselectivity and reactivity were maintained (67–90%, >20:1 N^α^:N^β^). With an amino pyrazole substrate, hydroamination occurs at N^α^ rather than the amino group (**3w**, 58%, 7:1 N^α^:N^β^). In the case of pyrazoles with 3-ester substituents, arylation at N^β^ (positioned further away from the ester group) is favoured (**3x**–**3y**, 1:9 to <1:20 N^α^:N^β^). We postulate that chelation between the ester and N^α^ with a Cu catalyst inverts selectivity to favour N^β^-aryl pyrazoles. With an isomeric 4-ester pyrazole (where the ester group position prohibits chelation), we observe formation of the corresponding *N*^*α*^-aryl pyrazole **3z** (>20:1 N^α^:N^β^). Extension to trisubstituted pyrazole and other important *N-*heterocycles provides pyrazole (**3aa**), indazole (**3ab**) and triazoles (**3ac** and **3ad**). While arylation occurs favourably at the more-hindered N^α^ for indazole (when using the bis(dicyclohexylphosphino)methane ligand) and 1,2,4-triazole, the 1,2,3-triazole substrate tends to arylate at the middle nitrogen^44^.

Next, we apply the N^α^ protocol to ten substituted aryne precursors, including eight Kobayashi precursors (**Ary-I**) and two oxonium precursors (**Ary-II**) that can be activated by weak bases^45^ (Fig. 2c). With Xantphos (**L1**–**L2**) and Josiphos ligands (**L3**–**L4**), we find that the *N*^α^-arylation of **1a** encompasses a variety of arynes (**2**) with different electronic and steric profiles. Symmetric benzynes and naphthalyne show high yields and regioselectivities to form C–N bonds with N^α^ (**5a**–**5c**, 50–76%, 11:1 to 16:1 N^α^:N^β^). In the case of unsymmetric arynes, up to four possible constitutional isomers can be generated in one step because of both carbon and nitrogen site selectivity. For unsymmetric arynes, Garg and Houk established a distortion model to predict regioselective attack at the more distorted carbon sites^46^. But coordination of arynes with metals can alter this selectivity. Roberts demonstrated site-selective functionalization of arynes via the use of unsymmetrical ligands with Pd- or Ni-bound arynes to block one carbon site via sterics^47,48^. Here, we show in the absence of Cu catalysts, pyrazole addition to an aryne bearing a methyl or trimethylsilyl group affords poor selectivity for both nitrogen and carbon sites (**5f** and **5g**). By contrast, Cu catalyst **L3** promotes the addition, with increased control for both the pyrazole and the aryne sites to generate **5f** (>20:1 N^α^:N^β^, 1:5 C^1^:C^2^) and **5g** (>20:1 N^α^:N^β^, <1:20 C^1^:C^2^). For an aryne bearing a phenyl group, we observe the formation of **5h** (6:1 N^α^:N^β^, <1:20 C^1^:C^2^), which favours the addition to the more hindered carbon site, in line with the catalyst-free conditions. For an electron-deficient aryne (such as one bearing a nitro group, **5i**), we observe a high selectivity for addition to the C1 position, in line with that observed and predicted for substrate control under the distortion model. While high nitrogen site selectivity is maintained with 4-substituted arynes (**5d** and **5e**), the regiocontrol over carbon sites remains elusive owing to the weak substituent effect^40^. Using free naphthalyne, we observe a mixture of isomers with a slight preference for C2 (1:2 C^1^:C^2^). But, using the Xantphos **L1** ligand, we were able to enhance the intrinsic carbon site selectivity (1:8 C^1^:C^2^) and provide improved *N*^α^*-*selectivity (**5j**). Surprisingly, by changing to the Josiphos **L4** ligand, we observed a complete switch to favour the C1 site-generating isomer **5j′** (6:1 C^1^:C^2^). In both cases, high nitrogen selectivity is observed. Together, these results highlight the promising ability of Cu–aryne catalysis to provide complementary control to Houk/Garg’s use of free arynes and Robert’s Pd/Ni–aryne complexes.

### Reaction development and scope of *N*^β^*-*arylation

Compared with the control experiment (Fig. 3a, entry 1), addition of Cu(MeCN)_4_PF_6_ salt dramatically increases *N*^β^-selectivity (entry 2). We sought to enhance this regiocontrol through ligands. Applying *rac-*BINAP increases the selectivity for N^β^ (entry 3); the use of 1,10-phenanthroline (**L5**) affords excellent control (<1:20 N^α^:N^β^) favouring **4a** (90%, entry 4).

Next, we illustrate the arylation of N^β^ using complementary conditions featuring ligand **L5** (Fig. 3b). With alkyl-substituted pyrazoles, we observe a similar trend in selectivity to N^α^ conditions. The regioselectivity increases in the order of 3-Me (**4b**, 1:9 N^α^:N^β^), 3-cyclopropyl (**4c**, 1:12 N^α^:N^β^), 3-^*i*^Pr (**4d**, <1:20 N^α^:N^β^) and 3-^*t*^Bu (**4e**, <1:20 N^α^:N^β^). Aryl- (**4f**–**4n**, 14–94%, 1:8 to <1:20 N^α^:N^β^) and furyl- (**4o**, 81%, 1:13 N^α^:N^β^) substituted pyrazoles afford the corresponding *N*^β^-aryl product with moderate-to-excellent regioselectivities and yields. Compared with the N^α^ conditions, the regioselectivity of disubstituted pyrazoles shows a switch from the more hindered N^α^ to N^β^, which has the smaller neighbouring substituent (**4p**–**4s**, 55–98%, 1:8 to <1:20 N^α^:N^β^). Indazole provides a 40% yield and 1:10 regioselectivity towards the less-hindered N^β^ (**4t**). We observe relatively poor compatibility with electron-deficient pyrazoles under N^β^ conditions. In comparison, similar regioselectivity and higher yields are generally found under uncatalysed reactions with these pyrazoles. Finally, to apply the orthogonal arylation conditions, we prepared a pyrazole-containing antipsychotic drug, CDPPB (**7**), and its constitutional isomer CDPPB-*N-*isomer (**8**), containing either an *N*^α^*-* or *N*^β^-phenyl motif^49^ (Fig. 3c). While the known allosteric modulator **7** can be selectively constructed through the coupling between pyrazole **6** and benzyne precursor **2a** under N^β^ conditions (56%, 1:6 N^α^:N^β^), our N^α^ conditions switch the site selectivity and furnish arylation product **8** selectively (31%, 4:1 N^α^:N^β^).

While the Cu catalyst has shown promise in controlling the nitrogen site selectivity on unsymmetric pyrazoles, we wondered how it would perform in the presence of additional N-sites^3^ (Fig. 3d). By running the standard reaction conditions with one equivalent of pyridine, aniline, imidazole and pyrrole as additives, we observe the maintenance of reactivity and selectivity favouring the N^α^ site, even though a side product of diphenyl amine is observed in the case of aniline. When running the standard reaction conditions with 1,2,3-triazole as an additive, we observe *N-*aryl triazole as the major product, suggesting the addition of triazole to benzyne outcompeting that of pyrazole. In general, our Cu system shows a favourable arylation towards the N–N linkage of a heterocycle.

### Origin of regiodivergence with steric versus electronic ligand effects

**Fig. 4:**
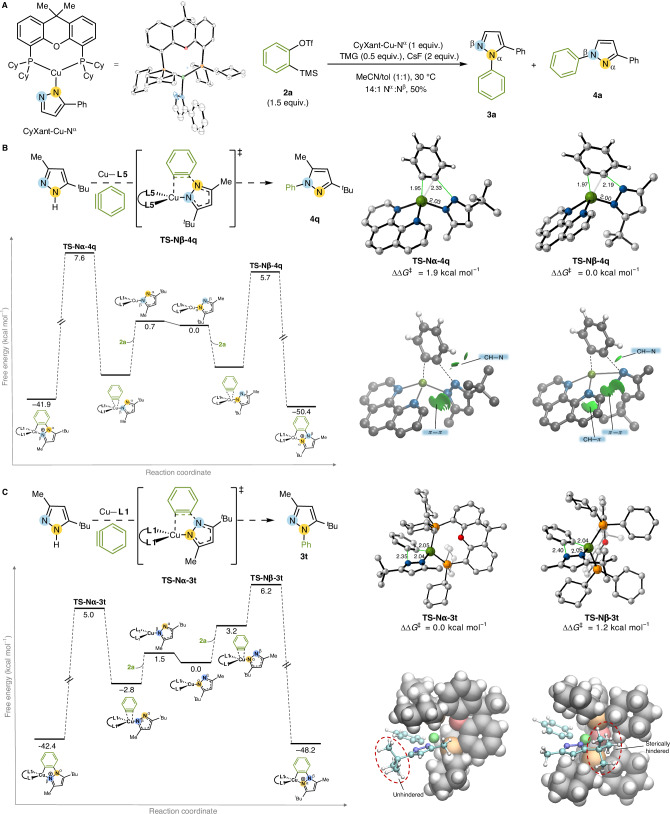
Origin of site selectivity. **a**, The synthesis of a Cu–pyrazolate metallotautomer and its stochiometric reactivity. **b**, Electronic-controlled regioselectivity. Left: energy diagram of ligand **L5** depicting the relative barriers of the regiodivergent aminocupration TSs relative to the pyrazolate tautomers. Top right: the lowest energy TSs of the five-centred aminocupration step leading to both *N*^α^- and *N*^β^-arylation at the M06-2X/6-311+G(d,p) PCM (acetonitrile)//M06-2X/6-31G(d) level of theory. Bottom right: non-covalent interaction plots depicting intermolecular dispersion interactions (shown in green) between pyrazole and Cu–**L5** and **2a** (isosurface 0.009) (ref. ^57^). **c**, Steric-controlled regioselectivity. Left: energy diagram of ligand **L1** (CyXantphos) depicting the relative barriers of the regiodivergent aminocupration TSs relative to the pyrazolate tautomers. Top right: the structure of the lowest energy TSs of the five-centred aminocupration step leading to both *N*^α^- and *N*^β^-arylation. Bottom right: space filling models illustrating the steric constraints of each TS. TS, transition state; tol, toluene, ΔΔ*G*^‡^, the difference in the activation energy between two catalytic pathways.

To investigate the key regio-determining step, we prepared a Cu–pyrazolate (CyXant-Cu-N^α^) using mesitylcopper, **L1** and pyrazole **1a**^50^. This Cu–pyrazolate was isolated and characterized by X-ray crystallography and represents a rare mononuclear Cu(I) complex with a non-bridging pyrazole ligand^51^. Next, we subjected CyXant-Cu-N^α^ to benzyne **2a** and identified the formation of **3a**. In this experiment, we observed a similar selectivity (14:1 N^α^:N^β^ versus 20:1 N^α^:N^β^) with a slightly diminished efficiency (50% versus 92% yield) (Fig. 4a). This stoichiometric experiment supports the feasibility of Cu–pyrazolate as a viable intermediate in coupling with transient arynes.

To further probe the ligand effects on switchable site selectivity, we used density functional theory calculations to model the reaction of unsymmetric 3*-t*-butyl-5-methyl pyrazole with benzyne precursor **2a** under N^α^- and N^β^-conditions. Density functional theory calculations were performed at the M06-2X /6-311+G(d,p) PCM (MeCN)//M06-2X/6-31G(d) level of theory, benchmarked against eight different levels of theory^52–57^. The lowest energy pathway for both ligand systems proceeds through a five-centred 1′,6′-migratory insertion aminocupration transition state^10^. There are two rapidly interconverting pyrazolate metallotautomers (Cu–N^α^ versus Cu–N^β^). Consistent with the Curtin–Hammett principle, the product ratio is independent of the relative ground state energies of the pyrazolate metallotautomers and is determined by the relative energies of the aminocupration transition states leading to each isomer^44^ (Fig. 4b,c). For the reaction of the unsymmetric pyrazole leading to **4q** (*N*^β^-aryl pyrazole) modelled with **L5** (1,10-phenanthroline), formation of the C–N bond is favoured at the N^β^ position by 1.9 kcal mol^−1^ (TS-Nβ-4q) (Fig. 4b), consistent with the experimentally observed regioselectivity (1:20 N^α^:N^β^). Notable differences in these two transition structures are (1) the key C–N bond-forming distance (2.19 Å versus 2.33 Å) and (2) the position of the *t-*butyl group relative to the phenanthroline *π* system. As such, the factors that dictate the extent of stabilization are derived from a dispersive CH–*π* interaction only present in **TS-Nβ-4q** and a stronger CH^…^N interaction (Fig. 4b, green areas in the dispersion maps). The significance of these interactions in selectivity is supported by an energy decomposition analysis, which shows that interaction energy favours the transition structure leading to the major regioisomer by 2.3 kcal mol^−1^. While the Cu–pyrazolate system intrinsically favours *N*^β^-arylation (1:12 N^α^:N^β^), this effect is further enhanced by increased dispersion interactions in the presence of **L5**, which accounts for the increase in both regioselectivity and yield. By contrast, the bulkier CyXantphos ligand (**L1**) can overcome this inherent selectivity to favour *N*^α^-arylation via steric control. The 1′,6′-migratory insertion transition structure that leads to the formation of the N^α^ regioisomer of product **3t** (C–N bond formation at 2.35 Å) is favoured by 1.2 kcal mol^−1^ (**TS-Nα-3t**) (Fig. 4c), consistent with the experimentally observed regioselectivity (20:1 N^α^:N^β^). Analysis of the key transition structures for the aminocupration steps indicates that the site selectivity is dictated by steric interactions between the *t-*butyl group and the bulky ligand, with the transition structure that avoids this steric clash (**TS-Nα-3t**) being lower in energy (Fig. 4b, space filling models). A distortion–interaction analysis on **TS-Nα-3t** and **TS-Nβ-3t** supports this finding, with both the distortion and Pauli repulsion components favouring the less sterically encumbered **TS-Nα-3t** (see the Supplementary Information for details on the analysis of both **L1**- and **L5**-controlled pathways)^58^. Therefore, regiodivergence is achieved by balancing the steric and electronic environment of the catalytic pocket—with less bulky ligands, such as **L5**, enhancing the intrinsic selectivity of the system for *N*^β^-arylation via more favourable interactions within the pocket. Alternatively, bulkier ligands such as **L1** overcome this inherent selectivity to favour *N*^α^-arylation via steric control.

## Outlook

Despite great progress in catalysts and mechanistic insights, industrial laboratory teams perform thousands of experiments to optimize for specific drug targets; this widely accepted strategy underscores the academic value of providing a conceptual blueprint versus the need for a universal protocol. Since Ullmann’s stoichiometric C–N coupling in 1901 and the discovery of arynes in 1902, both fields have expanded the reach of synthetic methods^36,50,59–63^. By merging these two ideas, we provide a site-selective arylation of pyrazoles, a critical heterocycle in drug discovery. The mechanistic insights from this study lay a foundation for future site- and stereoselective nitrogen-heterocycle transformations. As new precursors and conditions emerge, the chemistry of strained intermediates will continue to advance. Given the vast toolbox of chiral ligands, this study also sets the stage for enantioselective aryne chemistry, including cycloadditions, insertions and beyond.

## Methods

### General procedures of the synthesis of *N*-aryl pyrazoles

In a N_2_-filled glovebox, to a 1-dram vial equipped with a magnetic stir bar was added the copper catalyst (0.005 mmol, 10 mol%), the ligand (0.006 mmol, 12 mol%) and the solvent (1.0 ml, 0.05 M). After stirring for 30 min, pyrazole (0.05 mmol, 1.0 equiv.) and base additive (0.025 mmol, 0.5 equiv.) were added. The resulting mixture was stirred for another 30 min followed by the addition of aryne precursors (0.075 mmol, 1.5 equiv.) and activating reagent (0.1 mmol, 2.0 equiv.). The vial was sealed with a Teflon-lined screw cap and stirred at 30 °C for 12 h inside the glovebox. The reaction was monitored by thin-layer chromatography. If thin-layer chromatography showed a low conversion of pyrazole, additional precursor (0.075 mmol, 1.5 equiv.) and activating reagent (0.1 mmol, 2.0 equiv.) was added, and the resulting mixture was stirred for another 12 h at 30 °C. Upon completion, the reaction mixture was concentrated in vacuo, and the product was obtained by preparative thin-layer chromatography.

## Online content

Any methods, additional references, Nature Portfolio reporting summaries, source data, extended data, supplementary information, acknowledgements, peer review information; details of author contributions and competing interests; and statements of data and code availability are available at 10.1038/s41557-026-02148-z.

## Supplementary information

Supplementary Information Procedural details, synthesis and characterization data, NMR spectra, X-ray crystallographic data and computational studies. Supplementary figures (1–8) and tables (1–24) are included.

Supplementary Data 1 Compressed computational data.

## Data Availability

The data supporting the findings of this study are available within the Article and its Supplementary Information. Crystallographic data for the structure reported in this Article have been deposited at the Cambridge Crystallographic Data Centre, under deposition number CCDC-2384544 (CyXant-Cu-N^α^). Copies of the data can be obtained free of charge and are available via the CCDC at https://www.ccdc.cam.ac.uk/structures.
