# Supplementary material for: Copper-catalysed site-selective arylation of pyrazoles
Source: Nat Chem. 2026 Jun 12;18(8):1487–95. doi: 10.1038/s41557-026-02148-z (PMC13423811; doi:10.1038/s41557-026-02148-z)
Supplement: Supplementary file 1 — Procedural details, synthesis and characterization data, NMR spectra, X-ray crystallographic data and computational studies. Supplementary figures (1–8) and tables (1–24) are included. [file 41557_2026_2148_MOESM1_ESM.pdf]

nature chemistry

Supplementary information https://doi.org/10.1038/s41557-026-02148-z

Copper-catalysed site-selective arylation of pyrazoles

In the format provided by the authors and unedited

Table of Contents

1. Material and Methods 1
2. Reaction Optimization 2
   2.1. Reaction Optimization to Access More Hindered Nitrogen N^{α} 2
   2.2. Reaction Optimization to Access Less Hindered Nitrogen N^{β} 6
   2.3. Intrinsic Regioselectivity of Unsymmetric Pyrazoles under Non-catalytic Conditions . 8
3. General Procedure for Selective Addition of Unsymmetric Pyrazoles to Benzynes 10
   3.1. Procedure A - Addition of Benzynes to N^{α} with Kobayashi Precursor 10
   3.2. Procedure B - Addition of Benzynes to N^{β} with Kobayashi Precursor 11
   3.3. Procedure C - Addition of Benzynes to N^{α} with Smith Precursor 12
4. Product Charaterization 13
5. Synthesis of CDPPB (7) and CDPPB-*N*-isomer (8) 32
6. Synthesis and Reactivity of Cu-pyrazolate 34
7. DFT Calculations 35
   7.1. General Information 35
   7.2. Analysis of the Cy-XantPhos (L1)-Controlled N^{α}-Arylation 36
   7.3. Analysis of 1,10-Phenanthroline (L5)-Controlled N^{β}-Arylation 44
   7.4. Summary 48
8. Product Isomerization Study 50
9. Reaction Compatibility Test 51
10. X-Ray Crystallography Data 52
11. General Procedure to Prepare Aryne Precursors 71
12. NMR Spectra 73
13. References and Notes 147

1. Material and Methods

Commercial reagents were purchased from Combi-Block, Sigma Aldrich, Strem, Alfa Aesar, Ambeed, Synthonix, Synquest, Oakwood, or TCI and used without further purification. All pyrazole starting materials are from commercial sources without further purification. Acetonitrile (MeCN) (anhydrous >99.9% from Sigma Aldrich) were degassed and stored over 3Å MS within a N_2 filled glove box. All experiments were performed in oven-dried or flame-dried glassware under an atmosphere of N_2 or in a glove box with a N_2 atmosphere. Reactions were monitored using thin-layer chromatography (TLC; EMD Silica Gel 60 F_{254} plates). Visualization of the developed plates was performed under UV light (254 nm) or with a $KMnO_4$ stain. Organic solutions were concentrated under reduced pressure on a Büchi rotary evaporator. Purification and isolation of products was performed via silica gel chromatography (flash column chromatography or preparative thin-layer chromatography). Column chromatography was performed with Silicycle Silia-P Flash Silica Gel using glass columns. ^1H, ^{13}C, ^{19}F NMR spectra and NOE experiments were recorded on a Bruker AVANCE-600, GN-500 or DRX-400 spectrometer. ^1H NMR spectra were internally referenced to the residual solvent signal. ^{13}C NMR spectra were internally referenced to the residual solvent signal. Data for ^1H NMR are reported as follows: chemical shift (δ ppm), multiplicity (s = singlet, d = doublet, t = triplet, q = quartet, m = multiplet, br = broad), coupling constant (Hz), integration. Data for ^{13}C and ^{19}F NMR are reported in terms of chemical shift (δ ppm). Infrared (IR) spectra were obtained on a Nicolet iS5 FT-IR spectrometer equipped with an iD5 ATR accessory and are reported in terms of frequency of absorption (cm^{-1}). High resolution mass spectra (HRMS) were obtained by the University of California, Irvine Mass Spectrometry Center on a Micromass 70S-250 Spectrometer (EI) or an ABI/Sciex QStar Mass Spectrometer (ESI).

2. Reaction Optimization

2.1. Reaction Optimization to Access More Hindered Nitrogen N^{α}

General Procedure for Table 1-8: In a N_2 filled glovebox, to a 1-dram vial equipped with a magnetic stir bar was added Cu catalyst (2.5-10 mol%), phosphine ligand and acetonitrile. After stirring for 30 min, pyrazole **1a** (0.05 mmol, 1.0 equiv.) and base (0.2-1.0 equiv.) were added. The resulting mixture was stirred for another 30 min followed by the addition of 2-(trimethylsilyl)phenyl trifluoromethanesulfonate (**2a**) (1.0-1.5 equiv.) and CsF (2.0-4.0 equiv.). The vial was sealed with a Teflon-lined screw cap and stirred at 30 °C for 12-24 hours. The mixture was concentrated in vacuo. Regioselectivity (rr) was determined by ^{1}H NMR analysis of the crude mixture. Reaction yield (%) was determined by ^{1}H NMR analysis of the crude mixture with internal standard trimethoxy benzene.

Table 1. Evaluation of F Source

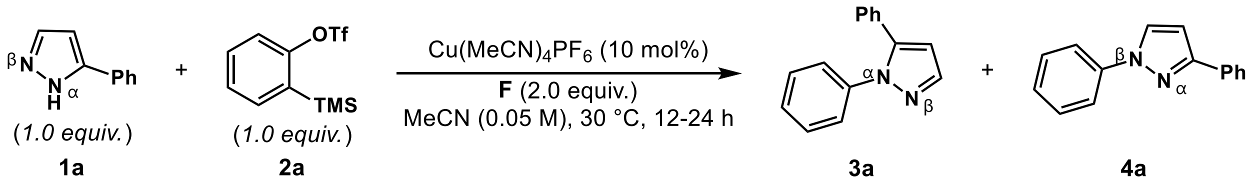

Entry	F source	*yield, N^{α}:N^{β}* (3a:4a)
1	CsF	38%, 1:12
2	TBAT	30%, 1:6
3	KF	no reaction
4	KMAF	no reaction
5	AgF	no reaction

Table 2. Evaluation of Base Additive

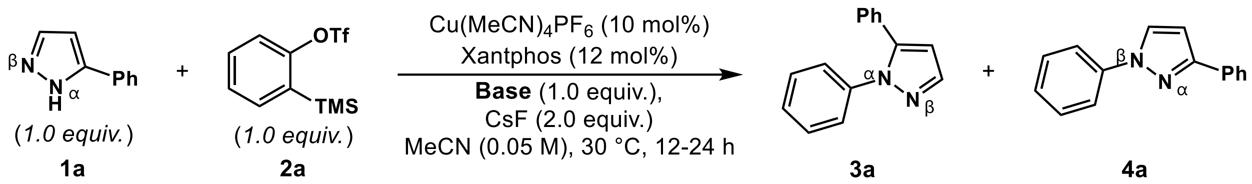

Entry	Base	$N^{\alpha}:N^{\beta}$ (3a:4a)
1	none	24%, 1:1
2	LiOiPr	40%, 1.8:1
3	LiOAc	32%, 1.2:1
4	Cs2CO3	61%, 1.8:1
5	K_3PO_4	52%, 2:1
6	DBU	22%, 1.3:1
**7**	**TMG**	**50%, 10:1**
8	TMG (w/o catalyst)	8%, 1:1.4

Table 3. Evaluation of Ligand

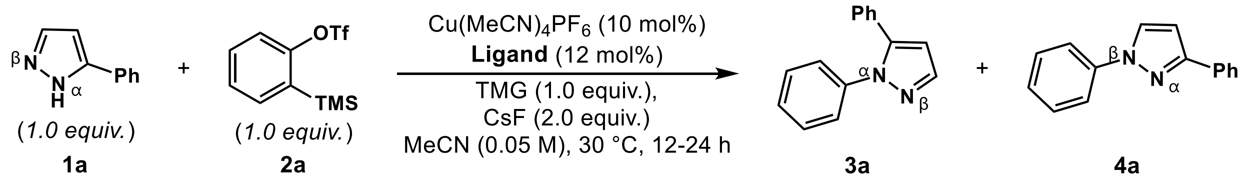

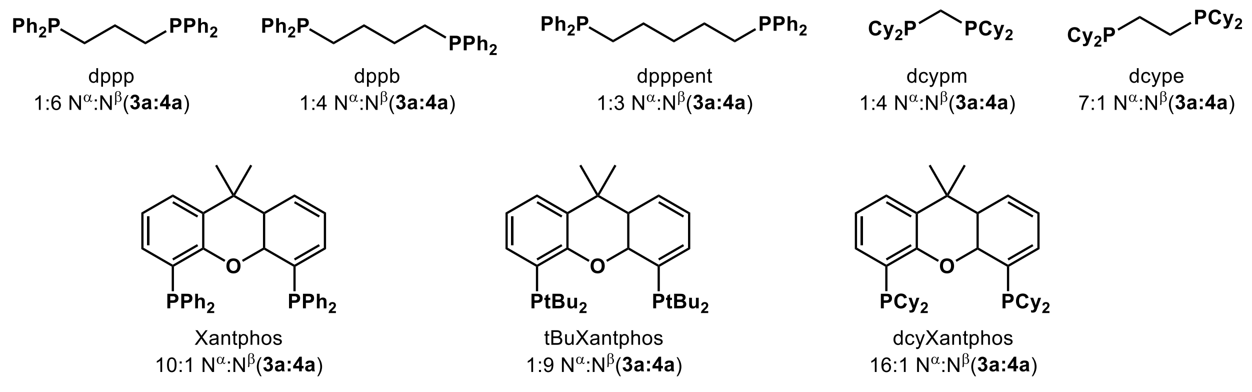

Table 4. Evaluation of CsF Loading and Reaction Concentration

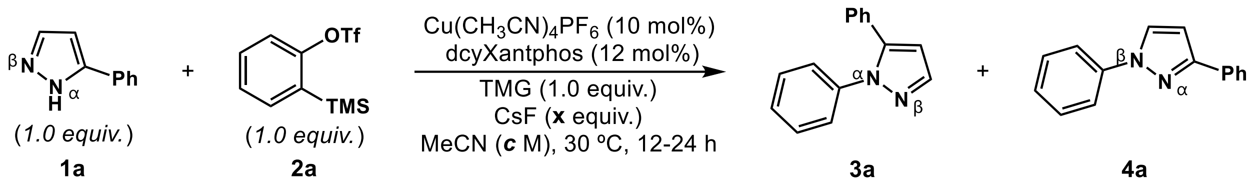

Entry	CsF/concentration	Yield, $N^{\alpha}:N^{\beta}$ (3a:4a)
1	**x** = 2.0, ***c*** = 0.05	60%, 16:1
2	**x** = 2.0, ***c*** = 0.025	38%, 10:1
3	**x** = 2.0, ***c*** = 0.013	51%, 15:1
4	**x** = 1.0, ***c*** = 0.05	35%, 10:1
5	**x** = 3.0, ***c*** = 0.05	46%, 12:1
6	**x** = 4.0, ***c*** = 0.05	10%, 4:1

Table 5. Evaluation of TMG Loading and Catalyst Loading

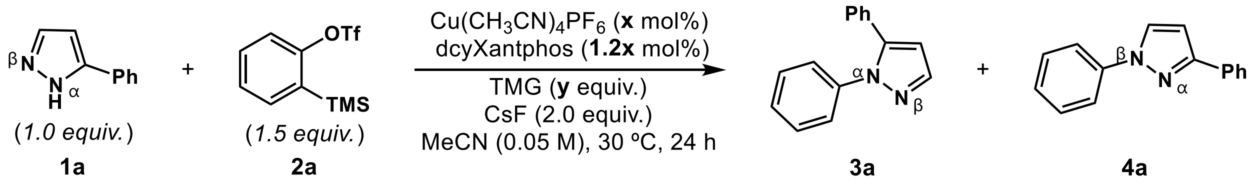

Entry	catalyst loading/TMG	Yield, $N^{\alpha}:N^{\beta}$ (3a:4a)
1	**x** = 10, **y** = 1.0	95%, >20:1
**2**	**x = 10, y = 0.5**	**92%, >20:1**
3	**x** = 10, **y** = 0.2	90%, 16:1
4	**x** = 10, **y** = 0	89%, 16:1
5	**x** = 5, **y** = 0.5	70%, 12:1
6	**x** = 5, **y** = 0	87%, 9:1
7	**x** = 2.5, **y** = 0.5	51%, 5:1
8	**x** = 2.5, **y** = 0	67%, 4:1
9	**x** = 10, K_3PO_4 instead of TMG (**y** = 2.0)	82%, 10:1

Table 6. Evaluation of Ligand for Smaller Substituents on Pyrazoles

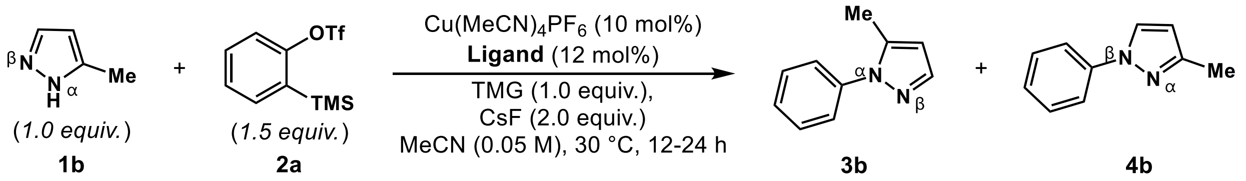

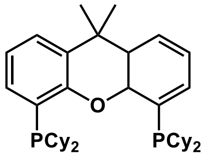

dcyXantphos
1:1 N^{α}:N^{β}(**3b**:**4b**)

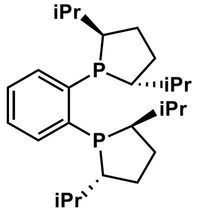

iPr-DuPhos
1.5:1 N^{α}:N^{β}(**3b**:**4b**)

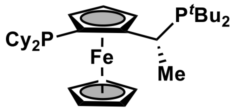

SL-J009-1
3.5:1 N^{α}:N^{β}(**3b**:**4b**)

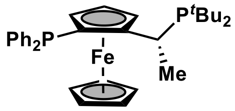

SL-J002-1
1.7:1 N^{α}:N^{β}(**3b**:**4b**)

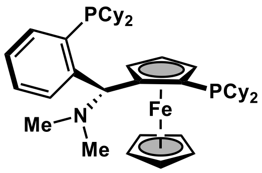

SL-T002-1
1.2:1 N^{α}:N^{β}(**3b**:**4b**)

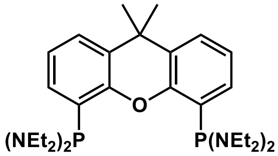

Et$_2$N-Xantphos
4:1 N^{α}:N^{β}(**3b**:**4b**)

Table 7. Evaluation of Base Additive for Smaller Substituents on Pyrazoles

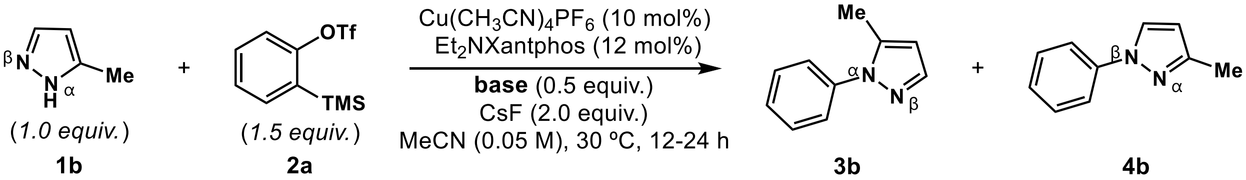

Entry	Base	Yield, N^{α}:N^{β} (3b:4b)
1	LiOiPr	64%, 3:1
2	LiOAc	63%, 1:1
3	Cs_2CO_3	57%, 3:1
4	K_3PO_4	49%, 3:1
5	iPr_2EtN	32%, 1:1
6	Barton's Base	24%, 2:1
7	no base	59%, 4:1

Table 8. Evaluation of Ligand for Substituted Aryne Precursors

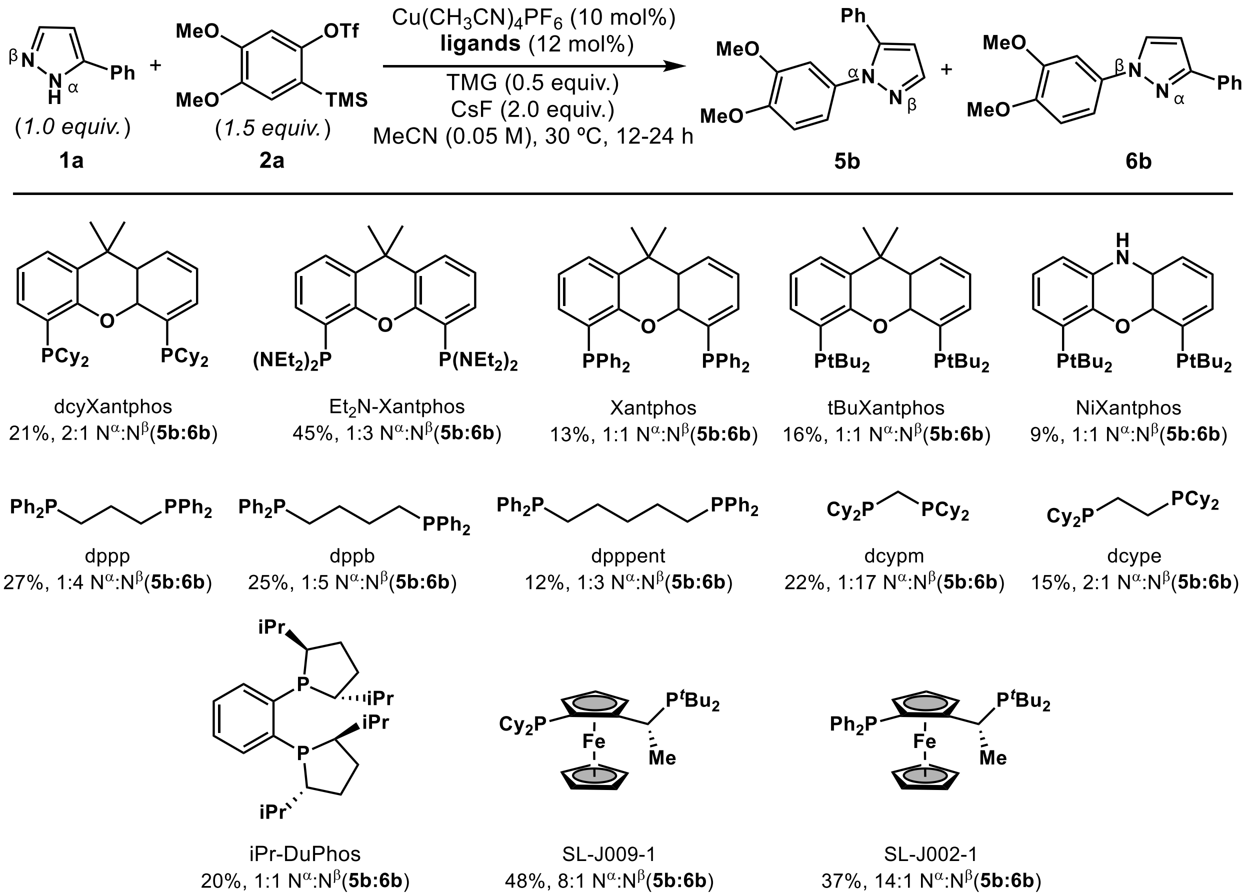

2.2. Reaction Optimization to Access Less Hindered Nitrogen N^{β}

General Procedure for Table 9-11: In a N_2 filled glovebox, to a 1-dram vial equipped with a magnetic stir bar was added $[Cu(CH_3CN)_4]PF_6$ (2.5-10 mol%), ligand (3-12 mol%) and acetonitrile. After stirring for 30 min, pyrazole **1a** (0.1 mmol, 1.0 equiv.), 2-(trimethylsilyl)phenyl trifluoromethanesulfonate (**2a**) (0.15 mmol, 1.5 equiv.) and CsF (0.2 mmol, 2.0 equiv.) were added. The vial was sealed with a Teflon-lined screw cap and stirred at 30 °C for 12-24 hours. The mixture was concentrated in vacuo. Regioselectivity (rr) was determined by ^{1}H NMR analysis of the crude mixture. Reaction yield (%) was determined by ^{1}H NMR analysis of the crude mixture with internal standard trimethoxy benzene.

Table 9. Evaluation of Ligand

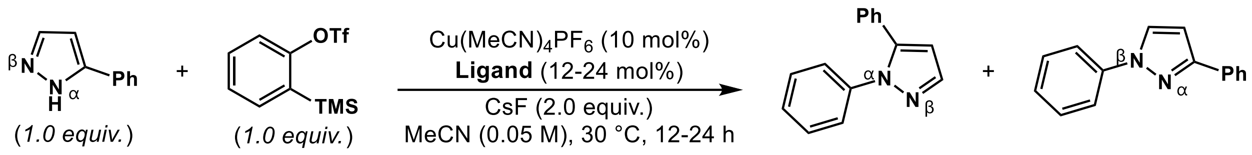

Entry	Ligand	Yield, $N^{\alpha}:N^{\beta}$ (3a:4a)
1	BINAP	55%, 1:15
2	SPhos	59%, 1:20
3	XPhos	45%, 1:20
4	PPh_3	39%, 1:10
5	$P(o\text{-}tol)_3$	55%, 1:20
6	$P(2\text{-}furyl)_3$	81%, 1:10
7	$P(4\text{-}F\text{-}Ph)_3$	40%, 1:4
8	dtbbpy	63%, 1:20
9	bpy	60%, 1:20
10	DavePhos	80%, 1:18
11	tBuDavePhos	38%, 1:17
12	JohnPhos	73%, 1:16
13	BrettPhos	43%, 1:15
14	AdBrettPhos	30%, <1:20
15	AlPhos	52%, <1:20
16	RuPhos	67%, <1:20
17	**1,10-phenanthroline**	**80%, 1:18**

Table 10. Evaluation of Catalyst Loading and Concentration

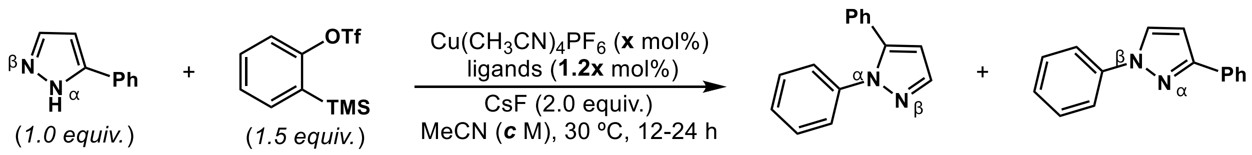

Entry	catalyst Loading/concentration	Yield, $N^{\alpha}:N^{\beta}$ (3a:4a)
1	**x** = 5, ***c*** = 0.05	84%, 1:19
2	**x** = 5, ***c*** = 0.1	87%, <1:20
3	**x** = 5, ***c*** = 0.25	90%, <1:20
4	**x** = 2.5, ***c*** = 0.05	71%, 1:18

Table 11. Evaluation of Cu Source and Ligand

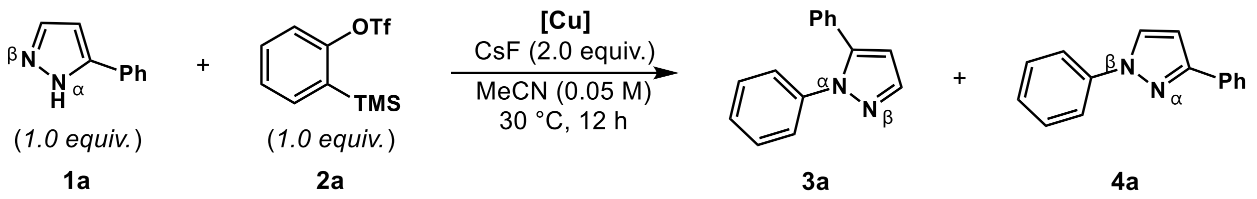

Entry	Derivation	*yield, N^{α} :N^{β} (3a:4a)*
1	CuCN (5 mol%)	24%, 1:8
2	CuI (5 mol%)	26%, 1:9
3	$Cu(OAc)_2$ (5 mol%)	26%, 1:8
4	$Cu(MeCN)_4PF_6$ (5 mol%)	35%, 1:8
5	CuOAc (5 mol%)	20%, 1:9
6	CuCN (5 mol%) + L5 (6 mol%)	40%, 1:17
7	CuI (5 mol%) + L5 (6 mol%)	27%, 1:13
8	$Cu(OAc)_2$ (5 mol%) + L5 (6 mol%)	28%, 1:12
9	$Cu(MeCN)_4PF_6$ (5 mol%) + L5 (6 mol%)	61%, 1:20
10	CuOAc (5 mol%) + L5 (6 mol%)	53%, 1:15

2.3. Intrinsic Regioselectivity of Unsymmetric Pyrazoles under Non-catalytic Conditions

General Procedure for Table 12: In a N_2 filled glovebox, to a 1-dram vial equipped with a magnetic stir bar was added pyrazole **1** (0.1 mmol, 1.0 equiv.), 2-(trimethylsilyl)phenyl trifluoromethanesulfonate (**2a**) (0.15 mmol, 1.5 equiv.), CsF (0.2 mmol, 2.0 equiv.) and acetonitrile. The vial was sealed with a Teflon-lined screw cap and stirred at 30 °C for 12-24 hours. The mixture was concentrated in vacuo. Regioselectivity (rr) was determined by ^{1}H NMR analysis of the crude mixture. Reaction yield (%) was determined by ^{1}H NMR analysis of the crude mixture with internal standard trimethoxy benzene.

Table 12. Summary of Blank Experiments

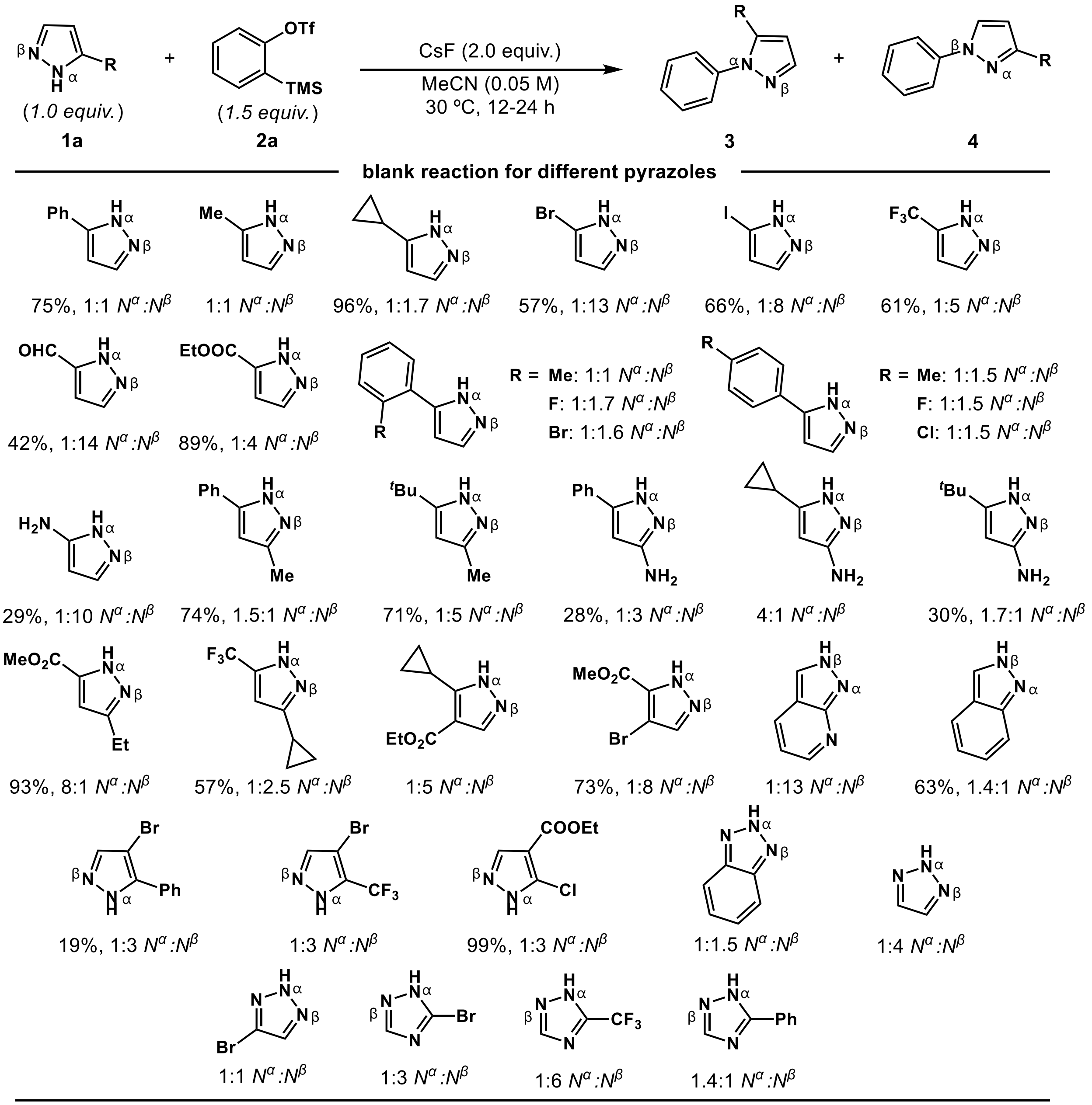

3. General Procedure for Selective Addition of Unsymmetric Pyrazoles to Benzynes

3.1. Procedure A - Addition of Benzynes to N$^\alpha$ with Kobayashi Precursor

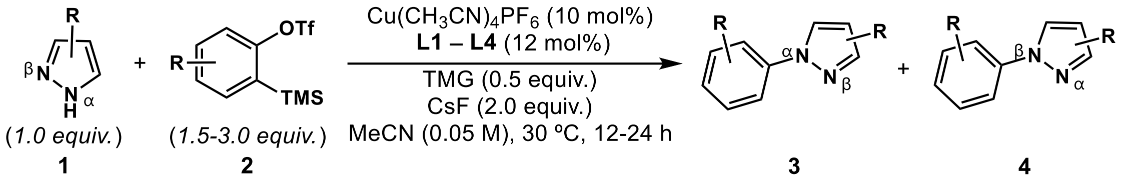

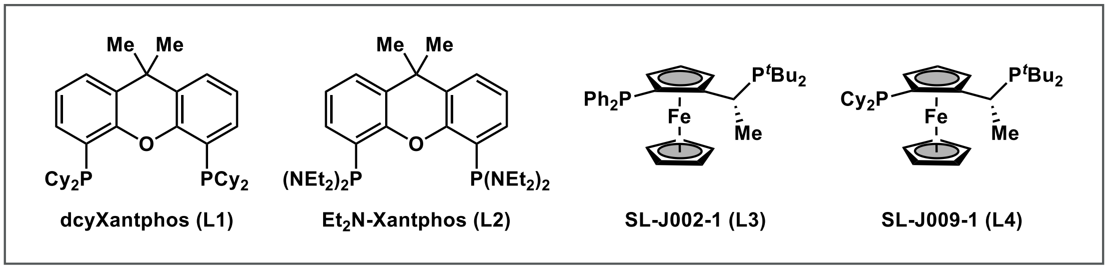

In a N_2 filled glovebox, to a 1-dram vial equipped with a magnetic stir bar was added $[Cu(CH_3CN)_4]PF_6$ (1.9 mg, 0.005 mmol, 10 mol%), phosphine ligand (0.006 mmol, 12 mol%) and acetonitrile (1.0 mL, 0.05 M). After stirring for 30 min, pyrazole **1** (0.05 mmol, 1.0 equiv.) and 1,1,3,3-tetramethylguanidine (TMG, 2.9 mg, 0.025 mmol, 0.5 equiv.) were added. The resulting mixture was stirred for another 30 min followed by the addition of 2-(trimethylsilyl)phenyl trifluoromethanesulfonate (**2**) (0.075 mmol, 1.5 equiv.) and CsF (15.2 mg, 0.1 mmol, 2.0 equiv.). The vial was sealed with a Teflon-lined screw cap and stirred at 30 °C for 12 hours. If TLC shows majority of pyrazole **1** was left, 2-(trimethylsilyl)phenyl trifluoromethanesulfonate (**2**) (0.075 mmol, 1.5 equiv.) and CsF (15.2 mg, 0.1 mmol, 2.0 equiv.) were added again and the mixture was stirred another 12 hours at 30 °C. The mixture was concentrated in vacuo. Regioselectivity (rr) was determined by ^{1}H NMR analysis of the crude mixture. N$^\alpha$- or N$^\beta$-arylated regioisomer was determined by ^{1}H NMR chemical shifts and NOE experiments. Pyrazole product **3** was isolated by preparatory TLC.

3.2. Procedure B - Addition of Benzynes to N^{β} with Kobayashi Precursor

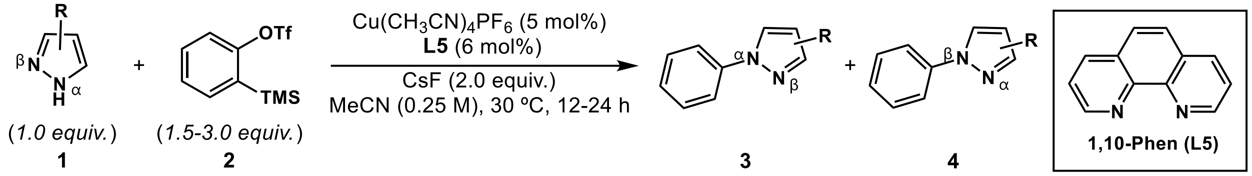

In a N_2 filled glovebox, to a 1-dram vial equipped with a magnetic stir bar was added $[Cu(CH_3CN)_4]PF_6$ (1.9 mg, 0.005 mmol, 5 mol%), 1,10-phenanthroline (**L5**) (1.1 mg, 0.006 mmol, 6 mol%) and acetonitrile (0.4 mL, 0.25 M). After stirring for 30 min, pyrazole **2** (0.1 mmol, 1.0 equiv.), 2-(trimethylsilyl)phenyl trifluoromethanesulfonate (**1**) (0.15 mmol, 1.5 equiv.) and CsF (30.4 mg, 0.2 mmol, 2.0 equiv.) were added. The vial was sealed with a Teflon-lined screw cap and stirred at 30 °C for 12 hours. If TLC shows majority of pyrazole **2** was left, 2-(trimethylsilyl)phenyl trifluoromethanesulfonate (**2**) (0.15 mmol, 1.5 equiv.) and CsF (30.4 mg, 0.2 mmol, 2.0 equiv.) were added again and the mixture was stirred another 12 hours at 30 °C. The mixture was concentrated in vacuo. Regioselectivity (rr) was determined by ^{1}H NMR analysis of the crude mixture. N^{α}- or N^{β}-arylated regioisomer was determined by ^{1}H NMR chemical shifts and NOE experiments. Pyrazole product **4** was isolated by preparatory TLC.

3.3. Procedure C - Addition of Benzynes to N$^{\alpha}$ with Smith Precursor

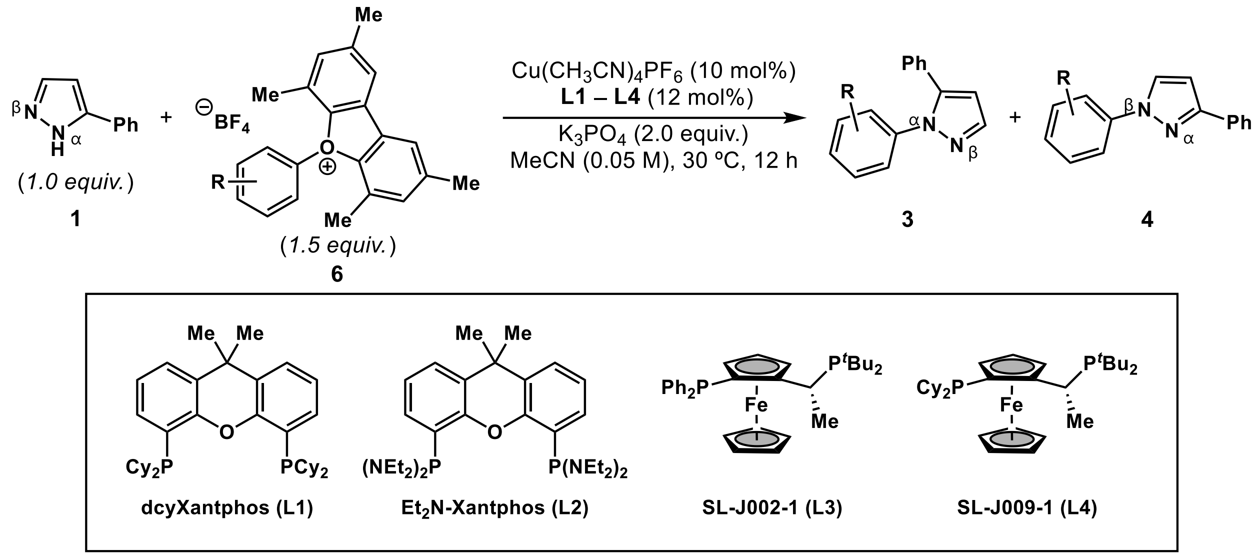

In a N_2 filled glovebox, to a 1-dram vial equipped with a magnetic stir bar was added $[Cu(CH_3CN)_4]PF_6$ (1.9 mg, 0.005 mmol, 10 mol%), phosphine ligand (0.006 mmol, 12 mol%) and acetonitrile (1.0 mL, 0.05 M). After stirring for 30 min, pyrazole **1** (0.05 mmol, 1.0 equiv.) was added. The resulting mixture was stirred for another 30 min followed by the addition of smith oxonium aryne precursor (**6**) (0.075 mmol, 1.5 equiv.) and K_3PO_4 (21.2 mg, 0.1 mmol, 2.0 equiv.). The vial was sealed with a Teflon-lined screw cap and stirred at 30 °C for 12 hours. The mixture was concentrated in vacuo. Regioselectivity (rr) was determined by ^{1}H NMR analysis of the crude mixture. N$^{\alpha}$- or N$^{\beta}$-arylated regioisomer was determined by ^{1}H NMR chemical shifts and NOE experiments. Pyrazole product **3** was isolated by preparatory TLC.

4. Product Charaterization

1,5-Diphenyl-1*H*-pyrazole (3a)

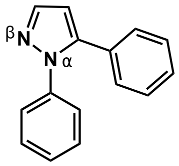

The title compound was synthesized according to general procedure A with **L1** and isolated by preparatory TLC (90:10 hexanes:EtOAc) as yellow oil [10 mg, 92% yield, >20:1 N^{α}:N^{β}].**^{1}H NMR** (600 MHz, $CDCl_3$) δ 7.73 (d, J = 1.9 Hz, 1H), 7.44 – 7.27 (m, 8H), 7.26 – 7.17 (m, 2H), 6.52 (d, J = 1.8 Hz, 1H). **^{13}C NMR** (151 MHz, $CDCl_3$) δ 143.10, 140.41, 140.23, 130.71, 128.99, 128.87, 128.55, 128.29, 127.51, 125.31, 107.95. The NMR data matches literature report.[63]

5-Methyl-1-phenyl-1*H*-pyrazole (3b)

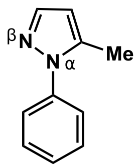

The title compound was synthesized according to general procedure A with **L2** and isolated by preparatory TLC (90:10 hexanes:EtOAc) as white solid [1.3 mg, 43% yield, 4:1 N^{α}:N^{β}]. **^{1}H NMR** (400 MHz, $CDCl_3$) δ 7.58 (s, 1H), 7.51 – 7.42 (m, 4H), 7.39 – 7.35 (m, 1H), 6.20 (s, 1H), 2.36 (s, 3H). **^{13}C NMR** (151 MHz, $CDCl_3$) δ 139.51, 139.30, 138.20, 129.25, 128.21, 125.14, 107.14, 12.52. The NMR data matches literature report.[63]

5-Cyclopropyl-1-phenyl-1*H*-pyrazole (3c)

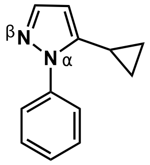

The title compound was synthesized according to general procedure A with **L2** and isolated by preparatory TLC (90:10 hexanes:EtOAc) as yellow oil [3.7 mg, 40% yield, 4:1 N^{α}:N^{β}]. **^{1}H NMR** (500 MHz, $CDCl_3$) δ 7.64 – 7.60 (m, 1H), 7.49 (t, J = 7.8 Hz, 2H), 7.40 (t, J = 7.5 Hz, 1H), 5.99 (d, J = 2.0 Hz, 1H), 1.82 (tt, J = 8.3, 5.1 Hz, 1H), 1.05 – 0.98 (m, 2H), 0.85 – 0.75 (m, 2H).**^{13}C NMR** (151 MHz, $CDCl_3$) δ 146.63, 139.46, 139.38, 129.24, 128.02, 125.07, 102.95, 29.84, 9.21, 7.85. **IR** (ATR): 3011, 2927, 2854, 1597, 1501, 1392, 1030, 926, 763, 695 cm^{-1}. **HRMS** calculated for $C_{12}H_{12}N_2$ $[M+H]^+$ 185.1079, found 185.1088.

5-Isopropyl-1-phenyl-1*H*-pyrazole (3d)

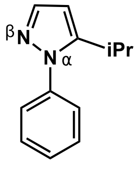

The title compound was synthesized according to general procedure A with **L2** and isolated by preparatory TLC (90:10 hexanes:EtOAc) as white solid [4.6 mg, 49% yield, 7:1 N^2:N^1]. **^{1}H NMR** (499 MHz, $CDCl_3$) δ 7.58 (d, J = 1.8 Hz, 1H), 7.50 – 7.45 (m, 2H), 7.44 – 7.39 (m, 3H), 6.22 (d, J = 1.9 Hz, 1H), 3.06 (sept, J = 6.5 Hz, 1H), 1.19 (d, J = 6.8 Hz, 7H). **^{13}C NMR** (151 MHz, $CDCl_3$) δ 150.45, 140.30, 139.89, 129.26, 128.21,

126.06, 102.92, 25.58, 23.16. **IR** (ATR): 3065, 2966, 1598, 1502, 1395, 1007, 925, 764, 692 cm^{-1}. **HRMS** calculated for $C_{12}H_{14}N_2$ $[M+Na]^+$ 209.1055, found 209.1046.

5-(Tert-butyl)-1-phenyl-1*H*-pyrazole (3e)

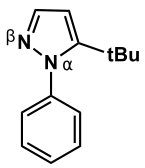

The title compound was synthesized according to general procedure A with **L1** and isolated by preparatory TLC (90:10 hexanes:EtOAc) as white solid [4.5 mg, 45% yield, >20:1 N^{α}:N^{β}]. **^{1}H NMR** (600 MHz, $CDCl_3$) δ 7.52 (d, J = 1.9 Hz, 1H), 7.48 – 7.43 (m, 3H), 7.41 – 7.37 (m, 2H), 6.19 (d, J = 2.0 Hz, 1H), 1.19 (s, 9H). **^{13}C NMR** (151 MHz, $CDCl_3$) δ 153.29, 142.31, 138.60, 129.26, 128.80, 128.76, 103.91, 32.05, 30.90. The NMR data matches literature report.[64]

5-Bromo-1-phenyl-1*H*-pyrazole (3f)

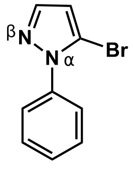

The title compound was synthesized according to general procedure A in 0.1 mmol scale of pyrazole **2** with **L2** and isolated by preparatory TLC (90:10 hexanes:EtOAc) as brown solid [4.5 mg, 70% yield, 5:1 N^{α}:N^{β}]. **^{1}H NMR** (400 MHz, $CDCl_3$) δ 7.68 (d, J = 1.8 Hz, 1H), 7.58 – 7.53 (m, 2H), 7.52 – 7.41 (m, 3H), 6.48 (d, J = 1.8 Hz, 1H). **^{13}C NMR** (151 MHz, $CDCl_3$) δ 141.57 , 139.09 , 129.06 , 128.65, 125.82, 112.94, 110.56. **IR** (ATR): 3132, 2924, 2853, 1598, 1502, 1416, 1394, 1092, 967, 918, 779, 755 cm^{-1}. **HRMS** calculated for $C_9H_7BrN_2$ $[M+H]^+$ 222.9871, found 222.9867.

5-Iodo-1-phenyl-1*H*-pyrazole (3g)

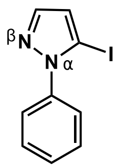

The title compound was synthesized according to general procedure A in 0.1 mmol scale of pyrazole **2** with **L2** and isolated by preparatory TLC (90:10 hexanes:EtOAc) as brown oil [4.1 mg, 59% yield, 10:1 N^{α}:N^{β}]. **^{1}H NMR** (400 MHz, $CDCl_3$) δ 7.69 (d, J = 1.8 Hz, 1H), 7.54 – 7.42 (m, 5H), 6.63 (d, J = 1.8 Hz, 1H). **^{13}C NMR** (151 MHz, $CDCl_3$) δ 142.79, 140.24, 129.01, 128.81, 126.44, 117.58, 80.94. The NMR data matches literature report.[65]

1-Phenyl-5-(o-tolyl)-1*H*-pyrazole (3h)

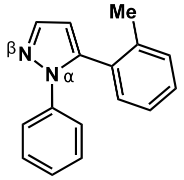

The title compound was synthesized according to general procedure A with **L2** and isolated by preparatory TLC (90:10 hexanes:EtOAc) as colorless oil [9.8 mg, 84% yield, 13:1 N^{α}:N^{β}]. **^{1}H NMR** (600 MHz, CDCl3) δ 7.75 (d, J = 1.8 Hz, 1H), 7.28 (td, J = 7.4, 1.8 Hz, 1H), 7.26 – 7.16 (m, 8H), 6.41 (d, J = 1.8 Hz, 1H), 1.98 (s, 3H). **^{13}C NMR** (151 MHz, CDCl3) δ 142.31, 140.28, 140.20, 137.24, 130.95, 130.67, 130.48, 129.05, 128.88, 126.96, 125.95, 123.72, 108.90, 20.04. I**R** (ATR): 2923, 1599, 1497, 1451, 1380, 960, 924, 758, 690, 651 cm^{-1}. **HRMS** calculated for $C_{16}H_{14}N_2$ $[M+H]^+$ 235.1235, found 235.1242.

5-(2-Fluorophenyl)-1-phenyl-1*H*-pyrazole (3i)

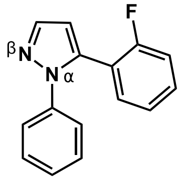

The title compound was synthesized according to general procedure A with **L2** and isolated by preparatory TLC (90:10 hexanes:EtOAc) as white solid [11 mg, 95% yield, >20:1 N^{α}:N^{β}]. **^{1}H NMR** (600 MHz, $CDCl_3$) δ 7.77 (d, J = 1.9 Hz, 1H), 7.36 – 7.27 (m, 6H), 7.20 (td, J = 7.5, 1.8 Hz, 1H), 7.10 (td, J = 7.6, 1.2 Hz, 1H), 7.05 (ddd, J = 9.7, 8.3, 1.2 Hz, 1H), 6.57 (s, 1H). **^{13}C NMR** (151 MHz, $CDCl_3$) δ 159.50 (d, J = 249.9 Hz), 140.41, 140.27, 136.81, 131.47 (d, J = 2.4 Hz), 130.69 (d, J = 8.2 Hz), 129.01, 127.53, 124.42, 124.27 (d, J = 3.8 Hz), 118.97 (d, J = 14.9 Hz), 116.23 (d, J = 21.6 Hz), 109.51 (d, J = 2.0 Hz). **^{19}F NMR** (565 MHz, $CDCl_3$) δ -112.82. I**R** (ATR): 2925, 1598, 1499, 1385, 1229, 1217, 964, 925, 756, 692 cm^{-1}. **HRMS** calculated for $C_{15}H_{11}FN_2$ $[M+H]^+$ 239.0984, found 239.0983.

5-(2-Bromophenyl)-1-phenyl-1*H*-pyrazole (3j)

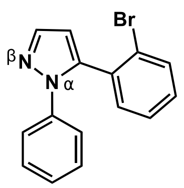

The title compound was synthesized according to general procedure A with **L2** and isolated by preparatory TLC (90:10 hexanes:EtOAc) as colorless oil [13 mg, 90% yield, >20:1 N^{α}:N^{β}]. **^{1}H NMR** (600 MHz, $CDCl_3$) δ 7.77 (d, J = 1.9 Hz, 1H), 7.59 (dd, J = 7.9, 1.3 Hz, 1H), 7.34 – 7.18 (m, 8H), 6.52 (d, J = 1.9 Hz, 1H). **^{13}C NMR** (151 MHz, $CDCl_3$) δ 141.37, 140.03, 133.26, 132.54, 132.35, 130.46, 130.42, 128.90, 127.42, 127.26, 124.23, 124.19, 109.48. The NMR data matches literature report.[66]

5-(2-Methoxyphenyl)-1-phenyl-1*H*-pyrazole (3k)

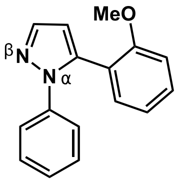

The title compound was synthesized according to general procedure A with **L1** and isolated by preparatory TLC (90:10 hexanes:EtOAc) as colorless oil [6.8 mg, 54% yield, 5:1 N^{α}:N^{β}]. **^{1}H NMR** (600 MHz, $CDCl_3$) δ 7.74 (d, J = 1.8 Hz, 1H), 7.34 (ddd, J = 8.3, 7.5, 1.8 Hz, 1H), 7.30 – 7.26 (m, 3H), 7.26 (d, J = 3.2 Hz, 2H), 7.24 – 7.18 (m, 1H), 6.97 (td, J = 7.5, 1.0 Hz, 1H), 6.79 (dd, J = 8.4, 1.0 Hz, 1H), 6.47 (d, J = 1.8 Hz, 1H), 3.36 (s, 3H). **^{13}C NMR** (151 MHz, $CDCl_3$) δ 156.59, 141.13, 140.05, 140.00, 131.37, 130.54, 128.64, 126.96, 123.73, 120.80, 120.23, 111.38, 108.84, 55.05. **IR** (ATR): 3200, 2928, 2365, 1499, 1385, 1261, 1248, 1024, 925, 790, 756, 691 cm^{-1}.

1-Phenyl-5-(p-tolyl)-1*H*-pyrazole (3l)

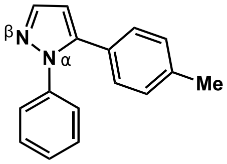

The title compound was synthesized according to general procedure A with **L2** and isolated by preparatory TLC (90:10 hexanes:EtOAc) as white solid [11.2 mg, 96% yield, 18:1 N^{α}:N^{β}]. **^{1}H NMR** (400 MHz, $CDCl_3$) δ 7.73 (d, J = 1.8 Hz, 1H), 7.38 – 7.27 (m, 6H), 7.15 – 7.07 (m, 4H), 6.49 (d, J = 1.9 Hz, 1H), 2.34 (s, 3H). **^{13}C NMR** (151 MHz, $CDCl_3$) δ 143.44, 140.07, 139.99, 138.46, 129.34, 129.05, 128.76, 127.69, 127.55, 125.41, 107.71, 21.39. **IR** (ATR): 3125, 3063, 2921, 1597, 1498, 1384, 961, 925, 823, 785, 761, 701 cm^{-1}. **HRMS** calculated for $C_{16}H_{14}N$ $[M+H]^+$ 235.1235, found 235.1233.

5-(4-Chlorophenyl)-1-phenyl-1*H*-pyrazole (3m)

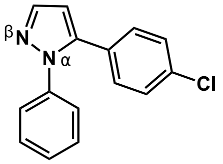

The title compound was synthesized according to general procedure A with **L2** and isolated by preparatory TLC (90:10 hexanes:EtOAc) as white solid [7.6 mg, 60% yield, 15:1 N^{α}:N^{β}]. **^{1}H NMR** (400 MHz, $CDCl_3$) δ 7.74 (d, J = 1.9 Hz, 1H), 7.40 – 7.32 (m, 3H), 7.31 – 7.26 (m, 4H), 7.19 – 7.13 (m, 2H), 6.51 (d, J = 1.9 Hz, 1H). **^{13}C NMR** (151 MHz, $CDCl_3$) δ 142.11, 140.29, 139.72, 134.57, 130.11, 129.23, 129.05, 128.93, 127.97, 125.42, 108.08. **IR** (ATR): 3063, 2923, 2851, 1598, 1499, 1483, 1407, 1382, 1093, 1016, 960, 925, 690, 655, 759, 629 cm^{-1}. **HRMS** calculated for $C_{15}H_{11}ClN_2$ $[M+H]^+$ 255.0689, found 255.0680. The NMR data matches literature report.[67]

5-(4-Fluorophenyl)-1-phenyl-1*H*-pyrazole (3n)

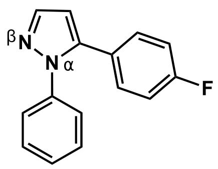

The title compound was synthesized according to general procedure A with **L2** and isolated by preparatory TLC (90:10 hexanes:EtOAc) as white solid [10.5 mg, 88% yield, >20:1 N^{α}:N^{β}]. **^{1}H NMR** (400 MHz, $CDCl_3$) δ 7.73 (d, J = 1.9 Hz, 1H), 7.39 – 7.27 (m, 5H), 7.24 – 7.17 (m, 2H), 7.03 – 6.96 (m, 2H), 6.49 (d, J = 1.9 Hz, 1H). **^{13}C NMR** (151 MHz, $CDCl_3$) δ 162.76 (d, J = 248.8 Hz), 142.29, 140.24, 139.81, 130.72 (d, J = 8.2 Hz), 129.16, 127.85, 126.68 (d, J = 3.4 Hz), 125.40, 115.77 (d, J = 21.6 Hz), 107.93. **^{19}F NMR** (376 MHz, $CDCl_3$) δ -113.18. **IR** (ATR): 3066, 2923, 1594, 1500, 1448, 1383, 1210, 835, 789, 768, 721, 607 cm^{-1}. **HRMS** calculated for $C_{15}H_{11}FN_2$ $[M+H]^+$ 239.0984, found 239.0993. The NMR data matches literature report.[68]

5-(4-Methoxyphenyl)-1-phenyl-1*H*-pyrazole (3o)

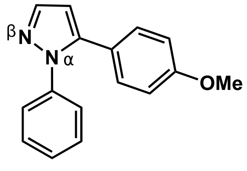

The title compound was synthesized according to general procedure A with **L2** and isolated by preparatory TLC (90:10 hexanes:EtOAc) as colorless oil [7.0 mg, 56% yield, 15:1 N^{α}:N^{β}]. **^{1}H NMR** (600 MHz, $CDCl_3$) δ 7.70 (d, J = 1.8 Hz, 1H), 7.36 – 7.27 (m, 5H), 7.15 (d, J = 8.7 Hz, 2H), 6.83 (d, J = 8.7 Hz, 2H), 6.45 (d, J = 1.8 Hz, 1H), 3.80 (s, 3H). **^{13}C NMR** (151 MHz, $CDCl_3$) δ 159.64, 142.96, 140.38, 130.17, 129.00, 127.44, 125.34, 123.16, 114.03, 107.45, 55.39. The NMR data matches literature report.[67]

5-(3-Methoxyphenyl)-1-phenyl-1*H*-pyrazole (3p)

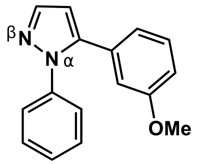

The title compound was synthesized according to general procedure A with **L1** and isolated by preparatory TLC (90:10 hexanes:EtOAc) as yellow oil [6.8 mg, 54% yield, 6:1 N^{α}:N^{β}]. **^{1}H NMR** (600 MHz, $CDCl_3$) δ 7.72 (d, J = 1.9 Hz, 1H), 7.39 – 7.28 (m, 5H), 7.21 (t, J = 7.9 Hz, 1H), 6.85 (ddd, J = 8.3, 2.6, 0.9 Hz, 1H), 6.82 (ddd, J = 7.6, 1.6, 0.9 Hz, 1H), 6.75 (dd, J = 2.6, 1.6 Hz, 1H), 6.52 (d, J = 1.9 Hz, 1H), 3.67 (s, 3H). **^{13}C NMR** (151 MHz, $CDCl_3$) δ 159.54, 143.03, 140.35, 140. 21, 131.88, 129.65, 129.03, 127.64, 125.41, 121.35, 114.25, 114.21, 107.94, 55.29. The NMR data matches literature report.[66]

5-(Furan-2-yl)-1-phenyl-1*H*-pyrazole (3q)

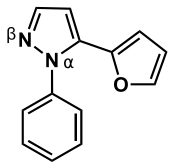

The title compound was synthesized according to general procedure A with **L1** and isolated by preparatory TLC (80:20 hexanes:EtOAc) as brown oil [8.9 mg, 85% yield, 7:1 N^{α}:N^{β}]. **^{1}H NMR** (500 MHz, $CDCl_3$) δ 7.69 (d, J = 1.9 Hz, 1H), 7.48 – 7.40 (m, 5H), 7.39 (dd, J = 1.8, 0.6 Hz, 1H), 6.67 (d, J = 1.9 Hz, 1H), 6.33 (dd, J = 3.4, 1.8 Hz, 1H), 5.97 (d, J = 3.4 Hz, 1H). **^{13}C NMR** (151 MHz, $CDCl_3$) δ 144.69 (s), 142.63 (s), 140.41 (s), 140.38 (s), 134.50 (s), 129.17 (s), 128.58 (s), 125.96 (s), 111.34 (s), 108.89 (s), 106.36 (s). **IR** (ATR): 3128, 2923, 2852, 1501, 1393, 1218, 1071, 977, 926, 763, 694 cm^{-1}. **HRMS** calculated for $C_{13}H_{10}N_2O$ $[M+H]^+$ 211.0871, found 211.0865.

4-(1-Phenyl-1*H*-pyrazol-5-yl)pyridine (3r)

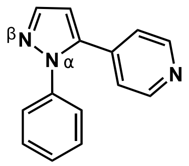

The title compound was synthesized according to general procedure A with **L2** and isolated by preparatory TLC (80:20 hexanes:EtOAc) as yellow oil [7.4 mg, 67% yield, >20:1 N^{α}:N^{β}]. **^{1}H NMR** (600 MHz, $CDCl_3$) δ 8.55 (d, J = 5.3 Hz, 2H), 7.78 (d, J = 1.9 Hz, 1H), 7.46 – 7.37 (m, 3H), 7.33 – 7.27 (m, 2H), 7.23 – 7.18 (m, 2H), 6.70 (d, J = 1.9 Hz, 1H). **^{13}C NMR** (151 MHz, $CDCl_3$) δ 148.50, 140.90, 139.73, 139.68, 139.57, 129.54, 128.62, 125.56, 123.22, 109.42. **IR** (ATR): 3365, 2923, 1601, 1498, 1386, 924, 763, 693cm^{-1}. **HRMS** calculated for $C_{14}H_{11}N_3$ $[M+H]^+$ 222.1031, found 222.1020.

3-Methyl-1,5-diphenyl-1*H*-pyrazole (3s)

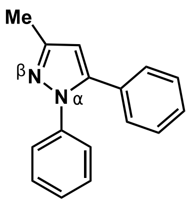

The title compound was synthesized according to general procedure A with **L2** and isolated by preparatory TLC (90:10 hexanes:EtOAc) as colorless oil [8.7 mg, 74% yield, >20:1 N^{α}:N^{β}]. **^{1}H NMR** (400 MHz, $CDCl_3$) δ 7.35 – 7.17 (m, 11H), 6.32 (s, 1H), 2.40 (s, 3H). **^{13}C NMR** (151 MHz, $CDCl_3$) δ 149.35, 144.22, 139.54, 130.37, 129.10, 128.80, 128.62, 128.52, 127.67, 125.41, 107.90, 13.47. The NMR data matches literature report.[63]

5-(Tert-butyl)-3-methyl-1-phenyl-1*H*-pyrazole (3t)

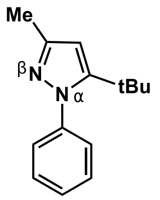

The title compound was synthesized according to general procedure A with **L1** and isolated by preparatory TLC (90:10 hexanes:EtOAc) as colorless oil [9.6 mg, 74% yield, >20:1 N^{α}:N^{β}]. **^{1}H NMR** (400 MHz, $CDCl_3$) δ 7.49 – 7.40 (m, 3H), 7.39 – 7.34 (m, 2H), 5.98 (s, 1H), 2.28 (s, 3H), 1.17 (s, 9H). **^{13}C NMR** (151 MHz, $CDCl_3$) δ 154.29, 147.47, 142.00, 129.23, 128.99, 128.79, 103.92, 32.09, 30.87, 13.40. **IR** (ATR): 2968, 2929, 1593, 1537, 1500, 1362, 1017, 1007, 774, 729, 696 cm^{-1}. **HRMS** calculated for $C_{14}H_{18}N_2$ $[M+Na]^+$ 237.1368, found 237.1369.

5-(Tert-butyl)-3-iodo-1-phenyl-1*H*-pyrazole (3u)

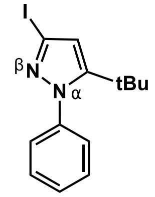

The title compound was synthesized according to general procedure A with **L1** and isolated by preparatory TLC (90:10 hexanes:EtOAc) as white solid [11.0 mg, 67% yield, >20:1 N^{α}:N^{β}]. **^{1}H NMR** (400 MHz, $CDCl_3$) δ 7.47 – 7.41 (m, 3H), 7.38 – 7.34 (m, 2H), 6.33 (s, 1H), 1.16 (s, 9H). **^{13}C NMR** (151 MHz, $CDCl_3$) δ 155.59, 141.49, 129.63, 128.87, 128.86, 112.99, 95.29, 32.13, 30.70. **IR** (ATR): 3135, 2966, 2927, 1500, 1362, 1337, 1257, 1005, 949, 785, 774, 706, 695 cm^{-1}. **HRMS** calculated for $C_{13}H_{15}IN_2$ $[M+Na]^+$ 349.0178, found 349.0175.

1,5-Diphenyl-1*H*-pyrazol-3-amine (3v)

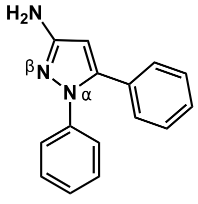

The title compound was synthesized according to general procedure A with **L1** and isolated by preparatory TLC (80:20 hexanes:EtOAc) as white solid [6.8 mg, 58% yield, 7:1 N^{α}:N^{β}]. **^{1}H NMR** (600 MHz, $CDCl_3$) δ 7.31 – 7.27 (m, 5H), 7.25 – 7.19 (m, 5H), 5.93 (s, 1H), 2.86 (s, 2H). **^{13}C NMR** (151 MHz, $CDCl_3$) δ 154.37, 144.85, 139.48, 130.32, 129.08, 128.79, 128.71, 128.63, 127.13, 124.98, 96.14. **IR** (ATR): 3320 (br), 2923, 1594, 1557, 1500, 1383, 760, 693 cm^{-1}. **HRMS** calculated for $C_{15}H_{13}N_3$ $[M+Na]^+$ 258.1007, found 258.1000.

3-Cyclopropyl-1-phenyl-5-(trifluoromethyl)-1*H*-pyrazole (3w)

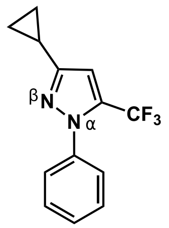

The title compound was synthesized according to general procedure A with **L2** and isolated by preparatory TLC (90:10 hexanes:EtOAc) as white solid [11.0 mg, 87% yield, >20:1 $N^{\alpha}:N^{\beta}$]. **^{1}H NMR** (600 MHz, $CDCl_3$) δ 7.52 – 7.37 (m, 5H), 6.47 (s, 1H), 2.00 (dddd, J = 8.5, 6.6, 4.2, 2.5 Hz, 1H), 0.99 (tdd, J = 6.1, 4.3, 1.5 Hz, 2H), 0.87 – 0.78 (m, 2H). **^{13}C NMR** (151 MHz, $CDCl_3$) δ 155.60, 139.33, 133.06 (q, J = 39.0 Hz), 129.16, 129.11, 125.76, 119.91 (q, J = 269.0 Hz), 105.65 (q, J = 2.5 Hz), 9.05, 8.35. ^{19}F NMR (565 MHz, $CDCl_3$) δ -57.55. **IR** (ATR): 2927, 1597, 1483, 1387, 1240, 1169, 1125, 1104, 973, 765, 686 cm^{-1}. The NMR data matches literature report.[69]

Methyl 5-ethyl-1-phenyl-1*H*-pyrazole-3-carboxylate (3x)

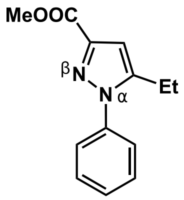

The title compound was synthesized according to general procedure A with **L2** and isolated by preparatory TLC (85:25 hexanes:EtOAc) as yellow oil [9.2 mg, 80% yield, 9:1 $N^{\alpha}:N^{\beta}$]. **^{1}H NMR** (400 MHz, $CDCl_3$) δ 7.49 – 7.37 (m, 5H), 6.84 (s, 1H), 3.78 (s, 3H), 2.74 (qd, J = 7.6, 0.5 Hz, 2H), 1.30 (t, J = 7.6 Hz, 3H). **^{13}C NMR** (151 MHz, $CDCl_3$) δ 159.86 (s), 155.05 (s), 140.37 (s), 133.47 (s), 128.70 (s), 128.59 (s), 126.09 (s), 110.88 (s), 52.07 (s), 21.41 (s), 13.81 (s). **IR** (ATR): 3065, 2969, 1731, 1503, 1444, 1283, 1227, 1105, 761, 692 cm^{-1}. **HRMS** calculated for $C_{13}H_{14}N_2O_2Na$ $[M+Na]^+$253.0953, found 253.0947.

Methyl 4-bromo-1-phenyl-1*H*-pyrazole-3-carboxylate (3y)

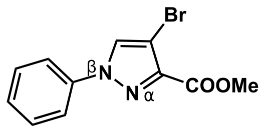

The title compound was synthesized according to general procedure A with **L1** and isolated by preparatory TLC (90:10 hexanes:EtOAc) as yellow solid [5.5 mg, 47% yield, <1:20 $N^{\alpha}:N^{\beta}$]. **^{1}H NMR** (400 MHz, $CDCl_3$) δ 8.00 (s, 1H), 7.70 – 7.68 (m, 2H), 7.50 – 7.46 (m, 2H), 7.40 – 7.37 (m, 1H), 3.98 (s, 3H). **^{13}C NMR** (151 MHz, $CDCl_3$) δ 161.59, 141.73, 139.18, 130.06, 129.80, 128.43, 120.11, 98.47, 52.43. **IR** (ATR): 3124, 2924, 1720, 1498, 1388, 1370, 1227, 1051, 957, 810, 746, 682, 649, 619 cm^{-1}. **HRMS** calculated for $C_{11}H_9BrN_2O_2$ $[M+Na]^+$ 302.9745, found 302.9738.

Ethyl 5-cyclopropyl-1-phenyl-1*H*-pyrazole-4-carboxylate (3z)

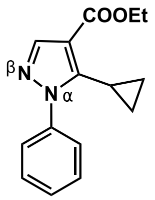

The title compound was synthesized according to general procedure A with **L1** and isolated by preparatory TLC (85:25 hexanes:EtOAc) as yellow oil [12.2 mg, 90% yield, >20:1 N^{α}:N^{β}]. **^{1}H NMR** (400 MHz, $CDCl_3$) δ 8.01 (s, 1H), 7.53 – 7.46 (m, 4H), 7.44 – 7.40 (m, 1H), 4.32 (q, J = 7.1 Hz, 2H), 1.97 (tt, J = 8.6, 5.5 Hz, 1H), 1.38 (t, J = 7.1 Hz, 3H), 0.95 – 0.88 (m, 2H), 0.68 – 0.62 (m, 2H). **^{13}C NMR** (151 MHz, $CDCl_3$) δ 163.35 (s), 147.48 (s), 142.34 (s), 139.62 (s), 129.07 (s), 128.49 (s), 125.58 (s, J = 3.8 Hz), 114.22 (s), 60.17 (s), 14.57 (s), 8.58 (s), 7.51 (s). **IR** (ATR): 3124, 2923, 1719, 1597, 1498, 1226, 1051, 958, 832, 746, 682, 650 cm^{-1}. **HRMS** calculated for $C_{15}H_{16}N_2O_2$ $[M+H]^+$ 257.1290, found 257.1281.

3,4-Dimethyl-1,5-diphenyl-1H-pyrazole (3aa)

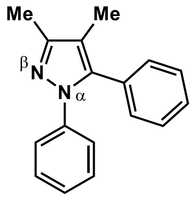

The title compound was synthesized according to general procedure A with **L2** and isolated by preparatory TLC (95:5 hexanes:EtOAc) as colorless oil [10.5 mg, 85% yield, >20:1 N^{α}:N^{β}]. **^{1}H NMR** (400 MHz, $CDCl_3$) δ 7.39-7.34 (m, 3H), 7.31 – 7.26 (m, 2H), 7.25 – 7.17 (m, 5H), 2.37 (s, 3H), 2.07 (s, 3H). **^{13}C NMR** (125 MHz, $CDCl_3$) δ 148.80, 140.28, 131.09, 129.83, 128.70, 128.45, 127.93, 126.43, 124.60, 114.73, 76.82, 12.08, 8.66. The NMR data matches literature report.[99]

1-Phenyl-1*H*-indazole (3ab)

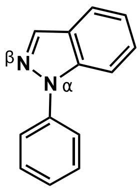

The title compound was synthesized according to general procedure A with dcypm ligand and isolated by preparatory TLC (90:10 hexanes:EtOAc) as yellow solid [6.4 mg, 46% yield, 15:1 N^{α}:N^{β}]. **^{1}H NMR** (600 MHz, $CDCl_3$) δ 8.21 (d, J = 1.0 Hz, 1H), 7.81 (dt, J = 8.1, 1.0 Hz, 1H), 7.77 (dt, J = 8.6, 0.9 Hz, 1H), 7.75 – 7.73 (m, 2H), 7.57 – 7.53 (m, 2H), 7.44 (ddd, J = 8.3, 6.9, 1.1 Hz, 1H), 7.39 – 7.35 (m, 1H), 7.24 (ddd, J = 8.0, 6.9, 0.8 Hz, 1H).**^{13}C NMR** (151 MHz, $CDCl_3$) δ 140.35, 138.90, 135.54, 129.60, 127.29, 126.79, 125.47, 122.90, 121.66, 121.48, 110.57. The NMR data matches literature report.[70]

1,5-Diphenyl-1*H*-1,2,4-triazole (3ac)

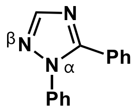

The title compound was synthesized according to general procedure A with **L2** and isolated by preparatory TLC (80:20 hexanes:EtOAc) as white solid [9 mg, 80% yield,

7:1 N$^{\alpha}$:N$^{\beta}$]. **^{1}H NMR** (600 MHz, $CDCl_3$) δ 8.10 (s, 1H), 7.51 – 7.47 (m, 2H), 7.44 – 7.38 (m, 4H), 7.38 – 7.32 (m, 4H). **^{13}C NMR** (151 MHz, $CDCl_3$) δ 154.23, 151.92, 138.52, 130.40, 129.76, 129.29, 129.23, 128.93, 128.10, 125.72. The NMR data matches literature report.[71]

4-Bromo-2-phenyl-2*H*-1,2,3-triazole (3ad)

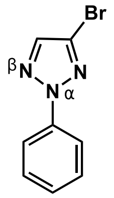

The title compound was synthesized according to general procedure A with **L1** and isolated by preparatory TLC (90:10 hexanes:EtOAc) as colorless solid [6.2 mg, 55% yield, 3:1 N$^{\alpha}$:N$^{\beta}$]. **^{1}H NMR** (600 MHz, $CDCl_3$) δ 8.05 – 8.01 (m, 2H), 7.76 (s, 1H), 7.49 (dd, J = 8.6, 7.4 Hz, 2H), 7.37 (t, J = 7.4 Hz, 1H). **^{13}C NMR** (151 MHz, $CDCl_3$) δ 139.58, 136.91, 129.52, 128.16, 124.12, 118.76. **IR** (ATR): 2924, 1597, 1496, 1445, 1371, 1130, 990, 959, 835, 753, 698, 688, 662 cm^{-1}. **HRMS** calculated for $C_8H_6BrN_3$ $[M+H]^+$222.9745, found 218.9856.

1,3-Diphenyl-1*H*-pyrazole (4a)

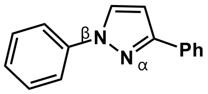

The title compound was synthesized according to general procedure B and isolated by preparatory TLC (90:10 hexanes:EtOAc) as yellow oil [10 mg, 90% yield, <1:20 N$^{\alpha}$:N$^{\beta}$]. **^{1}H NMR** (600 MHz, $CDCl_3$) δ 7.96 (d, *J* = 2.5 Hz, 1H), 7.95 – 7.91 (m, 2H), 7.80 – 7.76 (m, 2H), 7.50 – 7.42 (m, 4H), 7.37 – 7.33 (m, 1H), 7.31 – 7.28 (m, 1H), 6.78 (d, *J* = 2.5 Hz, 1H). ^{13}C NMR (151 MHz, $CDCl_3$) δ 153.28, 140.58, 133.47, 129.77, 129.00, 128.38, 128.34, 126.69, 126.19, 119.41, 105.38. The NMR data matches literature report.[63]

3-Methyl-1-phenyl-1*H*-pyrazole (4b)

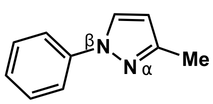

The title compound was synthesized according to general procedure B and isolated by preparatory TLC (90:10 hexanes:EtOAc) as colorless oil [11.6 mg, 84% yield, 1:9 N$^{\alpha}$:N$^{\beta}$]. **^{1}H NMR** (400 MHz, $CDCl_3$) δ 7.81 (s, 1H), 7.65 (d, J = 7.7 Hz, 2H), 7.43 (t, J = 7.8 Hz, 2H), 7.25 (t, J = 7.4 Hz, 1H), 6.25 (s, 1H), 2.39 (s, 3H). **^{13}C NMR** (151 MHz, $CDCl_3$) δ 150.65, 140.17, 129.53, 127.68, 126.23, 119.08, 107.71, 13.80. The NMR data matches literature report.[63]

3-Cyclopropyl-1-phenyl-1*H*-pyrazole (4c)

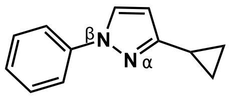

The title compound was synthesized according to general procedure B and isolated by preparatory TLC (90:10 hexanes:EtOAc) as yellow oil [14 mg, 75% yield, 1:12 N^{α}:N^{β}]. **^{1}H NMR** (500 MHz, $CDCl_3$) δ 7.78 (d, J = 2.5 Hz, 1H), 7.65 (d, J = 8.7 Hz, 2H), 7.42 (dd, J = 8.6, 7.4 Hz, 2H), 7.24 (tt, J = 7.4, 1.1 Hz, 1H), 6.11 (d, J = 2.5 Hz, 1H), 2.07 (tt, J = 8.4, 5.0 Hz, 1H), 1.05 – 0.95 (m, 2H), 0.85 – 0.78 (m, 2H). **^{13}C NMR** (125 MHz, $CDCl_3$) δ 157.18, 140.12, 129.49, 127.55, 126.14, 119.04, 104.14, 9.38, 8.35. The NMR data matches literature report.[72]

3-Isopropyl-1-phenyl-1*H*-pyrazole (4d)

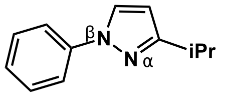

The title compound was synthesized according to general procedure B and isolated by preparatory TLC (90:10 hexanes:EtOAc) as yellow oil [16.5 mg, 89% yield, <1:20 N^{α}:N^{β}]. **^{1}H NMR** (400 MHz, $CDCl_3$) δ 7.81 (dd, J = 2.5, 0.5 Hz, 1H), 7.68 – 7.63 (m, 2H), 7.49 – 7.38 (m, 2H), 7.26 – 7.21 (m, 1H), 6.29 (dd, J = 2.4, 0.5 Hz, 1H), 3.10 (hept, J = 6.9 Hz, 1H), 1.33 (d, J = 7.0 Hz, 6H). **^{13}C NMR** (151 MHz, $CDCl_3$) δ 161.17, 140.37, 129.47, 127.37, 126.10, 119.19, 104.59, 28.12, 22.96. **IR** (ATR): 3049, 2962, 1601, 1529, 1502, 1302, 1042, 751, 689 cm^{-1}. The NMR data matches literature report.[73]

3-(Tert-butyl)-1-phenyl-1*H*-pyrazole (4e)

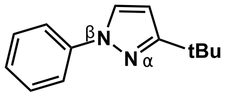

The title compound was synthesized according to general procedure B and isolated by preparatory TLC (90:10 hexanes:EtOAc) as colorless oil [19 mg, 95% yield, <1:20 N^{α}:N^{β}]. **^{1}H NMR** (600 MHz, $CDCl_3$) δ 7.80 (d, J = 2.4 Hz, 1H), 7.68 (d, J = 7.1 Hz, 2H), 7.42 (t, J = 7.9 Hz, 2H), 7.23 (t, J = 7.5 Hz, 1H), 6.32 (d, J = 2.4 Hz, 1H), 1.38 (s, 9H). **^{13}C NMR** (151 MHz, $CDCl_3$) δ 163.82, 140.63, 129.43, 126.95, 125.87, 119.06, 104.31, 32.47, 30.65. The NMR data matches literature report.[74]

1-Phenyl-3-(o-tolyl)-1*H*-pyrazole (4f)

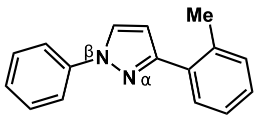

The title compound was synthesized according to general procedure B and isolated by preparatory TLC (90:10 hexanes:EtOAc) as white solid [16 mg, 70% yield, <1:20 N^{α}:N^{β}]. **^{1}H NMR** (600 MHz, $CDCl_3$) δ 7.99 (d, J = 2.4 Hz, 1H), 7.78 (dd, J = 8.6, 1.4 Hz, 2H), 7.67 (dd, J = 5.3, 3.6 Hz, 1H), 7.47 (dd, J = 8.6, 7.4 Hz, 2H), 7.31 – 7.27 (m, 4H), 6.65 (d, J = 2.4 Hz, 1H), 2.58 (s, 3H). **^{13}C NMR** (151 MHz, $CDCl_3$) δ 153.48,

140.29, 136.36, 132.92, 131.05, 129.53, 129.40, 128.04, 127.07, 126.34, 125.97, 119.00, 108.11, 21.57. The NMR data matches literature report.[75]

3-(2-Fluorophenyl)-1-phenyl-1*H*-pyrazole (4g)

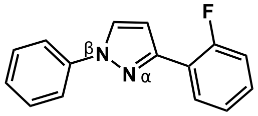

The title compound was synthesized according to general procedure B and isolated by preparatory TLC (90:10 hexanes:EtOAc) as colorless oil [16 mg, 69% yield, <1:20 N^{α}:N^{β}]. **^{1}H NMR** (600 MHz, $CDCl_3$) δ 8.18 (td, J = 7.8, 1.9 Hz, 1H), 7.99 (d, J = 2.5 Hz, 1H), 7.78 (dd, J = 8.6, 1.2 Hz, 2H), 7.48 (dd, J = 8.6, 7.4 Hz, 2H), 7.31 (t, J = 7.5 Hz, 2H), 7.23 (td, J = 7.5, 1.2 Hz, 1H), 7.16 (ddd, J = 11.3, 8.2, 1.2 Hz, 1H), 6.94 (dd, J = 4.0, 2.5 Hz, 1H). **^{13}C NMR** (151 MHz, $CDCl_3$) δ 160.44 (d, J = 249.5 Hz), 147.71, 140.20, 129.57, 129.46 (d, J = 8.4 Hz), 128.66 (d, J = 3.7 Hz), 127.87 (d, J = 2.5 Hz), 126.65, 124.42 (d, J = 3.3 Hz), 121.07 (d, J = 11.7 Hz), 119.28, 116.20 (d, J = 22.2 Hz), 108.64 (d, J = 10.5 Hz). **^{19}F NMR** (565 MHz, $CDCl_3$) δ -115.88. The NMR data matches literature report.[75]

3-(2-Bromophenyl)-1-phenyl-1*H*-pyrazole (4h)

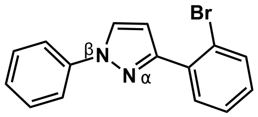

The title compound was synthesized according to general procedure B and isolated by preparatory TLC (90:10 hexanes:EtOAc) as colorless oil [21 mg, 14% yield, <1:20 N^{α}:N^{β}]. **^{1}H NMR** (600 MHz, $CDCl_3$) δ 7.99 (d, J = 2.5 Hz, 1H), 7.82 (dd, J = 7.7, 1.8 Hz, 1H), 7.77 (d, J = 8.5 Hz, 2H), 7.68 (dd, J = 8.1, 1.3 Hz, 1H), 7.47 (dd, J = 8.5, 7.4 Hz, 2H), 7.39 (td, J = 7.5, 1.2 Hz, 1H), 7.30 (t, J = 7.5 Hz, 1H), 7.22 (td, J = 7.7, 1.8 Hz, 1H), 6.97 (d, J = 2.5 Hz, 1H). **^{13}C NMR** (151 MHz, $CDCl_3$) δ 152.23, 140.17, 134.32, 133.69, 131.42, 129.58, 129.49, 127.59, 127.13, 126.66, 122.26, 119.30, 109.07. I**R** (ATR): 2922, 1599, 1521, 1445, 1024, 956, 749, 686 cm^{-1}. **HRMS** calculated for $C_{15}H_{11}BrN_2$ $[M+H]^+$ 299.0184, found 299.0171.

3-(2-Methoxyphenyl)-1-phenyl-1*H*-pyrazole (4i)

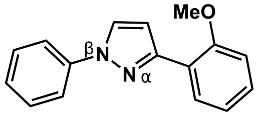

The title compound was synthesized according to general procedure B and isolated by preparatory TLC (90:10 hexanes:EtOAc) as colorless oil [20 mg, 81% yield, 1:12 N^{α}:N^{β}]. **^{1}H NMR** (600 MHz, $CDCl_3$) δ 8.11 (dd, J = 7.7, 1.8 Hz, 1H), 7.96 (d, J = 2.5 Hz, 1H), 7.79 (d, J = 8.7 Hz, 2H), 7.46 (t, J = 7.9 Hz, 2H), 7.33 (ddd, J = 8.7, 7.4, 1.8 Hz, 1H), 7.28 (t, J = 7.5 Hz, 1H), 7.06 (dd, J = 8.0, 6.9 Hz, 1H), 7.02 (d, J = 2.4

Hz, 1H), 7.01 (d, J = 8.3 Hz, 1H), 3.94 (s, 3H). **^{13}C NMR** (151 MHz, $CDCl_3$) δ 157.15, 150.12, 140.45, 129.50, 129.24, 129.03, 127.15, 126.29, 122.21, 121.03, 119.21, 111.46, 109.31, 55.65. The NMR data matches literature report.[72]

1-Phenyl-3-(p-tolyl)-1*H*-pyrazole (4j)

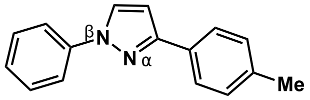

The title compound was synthesized according to general procedure B and isolated by preparatory TLC (90:10 hexanes:EtOAc) as white solid [22.1 mg, 94% yield, <1:20 N^{α}:N^{β}]. **^{1}H NMR** (500 MHz, $CDCl_3$) ^{1}H NMR (499 MHz, $CDCl_3$) δ 7.94 (d, J = 2.4 Hz, 1H), 7.86 – 7.80 (m, 2H), 7.79 – 7.75 (m, 2H), 7.50 – 7.43 (m, 2H), 7.34 – 7.27 (m, 1H), 7.27 – 7.22 (m, 2H), 6.75 (d, J = 2.4 Hz, 1H), 2.40 (s, 3H). **^{13}C NMR** (151 MHz, $CDCl_3$) δ 153.08, 140.31, 137.97, 130.35, 129.52, 129.47, 128.06, 126.36, 125.84, 119.13, 104.98, 21.46. **IR** (ATR): 3058, 2918, 1597, 1509, 1446, 1263, 1119, 1043, 953, 938, 823, 755, 690 cm^{-1}. **HRMS** calculated for $C_{16}H_{14}N_2$ $[M+H]^+$ 235.1235, found 235.1239. The NMR data matches literature report.[75]

3-(4-Chlorophenyl)-1-phenyl-1*H*-pyrazole (4k)

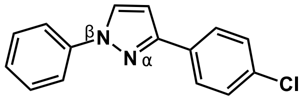

The title compound was synthesized according to general procedure B and isolated by preparatory TLC (90:10 hexanes:EtOAc) as white solid [8.0 mg, 31% yield, <1:20 N^{α}:N^{β}]. **^{1}H NMR** (400 MHz, $CDCl_3$) δ 7.96 (d, J = 2.6 Hz, 1H), 7.89 – 7.83 (m, 2H), 7.79 – 7.73 (m, 2H), 7.53 – 7.45 (m, 2H), 7.42 – 7.37 (m, 2H), 7.31 (ddt, J = 8.6, 7.0, 1.2 Hz, 1H), 6.75 (d, J = 2.5 Hz, 1H). **^{13}C NMR** (151 MHz, $CDCl_3$) δ 151.94, 140.21, 133.91, 131.74, 129.61, 128.97, 128.36, 127.23, 126.69, 119.26, 105.12. **IR** (ATR): 3058, 1598, 1505, 1441, 1087, 1044, 1012, 953, 939, 827, 754, 690 cm^{-1}. **HRMS** calculated for $C_{15}H_{11}ClN_2$ $[M+H]^+$255.0689, found 255.0678. The NMR data matches literature report.[75]

3-(4-Fluorophenyl)-1-phenyl-1*H*-pyrazole (4l)

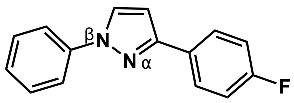

The title compound was synthesized according to general procedure B and isolated by preparatory TLC (90:10 hexanes:EtOAc) as white solid [11.9 mg, 50% yield, <1:20 N^{α}:N^{β}]. **^{1}H NMR** (400 MHz, $CDCl_3$) δ 7.95 (d, J = 2.5 Hz, 1H), 7.92 – 7.86 (m, 2H), 7.79 – 7.74 (m, 2H), 7.50 – 7.44 (m, 2H), 7.33 – 7.27 (m, 1H), 7.16 – 7.08 (m, 2H), 6.73 (d, J = 2.5 Hz, 1H). **^{13}C NMR** (151 MHz, $CDCl_3$) δ 162.92 (d, J = 247.3 Hz), 152.16,

140.23, 129.59, 129.43 (d, J = 3.02 Hz), 128.30, 127.68 (d, J = 8.3 Hz), 126.60, 119.22, 115.71 (d, J = 21.6 Hz), 104.96. **^{19}F NMR** (376 MHz, $CDCl_3$) δ -114.35. **IR** (ATR): 3059, 2923, 1597, 1506, 1446, 1221, 1155, 1043, 840, 751, 693, 605 cm^{-1}. **HRMS** calculated for $C_{15}H_{12}FN_2$ $[M+H]^+$ 239.0984, found 239.0993. The NMR data matches literature report.[72]

3-(4-Methoxyphenyl)-1-phenyl-1*H*-pyrazole (4m)

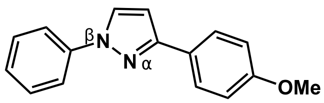

The title compound was synthesized according to general procedure B and isolated by preparatory TLC (90:10 hexanes:EtOAc) as white solid [20 mg, 80% yield, 1:17 $N^{\alpha}:N^{\beta}$]. **^{1}H NMR** (600 MHz, $CDCl_3$) δ 7.93 (d, J = 2.4 Hz, 1H), 7.87 – 7.85 (m, 2H), 7.77 (dq, J = 7.8, 1.1 Hz, 2H), 7.50 – 7.43 (m, 2H), 7.28 (t, J = 7.4 Hz, 1H), 6.98 (d, J = 8.9 Hz, 2H), 6.71 (d, J = 2.5 Hz, 1H), 3.86 (s, 3H). **^{13}C NMR** (151 MHz, $CDCl_3$) δ 159.74, 152.90, 140.36, 129.52, 128.04, 127.24, 126.29, 126.03, 119.07, 114.18, 104.71, 55.44. The NMR data matches literature report.[72]

3-(3-Methoxyphenyl)-1-phenyl-1*H*-pyrazole (4n)

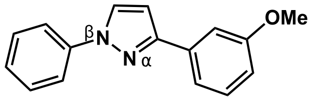

The title compound was synthesized according to general procedure B and isolated by preparatory TLC (90:10 hexanes:EtOAc) as white solid [22 mg, 88% yield, <1:20 $N^{\alpha}:N^{\beta}$]. **^{1}H NMR** (600 MHz, $CDCl_3$) δ 7.96 (d, J = 2.5 Hz, 1H), 7.81 – 7.75 (m, 2H), 7.53 – 7.45 (m, 4H), 7.35 (t, J = 7.9 Hz, 1H), 7.30 (tt, J = 7.4, 1.2 Hz, 1H), 6.91 (ddd, J = 8.3, 2.6, 1.0 Hz, 1H), 6.77 (d, J = 2.5 Hz, 1H), 3.90 (s, 3H). **^{13}C NMR** (151 MHz, $CDCl_3$) δ 160.06, 152.92, 140.33, 134.62, 129.80, 129.55, 128.12, 126.50, 118.97, 118.57, 114.12, 111.10, 105.33, 55.45. The NMR data matches literature report.[72]

3-(Furan-2-yl)-1-phenyl-1*H*-pyrazole (4o)

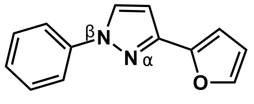

The title compound was synthesized according to general procedure B with $[Cu(CH_3CN)_4]PF_6$ (3.8 mg, 0.01 mmol, 10 mol%). It was isolated by preparatory TLC (80:20 hexanes:EtOAc) as yellow oil [17.1 mg, 81% yield, 1:13 $N^{\alpha}:N^{\beta}$]. **^{1}H NMR** (400 MHz, $CDCl_3$) δ 7.92 (d, J = 2.5 Hz, 1H), 7.77 – 7.71 (m, 2H), 7.50 (dd, J = 1.8, 0.8 Hz, 1H), 7.49 – 7.42 (m, 2H), 7.33 – 7.27 (m, 1H), 6.78 (dd, J = 3.4, 0.8 Hz, 1H), 6.70 (d, J = 2.5 Hz, 1H), 6.50 (ddd, J = 3.3, 1.8, 0.5 Hz, 1H). **^{13}C NMR** (151 MHz, $CDCl_3$) δ 148.62, 145.56,

142.31, 140.06, 129.56, 128.06, 126.73, 119.45, 111.54, 106.70, 105.10. The NMR data matches literature report.[72]

5-Methyl-1,3-diphenyl-1*H*-pyrazole (4p)

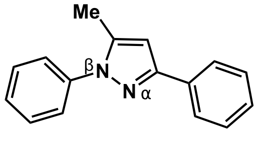

The title compound was synthesized according to general procedure B and isolated by preparatory TLC (90:10 hexanes:EtOAc) as yellow oil [18 mg, 76% yield, 1:8 N^{α}:N^{β}]. **^{1}H NMR** (400 MHz, $CDCl_3$) δ 7.89 – 7.85 (m, 2H), 7.58 – 7.46 (m, 4H), 7.44 – 7.36 (m, 3H), 7.35 – 7.28 (m, 1H), 6.53 (s, 1H), 2.39 (s, 3H). **^{13}C NMR** (151 MHz, $CDCl_3$) δ 151.61, 140.36, 139.98, 133.38, 129.23, 128.70, 127.93, 127.80, 125.88, 125.16, 104.51, 12.70. The NMR data matches literature report.[63]

3-(Tert-butyl)-5-methyl-1-phenyl-1*H*-pyrazole (4q)

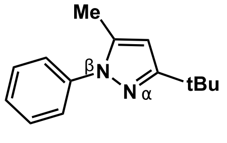

The title compound was synthesized according to general procedure B and isolated by preparatory TLC (90:10 hexanes:EtOAc) as yellow oil [21 mg, 98% yield, <1:20 N^{α}:N^{β}]. **^{1}H NMR** (600 MHz, $CDCl_3$) δ 7.46 (d, J = 4.3 Hz, 4H), 7.38 – 7.34 (m, 1H), 6.10 (d, J = 0.8 Hz, 1H), 2.31 (s, 3H), 1.37 (s, 9H). **^{13}C NMR** (151 MHz, $CDCl_3$) δ 162.17, 140.03, 139.17, 129.13, 127.37, 125.09, 103.82, 32.21, 30.65, 12.64.

1,3-Diphenyl-1*H*-pyrazol-5-amine (4r)

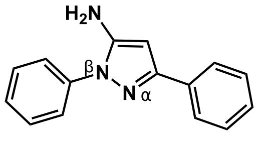

The title compound was synthesized according to general procedure B and isolated by preparatory TLC (80:20 hexanes:EtOAc) as white solid [13 mg, 55% yield, 1:12 N^{α}:N^{β}]. **^{1}H NMR** (600 MHz, $CDCl_3$) δ 7.82 (d, J = 6.9 Hz, 0H), 7.64 (d, J = 7.3 Hz, 0H), 7.50 (t, J = 7.9 Hz, 1H), 7.44 – 7.35 (m, 2H), 7.31 (t, J = 7.3 Hz, 0H), 5.97 (s, 1H), 3.86 (s, 1H). **^{13}C NMR** (151 MHz, $CDCl_3$) δ 151.65, 145.91, 138.78, 133.59, 129.65, 128.61, 127.95, 127.60, 125.76, 124.29, 88.27.

Ethyl 3-cyclopropyl-1-phenyl-1*H*-pyrazole-4-carboxylate (4s)

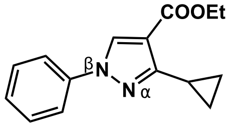

The title compound was synthesized according to general procedure B and isolated by preparatory TLC (75:25 hexanes:EtOAc) as white solid [21.5 mg, 84% yield, <1:20 N^{α}:N^{β}]. **^{1}H NMR** (400 MHz, $CDCl_3$) δ 8.30 (s, 1H), 7.69 – 7.60 (m, 2H), 7.50 – 7.39 (m, 2H), 7.29 (ddt, J = 8.0, 7.0, 1.2 Hz, 1H), 4.35 (q, J = 7.1 Hz, 2H),

2.59 (ddt, J = 8.4, 7.5, 5.1 Hz, 1H), 1.38 (t, J = 7.1 Hz, 3H), 1.08 – 0.97 (m, 4H). **^{13}C NMR** (151 MHz, $CDCl_3$) δ 163.85, 157.63, 139.54, 131.08, 129.58, 127.11, 119.39, 114.58, 60.22, 14.59, 8.98, 8.26. **IR** (ATR): 3133, 2974, 1469, 1541, 1263, 1249, 1189, 1069, 772, 750, 684 cm^{-1}. **HRMS** calculated for $C_{15}H_{16}N_2O_2$ $[M+H]^+$ 257.1290, found 257.1280.

2-Phenyl-2*H*-indazole (4t)

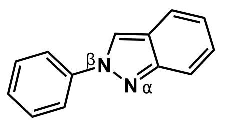

The title compound was synthesized according to general procedure B and isolated by preparatory TLC (90:10 hexanes:EtOAc) as yellow solid [7.8 mg, 40% yield, 1:10 N^{α}:N^{β}]. **^{1}H NMR** (600 MHz, $CDCl_3$) δ 8.42 (d, J = 1.0 Hz, 1H), 7.91 (dd, J = 8.6, 1.2 Hz, 2H), 7.80 (dd, J = 8.8, 1.0 Hz, 1H), 7.72 (d, J = 8.4 Hz, 1H), 7.53 (dd, J = 8.6, 7.3 Hz, 2H), 7.44 – 7.39 (m, 1H), 7.33 (ddd, J = 8.8, 6.6, 1.1 Hz, 1H), 7.12 (ddd, J = 8.4, 6.6, 0.9 Hz, 1H). **^{13}C NMR** (151 MHz, $CDCl_3$) δ 149.95, 140.70, 133.59, 129.73, 128.07, 126.99, 122.62, 121.18, 120.58, 120.53, 118.10. The NMR data matches literature report.[76]

1-(3,4-Dimethylphenyl)-5-phenyl-1*H*-pyrazole (5a)

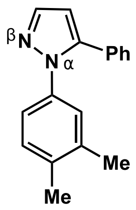

The title compound was synthesized according to general procedure A with **L2** and isolated by preparatory TLC (90:10 hexanes:EtOAc) as white solid [9.4 mg, 76% yield, 16:1 N^{α}:N^{β}]. **^{1}H NMR** (500 MHz, $CDCl_3$) δ 7.75 (d, J = 1.9 Hz, 1H), 7.33 – 7.29 (m, 3H), 7.26 – 7.23 (m, 2H), 7.20 (d, J = 2.3 Hz, 1H), 7.05 (d, J = 8.0 Hz, 1H), 6.92 (dd, J = 8.1, 2.3 Hz, 1H), 6.52 (d, J = 1.9 Hz, 1H), 2.26 (s, 3H), 2.23 (s, 3H). **^{13}C NMR** (151 MHz, $CDCl_3$) δ 143.44, 139.37, 137.79, 137.20, 136.65, 130.30, 130.03, 128.87, 128.59, 128.53, 126.43, 122.76, 107.61, 19.88, 19.59. **IR** (ATR): 3096, 3030, 2920, 2854, 1505, 1449, 1375, 971, 876, 786, 757, 699 cm^{-1}. **HRMS** calculated for $C_{17}H_{16}N_2$ $[M+H]^+$ 249.1392, found 249.1390.

1-(3,4-Dimethoxyphenyl)-5-phenyl-1*H*-pyrazole (5b)

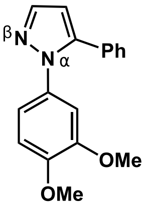

The title compound was synthesized according to general procedure A with **L3** and isolated by preparatory TLC (70:30 hexanes:EtOAc) as brown solid [11.4 mg, 81% yield, 12:1 N^{α}:N^{β}]. **^{1}H NMR** (499 MHz, $CDCl_3$) δ 7.70 (d, J = 1.8 Hz, 1H), 7.32 – 7.28 (m, 3H), 7.26 – 7.21 (m, 2H), 6.87 (d, J = 2.1 Hz, 1H), 6.79 (d, J = 2.1 Hz, 1H), 6.78 (s, 1H), 6.50 (d, J = 1.8 Hz, 1H), 3.88 (s, 3H), 3.73 (s, 3H). **^{13}C NMR** (151 MHz, $CDCl_3$) δ 149.03, 148.40, 143.10, 140.10, 133.52, 130.83, 128.88, 128.55, 128.25, 117.59, 110.81, 109.22,

107.54, 56.14, 56.01. **IR** (ATR): 3184, 2924, 1599, 1518, 1247, 1224, 1176, 1125, 1022, 858, 791, 761, 702 cm^{-1}. **HRMS** calculated for $C_{17}H_{16}N_2O_2$ $[M+H]^+$ 281.1290, found 281.1277.

1-(Naphthalen-2-yl)-5-phenyl-1*H*-pyrazole (5c)

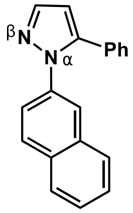

The title compound was synthesized according to general procedure A with **L3** and isolated by preparatory TLC (70:30 hexanes:EtOAc) as yellow solid [7.5mg, 50% yield, 11:1 N^{α}:N^{β}]. **^{1}H NMR** (500 MHz, $CDCl_3$) δ 7.85 – 7.83 (m, 2H), 7.79 – 7.74 (m, 3H), 7.51 – 7.47 (m, 2H), 7.38 (dd, J = 8.7, 2.1 Hz, 1H), 7.30 – 7.27 (m, 5H), 6.56 (d, J = 1.7 Hz, 1H). **^{13}C NMR** (151 MHz, $CDCl_3$) δ 143.31, 140.66, 137.77, 133.38, 132.34, 130.75, 128.94, 128.84, 128.65, 128.40, 128.29, 127.87, 126.81, 126.54, 123.70, 123.44, 108.15. **IR** (ATR): 3057, 2922, 1598, 1509, 1128, 926, 868, 830, 763, 753, 695 cm^{-1}. **HRMS** calculated for $C_{19}H_{14}N_2$ $[M+Na]^+$293.1055, found 293.1050.

5-Phenyl-1-(p-tolyl)-1*H*-pyrazole (5d)

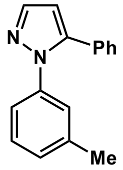

The title compounds was synthesized according to general procedure A with **L2** and isolated by preparatory TLC (90:10 hexanes:EtOAc) as white solid [11 mg, 98% yield, >20:1 N^{α}:N^{β}, 1:1 C^1:C^2]. **^{1}H NMR** (600 MHz, $CDCl_3$) δ 7.71 (d, *J* = 1.9 Hz, 1H), 7.32 – 7.28 (m, 3H), 7.25 – 7.24 (m, 3H), 7.17 (t, *J* = 7.8 Hz, 1H), 7.11 (d, *J* = 7.7 Hz, 1H), 6.98 (dt, *J* = 8.1, 1.6 Hz, 1H), 6.50 (d, *J* = 1.8 Hz, 1H), 2.33 (s, 3H). **^{13}C NMR** (151 MHz, $CDCl_3$) δ 142.98, 140.21, 140.10, 139.10, 130.72, 128.77, 128.55, 128.44, 128.22, 128.17, 125.85, 122.35, 107.74, 21.34. The NMR data matches literature report.[78]

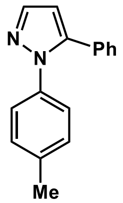

^{1}H NMR (600 MHz, $CDCl_3$) δ 7.70 (d, J = 1.8 Hz, 1H), 7.31 – 7.30 (m, 3H), 7.25 – 7.23 (m, 2H), 7.18 (d, J = 8.3 Hz, 2H), 7.12 (d, J = 8.1 Hz, 2H), 6.50 (d, J = 1.8 Hz, 1H), 2.36 (s, 3H). The NMR data matches literature report.[78]

1-(4-Chlorophenyl)-5-phenyl-1*H*-pyrazole (5e)

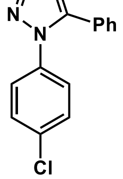

The title compound was synthesized according to general procedure C with **L2** and isolated by preparatory TLC (90:10 hexanes:EtOAc) as yellow oil [5.1 mg, 50% yield, 12:1 N^{α}:N^{β}, 2:1 C^1:C^2]. **^{1}H NMR** (600 MHz, $CDCl_3$) δ 7.72 (d, *J* = 1.7 Hz, 1H), 7.39 –

7.27 (m, 5H), 7.27 – 7.20 (m, 4H), 6.51 (d, *J* = 1.6 Hz, 1H). **IR** (ATR): 2923, 1594, 1496, 1382, 1093, 959, 923, 830, 759, 696 cm^{-1}. The NMR data matches literature report.[78]

5-Phenyl-1-(m-tolyl)-1*H*-pyrazole (5f)

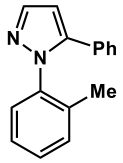

The title compound was synthesized according to general procedure A with **L3** and isolated by preparatory TLC (90:10 hexanes:EtOAc) as white oil [9.8 mg, 84% yield, >20:1 N^{α}:N^{β}, 1:5 C^1:C^2]. **^{1}H NMR** (499 MHz, $CDCl_3$) δ 7.73 (d, J = 1.9 Hz, 1H), 7.32–7.26 (m, 1H), 7.26 – 7.21 (m, 6H), 7.18 – 7.15 (m, 2H), 6.56 (d, J = 1.9 Hz, 1H), 1.96 (s, 3H). **^{13}C NMR** (151 MHz, $CDCl_3$) δ 144.18, 140.20, 139.68, 135.75, 131.12, 130.42, 129.05, 128.54, 128.22, 128.13, 127.99, 126.71, 106.05, 17.67. **IR** (ATR): 3057, 2922, 1498, 1384, 1067, 960, 925, 757, 693 cm^{-1}. **HRMS** calculated for $C_{16}H_{14}N_2$ $[M+H]^+$ 235.1235, found 235.1244. The NMR data matches literature report.[77]

5-Phenyl-1-(2-(trimethylsilyl)phenyl)-1*H*-pyrazole (5g)

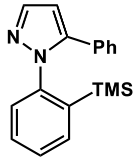

The title compound was synthesized according to general procedure A with **L1** and isolated by preparatory TLC (90:10 hexanes:EtOAc) as colorless oil [7.2 mg, 84% yield, >20:1 N^{α}:N^{β}, >20:1 C^1:C^2]. **^{1}H NMR** (600 MHz, CD_3CN) δ 7.75 (d, J = 7.5 Hz, 1H), 7.65 (t, J = 1.6 Hz, 1H), 7.43 (t, J = 7.4 Hz, 1H), 7.30 (tt, J = 7.7, 1.5 Hz, 1H), 7.28 – 7.23 (m, 3H), 7.20 – 7.16 (m, 2H), 6.89 (d, J = 7.8 Hz, 1H), 6.63 (t, J = 1.6 Hz, 1H), 0.04 (s, 9H).**^{13}C NMR** (151 MHz, CD_3CN) δ 146.41, 144.42, 140.10, 139.12, 136.87, 131.23, 130.56, 129.40, 129.25, 129.01, 128.97, 128.66, 107.76, -0.62. **IR** (ATR): 3059, 2951, 1480, 1432, 1245, 1121, 925, 837, 757, 724, 694 cm^{-1}. **HRMS** calculated for $C_{18}H_{20}N_2Si$ $[M+H]^+$ 293.1474, found 293.1476.

1-([1,1'-Biphenyl]-2-yl)-5-phenyl-1*H*-pyrazole (5h)

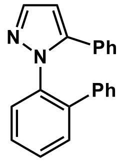

The title compound was synthesized according to general procedure A with **L1** and isolated by preparatory TLC (90:10 hexanes:EtOAc) as white solid [6.8 mg, 46% yield, 6:1 N^{α}:N^{β}, <1:20 C^1:C^2]. **^{1}H NMR** (600 MHz, CD_3CN) δ 7.73 (d, J = 1.9 Hz, 1H), 7.60 (ddd, J = 7.8, 1.8, 1.0 Hz, 1H), 7.47 (t, J = 7.9 Hz, 1H), 7.45 – 7.43 (m, 3H), 7.40 (td, J = 6.6, 1.8 Hz, 2H), 7.38 – 7.29 (m, 7H), 6.60 (d, J = 1.8 Hz, 1H). **^{13}C NMR** (151 MHz, $CDCl_3$) δ 143.23, 142.09, 140.58, 140.53, 140.17, 130.89, 129.39, 129.03, 128.89, 128.69, 128.41, 127.78, 127.22,

126.12, 124.04, 123.88, 108.08. **IR** (ATR): 3057, 2922, 1599, 1483, 1425, 1384, 965, 924, 888, 792, 756, 696 cm^{-1}. **HRMS** calculated for $C_{21}H_{16}N_2$ $[M+H]^+$ 297.1392, found 297.1378.

1-(3-Nitrophenyl)-5-phenyl-1*H*-pyrazole (5i)

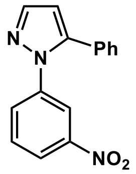

The title compound was synthesized according to general procedure C with **L1** and isolated by preparatory TLC (70:30 hexanes:EtOAc) as yellow solid [6.2 mg, 47% yield, 2:1 N^{α}:N^{β}, >20:1 C^1:C^2]. **^{1}H NMR** (600 MHz, $CDCl_3$) δ 8.24 (t, J = 2.2 Hz, 1H), 8.14 (ddd, J = 8.2, 2.2, 1.0 Hz, 1H), 7.78 (d, J = 1.8 Hz, 1H), 7.59 (ddd, J = 8.1, 2.2, 1.0 Hz, 1H), 7.48 (t, J = 8.1 Hz, 1H), 7.41 – 7.33 (m, 3H), 7.26 – 7.21 (m, 2H), 6.55 (d, J = 1.8 Hz, 1H). **^{13}C NMR** (151 MHz, $CDCl_3$) δ 148.55, 143.59, 141.51, 141.13, 130.43, 130.05, 129.77, 129.08, 129.03, 128.98, 121.90, 119.93, 109.15. **IR** (ATR): 3098, 2922, 1528, 1490, 1440, 1378, 1348, 923, 893, 801, 761, 737, 697, 674, 644 $cm^{-1.}$ **HRMS** calculated for $C_{15}H_{11}N_3O_2$ $[M+H]^+$ 266.0930, found 266.0932.

1-(Naphthalen-1-yl)-5-phenyl-1*H*-pyrazole (5j')

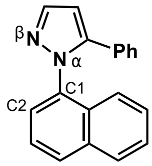

The title compounds was synthesized according to general procedure A with **L4** and isolated by preparatory TLC (90:10 hexanes:EtOAc) as white solid [6.4 mg, 47% yield, 14:1 N^{α}:N^{β}, 6:1 C^1:C^2]. **^{1}H NMR** (600 MHz, $CDCl_3$) δ 7.91 (d, J = 8.2 Hz, 2H), 7.85 (d, J = 1.5 Hz, 1H), 7.58 (d, J = 8.3 Hz, 1H), 7.51 (t, J = 7.4 Hz, 1H), 7.49 – 7.45 (m, 1H), 7.42 (t, J = 7.7 Hz, 1H), 7.32 (d, J = 7.2 Hz, 1H), 7.19 – 7.09 (m, 5H), 6.66 (d, J = 1.6 Hz, 1H). **^{13}C NMR** (151 MHz, $CDCl_3$) δ 145.29, 140.60, 136.86, 134.42, 130.74, 130.26, 129.38, 128.45, 128.19, 128.17, 128.11, 127.43, 126.73, 125.77, 125.15, 123.52, 106.46. **IR** (ATR): 3054, 2924, 1510, 1408, 1129, 924, 801, 773, 757, 730, 695 cm^{-1}. **HRMS** calculated for $C_{19}H_{14}N_2$ $[M+H]^+$ 271.1235, found 271.1234.

5. Synthesis of CDPPB (7) and CDPPB-*N*-isomer (8)

The preparation of 3-cyano-*N*-(5-phenyl-1*H*-pyrazol-3-yl)benzamide (**6**) was performed using literature reported procedure.[79]

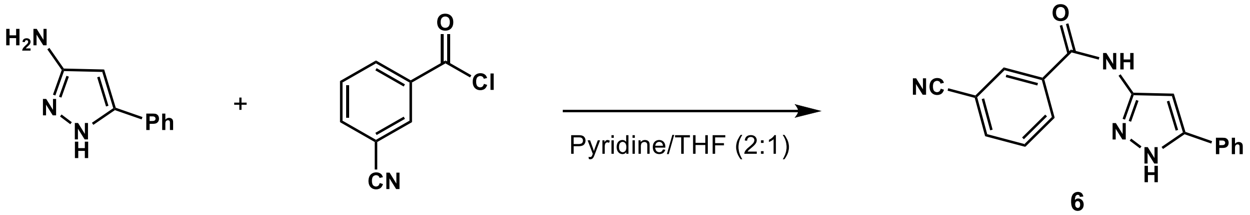

6

5-Phenyl-1*H*-pyrazol-3-amine (200 mg, 1.26 mmol, 1.00 equiv.) was suspended in dry pyridine/dry THF (12/6 ml) mixture at 0 °C. A solution of 3-cyanobenzoyl chloride (196 µL, 250 mg, 1.51 mmol, 1.20 equiv.) in dry THF (2 mL) was added to this suspension dropwise via a syringe pump (1 mL/h), the suspension was stirred for 1 h at room temperature. The reaction mixture was quenched with H_2O. The precipitate was filtered off, washed with H_2O (3×) and dried in vacuo. 146 mg pink solid was isolated in 40% yield.

3-Cyano-*N*-(5-phenyl-1*H*-pyrazol-3-yl)benzamide (6)

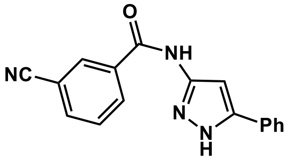

^{1}H NMR (600 MHz, DMSO) δ 13.02 (s, 1H), 11.13 (s, 1H), 8.46 (s, 1H), 8.33 – 8.29 (m, 1H), 8.05 (d, *J* = 7.7 Hz, 1H), 7.75 (dd, *J* = 20.7, 7.8 Hz, 3H), 7.46 (t, *J* = 7.6 Hz, 2H), 7.36 (t, *J* = 7.4 Hz, 1H), 7.07 (s, 1H). **^{13}C NMR** (151 MHz, DMSO) δ 162.32, 149.21, 147.61, 141.53, 134.60, 132.13, 131.08, 129.37, 128.88, 128.62, 127.77, 124.59, 117.92, 111.12, 94.23. **IR** (ATR): 3329, 2235, 1662, 1609, 1540, 1507, 1466, 1418, 1296, 1192, 1072, 1027, 953, 756, 739, 693, 676, 620, 587, 580 cm^{-1}. **HRMS** calculated for $C_{17}H_{12}N_4O$ $[M+Na]^+$ 311.0909, found 311.0895.

3-Cyano-*N*-(1,3-diphenyl-1*H*-pyrazol-5-yl)benzamide (7, CDPPB)

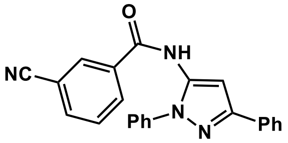

The title compound was synthesized according to general procedure B with **L5** and isolated by preparatory TLC (60:40 hexanes:EtOAc) as white solid [10.2 mg, 56% yield, 1:6 $N^{\alpha}:N^{\beta}$]. **^{1}H NMR** (600 MHz, DMSO) δ 10.75 (s, 1H), 8.31 (s, 1H), 8.18 (d, *J* = 8.0 Hz, 1H), 8.10 (d, *J* = 7.8 Hz, 1H), 7.91 (d, *J* = 7.6 Hz,

2H), 7.76 (t, *J* = 7.8 Hz, 1H), 7.65 (d, *J* = 7.8 Hz, 2H), 7.54 – 7.49 (m, 2H), 7.46 (t, *J* = 7.5 Hz, 2H), 7.38 (dt, *J* = 14.3, 7.4 Hz, 2H), 7.02 (d, *J* = 1.5 Hz, 1H). **^{13}C NMR** (151 MHz, DMSO) δ 164.03, 149.97, 138.45, 136.47, 135.35, 133.90, 132.43, 132.17, 131.10, 129.80, 128.97, 128.50, 127.86, 127.29, 124.91, 123.09, 117.85, 111.49, 101.08. **IR** (ATR): 3252, 2922, 2231, 1648, 1595, 1564, 1519, 1500, 1475, 1456, 1418, 1363, 1303, 1169, 1074, 949, 914, 817, 802, 760, 747, 687, 679, 640 cm^{-1}. **HRMS** calculated for $C_{23}H_{16}N_4O$ $[M+H]^+$ 365.1402, found 365.1411. The NMR data matches literature report.[49]

3-Cyano-*N*-(1,5-diphenyl-1*H*-pyrazol-3-yl)benzamide (8, CDPPB-*N*-Isomer)

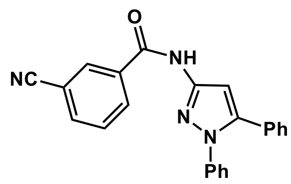

The title compound was synthesized according to general procedure A with **L2** and isolated by preparatory TLC (60:40 hexanes:EtOAc) as white solid [5.6 mg, 31% yield, 4:1 N^{α}:N^{β}]. **^{1}H NMR** (600 MHz, DMSO) δ 11.42 (s, 1H), 8.49 (s, 1H), 8.33 (d, *J* = 8.0 Hz, 1H), 8.07 (d, *J* = 7.7 Hz, 1H), 7.75 (t, *J* = 7.8 Hz, 1H), 7.42 (t, *J* = 7.6 Hz, 2H), 7.38 (t, *J* = 3.1 Hz, 4H), 7.29 (d, *J* = 6.8 Hz, 4H), 7.07 (s, 1H). **^{13}C NMR** (151 MHz, DMSO) δ 162.77, 147.70, 142.50, 139.20, 134.91, 134.61, 132.31, 131.32, 129.56, 129.37, 128.84, 128.42, 128.33, 128.19, 127.34, 124.85, 118.02, 111.31, 100.02. **IR** (ATR): 3243, 3067, 2922, 2852, 2232, 1667, 1567, 1495, 1471, 1368, 1280, 1191, 1073, 1016, 971, 913, 806, 762, 738 cm^{-1}. **HRMS** calculated for $C_{23}H_{16}N_4O$ $[M+Na]^+$ 387.1222, found 387.1217.

6. Synthesis and Reactivity of Cu-pyrazolate

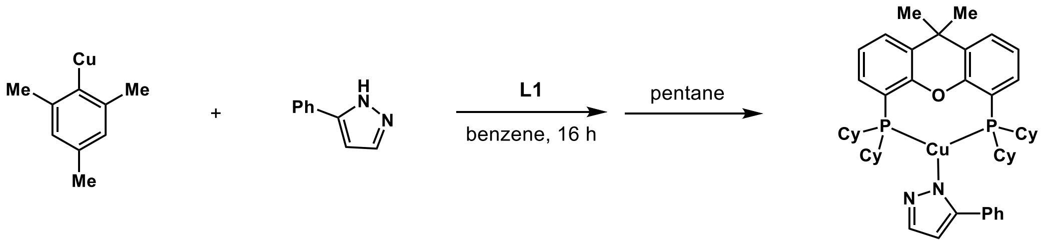

CyXant-Cu-N$^{\alpha}$

Preparation of CyXant-Cu-N$^{\alpha}$: In a nitrogen-filled glovebox, an oven-dried 4 mL amber-glass vial was charged with pyrazole (14.4 mg, 0.10 mmol), a stir bar, and a solution of mesitylcopper (18.3 mg, 0.10 mmol) in benzene (400 uL). The mixture was stirred for 10 min, and then a solution of **L1** (60.3 mg, 0.10 mmol) in benzene (600 uL) was added. The resulting reaction mixture was stirred for 6 h, and then pentane (2.0 mL) was added dropwise. This mixture was stirred for 1 h, during which time a white precipitate formed. The white precipitate was filtered, rinsed with pentane (5 mL), and dried to give 45 mg (56% yield) of the desired product as a white powder. X-ray quality crystals were obtained by slow evaporation of solvent from a saturated solution in a mixture of benzene/ether/pentane. [22]

^{1}H NMR (600 MHz, THF) δ 7.91 (d, *J* = 7.6 Hz, 2H), 7.58 (d, *J* = 7.6 Hz, 2H), 7.51 (d, *J* = 1.6 Hz, 1H), 7.48 – 7.44 (m, 2H), 7.21 (dt, *J* = 20.9, 7.6 Hz, 4H), 6.99 (t, *J* = 7.3 Hz, 1H), 6.42 (d, *J* = 1.7 Hz, 1H), 2.41 (t, *J* = 11.7 Hz, 4H), 2.09 (d, *J* = 11.3 Hz, 4H), 1.69 – 1.52 (m, 17H), 1.30 (dddd, *J* = 53.4, 26.6, 14.4, 7.9 Hz, 18H), 1.09 (ddt, *J* = 18.8, 12.9, 6.4 Hz, 4H). **^{13}C NMR** (151 MHz, THF) δ 138.43, 134.90, 130.92, 127.49, 126.04, 124.60, 123.88, 123.30, 98.70, 33.31, 33.25, 33.19, 29.08, 28.34, 26.97, 26.93, 26.87, 26.83, 26.80, 25.97. **^{31}P NMR** (243 MHz, THF) δ -15.54.

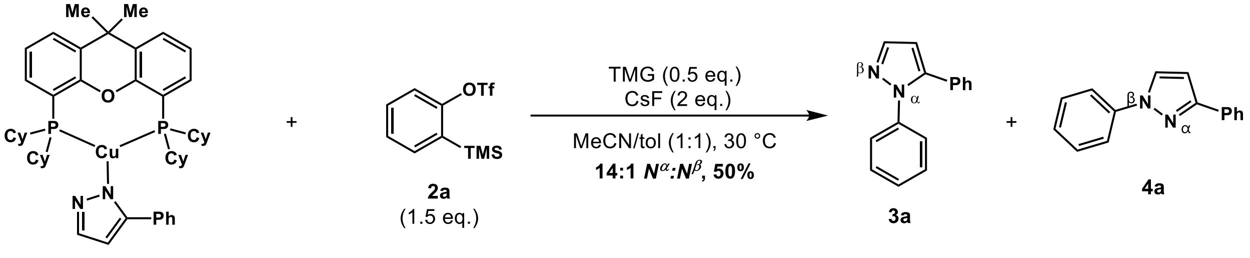

CyXant-Cu-N$^{\alpha}$ **2a** (1.5 eq.) **3a** **4a**

Stoichiometric reaction of CyXant-Cu-N$^{\alpha}$: In a N_2 filled glovebox, to a 1-dram vial equipped with a magnetic stir bar was added **CyXant-Cu-N$^{\alpha}$** (40.5 mg, 0.05 mmol, 1.0 equiv.) and acetonitrile (1.0 mL, 0.05 M). After stirring for 30 min, 1,1,3,3-tetramethylguanidine (TMG, 2.9 mg, 0.025 mmol, 0.5 equiv.) was added. The resulting mixture was stirred for another 30 min followed by the addition of 2-(trimethylsilyl)phenyl trifluoromethanesulfonate (**2**) (18.2 μL, 22.4 mg, 0.075 mmol, 1.5 equiv.) and CsF (15.2 mg, 0.1 mmol, 2.0 equiv.). The vial was sealed with a Teflon-lined screw cap and stirred at 30 °C for 12 hours. The mixture was concentrated in vacuo. Regioselectivity (rr) was determined by ^{1}H NMR analysis of the crude mixture (14:1 N$^{\alpha}$:N$^{\beta}$). Isolated yield reported.

7. DFT Calculations

7.1. General Information

Density functional theory (DFT) was employed to study the regioselectivity-determining aminocupration step in the arylation of substituted pyrazoles via copper-catalysis. Pyrazole **3t** and benzyne precursor **2a** were chosen as the model substrate system for our theoretical study using Cu-Cy-XantPhos (**L1**) as the catalytic species (***N$^{\alpha}$*-arylation**), while pyrazole **4q** was employed for analysis using Cu-1,10-phenanthroline (**L5**) as the catalytic species (***N$^{\beta}$*-arylation**). All ground state and transition state optimizations as well as the calculation of vibrational frequencies were performed at the M062X/6-31G(d) level of theory in gas phase, as implemented in Gaussian 16. Single point energies were computed at the M062X/6-311+G(d,p) level of theory utilizing a polarized continuum solvation model (PCM) for acetonitrile. Thermal corrections to free energies at 30°C were implemented using Grimme's quasi-rigid rotor harmonic oscillator (QRRHO) approximation.[80] Transition structures (TSs) were verified as exhibiting one sole imaginary frequency, and intrinsic reaction coordinate calculations were performed to confirm the TSs connect the minima along the potential energy surface. Conformational analysis was carried out for all TSs. Molecular graphics were generated using CYLView,[81] Multiwfn,[82] VMD,[83] and ChimeraX.[84]

7.2. Analysis of the Cy-XantPhos (L1)-Controlled N^{α}-Arylation

Two possible mechanisms of aminocupration were explored for the addition of pyrazole **3t** to benzyne: a 1,2-migratory insertion pathway and a 1',6'-migratory insertion pathway.[10] The latter can be described as a 5-centered aminocupration transition state with copper remaining bound to one nitrogen of pyrazole during arylation of the other nitrogen heteroatom by **2a (Figure 1A)**. Transition states were located for the addition of **2a** to **3t**'s more sterically hindered N^{α} via metallo-tautomer **Cu-Nβ-3t** (**TS-Nα-3t**) as well as the addition of **2a** to its less sterically hindered N^{β} via metallo-tautomer **Cu-Nα-3t** (**TS-Nβ-3t**). **TS-Nβ-3t**, leading to the minor N^{β}-regioisomer, was found to be 1.2 kcal/mol higher in energy than **TS-Nα-3t**, leading to the major N^{α}-regioisomer. This is consistent with the experimentally observed >20:1 N^{α}:N^{β} regioselectivity under **L1** reaction conditions.

Alternatively, the 1,2-migratory insertion pathway—arylation occurring via a 4-centered transition state with the nitrogen involved in the Cu-pyrazolate complex undergoing C-N bond formation with **2a**—was also explored for the addition of both the more sterically hindered N^{α} and the less hindered N^{β} (**TS-MI$_{\mathbf{N\alpha\text{-}3t}}$** and **TS-MI$_{\mathbf{N\beta\text{-}3t}}$**, respectively) **(Figure 1B)**. However, both transition states were higher in energy than their respective 1',6'-migratory insertion counterparts. Overall, **TS-Nα-3t** represents the lowest energy TS for arylation of **3t** with the 1',6'-migratory insertion pathway being favored over the 1,2-migratory pathway by 3.4-9.5 kcal/mol.

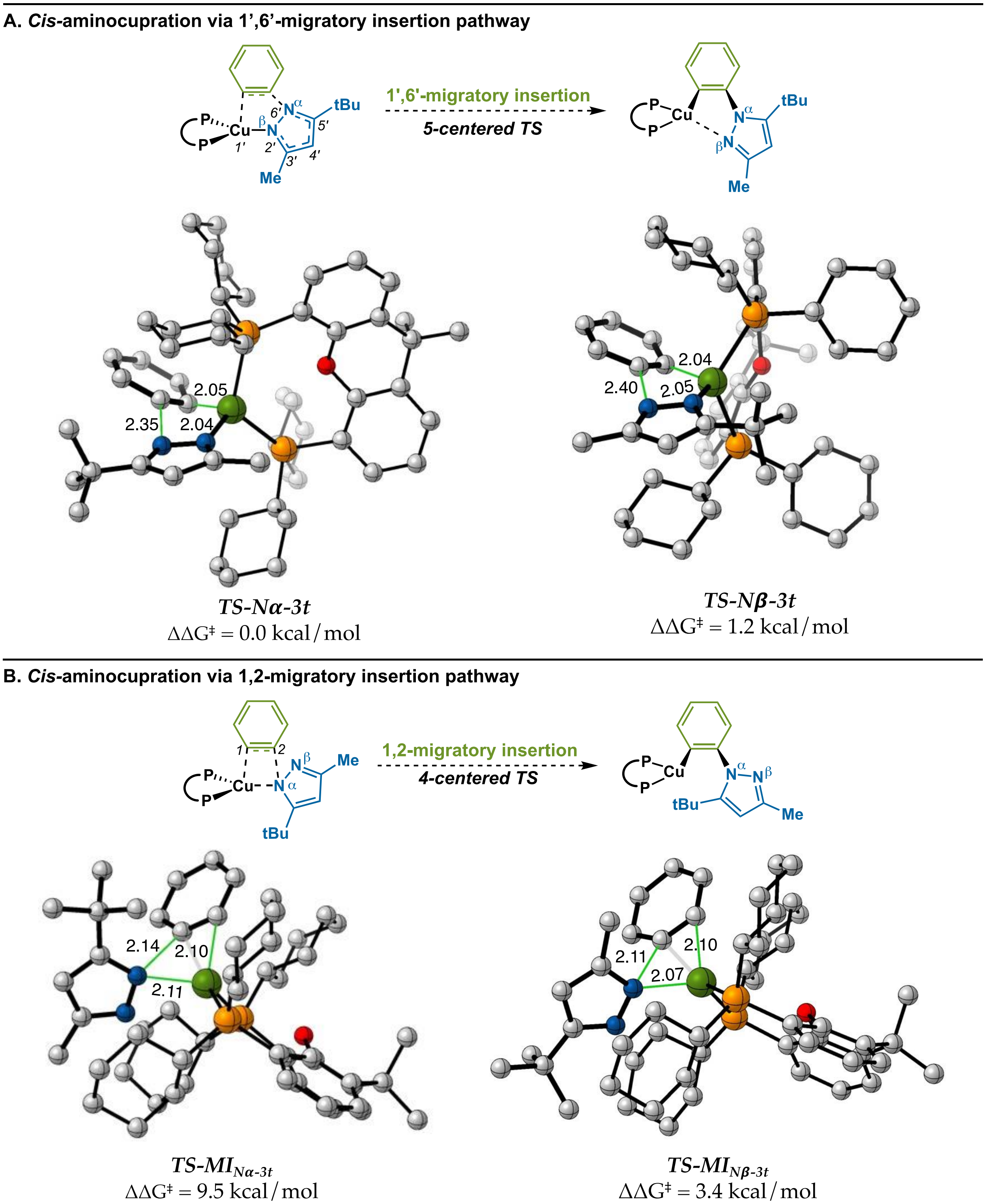

Figure 1. TSs for the favored, 5-centered 1',6'-migratory insertion pathway **(A)** and the disfavored, 4-centered 1,2-migratory insertion pathway **(B)** for the arylation of **3t**. Free energies provided are relative to **TS-Nα-3t**.

The relative potential energy surface for this transformation was validated across several levels of theory in which the functional, basis set and solvation models were varied. The results of this are summarized below in **Figure 2**, depicting the relative energy difference ($\Delta\Delta G^{\ddagger}$) between **TS-Nα-3t** and **TS-Nβ-3t** as well as the activation barrier for each TS relative to their aryne-coordinated Cu-pyrazolate complex obtained by reverse reaction coordinate analysis. This data has also been plotted along the potential energy surface for visual comparison. While the exact energies vary, all explored levels of theory exhibit a similar potential energy profile and unanimously favor Nα-arylation—an average $\Delta\Delta G^{\ddagger}$ of 2.2 kcal/mol is observed between **TS-Nα-3t** and **TS-Nβ-3t.** A conservative $\Delta\Delta G^{\ddagger}$ of 1.2 kcal/mol at M06-2X/6-311+G(d,p) PCM (MeCN) // M06-2X/6-31G(d) was chosen for our study.

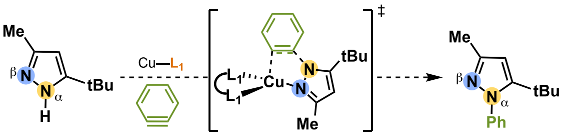

TS-Nα-3t

Level of Theory	ΔΔG‡ (α **vs. β**) (kcal/mol)	Barrier of **TS-Nα-3t** from rev qrc (kcal/mol)	Barrier of **TS-Nβ-3t** from rev qrc (kcal/mol)
M062X/6-31G* PCM (MeCN) // M062X/6-311+G** PCM (MeCN)	0.5	7.5	1.7
M062X/6-31G* // M062X/6-311+G** PCM (MeCN)	1.2	7.8	3.0
M062X/6-31G* SDD (Cu) // M062X/6-311+G** SDD (Cu) PCM (MeCN)	1.3	5.2	1.7
wB97xD/def2SVP // wB97xD/def2TZVP SMD (MeCN)	1.6	12.4	5.1
B97D/def2SVP // wB97xD/def2TZVP SMD (MeCN)	3.2	9.9	5.8
B3LYP-D3/6-31G* PCM (MeCN) // B3LYP-D3/6-311+G** PCM (MeCN)	3.5	9.1	3.2
B3LYP-D3/6-31G* // B3LYP-D3/6-311+G** PCM (MeCN)	4.3	9.1	5.0
**Average**	**2.2**	**8.7**	**3.9**

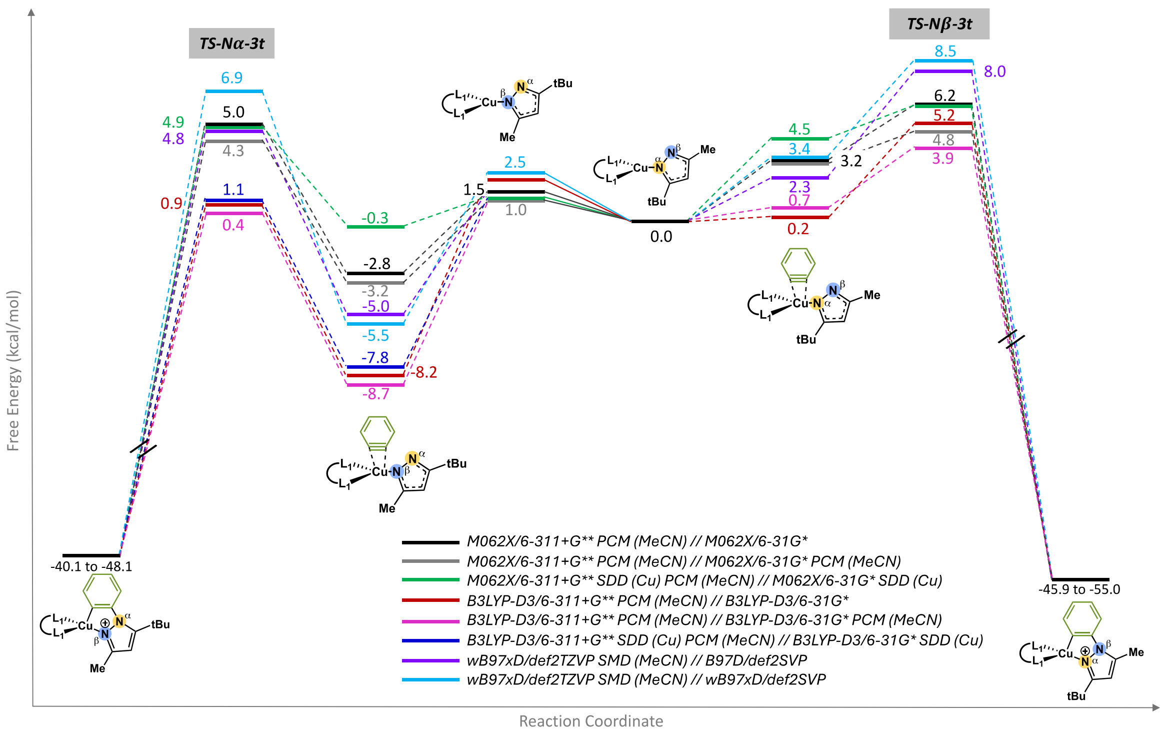

Figure 2. Comparison of the relative energy difference (ΔΔG‡) of TS Nα-3t **and** TS-Nβ-3t as well as the activation energy for each across various levels of theory. The chosen functional/basis set is shown in blue. A potential energy surface is also provided showing good agreement in the relative barriers of the key TSs and intermediates.

For geometrical comparison, the transition state structures of **TS-Nα-3t** and **TS-Nβ-3t** at three of these levels of theory are shown in **Figure 3**. Despite earlier TSs being located for two levels of theory, similar geometries are observed.

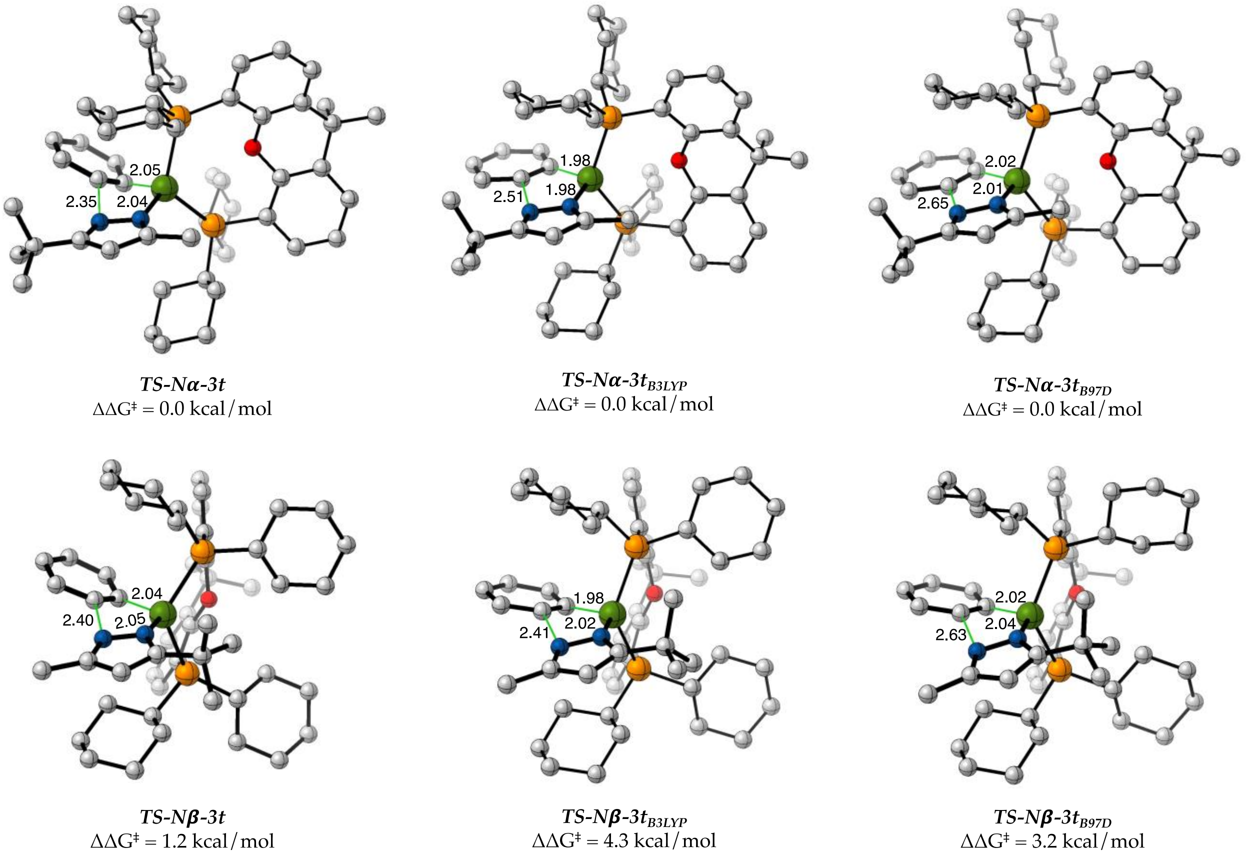

Figure 3. Comparison of arylation TSs at either N^{α} or N^{β} of **3t** across three levels of theory: (*left*) **TS-Nα-3t** and **TS-Nβ-3t** for M062X/6-311+G(d,p) PCM(MeCN) // M062X/6-31G(d), (*center*) **TS-Nα-3t$_{B3LYP}$** and **TS-Nβ-3t$_{B3LYP}$** for B3LYP-D3/6-311+G(d,p) PCM(MeCN) // B3LYP-D3/6-31G(d),[73,74] and (*right*) **TS-Nα-3t$_{B97D}$** and **TS-Nβ-3t$_{B97D}$** for ωB97XD/def2-TZVP SMD(MeCN) // B97D/def2-SVP.[75-79] Free energies provided are relative to the lower energy N^{α}-arylation TS (either **TS-Nα-3t**, **TS-Nα-3t$_{B3LYP}$**, or **TS-Nα-3t$_{B97D}$**).

To probe the origin of regioselectivity in the Cy-XantPhos (**L1**) reaction, a distortion-interaction analysis[100-102] was conducted on **TS-Nα-3t$_{B3LYP}$** and **TS-Nβ-3t$_{B3LYP}$** (**Table 13**). Cu-**L1** and **2a** were chosen as the catalyst ("Cat") fragment and the substituted pyrazole **3t** as the reactant ("Rct") fragment. The results of this analysis indicate a net distortion energy difference between TSs of 3.5 kcal/mol in favor of the TS leading to the major regioisomer (**TS-Nα-3t**). This is almost entirely an effect of the increased catalyst distortion (Distortion$_{Cat}$) required in order to accommodate the sterically bulky pyrazole substituent in **TS-Nβ-3t**, as shown visually in the

space-filling models in **Figure 5b**. The impact of sterics is also reflected in increased Pauli repulsions. Pauli repulsions is one aspect of the Int_{Other} component of our analysis, which also favors the less sterically encumbered **TS-Nα-3t** by 1.5 kcal/mol. As such, this supports our conclusion that regioselectivity under **L1** is sterically controlled.

Table 13. Distortion (ΔD)-interaction (ΔI) analysis performed on **TS-Nα-3t**$_{B3LYP}$ and **TS-Nβ-3t**$_{B3LYP}$. Green boxes represent energy components favoring the TS leading to the major N^{α}-arylated regioisomer

B3LYP-D3/6-311+G(d,p) PCM (MeCN) // B3LYP-D3/6-31G(d)								
	Distortion (kcal/mol)	$Distortion_{Cat}$ (kcal/mol)	$Distortion_{Rct}$ (kcal/mol)	Interaction (kcal/mol)	$Int_{Dispersion}$ (kcal/mol)	Int_{ESP} (kcal/mol)	Int_{Other} (kcal/mol)	Overall (kcal/mol)
Major (TS-Nα-3t)	25.9	26.1	-0.2	-40.2	-24.7	-89.9	74.4	
Minor (TS-Nβ-3t)	29.4	29.6	-0.2	-40.7	-25.8	-90.8	75.9	
$\Delta\Delta E^{\ddagger}_{major}$	**-3.5**	**-3.5**	**0.0**	**0.5**	**1.1**	**0.9**	**-1.5**	**-3.1**

Using the SEQCROW[92,93] bundle for ChimeraX, buried volume analyses were performed on the Cu(**L1**)-**3t** fragment of **TS-Nα-3t** and **TS-Nβ-3t** to gauge the steric environment faced by an incoming **2a** substrate prior to aminocupration. The **Cu-Nβ-3t** fragment of **TS-Nα-3t** has an overall buried volume of 76.5%, notably lower than the 80.6% buried volume **Cu-Nα-3t** occupies in **TS-Nβ-3t.** With the t-butyl group of **3t** protruding into the ligand space of **TS-Nβ-3t**, it is unsurprising that there exists more steric bulk in the catalytic pocket in this orientation than in **TS-Nα-3t** where the t-butyl substituent lies unencumbered outside the pocket. This is further illustrated in the topographic steric maps shown in **Figure 4**. The higher buried volume of 23.2% in the bottom right quadrant of **TS-Nβ-3t** reflects the effect of this t-butyl group especially in comparison to the less crowded 20.9% buried volume bottom right quadrant of **TS-Nα-3t.** As such, steric control is perceived to be responsible for the regioselectivity observed under **L1**.

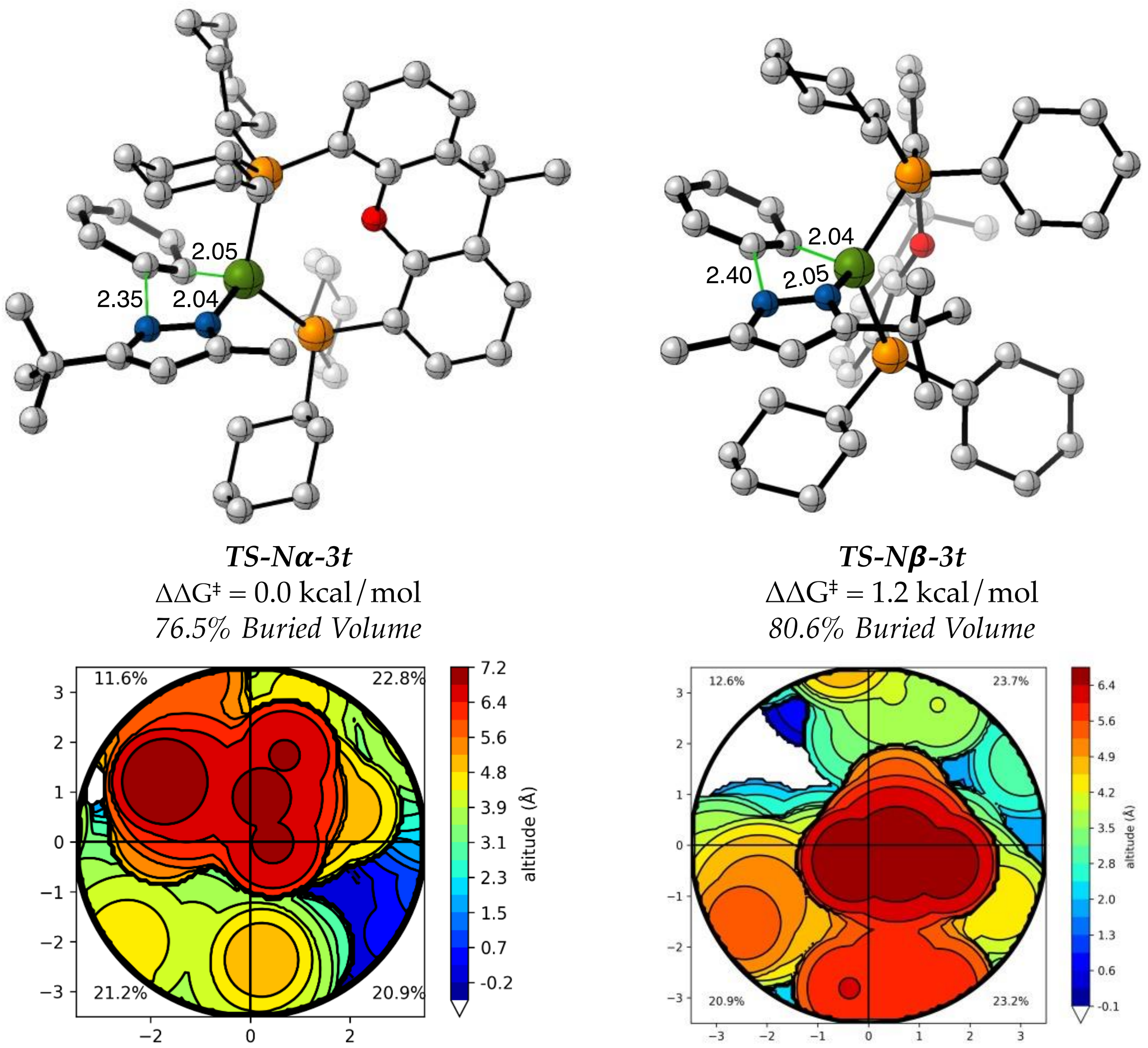

Figure 4. Steric maps depicting the catalytic pocket (Cu-**L1**-**3t**) prior to coordination of aryne **2a.** The orientation of the steric maps follows that of the above CYLviews with **2a** entering in the upper left quadrant.

An additional energy decomposition analysis was conducted on the regioisomeric TSs (**TS-Nα-3t**$_{\mathbf{B3LYP}}$ and **TS-Nβ-3t**$_{\mathbf{B3LYP}}$) from the perspective of the benzyne substrate—**2a** serving as the reactant ("Rct") fragment and Cu-pyrazolate (Cu-**L1** and **3t**) as the catalyst ("Cat") fragment. As shown in **Table 14**, the results of this analysis reveal that the major regioisomer (**TS-Nα-3t**) is favored by both distortion and interaction components. It undergoes 1.8 kcal/mol less distortion compared to the minor **TS-Nβ-3t**, which exclusively occurs within the Cu-pyrazolate portion. Additionally, benzyne **2a** engages in more favorable interactions with the Cu-pyrazolate complex

in **TS-Nα-3t** (2.5 kcal/mol more than the minor), despite there being more favorable non-covalent dispersion interactions occurring within the minor **TS-Nβ-3t.** This offers additional support for a sterically controlled system.

Table 14. Distortion (ΔD)-interaction (ΔI) analysis performed on **TS-Nα-3t**$_{B3LYP}$ and **TS-Nβ-3t**$_{B3LYP}$ with respect to benzyne **2a.** Green boxes represent energy components favoring the TS leading to the major N^{α}-arylated regioisomer.

B3LYP-D3/6-311+G(d,p) PCM (MeCN) // B3LYP-D3/6-31G(d)—Benzyne 2a as Rct Fragment								
	Distortion (kcal/mol)	Distortion$_{Cat}$ (kcal/mol)	Distortion$_{Rct}$ (kcal/mol)	Interaction (kcal/mol)	Int$_{Dispersion}$ (kcal/mol)	Int$_{ESP}$ (kcal/mol)	Int$_{Other}$ (kcal/mol)	Overall (kcal/mol)
Major (TS-Nα-3t)	11.4	8.6	2.8	-27.7	-17.4	-4.3	-6.0	
Minor (TS-Nβ-3t)	13.2	10.5	2.7	-25.2	-19.8	-0.7	-4.7	
$\Delta\Delta E^{‡}_{major}$	**-1.8**	**-1.9**	**0.1**	**-2.5**	**2.4**	**-3.6**	**-1.3**	**-4.3**

The impact of sterics in the **L1-**controlled reaction is further exemplified on the potential energy surface in the energy difference of the pre-aminocupration ground states structures. π-coordination of aryne **2a** to **Cu-Nβ** pyrazolate prior to undergoing N^{α}-arylation (**TS-Nα-3t**) yields a stable intermediate lying at -2.8 kcal/mol on the potential energy surface. Comparatively, coordination of aryne **2a** to **Cu-Nα** pyrazolate prior to undergoing N^{β}-arylation (**TS-Nβ-3t**) yields a less stable intermediate that lies at 3.2 kcal/mol on the potential energy surface. This reinforces the notion

that significant distortion is required of the Cu-**L1** catalytic pocket in order to accommodate both an incoming aryne substrate and the t-butyl substituent of the bound pyrazole.

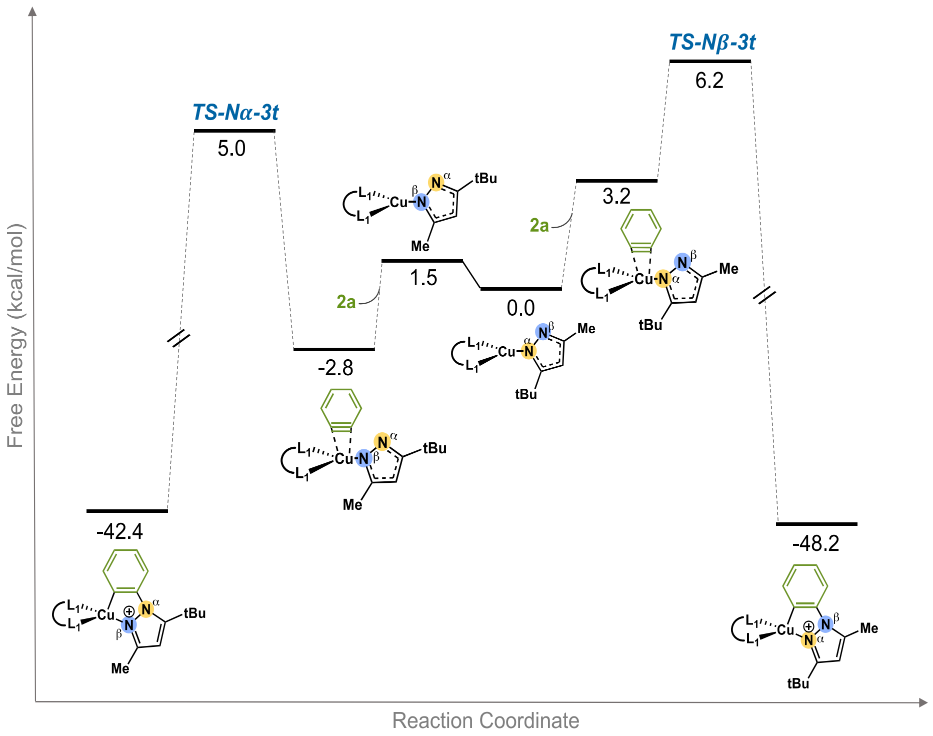

Figure 5. Potential energy surface showing the relative barriers of the regiodivergent aminocupration Ss, Cu-pyrazolate tautomers and **2a**-coordinated pre-aminocupration complexes.

7.3. Analysis of 1,10-Phenanthroline (L5)-Controlled N$^{\beta}$-Arylation

1',6'-migratory insertion transition states were also located for the addition of **2a** to (1) **4q**'s more sterically hindered N^{α} via metallo-tautomer **Cu-Nβ-4q** (**TS-Nα-4q**) and (2) **4q**'s less sterically hindered N^{β} via metallo-tautomer **Cu-Nα-4q** (**TS-Nβ-4q**). **TS-Nα-4q**, leading to the minor N^{α}-regioisomer, was found to be 1.9 kcal/mol higher in energy than **TS-Nβ-4q**, leading to the minor N^{β}-regioisomer—consistent with the experimentally observed >1:20 N^{α}:N^{β} regioselectivity under **L5** reaction conditions. Theoretical preference for the N^{β}-arylation pathway under **L5** control was validated across several levels of theory similar to the benchmarking described previously for the **L1**-catalyzed pathway (**Table 15**). Across the levels of theory explored, similar potential energy surfaces are obtained with an average $\Delta\Delta G^{\ddagger}$ (**TS-Nα-4q** and **TS-Nβ-4q**) of 2.8 kcal/mol in favor of N^{β}-arylation.

Table 15. Comparison of the relative energy difference (ΔΔG‡) of **TS-Nα-4q** and **TS-Nβ-4q** as well as the activation energy for each across various levels of theory. The chosen functional/basis set is shown in blue.

Level of Theory	ΔΔG‡ (α **vs. β**) (kcal/mol)	Barrier of **TS-Nα-4q** from rev qrc (kcal/mol)	Barrier of **TS-Nβ-4q** from rev qrc (kcal/mol)
B3LYP-D3/6-311+G** PCM (MeCN) // B3LYP-D3/6-31G* PCM (MeCN)	0.9	16.4	16.1
M062X/6-311+G** PCM (MeCN) // M062X/6-31G* PCM (MeCN)	1.4	13.7	12.3
M062X/6-311+G** PCM (MeCN) // M062X/6-31G*	1.9	13.8	11.2
M062X/6-311+G** SDD (Cu) PCM (MeCN) // M062X/6-31G* SDD (Cu)	2.7	8.4	7.4
B3LYP-D3/6-311+G** PCM (MeCN) // B3LYP-D3/6-31G*	2.8	18.8	15.4
B3LYP-D3/6-311+G** SDD (Cu) PCM (MeCN) // B3LYP-D3/6-31G* SDD (Cu)	3.6	17.6	13.5
wB97xD/def2TZVP SMD (MeCN) // wB97xD/def2SVP	4.7	21.1	16.1
wB97xD/def2TZVP SMD (MeCN) // B97D/def2SVP	4.8	20.3	14.9
**Average**	**2.8**	**16.3**	**13.4**

For comparison, the transition state structures of **TS-Nα-4q** and **TS-Nβ-4q** at three levels of theory are shown in **Figure 6**: (1) M06-2X/6-311+G(d,p) PCM (MeCN)//M06-2X/6-31G(d), (2) B3LYP-D3/6-311+G(d,p) PCM (MeCN)//B3LYP-D3/6-31G(d), and (3) ωB97XD/def2-TZVP SMD (MeCN)//B97D/def2-SVP. Of note geometrically is the discrepancy in the key C–N bonding forming distance across **TS-Nα-4q** and **TS-Nβ-4q** (2.19 Å v/s 2.33 Å) that is not present in the analogous TSs located across the other levels of theory—2.36 Å v/s 2.32 Å for **TS-Nα-4q$_{\mathbf{B3LYP}}$** and **TS-Nβ-4q$_{\mathbf{B3LYP}}$**, and 2.57 Å v/s 2.54 Å for **TS-Nα-4q$_{\mathbf{B97D}}$** and **TS-Nβ-4q$_{\mathbf{B97D}}$**. Regardless, across all levels of theory explored, arylation at the less sterically hindered N^{β} position of **4q** (**TS-Nβ-4q**) is lower in energy compared to arylation at the more sterically hindered N^{α} position (**TS-Nα-4q**).

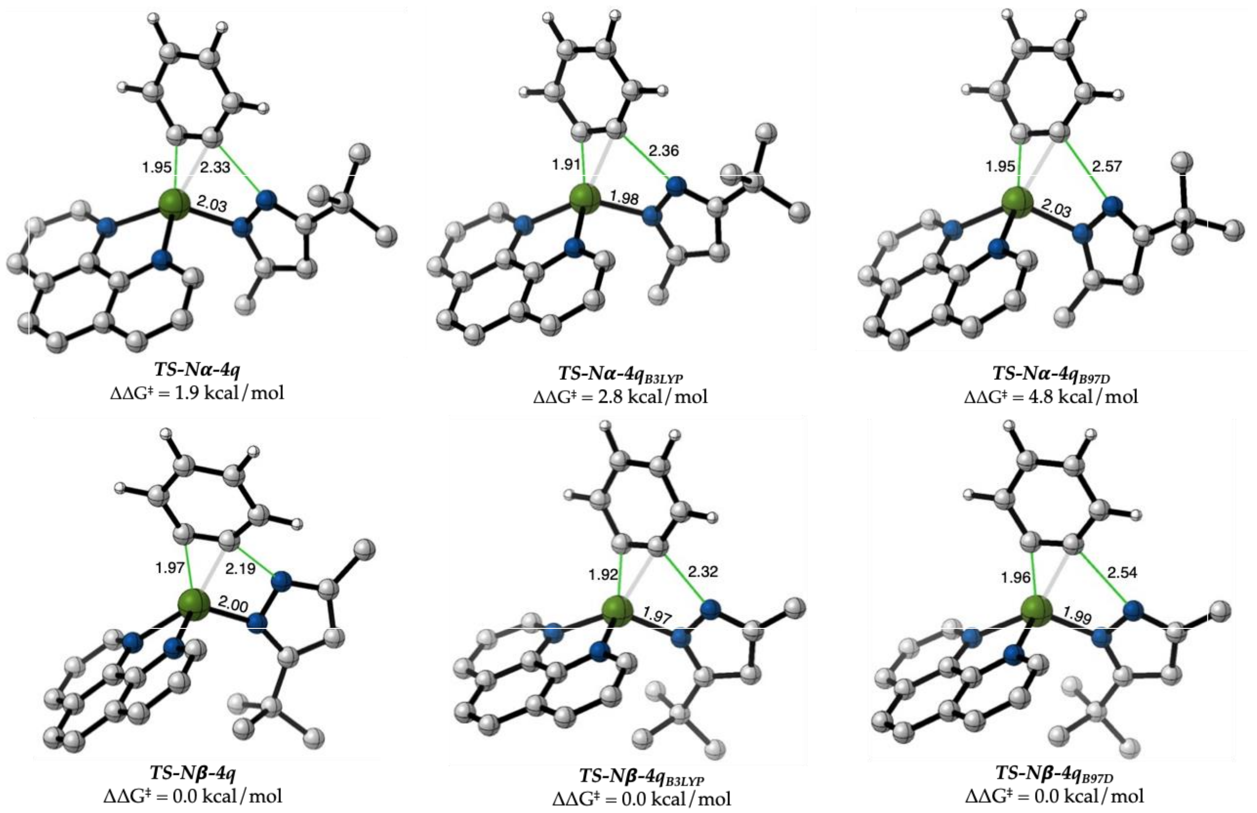

Figure 6. Comparison of arylation TSs at either N^{α} or N^{β} of **4q** across three levels of theory: (*left*) **TS-Nα-4q** and **TS-Nβ-4q** for M062X/6-311+G(d,p) PCM(MeCN) // M062X/6-31G(d), (*center*) **TS-Nα-4q$_{B3LYP}$** and **TS-Nβ-4q$_{B3LYP}$** for B3LYP-D3/6-311+G(d,p) PCM(MeCN) // B3LYP-D3/6-31G(d), and (*right*) **TS-Nα-4q$_{B97D}$** and **TS-Nβ-4q$_{B97D}$** for ωB97XD/def2-TZVP SMD(MeCN) // B97D/def2-SVP. Free energies provided are relative to the lower energy N^{β}-arylation TS (either **TS-Nβ-4q**, **TS-Nβ-4q$_{B3LYP}$**, or **TS-Nβ-4q$_{B97D}$**).

To probe the origin of regioselectivity in the 1,10-phenanthroline (**L5**) reaction, a distortion-interaction analysis was conducted on **TS-Nα-4q$_{B3LYP}$** and **TS-Nβ-4q$_{B3LYP}$** (**Table 16**). Cu-**L5** and **2a** were chosen as the catalyst ("Cat") fragment and the substituted pyrazole **4q** as the reactant ("Rct") fragment. Unsurprisingly, a negligible difference in distortion energy is observed between TSs leading to each regioisomer, and it is the interaction energy difference of 2.3 kcal/mol (favoring **TS-Nβ-4q)** that distinguishes the two TSs. Of this interaction energy difference, dispersion interactions ($Int_{Dispersion}$) are the predominate factor, making up 1.7 kcal/mol of this energy difference.

Table 16. Distortion (ΔD)-interaction (ΔI) analysis performed on **TS-Nα-4q$_{\mathbf{B3LYP}}$** and **TS-Nβ-4q$_{\mathbf{B3LYP}}$.** Green boxes represent energy components favoring the TS leading to the major N^{β}-arylated regioisomer (**TS-Nβ-4q$_{\mathbf{B3LYP}}$**), whereas the red boxes indicate those favoring the TS leading to the minor regioisomer (**TS-Nα-4q$_{\mathbf{B3LYP}}$**).

	Distortion (kcal/mol)	$\text{Distortion}_{\text{Cat}}$ (kcal/mol)	$\text{Distortion}_{\text{Rct}}$ (kcal/mol)	Interaction (kcal/mol)	$\text{Int}_{\text{Dispersion}}$ (kcal/mol)	Int_{ESP} (kcal/mol)	$\text{Int}_{\text{Other}}$ (kcal/mol)	Overall (kcal/mol)
Major (TS-Nβ-4q)	17.8	18.0	-0.2	-28.9	-13.9	-86.2	71.2	
Minor (TS-Nα-4q)	17.7	17.8	-0.1	-26.6	-12.2	-87.0	72.6	
$\Delta\Delta E^{\ddagger}_{\text{major}}$	**0.1**	**0.2**	**-0.1**	**-2.3**	**-1.7**	**0.8**	**-1.4**	**-2.2**

Using Multiwfn 3.8 software, IGMH analyses (independent gradient model based on Hirshfeld partition of molecular density) were performed to visualize the weak, non-covalent interactions between **4q** and Cu-**L5**/**2a** in **TS-Nα-4q**/**TS-Nβ-4q (Figure 7)**. The TSs leading to regioisomers N^{α} and N^{β} both exhibit T-shaped π-π interactions between phenanthroline backbone and **4q** as well as CH···N interactions between the bond-forming heteroatom of pyrazole and the nearby aromatic hydrogen of **2a**. However, in the favored TS (**TS-Nβ-4q**) only, coordination of N^{α} to Cu allows for its adjacent t-butyl group to also engage in favorable CH–π interactions with the phenanthroline backbone. As such, electronic control is perceived to be responsible for the regioselectivity observed under **L5**-control.

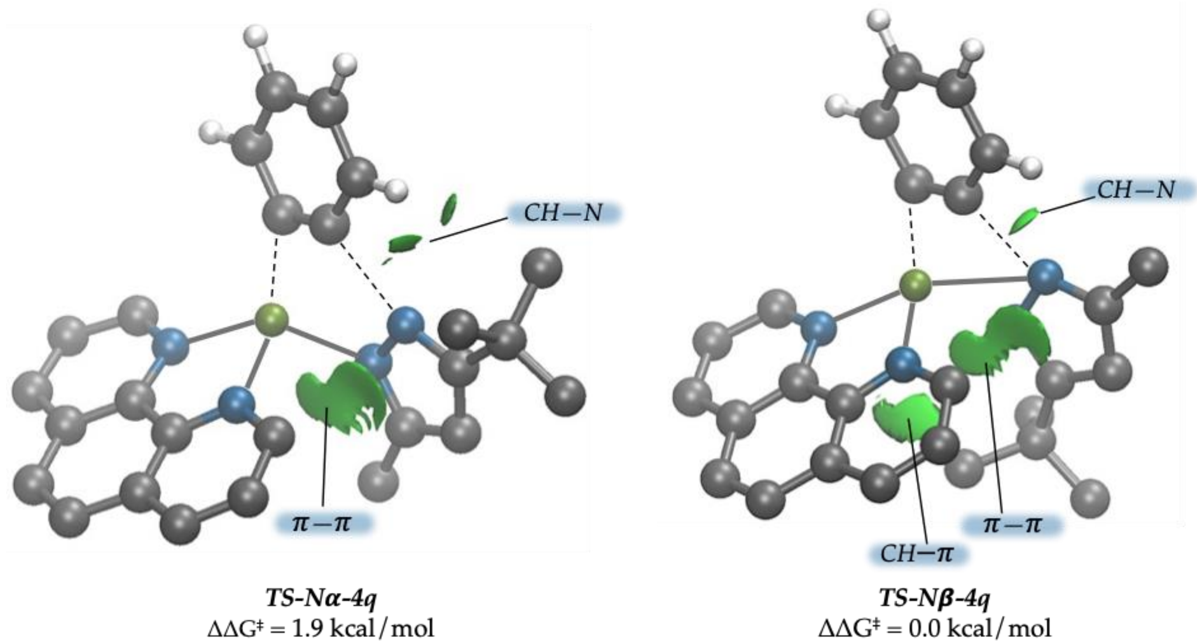

Figure 7. Non-covalent interaction plots depicting the dispersive interactions (shown in green) of pyrazole substrate **4q** with Cu-**L5** and aryne substrate **2a** at the TSs leading to each regioisomer, **TS-Nα-4q** and **TS-Nβ-4q** (isosurface of 0.009).

7.4. Summary

Table 17. Energies of TSs, intermediates and starting materials at M06-2X/6-311+G(d,p) PCM (MeCN)//M06-2X/6-31G(d), as featured in the potential energy surfaces of Figure 4 and the CYL structures within the Supporting Information. Cartesian coordinates for the following structures are available in the accompanying documentation.

Structure	Thermal Free Energy Correction (30 °C)	Electronic Energy	Corrected Free Energy	ΔG‡ (kcal/mol)
**Benzyne**	0.048492	-230.870275604	-230.821783604	*starting material*
**Pyrazole_3t**	0.178339	-422.7091131851	-422.5307741851	*starting material*
**Cu_Nα_3t**	1.01326	-4340.1840533	-4339.17079330	0.0
**Cu_Nβ_3t**	1.014369	-4340.18253660	-4339.16816760	1.5
**PRC_Nα_3t**	1.088557	-4571.08540295	-4569.99684595	3.2
**PRC_Nβ_3t**	1.090332	-4571.07759772	-4569.98726572	-2.8
**TS_Nα_3t**	1.088364	-4571.07271827	-4569.98435427	5.0
**TS_Nβ_3t**	1.087835	-4571.07027409	-4569.98243909	6.2
**Prod_Nα_3t**	1.093652	-4571.15362074	-4570.05996874	-42.4
**Prod_Nβ_3t**	1.093310	-4571.16237140	-4570.06906140	-48.2
**TS_MI_Nα_3t**	1.090497	-4571.05967414	-4569.96917714	14.5
**TS_MI_Nβ_3t**	1.089284	-4571.06631438	-4569.97703038	8.4
**TS_Nα_3t_B3LYP**	1.081245	-4572.39240232	-4571.31115732	0.9
**TS_Nβ_3t_B3LYP**	1.083106	-4572.38738869	-4571.30428269	5.2
**TS_Nα_3t_B97D**	1.038257	-4571.83505194	-4570.79679494	4.8
**TS_Nβ_3t_B97D**	1.040308	-4571.83194205	-4570.79163405	8.0
**PRC_Nα_3t_B3LYP**	1.082894	-4572.40850251	-4571.32560851	-8.2
**PRC_Nβ_3t_B3LYP**	1.083575	-4572.39582898	-4571.31225398	0.2
**PRC_Nα_3t_B97D**	1.039176	-4571.85172644	-4570.81255044	-5.0
**PRC_Nβ_3t_B97D**	1.040839	-4571.84163873	-4570.80079973	2.3
**Cu_Nα_3t_B3LYP**	1.008372	-4341.38895719	-4340.38058519	0.0
**Cu_Nβ_3t_B3LYP**	1.010043	-4341.38719746	-4340.37715446	2.2
**Cu_Nα_3t_B97D**	0.968433	-4340.91055085	-4339.94211785	0.0
**Cu_Nβ_3t_B97D**	0.968542	-4340.90819541	-4339.93965341	1.5
**Pyrazole_4q**	0.178339	-422.7091131851	-422.5307741851	*starting material*
**Cu_Nα_4q**	0.326447	-2634.12639855	-2633.79995155	0.0
**Cu_Nβ_4q**	0.326050	-2634.12487068	-2633.79882068	0.7
**PRC_Nα_4q**	0.399699	-2865.03124773	-2864.63154873	-6.7

**PRC_Nβ_4q**	0.401089	-2865.03160673	-2864.63051773	-5.5
**TS_Nα_4q**	0. 399787	-2865.00938805	-2864.60960105	7.6
**TS_Nβ_4q**	0.398857	-2865.01146681	-2864.61260981	5.7
**Prod_Nα_4q**	0.404577	-2865.09313554	-2864.68855854	-41.9
**Prod_Nβ_4q**	0.404618	-2865.10660956	-2864.70199156	-50.4
**TS_Nα_4q_B3LYP**	0.396380	-2865.67675617	-2865.28037617	--
**TS_Nβ_4q_B3LYP**	0.395344	-2865.68020910	-2865.28486510	--
**TS_Nα_4q_B97D**	0.379519	-2865.40468395	-2865.02516495	--
**TS_Nβ_4q_B97D**	0.379270	-2865.41203447	-2865.03276447	--

8. Product Isomerization Study

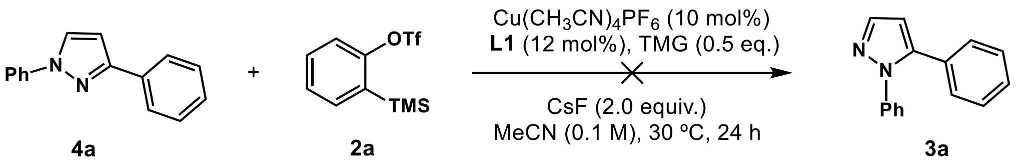

In a N_2 filled glovebox, to a 1-dram vial equipped with a magnetic stir bar was added $[Cu(CH_3CN)_4]PF_6$ (1.3 mg, 0.0034 mmol, 10 mol%), **L1** (2.4 mg, 0.004 mmol, 12 mol%) and acetonitrile (0.34 mL, 0.1 M). After stirring for 30 min, pyrazole **4a** (7.4 mg, 0.034 mmol, 1.0 equiv.) and 1,1,3,3-tetramethylguanidine (TMG, 1.9 mg, 0.017 mmol, 0.5 equiv.) were added. The resulting mixture was stirred for another 30 min followed by the addition of 2-(trimethylsilyl)phenyl trifluoromethanesulfonate (**2**) (12 µL, 0.05 mmol, 1.5 equiv.) and CsF (10 mg, 0.067 mmol, 2.0 equiv.). The vial was sealed with a Teflon-lined screw cap and stirred at 30 °C for 24 hours. Starting material **4a** was fully recovered.

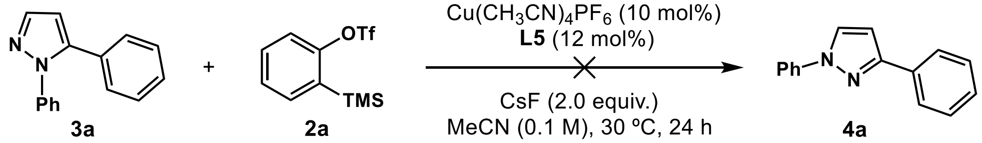

In a N_2 filled glovebox, to a 1-dram vial equipped with a magnetic stir bar was added $[Cu(CH_3CN)_4]PF_6$ (3.0 mg, 0.008 mmol, 10 mol%), **L1** (1.7 mg, 0.0096 mmol, 12 mol%) and acetonitrile (0.8 mL, 0.1 M). After stirring for 30 min, pyrazole **3a** (17.6 mg, 0.08 mmol, 1.0 equiv.) was added. The resulting mixture was stirred for another 30 min followed by the addition of 2-(trimethylsilyl)phenyl trifluoromethanesulfonate (**2**) (29 µL, 0.12 mmol, 1.5 equiv.) and CsF (24 mg, 0.16 mmol, 2.0 equiv.). The vial was sealed with a Teflon-lined screw cap and stirred at 30 °C for 24 hours. Starting material **3a** was fully recovered.

9. Reaction Compatibility Test

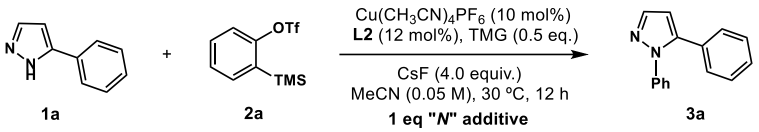

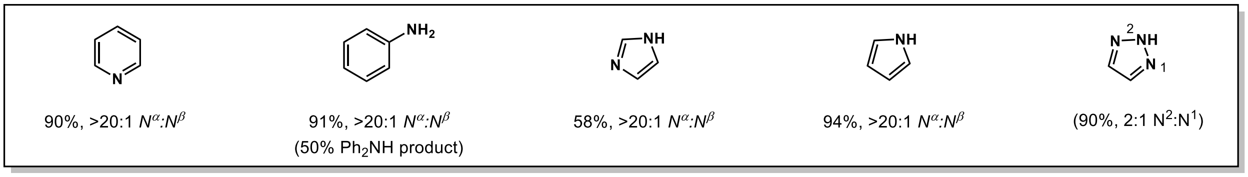

In a N_2 filled glovebox, to a 1-dram vial equipped with a magnetic stir bar was added $[Cu(CH_3CN)_4]PF_6$ (1.9 mg, 0.005 mmol, 10 mol%), **L2** (3.4 mg, 0.006 mmol, 12 mol%) and acetonitrile (1 mL, 0.1 M). After stirring for 30 min, pyrazole **1a** (7.2 mg, 0.05 mmol, 1.0 equiv.) and “N” additives (1 equiv.) were added. The resulting mixture was stirred for another 30 min followed by the addition of 2-(trimethylsilyl)phenyl trifluoromethanesulfonate (**2**) (36 μL, 0.15 mmol, 3 equiv.) and CsF (30 mg, 0.2 mmol, 4.0 equiv.). The vial was sealed with a Teflon-lined screw cap and stirred at 30 °C for 24 hours. The mixture was concentrated in vacuo. Regioselectivity (rr) was determined by ^{1}H NMR analysis of the crude mixture

10. X-Ray Crystallography Data

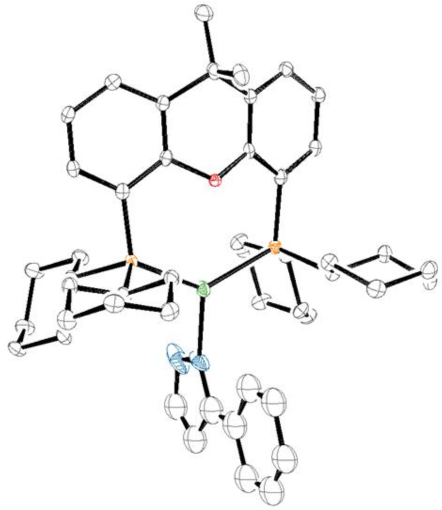

CCDC-2384544

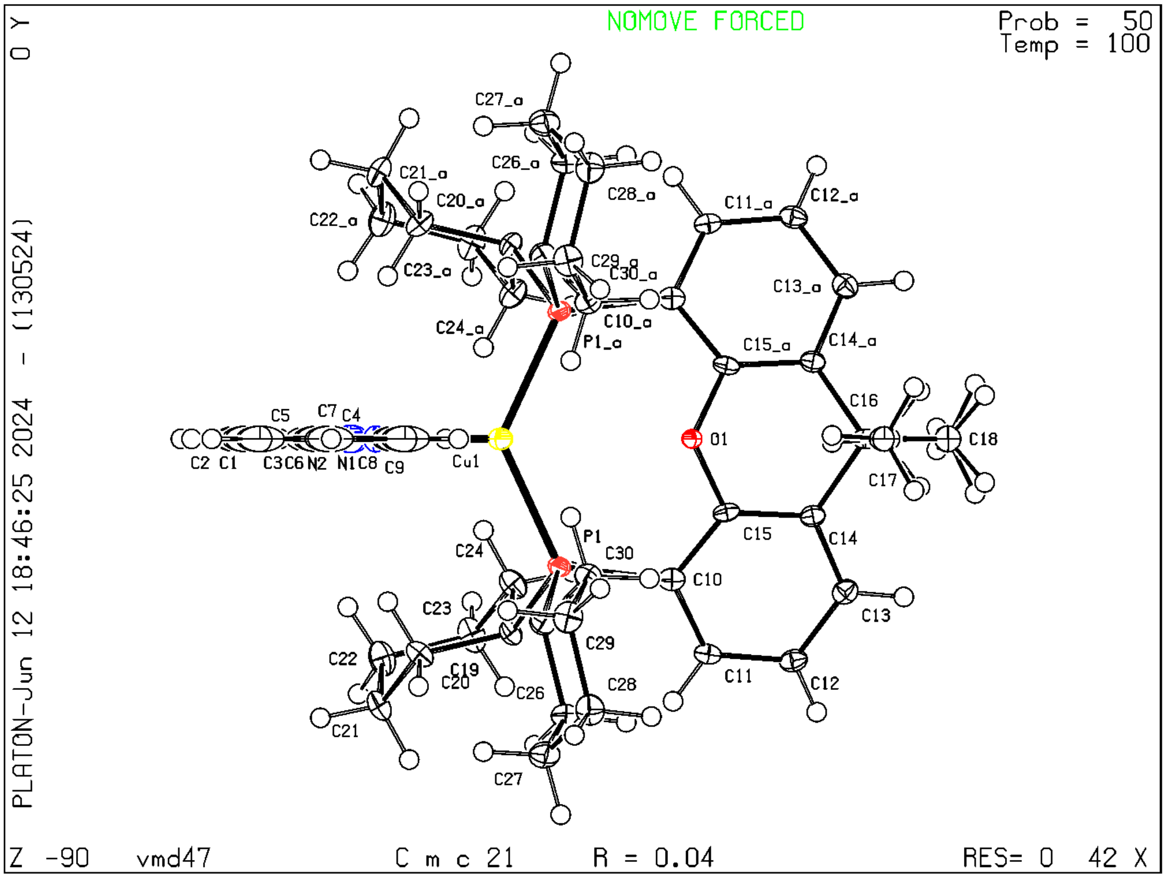

Figure 8. CyXant-Cu-N$^{\alpha}$ structure determined by X-ray diffraction

Experimental summary

A colorless crystal of approximate dimensions 0.206 x 0.094 x 0.075 mm was mounted in a cryoloop/on a glass fiber and transferred to a Bruker X8 Prospector diffractometer system. The APEX3 program package was used to determine the unit-cell parameters and for data collection (10 sec/frame scan time). The raw frame data was processed using SAINT and SADABS to yield the reflection data file. Subsequent calculations were carried out using the SHELXTL program package. The diffraction symmetry was *mmm* and the systematic absences were consistent with the orthorhombic space group $Cmc2_1$ that was later determined to be correct.

The structure was solved by direct methods and refined on F^2 by full-matrix least-squares techniques. The analytical scattering factors for neutral atoms were used throughout the analysis. Hydrogen atoms were located from a difference-Fourier map and refined (x,y,z and U_{iso}).

Least squares analysis yielded wR2 = 0.0999 and Goof = 1.027 for 238 variables refined against 4050 data (0.82 Å), R1 = 0.0397 for those 3906 data with $I > 2.0\sigma(I)$. The absolute structure was assigned by refinement of the Flack parameter.[94]

Definitions:

$wR2 = [\Sigma[w(F_o^2-F_c^2)^2] / \Sigma[w(F_o^2)^2]]^{1/2}$

$R1 = \Sigma||F_o|-|F_c|| / \Sigma|F_o|$

Goof = S = $[\Sigma[w(F_o^2-F_c^2)^2] / (n-p)]^{1/2}$ where n is the number of reflections and p is the total number of parameters refined.

The thermal ellipsoid plot is shown at the 50% probability level.

Table 18. Crystal data and structure refinement.

Identification code	CyXant-Cu-N$^{\alpha}$	
Empirical formula	C48 H63 Cu N2 O P2	
Formula weight	809.48	
Temperature	100(2) K	
Wavelength	1.54178 Å	
Crystal system	Orthorhombic	
Space group	$Cmc2_1$	
Unit cell dimensions	a = 14.3615(6) Å	α = 90°.
	b = 18.6461(9) Å	β = 90°.
	c = 15.8234(8) Å	γ = 90°.
Volume	4237.3(3) Å^3	
Z	4	
Density (calculated)	1.269 Mg/m^3	
Absorption coefficient	1.722 mm^{-1}	
F(000)	1728	
Crystal color	?	
Crystal size	0.206 x 0.094 x 0.075 mm^3	
Theta range for data collection	3.885 to 68.331°	
Index ranges	-17 <= *h* <= 17, -22 <= *k* <= 22, -19 <= *l* <= 19	
Reflections collected	44717	
Independent reflections	4050 [R(int) = 0.0812]	
Completeness to theta = 67.679°	100.0 %	
Absorption correction	Semi-empirical from equivalents	
Max. and min. transmission	0.7531 and 0.6791	
Refinement method	Full-matrix least-squares on F^2	
Data / restraints / parameters	4050 / 1 / 238	
Goodness-of-fit on F^2	1.027	
Final R indices [I>2sigma(I) = 3906 data]	R1 = 0.0397, wR2 = 0.0987	
R indices (all data, ? Å)	R1 = 0.0425, wR2 = 0.0999	
Absolute structure parameter	0.000(8)	
Extinction coefficient	n/a	
Largest diff. peak and hole	0.858 and -0.640 e.Å^{-3}	

Table 19. Atomic coordinates (x 10^4) and equivalent isotropic displacement parameters (Å^2 x 10^3).

U(eq) is defined as one third of the trace of the orthogonalized U^{ij} tensor.

	x	y	z	U(eq)
Cu(1)	5000	3121(1)	4824(1)	17(1)
P(1)	3579(1)	2762(1)	4403(1)	13(1)
O(1)	5000	1765(2)	3709(2)	12(1)
N(1)	5000	3918(4)	5662(5)	38(2)
C(11)	2606(3)	2076(2)	3054(2)	14(1)
C(13)	3298(3)	1194(2)	2133(2)	16(1)
C(12)	2533(3)	1603(2)	2378(2)	16(1)
C(28)	1986(3)	1112(2)	6128(3)	21(1)
C(19)	2842(3)	3538(2)	4110(2)	15(1)
C(20)	2606(3)	3992(2)	4893(3)	18(1)
C(15)	4182(3)	1721(2)	3243(2)	13(1)
C(21)	2033(3)	4652(2)	4632(2)	19(1)
C(10)	3436(3)	2148(2)	3506(3)	14(1)
C(29)	3016(3)	1252(2)	6278(3)	21(1)
C(22)	2557(3)	5107(2)	3983(3)	22(1)
C(30)	3468(3)	1602(2)	5505(3)	17(1)
C(27)	1484(3)	1809(2)	5897(3)	21(1)
C(26)	1918(3)	2151(2)	5122(3)	18(1)
C(17)	5000	132(3)	2945(4)	23(1)
N(2)	5000	4542(4)	5217(7)	59(2)
C(23)	2818(3)	4661(2)	3209(3)	23(1)
C(25)	2953(3)	2302(2)	5274(3)	16(1)
C(14)	4141(3)	1248(2)	2556(2)	14(1)
C(24)	3383(3)	3995(2)	3466(3)	19(1)
C(18)	5000	541(4)	1438(4)	27(1)
C(16)	5000	795(3)	2354(4)	18(1)
C(3)	5000	4029(8)	6511(8)	69(1)
C(9)	5000	2745(7)	6911(8)	69(1)
C(1)	5000	5073(8)	5793(7)	69(1)
C(4)	5000	3487(8)	7124(8)	69(1)
C(2)	5000	4805(7)	6585(8)	69(1)

C(8)	5000	2190(7)	7512(7)	69(1)
C(6)	5000	3058(7)	8567(8)	69(1)
C(7)	5000	2407(8)	8382(8)	69(1)
C(5)	5000	3642(7)	7999(7)	69(1)

Table 20. Bond lengths [Å] and angles [°].

Cu(1)-N(1)	1.992(6)
Cu(1)-P(1)	2.2484(10)
Cu(1)-P(1)#1	2.2484(10)
P(1)-C(10)	1.836(4)
P(1)-C(19)	1.851(4)
P(1)-C(25)	1.856(4)
O(1)-C(15)#1	1.389(4)
O(1)-C(15)	1.389(4)
N(1)-C(3)	1.358(13)
N(1)-N(2)	1.361(12)
C(11)-C(12)	1.389(6)
C(11)-C(10)	1.396(6)
C(11)-H(11)	0.9500
C(13)-C(14)	1.388(6)
C(13)-C(12)	1.394(6)
C(13)-H(13)	0.9500
C(12)-H(12)	0.9500
C(28)-C(29)	1.521(6)
C(28)-C(27)	1.531(6)
C(28)-H(28A)	0.9900
C(28)-H(28B)	0.9900
C(19)-C(24)	1.539(5)
C(19)-C(20)	1.539(5)
C(20)-C(21)	1.536(5)
C(20)-H(20A)	0.9900
C(20)-H(20B)	0.9900
C(15)-C(10)	1.399(6)
C(15)-C(14)	1.401(5)
C(21)-C(22)	1.532(6)
C(21)-H(21A)	0.9900
C(21)-H(21B)	0.9900
C(29)-C(30)	1.531(6)
C(29)-H(29A)	0.9900
C(29)-H(29B)	0.9900
C(22)-C(23)	1.527(6)

C(22)-H(22A)	0.9900
C(22)-H(22B)	0.9900
C(30)-C(25)	1.544(5)
C(30)-H(30A)	0.9900
C(30)-H(30B)	0.9900
C(27)-C(26)	1.517(6)
C(27)-H(27A)	0.9900
C(27)-H(27B)	0.9900
C(26)-C(25)	1.531(6)
C(26)-H(26A)	0.9900
C(26)-H(26B)	0.9900
C(17)-C(16)	1.550(8)
C(17)-H(17A)	0.9800
C(17)-H(17B)	0.9800
C(17)-H(17C)	0.9800
N(2)-C(1)	1.345(13)
C(23)-C(24)	1.539(6)
C(23)-H(23A)	0.9900
C(23)-H(23B)	0.9900
C(14)-C(16)	1.529(5)
C(24)-H(24A)	0.9900
C(24)-H(24B)	0.9900
C(18)-C(16)	1.525(9)
C(18)-H(18A)	0.9800
C(18)-H(18B)	0.9800
C(18)-H(18C)	0.9800
C(16)-C(14)#1	1.529(5)
C(3)-C(4)	1.402(19)
C(3)-C(2)	1.452(18)
C(9)-C(8)	1.405(18)
C(9)-C(4)	1.424(18)
C(9)-H(9)	0.9500
C(1)-C(2)	1.349(18)
C(1)-H(1)	0.9500
C(4)-C(5)	1.414(15)
C(2)-H(2)	0.9500
C(8)-C(7)	1.435(15)

C(8)-H(8)	0.9500
C(6)-C(7)	1.248(17)
C(6)-C(5)	1.413(18)
C(6)-H(6)	0.9500
C(7)-H(7)	0.9500
C(5)-H(5)	0.9500
N(1)-Cu(1)-P(1)	114.76(3)
N(1)-Cu(1)-P(1)#1	114.76(3)
P(1)-Cu(1)-P(1)#1	130.41(6)
C(10)-P(1)-C(19)	103.27(18)
C(10)-P(1)-C(25)	103.39(18)
C(19)-P(1)-C(25)	105.71(18)
C(10)-P(1)-Cu(1)	121.05(13)
C(19)-P(1)-Cu(1)	111.15(13)
C(25)-P(1)-Cu(1)	110.95(14)
C(15)#1-O(1)-C(15)	115.5(4)
C(3)-N(1)-N(2)	112.4(8)
C(3)-N(1)-Cu(1)	140.5(8)
N(2)-N(1)-Cu(1)	107.1(6)
C(12)-C(11)-C(10)	121.4(4)
C(12)-C(11)-H(11)	119.3
C(10)-C(11)-H(11)	119.3
C(14)-C(13)-C(12)	120.9(4)
C(14)-C(13)-H(13)	119.6
C(12)-C(13)-H(13)	119.6
C(11)-C(12)-C(13)	120.2(4)
C(11)-C(12)-H(12)	119.9
C(13)-C(12)-H(12)	119.9
C(29)-C(28)-C(27)	110.4(3)
C(29)-C(28)-H(28A)	109.6
C(27)-C(28)-H(28A)	109.6
C(29)-C(28)-H(28B)	109.6
C(27)-C(28)-H(28B)	109.6
H(28A)-C(28)-H(28B)	108.1
C(24)-C(19)-C(20)	109.9(3)
C(24)-C(19)-P(1)	108.1(3)

C(20)-C(19)-P(1)	110.8(3)
C(21)-C(20)-C(19)	110.0(3)
C(21)-C(20)-H(20A)	109.7
C(19)-C(20)-H(20A)	109.7
C(21)-C(20)-H(20B)	109.7
C(19)-C(20)-H(20B)	109.7
H(20A)-C(20)-H(20B)	108.2
O(1)-C(15)-C(10)	117.2(4)
O(1)-C(15)-C(14)	119.0(3)
C(10)-C(15)-C(14)	123.9(3)
C(22)-C(21)-C(20)	111.1(3)
C(22)-C(21)-H(21A)	109.4
C(20)-C(21)-H(21A)	109.4
C(22)-C(21)-H(21B)	109.4
C(20)-C(21)-H(21B)	109.4
H(21A)-C(21)-H(21B)	108.0
C(11)-C(10)-C(15)	116.5(4)
C(11)-C(10)-P(1)	123.5(3)
C(15)-C(10)-P(1)	120.0(3)
C(28)-C(29)-C(30)	111.2(4)
C(28)-C(29)-H(29A)	109.4
C(30)-C(29)-H(29A)	109.4
C(28)-C(29)-H(29B)	109.4
C(30)-C(29)-H(29B)	109.4
H(29A)-C(29)-H(29B)	108.0
C(23)-C(22)-C(21)	110.9(3)
C(23)-C(22)-H(22A)	109.5
C(21)-C(22)-H(22A)	109.5
C(23)-C(22)-H(22B)	109.5
C(21)-C(22)-H(22B)	109.5
H(22A)-C(22)-H(22B)	108.1
C(29)-C(30)-C(25)	110.2(3)
C(29)-C(30)-H(30A)	109.6
C(25)-C(30)-H(30A)	109.6
C(29)-C(30)-H(30B)	109.6
C(25)-C(30)-H(30B)	109.6
H(30A)-C(30)-H(30B)	108.1

C(26)-C(27)-C(28)	110.8(4)
C(26)-C(27)-H(27A)	109.5
C(28)-C(27)-H(27A)	109.5
C(26)-C(27)-H(27B)	109.5
C(28)-C(27)-H(27B)	109.5
H(27A)-C(27)-H(27B)	108.1
C(27)-C(26)-C(25)	110.4(3)
C(27)-C(26)-H(26A)	109.6
C(25)-C(26)-H(26A)	109.6
C(27)-C(26)-H(26B)	109.6
C(25)-C(26)-H(26B)	109.6
H(26A)-C(26)-H(26B)	108.1
C(16)-C(17)-H(17A)	109.5
C(16)-C(17)-H(17B)	109.5
H(17A)-C(17)-H(17B)	109.5
C(16)-C(17)-H(17C)	109.5
H(17A)-C(17)-H(17C)	109.5
H(17B)-C(17)-H(17C)	109.5
C(1)-N(2)-N(1)	106.2(10)
C(22)-C(23)-C(24)	110.9(3)
C(22)-C(23)-H(23A)	109.5
C(24)-C(23)-H(23A)	109.5
C(22)-C(23)-H(23B)	109.5
C(24)-C(23)-H(23B)	109.5
H(23A)-C(23)-H(23B)	108.0
C(26)-C(25)-C(30)	110.3(3)
C(26)-C(25)-P(1)	116.0(3)
C(30)-C(25)-P(1)	109.5(3)
C(13)-C(14)-C(15)	117.2(3)
C(13)-C(14)-C(16)	124.2(4)
C(15)-C(14)-C(16)	118.5(4)
C(19)-C(24)-C(23)	110.8(3)
C(19)-C(24)-H(24A)	109.5
C(23)-C(24)-H(24A)	109.5
C(19)-C(24)-H(24B)	109.5
C(23)-C(24)-H(24B)	109.5
H(24A)-C(24)-H(24B)	108.1

C(16)-C(18)-H(18A)	109.5
C(16)-C(18)-H(18B)	109.5
H(18A)-C(18)-H(18B)	109.5
C(16)-C(18)-H(18C)	109.5
H(18A)-C(18)-H(18C)	109.5
H(18B)-C(18)-H(18C)	109.5
C(18)-C(16)-C(14)#1	111.8(3)
C(18)-C(16)-C(14)	111.8(3)
C(14)#1-C(16)-C(14)	107.5(5)
C(18)-C(16)-C(17)	109.0(5)
C(14)#1-C(16)-C(17)	108.3(3)
C(14)-C(16)-C(17)	108.3(3)
N(1)-C(3)-C(4)	125.1(11)
N(1)-C(3)-C(2)	103.4(12)
C(4)-C(3)-C(2)	131.5(11)
C(8)-C(9)-C(4)	123.7(11)
C(8)-C(9)-H(9)	118.2
C(4)-C(9)-H(9)	118.2
N(2)-C(1)-C(2)	110.9(13)
N(2)-C(1)-H(1)	124.5
C(2)-C(1)-H(1)	124.5
C(3)-C(4)-C(5)	122.0(13)
C(3)-C(4)-C(9)	122.4(11)
C(5)-C(4)-C(9)	115.6(13)
C(1)-C(2)-C(3)	107.1(11)
C(1)-C(2)-H(2)	126.5
C(3)-C(2)-H(2)	126.5
C(9)-C(8)-C(7)	116.2(12)
C(9)-C(8)-H(8)	121.9
C(7)-C(8)-H(8)	121.9
C(7)-C(6)-C(5)	126.9(13)
C(7)-C(6)-H(6)	116.5
C(5)-C(6)-H(6)	116.5
C(6)-C(7)-C(8)	120.0(14)
C(6)-C(7)-H(7)	120.0
C(8)-C(7)-H(7)	120.0
C(6)-C(5)-C(4)	117.7(12)

C(6)-C(5)-H(5)	121.2
C(4)-C(5)-H(5)	121.2

Symmetry transformations used to generate equivalent atoms:

#1 -x+1,y,z

Table 21. Anisotropic displacement parameters ($Å^2 \times 10^3$).

The anisotropic displacement factor exponent takes the form: $-2\pi^2[h^2 a^{*2}U^{11} + ... + 2 h k a^* b^* U^{12}]$

	U^{11}	U^{22}	U^{33}	U^{23}	U^{13}	U^{12}
Cu(1)	12(1)	16(1)	23(1)	-3(1)	0	0
P(1)	10(1)	13(1)	15(1)	0(1)	1(1)	1(1)
O(1)	9(2)	16(2)	12(2)	-3(1)	0	0
N(1)	18(3)	35(4)	60(4)	-31(3)	0	0
C(11)	10(2)	16(2)	17(2)	4(2)	1(2)	1(2)
C(13)	16(2)	17(2)	15(2)	2(2)	1(2)	-3(2)
C(12)	13(2)	20(2)	15(2)	3(2)	-2(2)	-1(2)
C(28)	25(2)	20(2)	18(2)	3(2)	4(2)	-3(2)
C(19)	16(2)	12(2)	17(2)	-1(2)	1(2)	4(2)
C(20)	17(2)	19(2)	17(2)	2(2)	4(2)	5(2)
C(15)	9(2)	15(2)	15(2)	4(2)	-1(2)	-2(2)
C(21)	23(2)	16(2)	19(2)	-2(2)	4(2)	5(2)
C(10)	13(2)	15(2)	14(2)	4(2)	1(2)	-1(2)
C(29)	26(2)	19(2)	18(2)	4(2)	-2(2)	1(2)
C(22)	32(2)	16(2)	19(2)	2(2)	1(2)	6(2)
C(30)	17(2)	17(2)	17(2)	2(2)	-1(2)	2(2)
C(27)	18(2)	23(2)	23(2)	2(2)	5(2)	-5(2)
C(26)	14(2)	20(2)	20(2)	2(2)	1(2)	0(2)
C(17)	16(3)	16(3)	38(4)	-1(3)	0	0
N(2)	20(3)	37(4)	119(7)	-37(4)	0	0
C(23)	32(2)	20(2)	18(2)	2(2)	2(2)	6(2)
C(25)	15(2)	15(2)	16(2)	1(2)	1(2)	2(2)
C(14)	12(2)	14(2)	14(2)	2(1)	0(2)	-1(2)
C(24)	22(2)	19(2)	16(2)	3(2)	3(2)	5(2)
C(18)	18(3)	30(3)	32(4)	-13(3)	0	0
C(16)	10(3)	21(3)	25(3)	-3(2)	0	0
C(3)	18(1)	116(3)	74(2)	-35(2)	0	0
C(9)	18(1)	116(3)	74(2)	-35(2)	0	0
C(1)	18(1)	116(3)	74(2)	-35(2)	0	0
C(4)	18(1)	116(3)	74(2)	-35(2)	0	0
C(2)	18(1)	116(3)	74(2)	-35(2)	0	0
C(8)	18(1)	116(3)	74(2)	-35(2)	0	0

C(6)	18(1)	116(3)	74(2)	-35(2)	0	0
C(7)	18(1)	116(3)	74(2)	-35(2)	0	0
C(5)	18(1)	116(3)	74(2)	-35(2)	0	0

Table 22. Hydrogen coordinates (x 10^4) and isotropic displacement parameters (Å^2 x 10^3).

	x	y	z	U(eq)
H(11)	2081	2355	3212	17
H(13)	3242	873	1669	19
H(12)	1960	1559	2083	19
H(28A)	1704	905	6644	25
H(28B)	1911	760	5663	25
H(20A)	2246	3700	5301	21
H(20B)	3188	4149	5173	21
H(21A)	1432	4493	4388	23
H(21B)	1896	4946	5138	23
H(29A)	3335	793	6403	25
H(29B)	3090	1570	6774	25
H(22A)	2162	5516	3806	27
H(22B)	3129	5304	4244	27
H(30A)	3447	1267	5020	21
H(30B)	4130	1710	5629	21
H(27A)	819	1707	5786	26
H(27B)	1520	2147	6379	26
H(26A)	1592	2605	4990	22
H(26B)	1848	1825	4631	22
H(17A)	4463	-172	2813	35
H(17B)	4961	292	3535	35
H(17C)	5576	-141	2862	35
H(23A)	3189	4958	2814	28
H(23B)	2244	4509	2912	28
H(24A)	3520	3703	2958	23
H(24B)	3983	4147	3716	23
H(18A)	4514	178	1361	40
H(18B)	5608	333	1301	40
H(18C)	4878	949	1064	40
H(9)	5000	2618	6329	83
H(1)	5000	5570	5660	83
H(2)	5000	5073	7095	83

H(8)	5000	1699	7351	83
H(6)	5000	3172	9152	83
H(7)	5000	2056	8816	83
H(5)	5000	4123	8197	83

Table 23. Torsion angles [°].

C(10)-C(11)-C(12)-C(13)	-0.6(6)
C(14)-C(13)-C(12)-C(11)	0.1(6)
C(10)-P(1)-C(19)-C(24)	-78.7(3)
C(25)-P(1)-C(19)-C(24)	173.1(3)
Cu(1)-P(1)-C(19)-C(24)	52.6(3)
C(10)-P(1)-C(19)-C(20)	160.9(3)
C(25)-P(1)-C(19)-C(20)	52.7(3)
Cu(1)-P(1)-C(19)-C(20)	-67.8(3)
C(24)-C(19)-C(20)-C(21)	58.1(4)
P(1)-C(19)-C(20)-C(21)	177.4(3)
C(15)#1-O(1)-C(15)-C(10)	-143.2(3)
C(15)#1-O(1)-C(15)-C(14)	38.0(6)
C(19)-C(20)-C(21)-C(22)	-57.8(4)
C(12)-C(11)-C(10)-C(15)	-0.2(6)
C(12)-C(11)-C(10)-P(1)	180.0(3)
O(1)-C(15)-C(10)-C(11)	-177.3(3)
C(14)-C(15)-C(10)-C(11)	1.4(6)
O(1)-C(15)-C(10)-P(1)	2.5(5)
C(14)-C(15)-C(10)-P(1)	-178.7(3)
C(19)-P(1)-C(10)-C(11)	-34.7(4)
C(25)-P(1)-C(10)-C(11)	75.3(4)
Cu(1)-P(1)-C(10)-C(11)	-159.8(3)
C(19)-P(1)-C(10)-C(15)	145.4(3)
C(25)-P(1)-C(10)-C(15)	-104.6(3)
Cu(1)-P(1)-C(10)-C(15)	20.3(4)
C(27)-C(28)-C(29)-C(30)	-56.8(5)
C(20)-C(21)-C(22)-C(23)	56.6(5)
C(28)-C(29)-C(30)-C(25)	56.5(5)
C(29)-C(28)-C(27)-C(26)	57.6(5)
C(28)-C(27)-C(26)-C(25)	-58.2(5)
C(3)-N(1)-N(2)-C(1)	0.000(3)
Cu(1)-N(1)-N(2)-C(1)	180.000(3)
C(21)-C(22)-C(23)-C(24)	-55.6(5)
C(27)-C(26)-C(25)-C(30)	57.6(4)
C(27)-C(26)-C(25)-P(1)	-177.1(3)

C(29)-C(30)-C(25)-C(26)	-56.6(4)
C(29)-C(30)-C(25)-P(1)	174.6(3)
C(10)-P(1)-C(25)-C(26)	-59.3(3)
C(19)-P(1)-C(25)-C(26)	48.9(3)
Cu(1)-P(1)-C(25)-C(26)	169.5(3)
C(10)-P(1)-C(25)-C(30)	66.4(3)
C(19)-P(1)-C(25)-C(30)	174.6(3)
Cu(1)-P(1)-C(25)-C(30)	-64.8(3)
C(12)-C(13)-C(14)-C(15)	1.0(6)
C(12)-C(13)-C(14)-C(16)	177.1(4)
O(1)-C(15)-C(14)-C(13)	176.9(3)
C(10)-C(15)-C(14)-C(13)	-1.8(6)
O(1)-C(15)-C(14)-C(16)	0.6(6)
C(10)-C(15)-C(14)-C(16)	-178.1(4)
C(20)-C(19)-C(24)-C(23)	-57.8(4)
P(1)-C(19)-C(24)-C(23)	-178.7(3)
C(22)-C(23)-C(24)-C(19)	56.6(5)
C(13)-C(14)-C(16)-C(18)	26.1(6)
C(15)-C(14)-C(16)-C(18)	-157.9(4)
C(13)-C(14)-C(16)-C(14)#1	149.0(3)
C(15)-C(14)-C(16)-C(14)#1	-34.9(6)
C(13)-C(14)-C(16)-C(17)	-94.1(5)
C(15)-C(14)-C(16)-C(17)	81.9(5)
N(2)-N(1)-C(3)-C(4)	180.000(3)
Cu(1)-N(1)-C(3)-C(4)	0.000(6)
N(2)-N(1)-C(3)-C(2)	0.000(3)
Cu(1)-N(1)-C(3)-C(2)	180.000(4)
N(1)-N(2)-C(1)-C(2)	0.000(5)
N(1)-C(3)-C(4)-C(5)	180.000(4)
C(2)-C(3)-C(4)-C(5)	0.000(6)
N(1)-C(3)-C(4)-C(9)	0.000(6)
C(2)-C(3)-C(4)-C(9)	180.000(4)
C(8)-C(9)-C(4)-C(3)	180.000(4)
C(8)-C(9)-C(4)-C(5)	0.000(5)
N(2)-C(1)-C(2)-C(3)	0.000(4)
N(1)-C(3)-C(2)-C(1)	0.000(4)
C(4)-C(3)-C(2)-C(1)	180.000(4)

C(4)-C(9)-C(8)-C(7)	0.000(4)
C(5)-C(6)-C(7)-C(8)	0.000(6)
C(9)-C(8)-C(7)-C(6)	0.000(6)
C(7)-C(6)-C(5)-C(4)	0.000(6)
C(3)-C(4)-C(5)-C(6)	180.000(4)
C(9)-C(4)-C(5)-C(6)	0.000(5)

Symmetry transformations used to generate equivalent atoms:

#1 -x+1,y,z

Table 24. Hydrogen bonds [Å and °].

D-H...A	d(D-H)	d(H...A)	d(D...A)	<(DHA)

11. General Procedure to Prepare Aryne Precursors

The synthesis of trifluoromethanesulfonate (Kobayashi precursors) were performed using standard literature-reported procedures.[95]

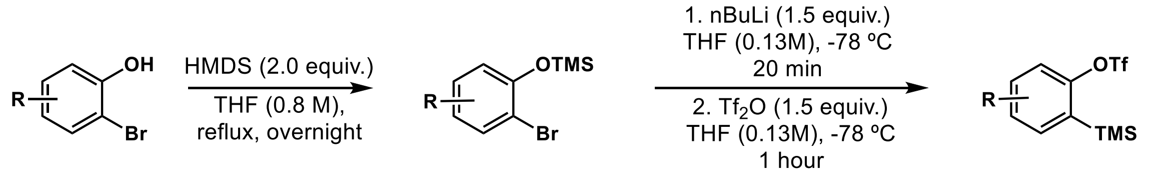

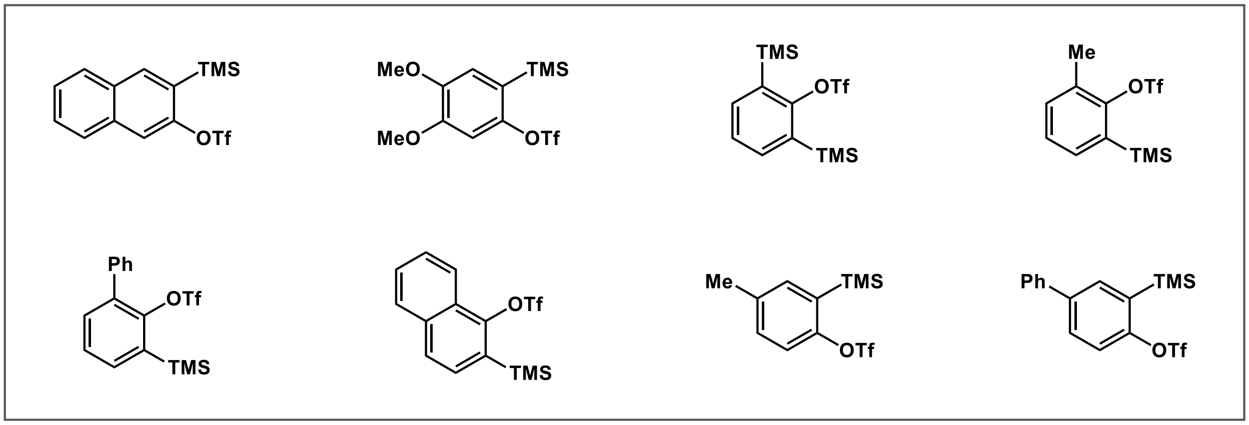

The synthesis of triaryoxonium ions (Smith precursors) were performed using standard literature-reported procedures.[45]

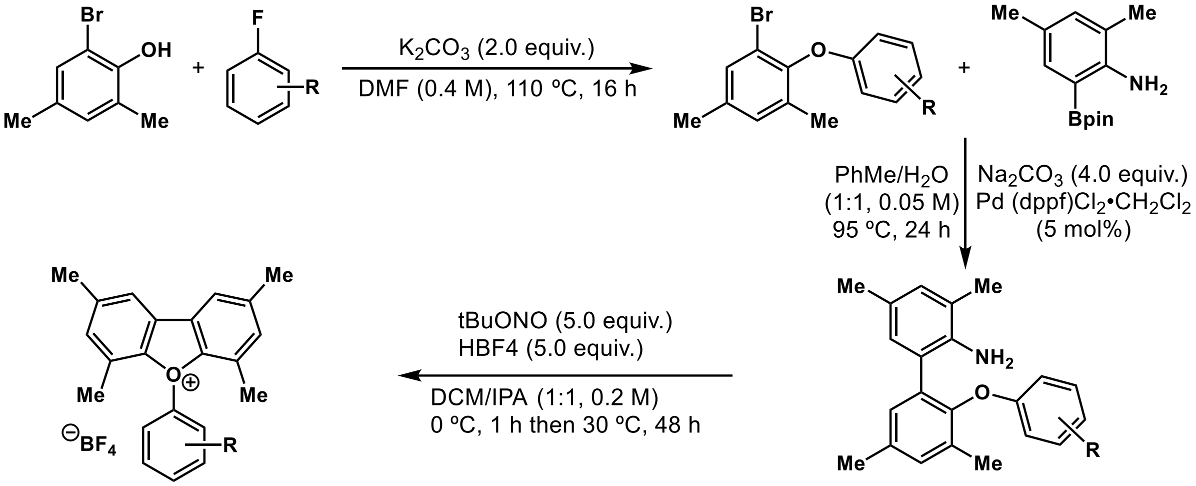

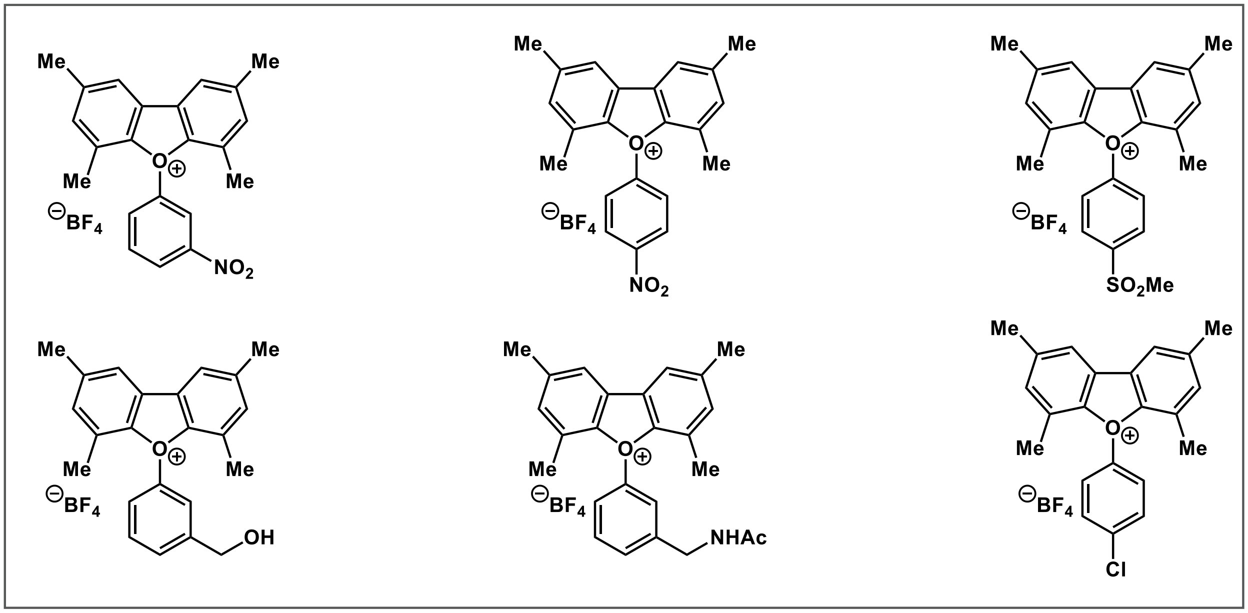

12. NMR Spectra

1,5-Diphenyl-1*H*-pyrazole (3a)

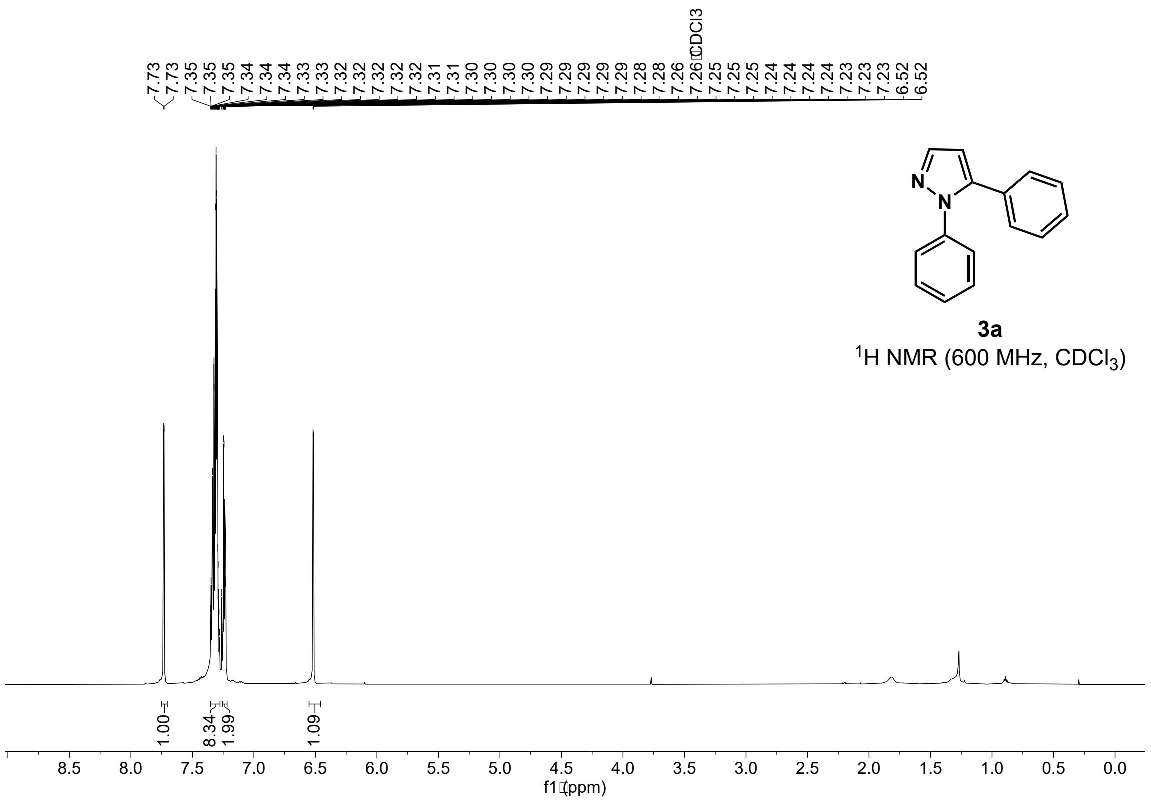

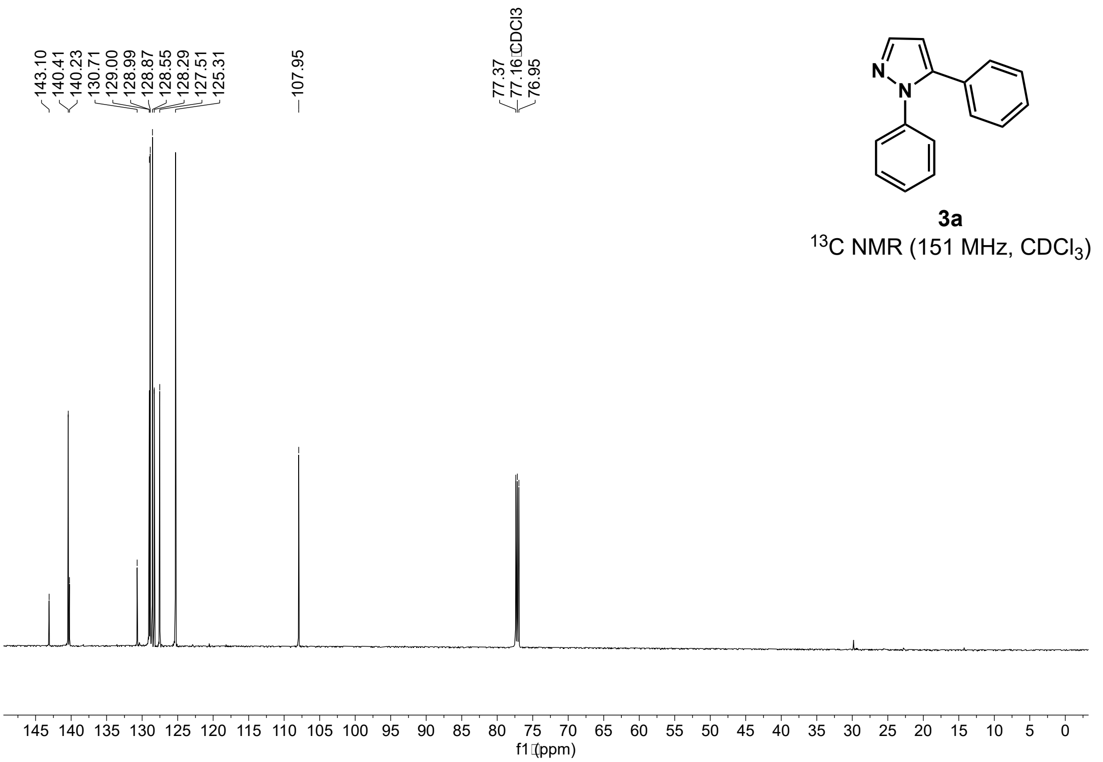

5-Methyl-1-phenyl-1*H*-pyrazole (3b)

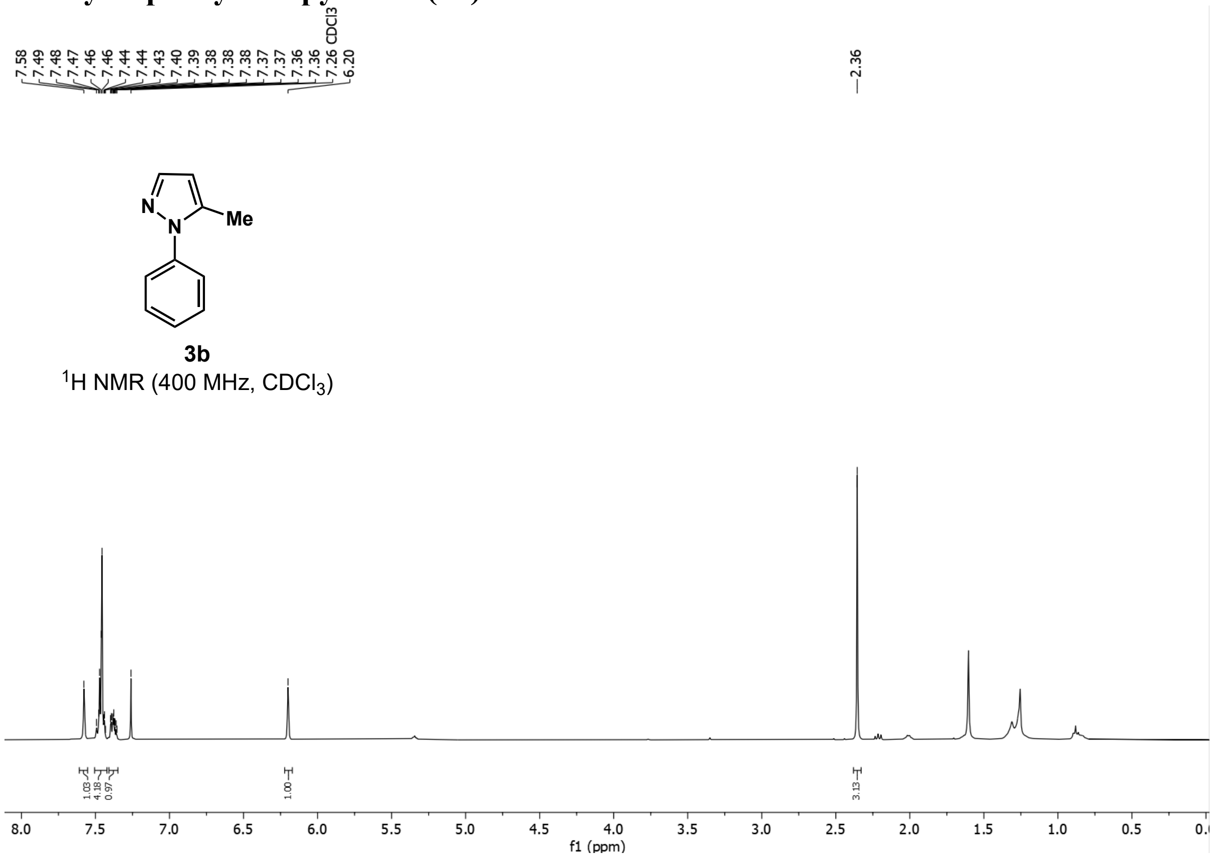

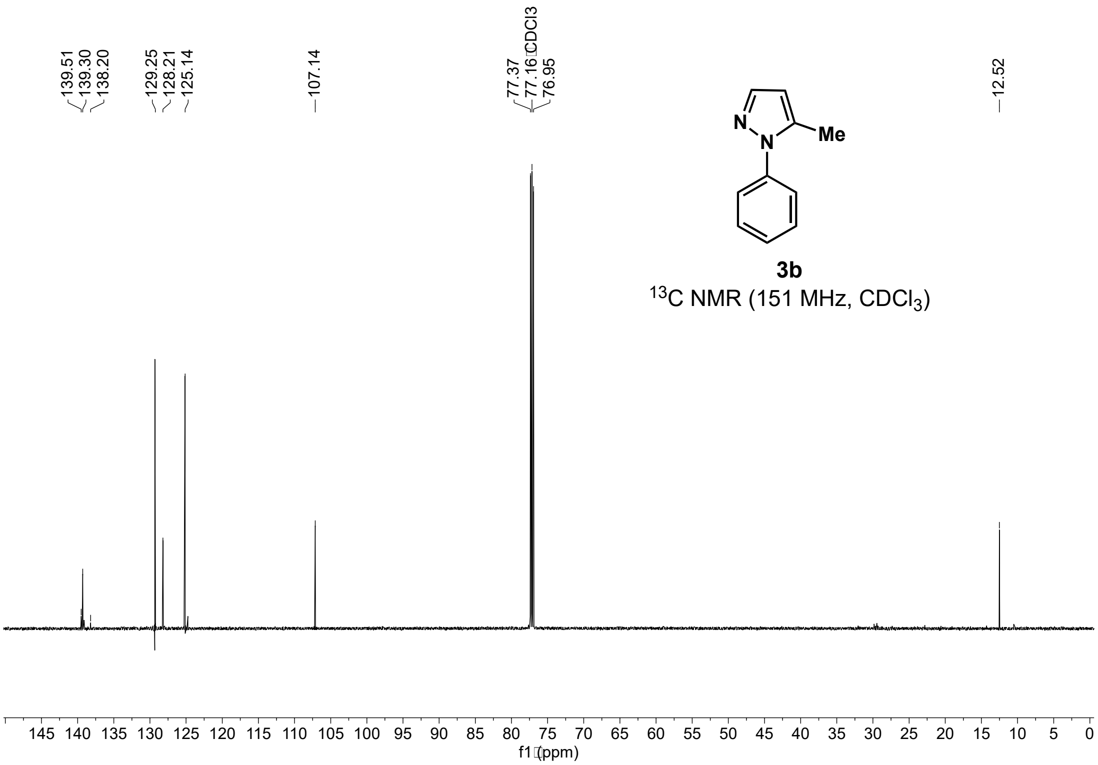

5-Cyclopropyl-1-phenyl-1*H*-pyrazole (3c)

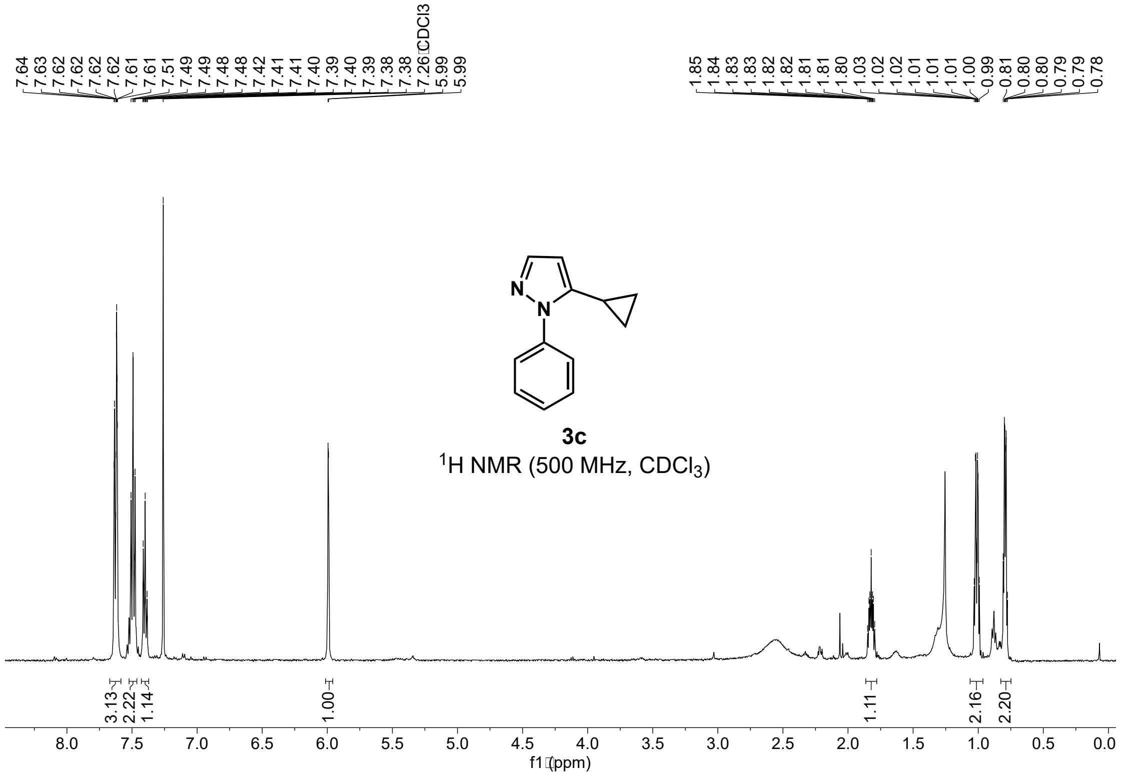

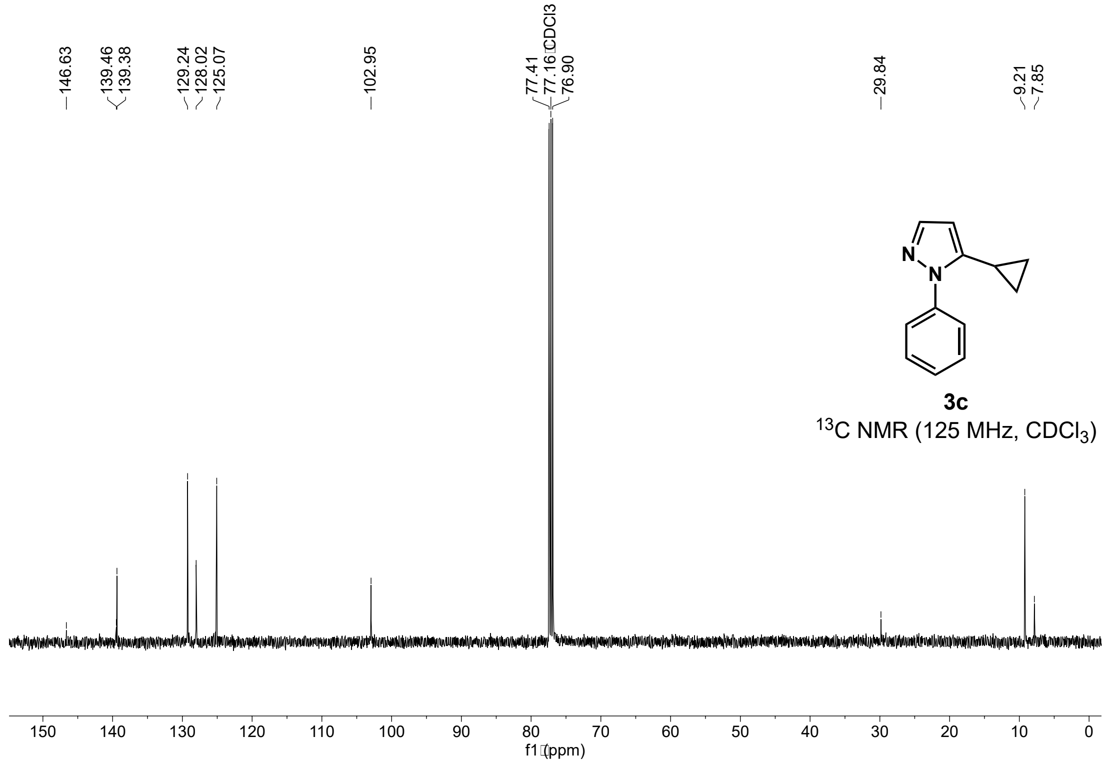

5-Isopropyl-1-phenyl-1*H*-pyrazole (3d)

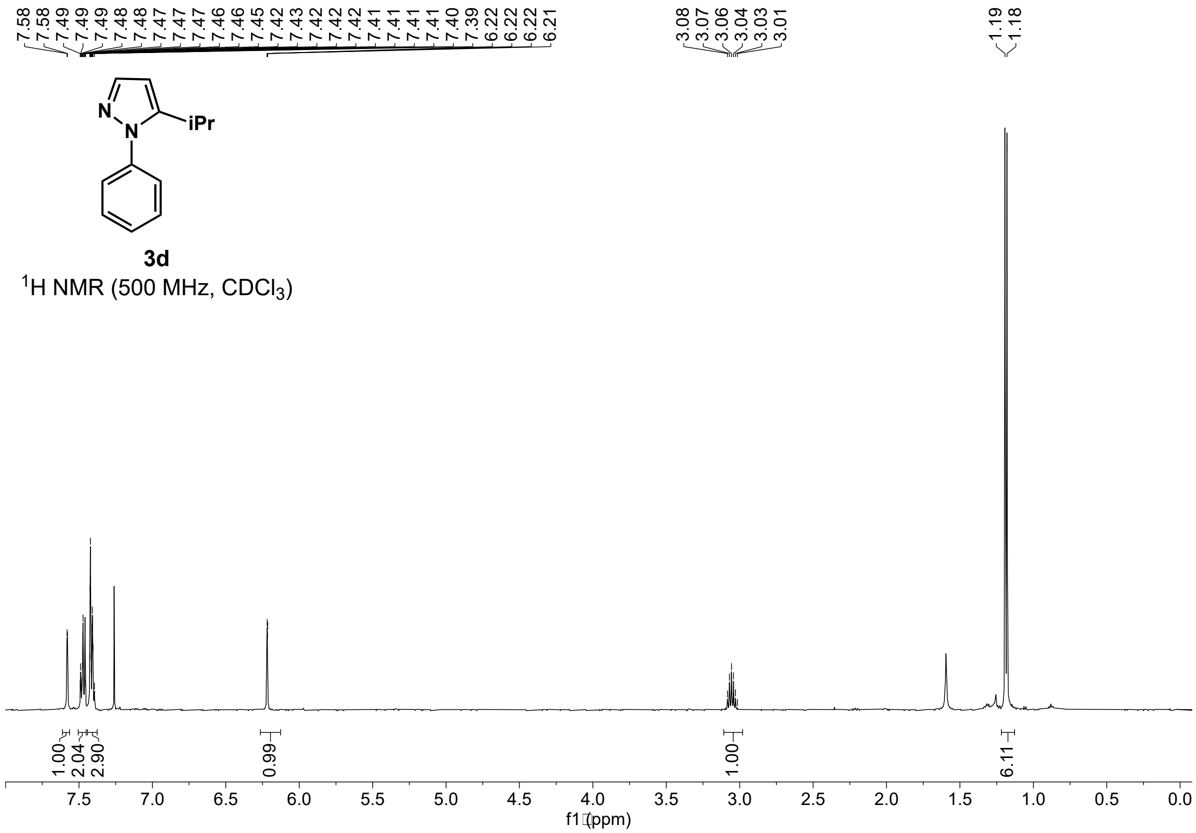

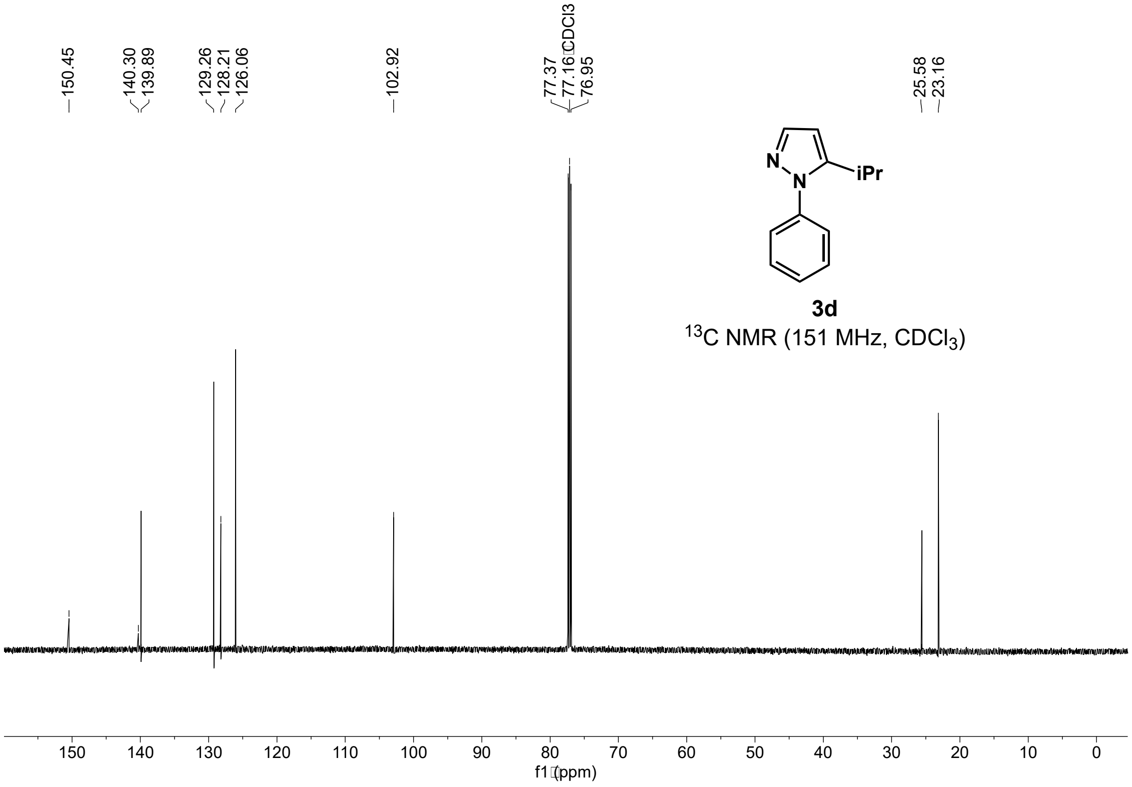

5-(Tert-butyl)-1-phenyl-1*H*-pyrazole (3e)

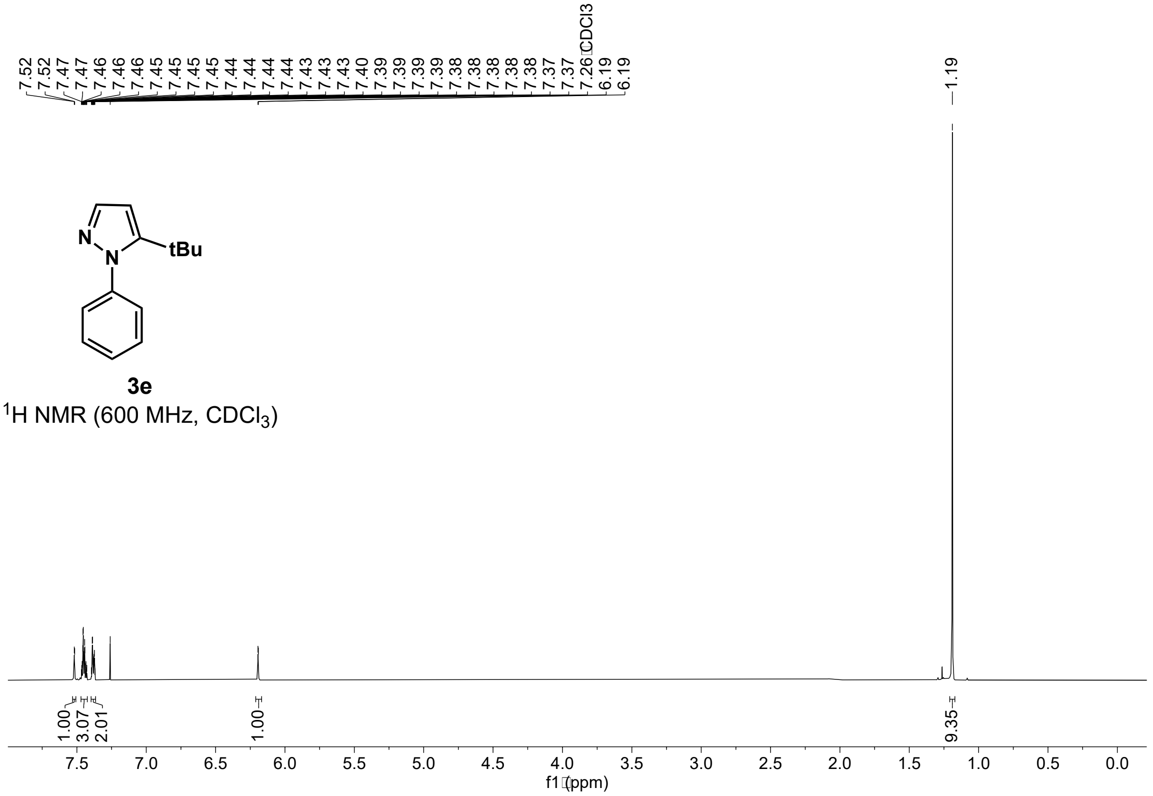

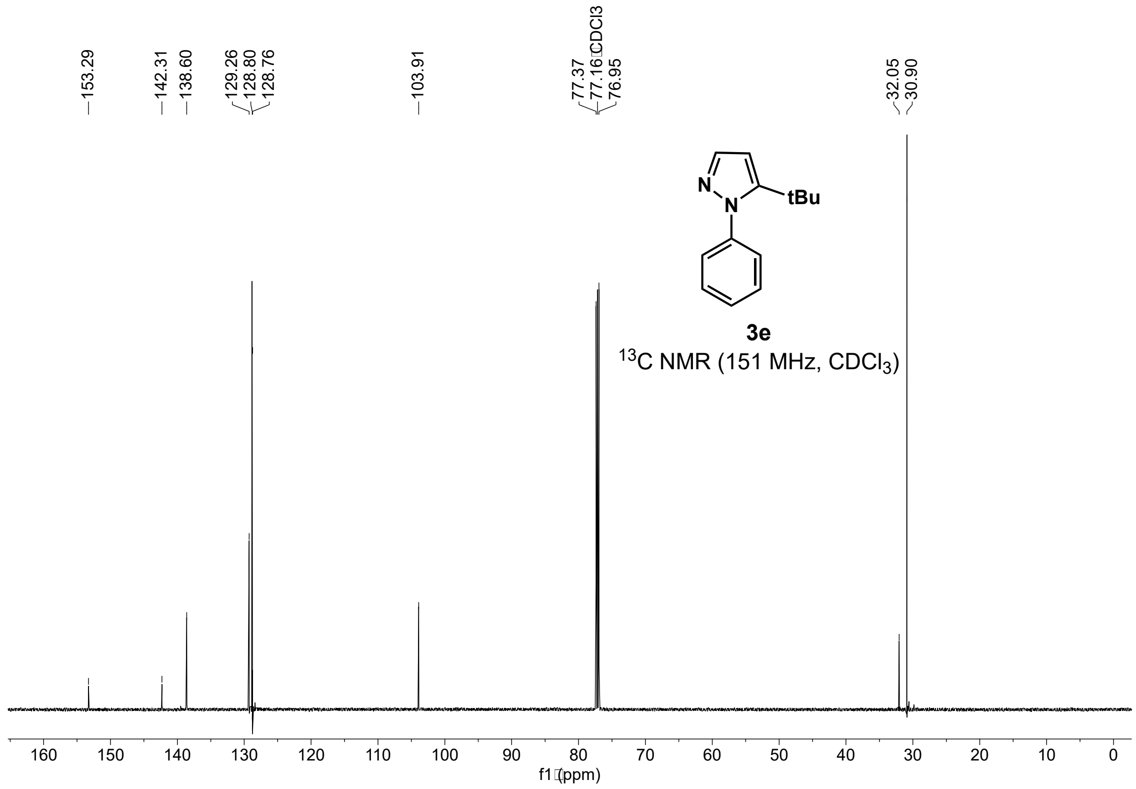

5-Bromo-1-phenyl-1*H*-pyrazole (3f)

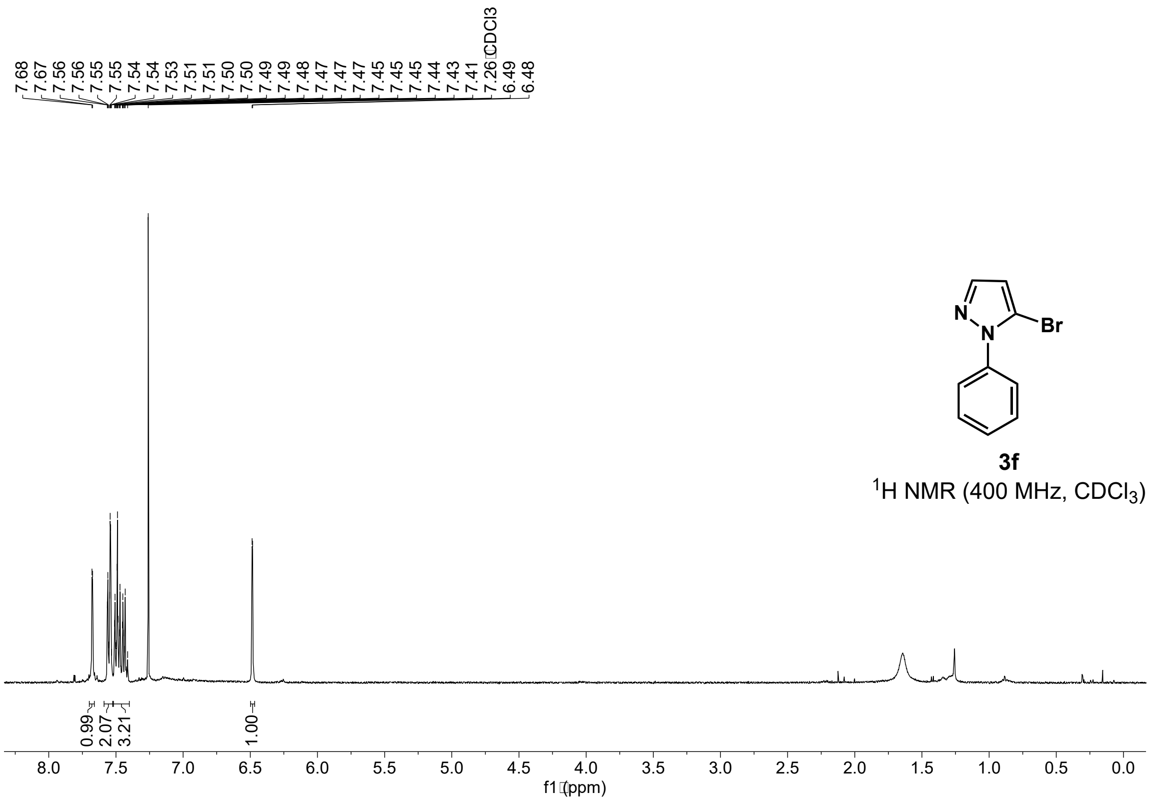

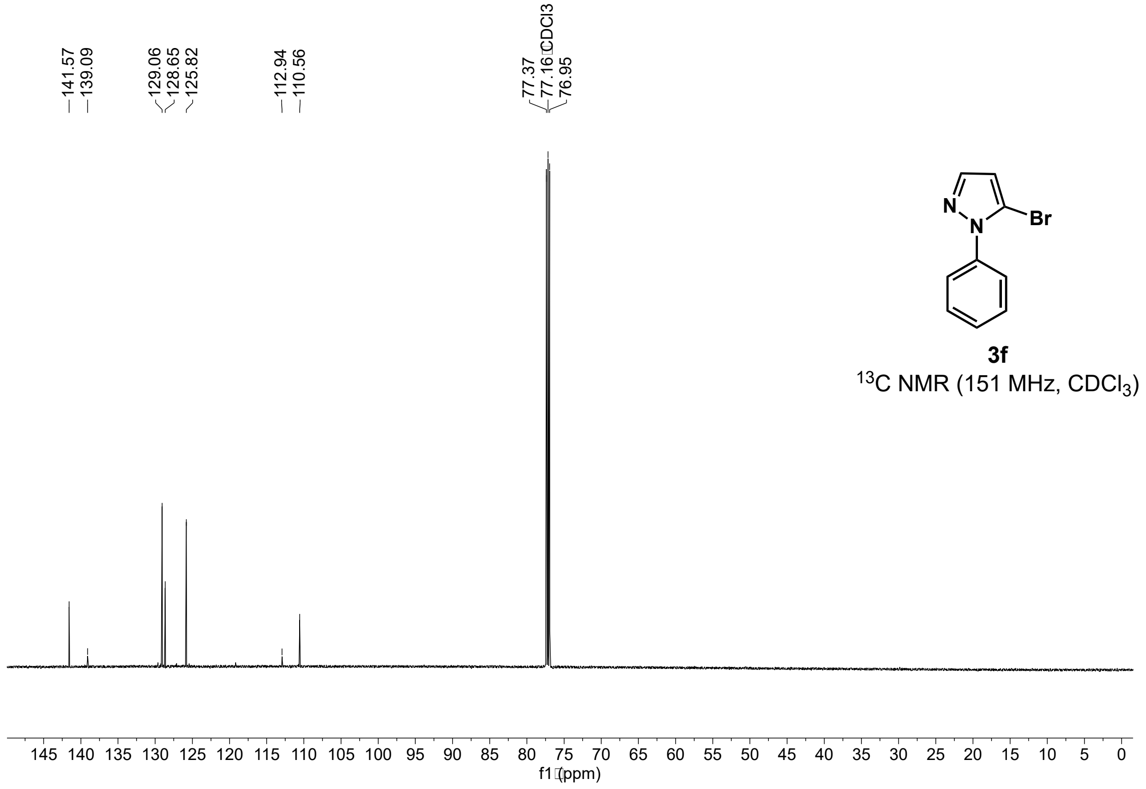

5-Iodo-1-phenyl-1*H*-pyrazole (3g)

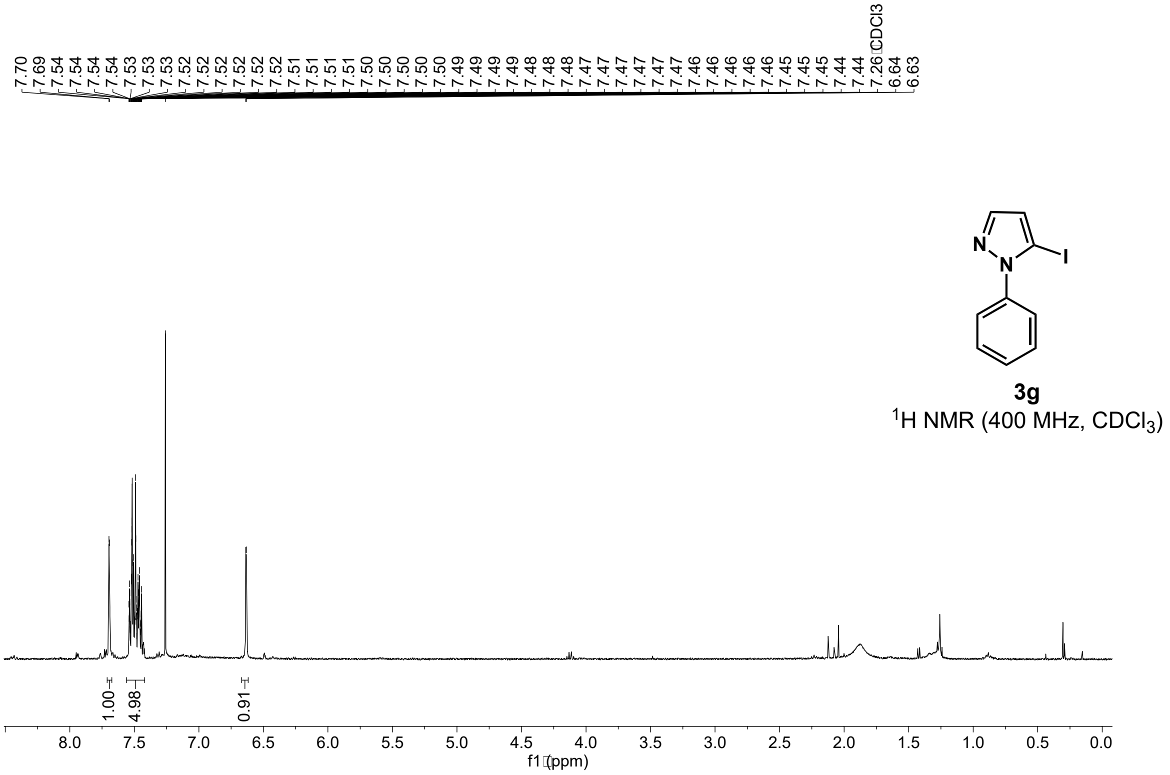

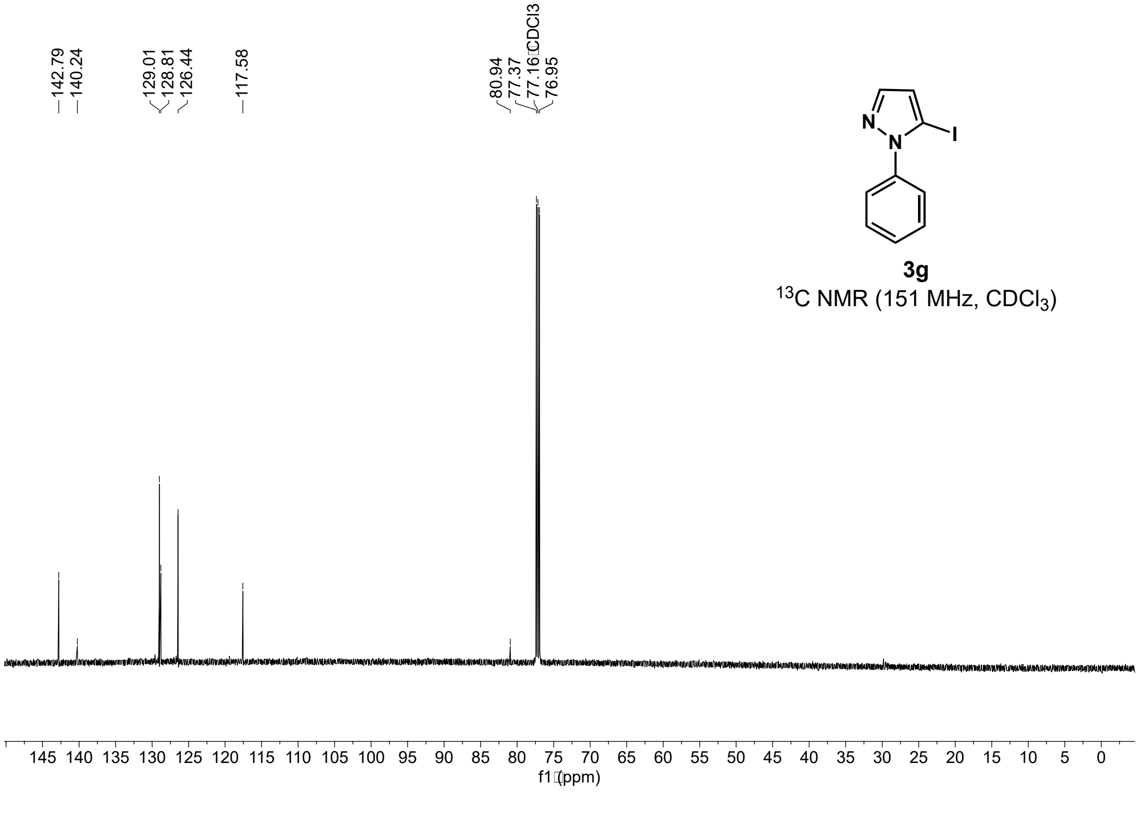

1-Phenyl-5-(o-tolyl)-1*H*-pyrazole (3h)

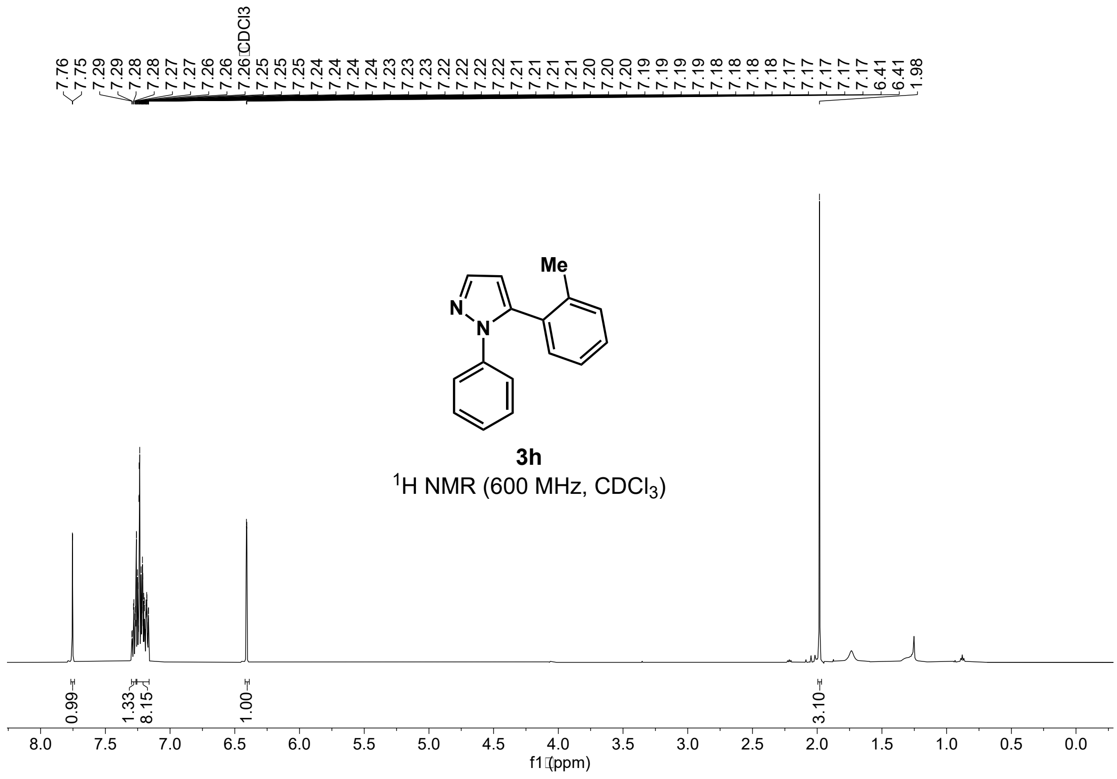

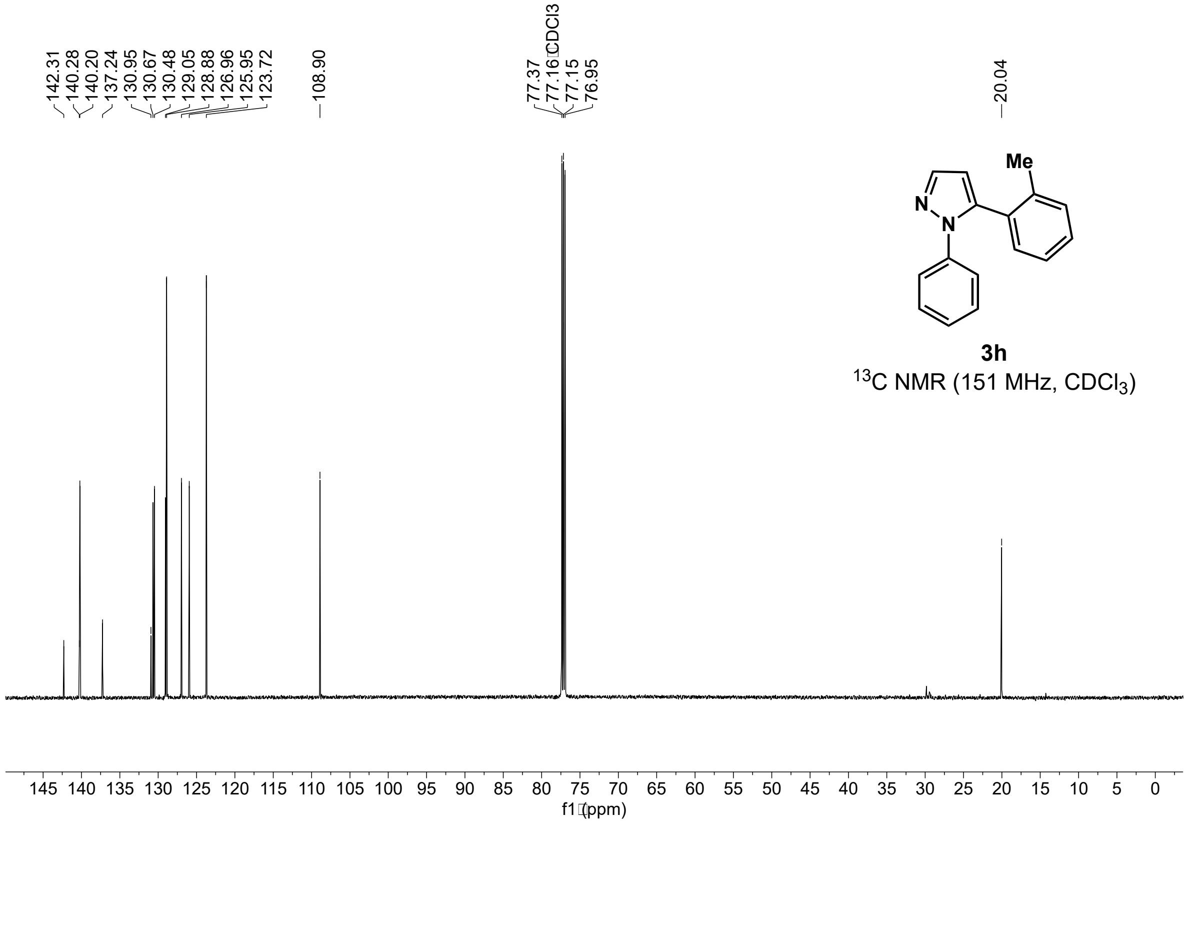

5-(2-Fluorophenyl)-1-phenyl-1*H*-pyrazole (3i)

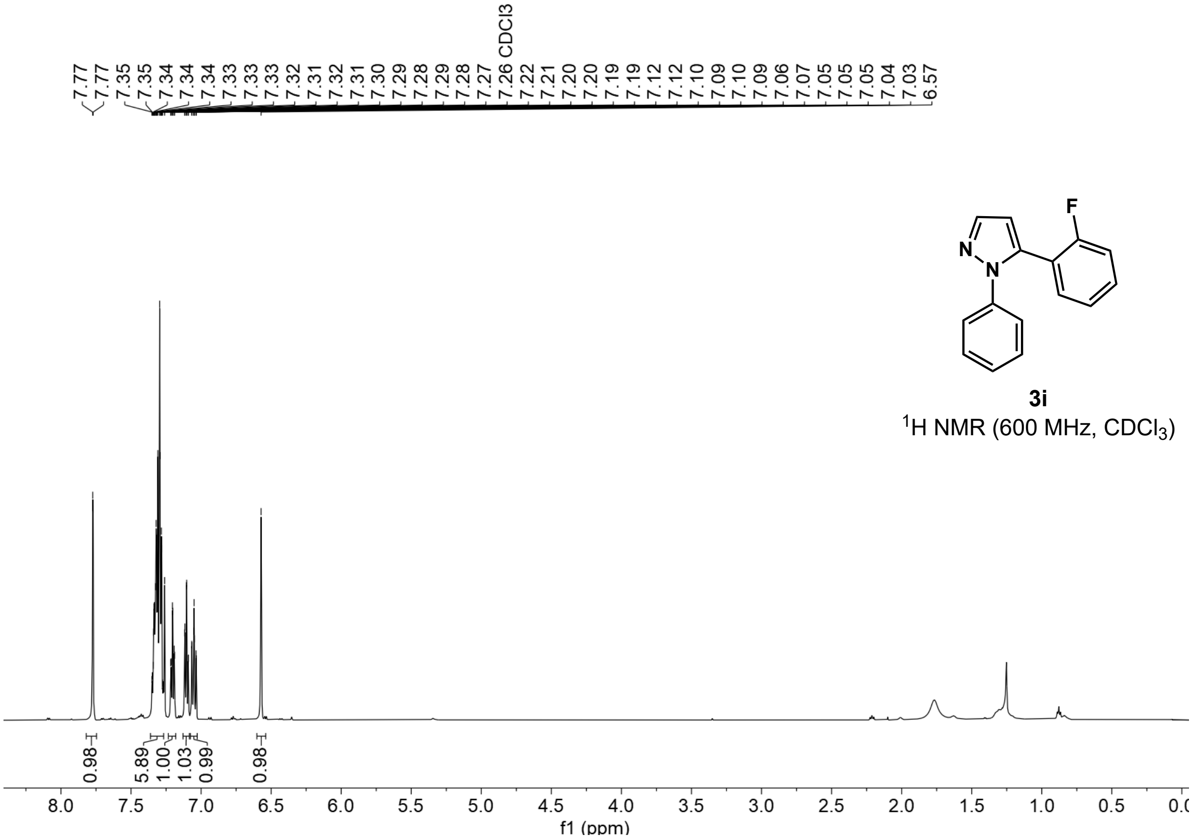

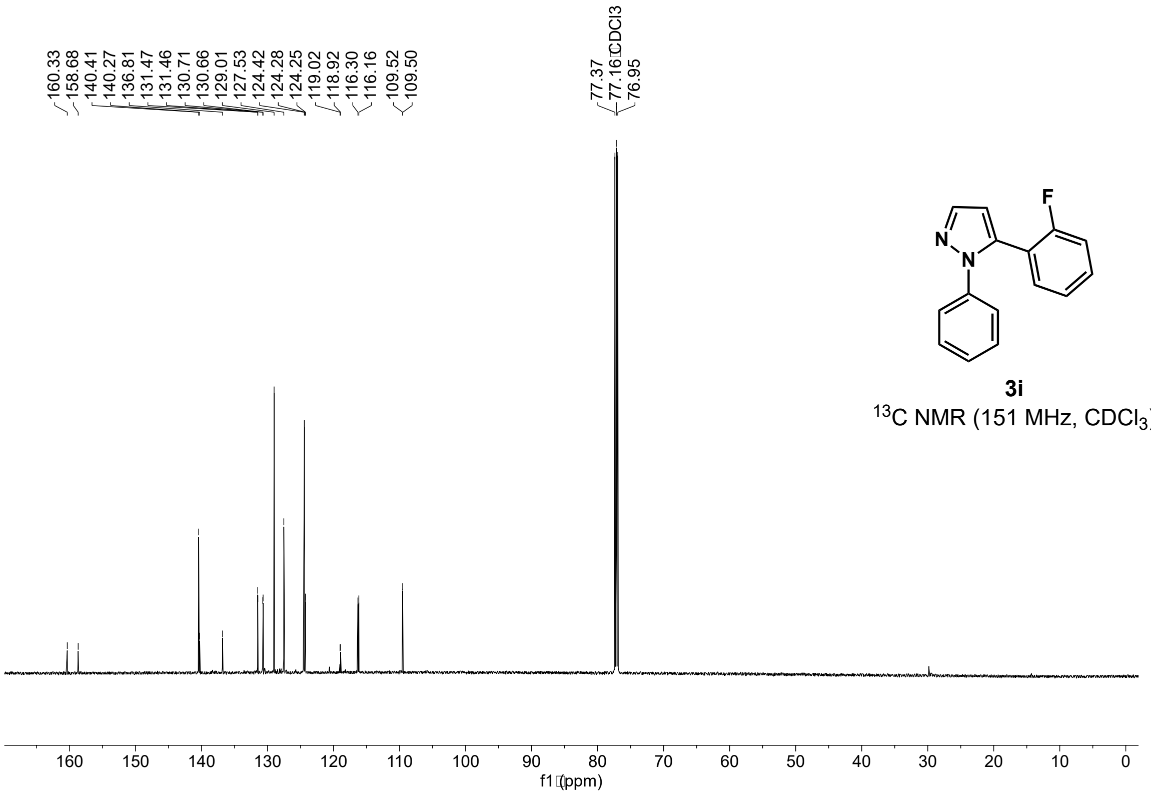

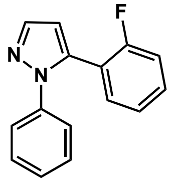

3i

^{19}F NMR (565 MHz, $CDCl_3$)

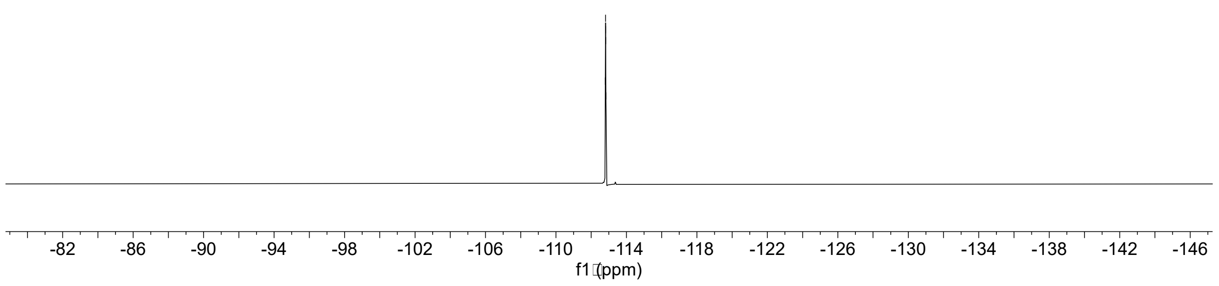

5-(2-Bromophenyl)-1-phenyl-1*H*-pyrazole (3j)

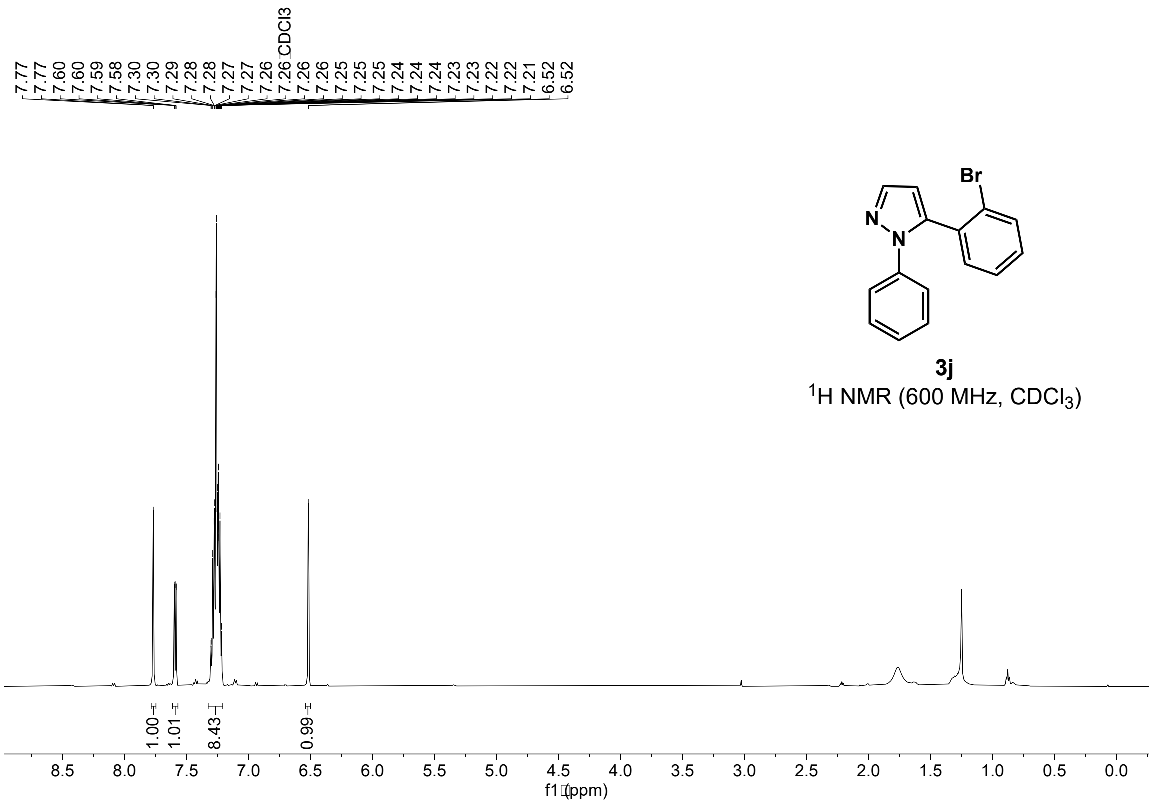

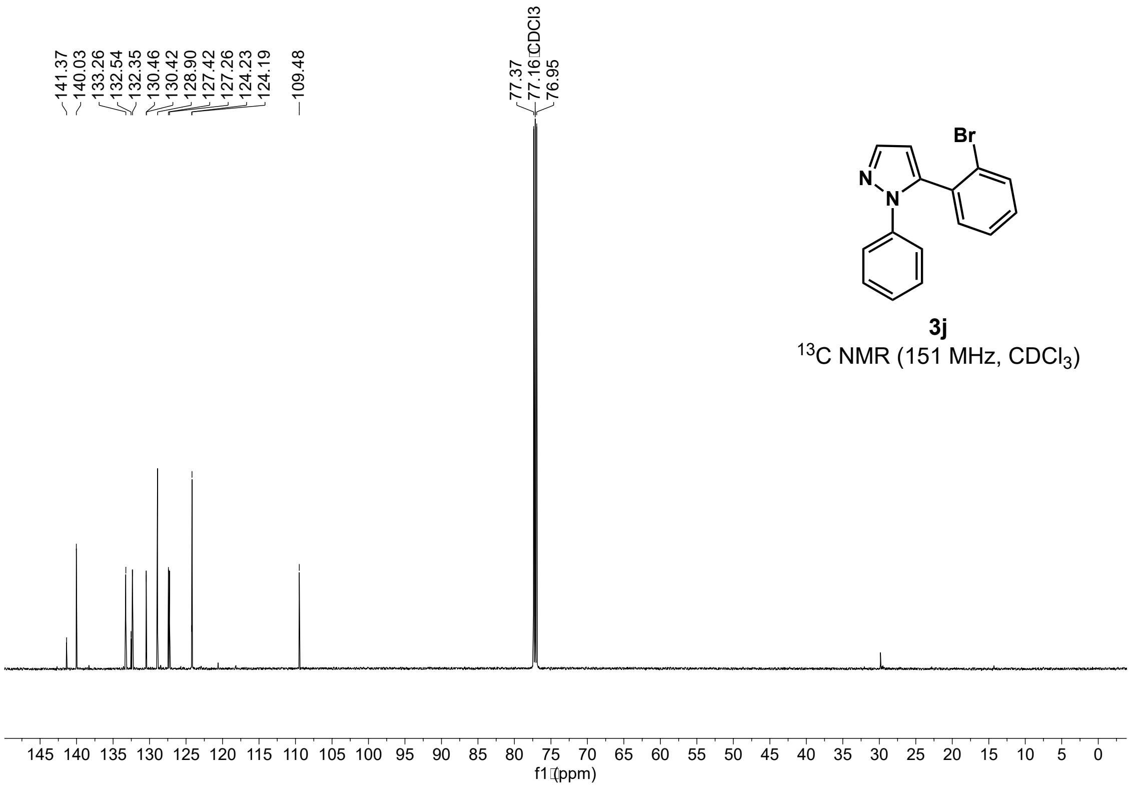

5-(2-Methoxyphenyl)-1-phenyl-1*H*-pyrazole (3k)

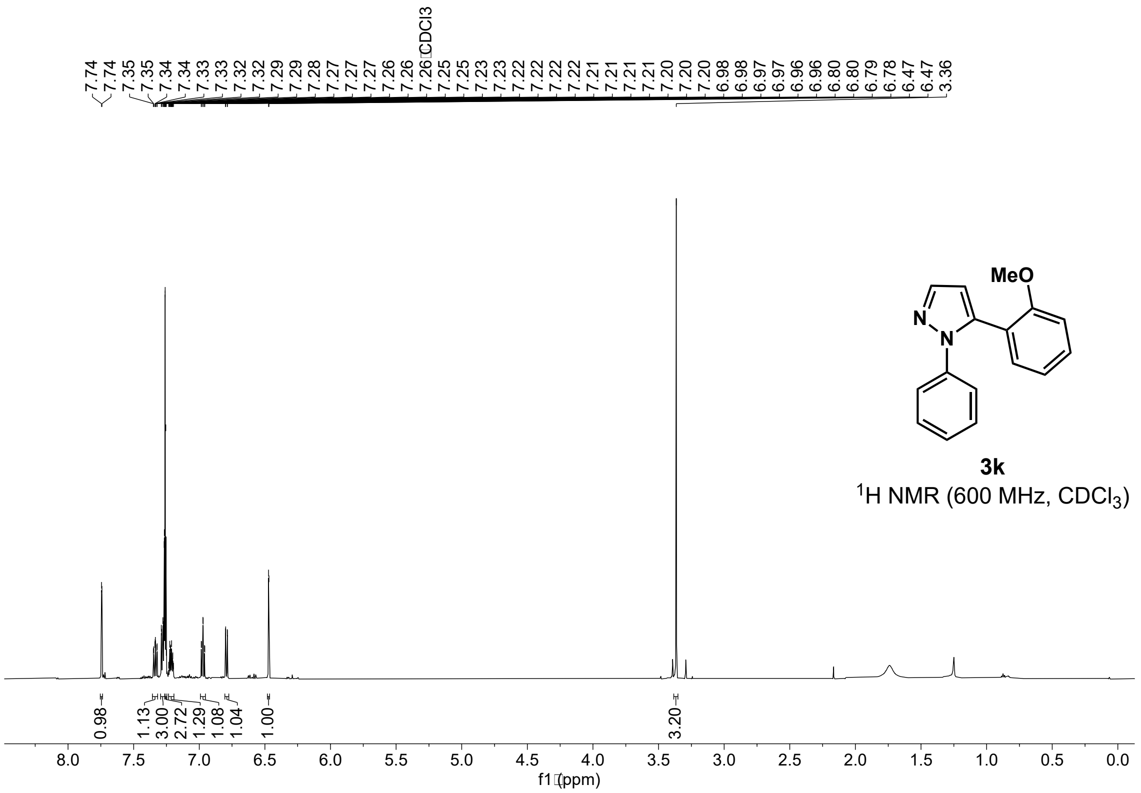

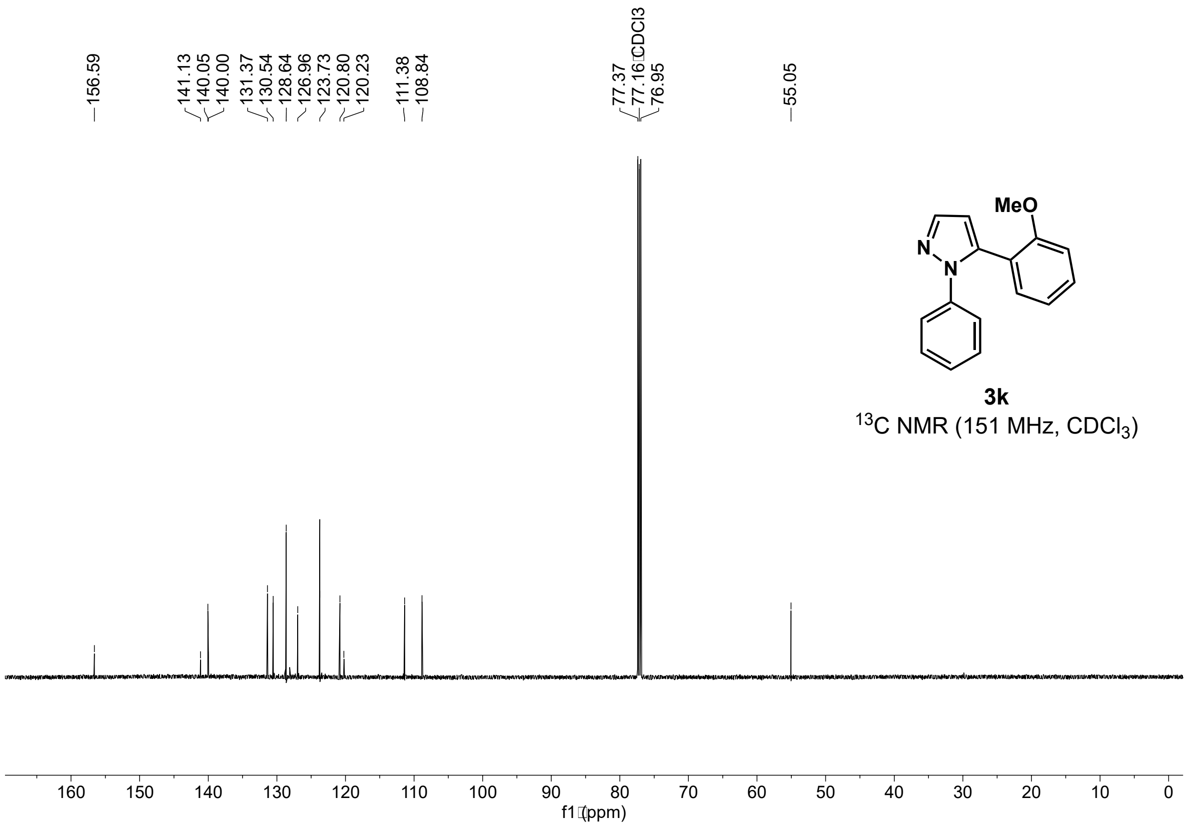

1-Phenyl-5-(p-tolyl)-1*H*-pyrazole (3l)

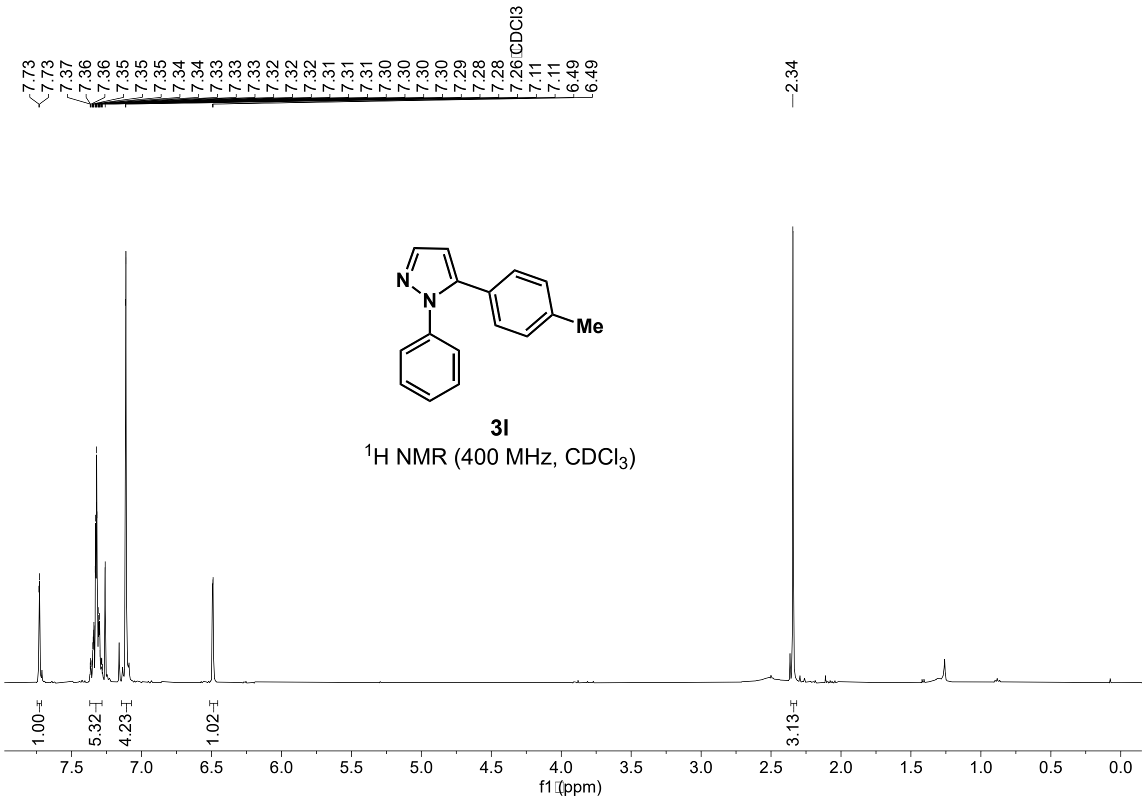

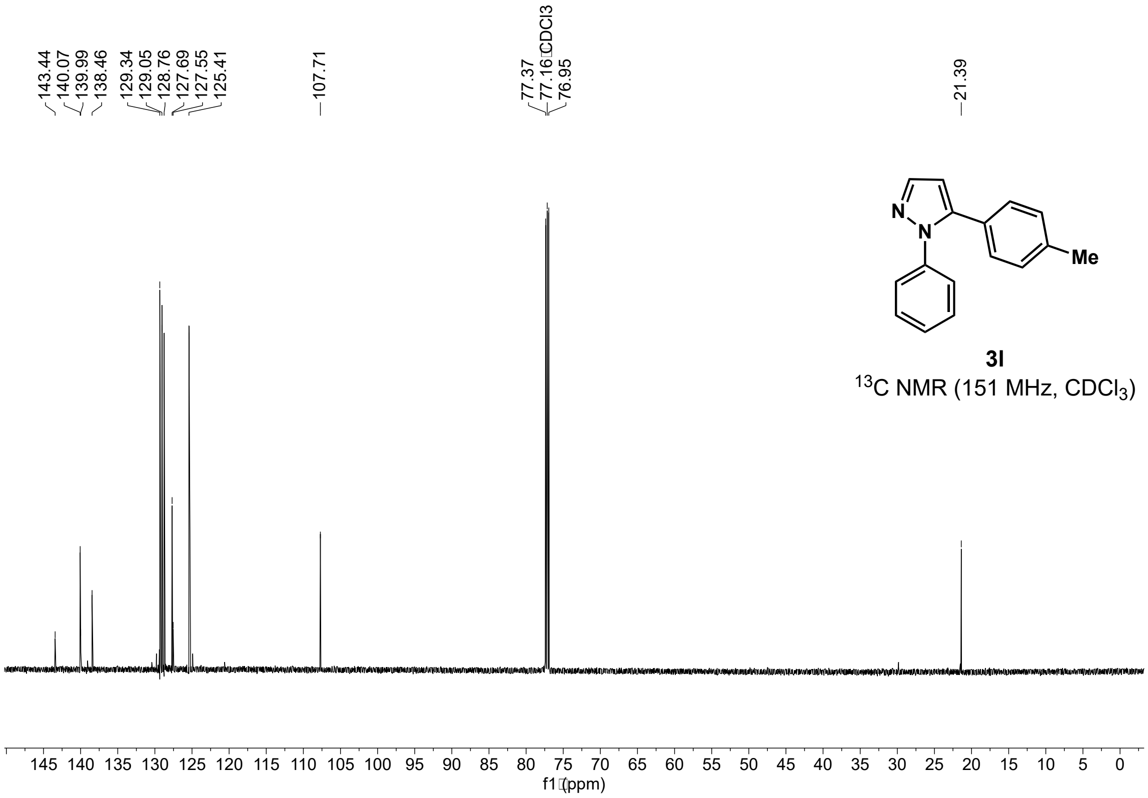

5-(4-Chlorophenyl)-1-phenyl-1*H*-pyrazole (3m)

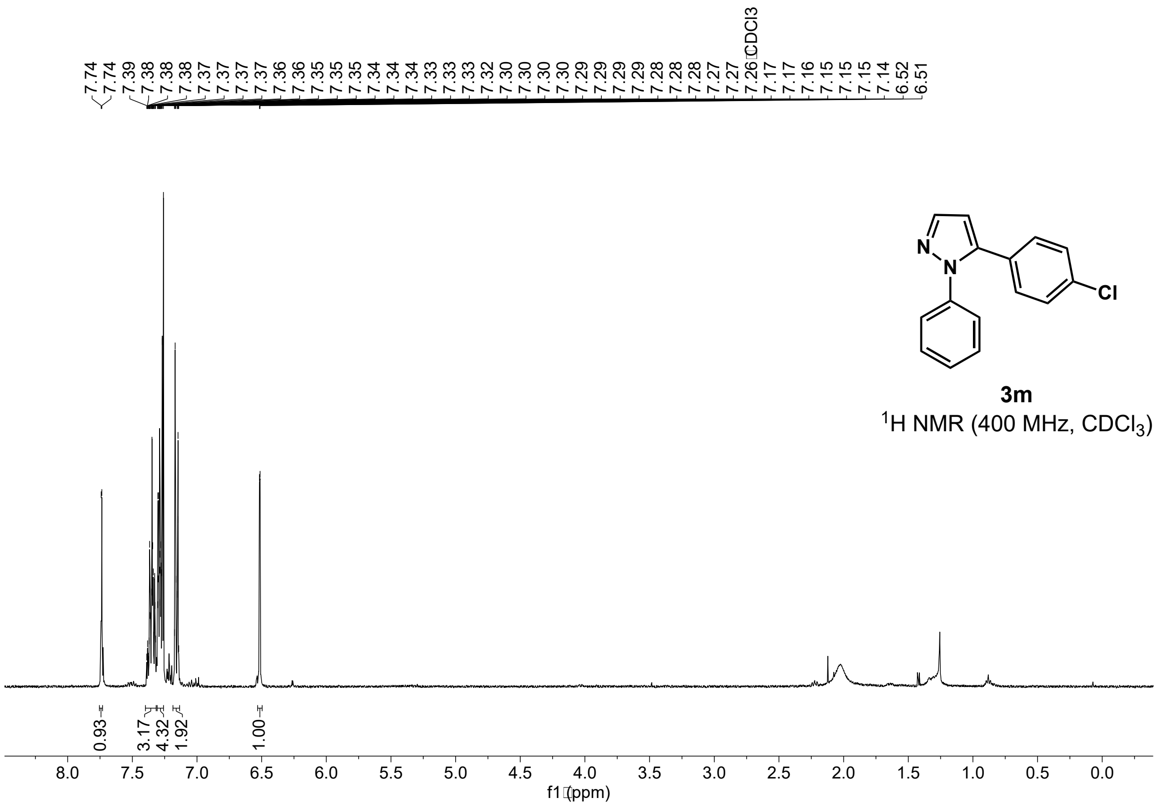

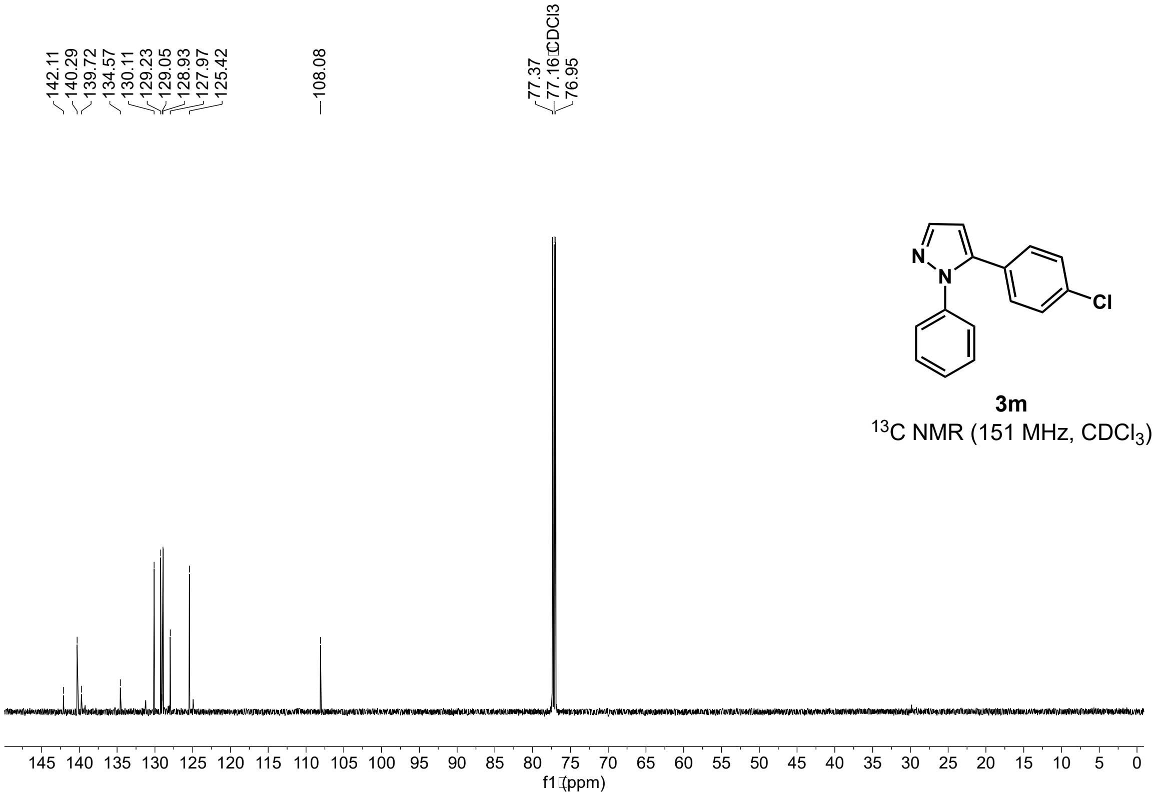

5-(4-Fluorophenyl)-1-phenyl-1*H*-pyrazole (3n)

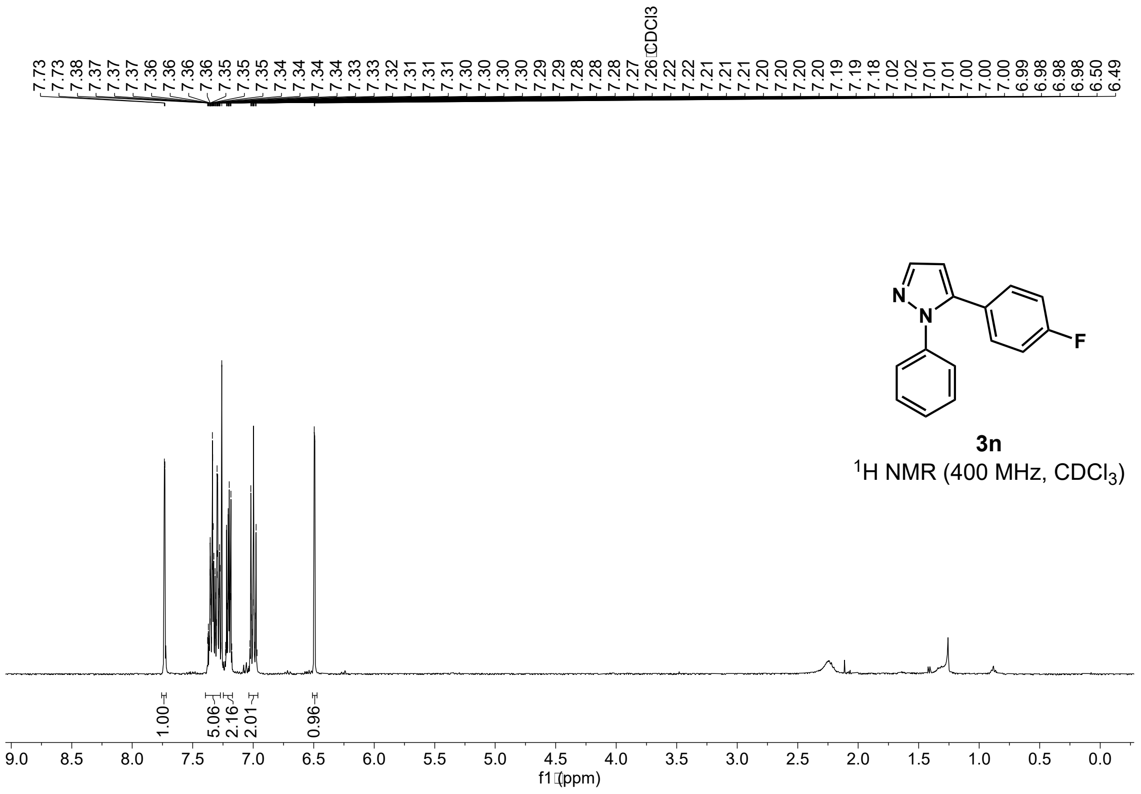

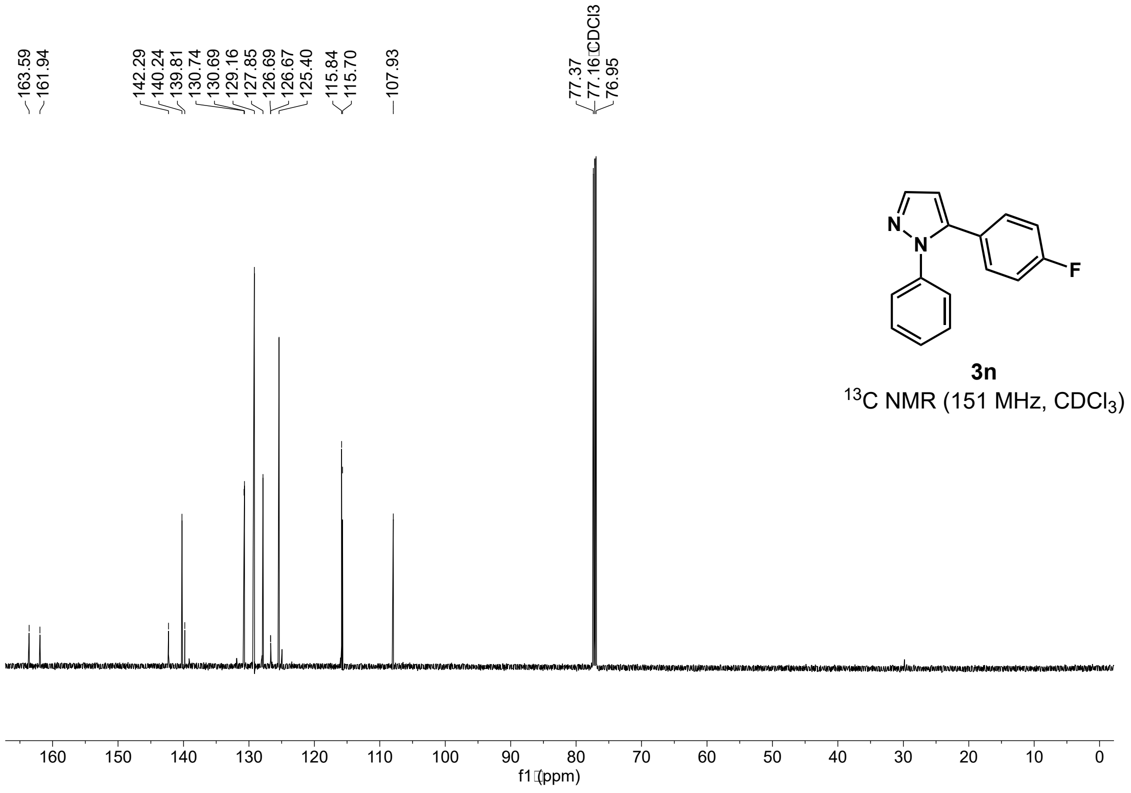

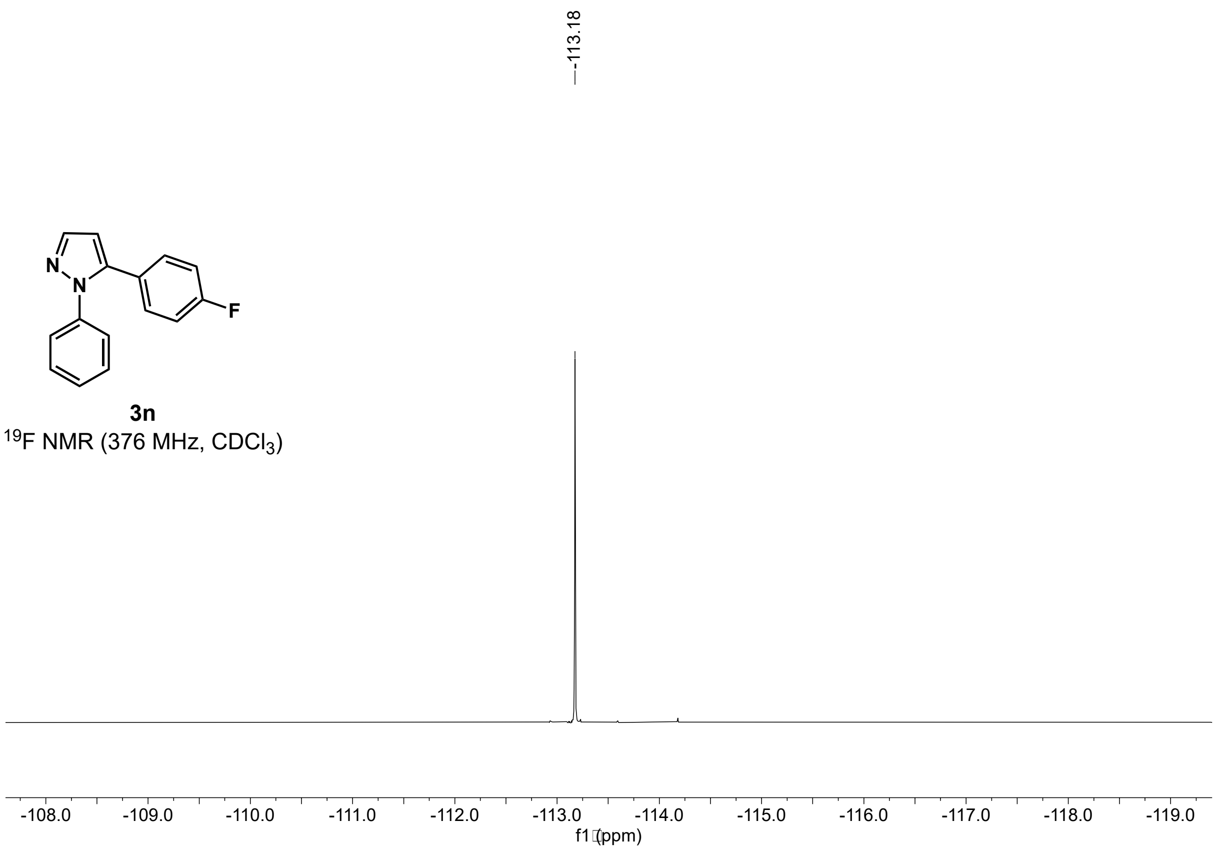

3n

^{19}F NMR (376 MHz, $CDCl_3$)

5-(3-Methoxyphenyl)-1-phenyl-1*H*-pyrazole (3o)

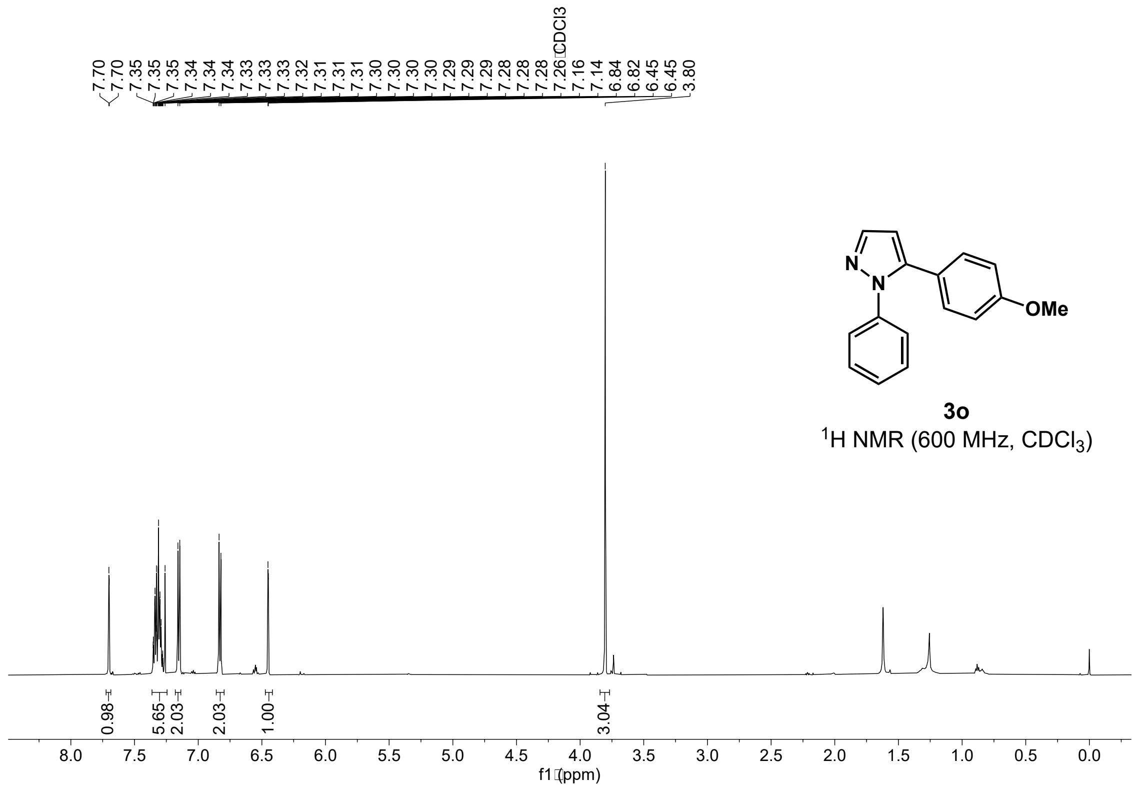

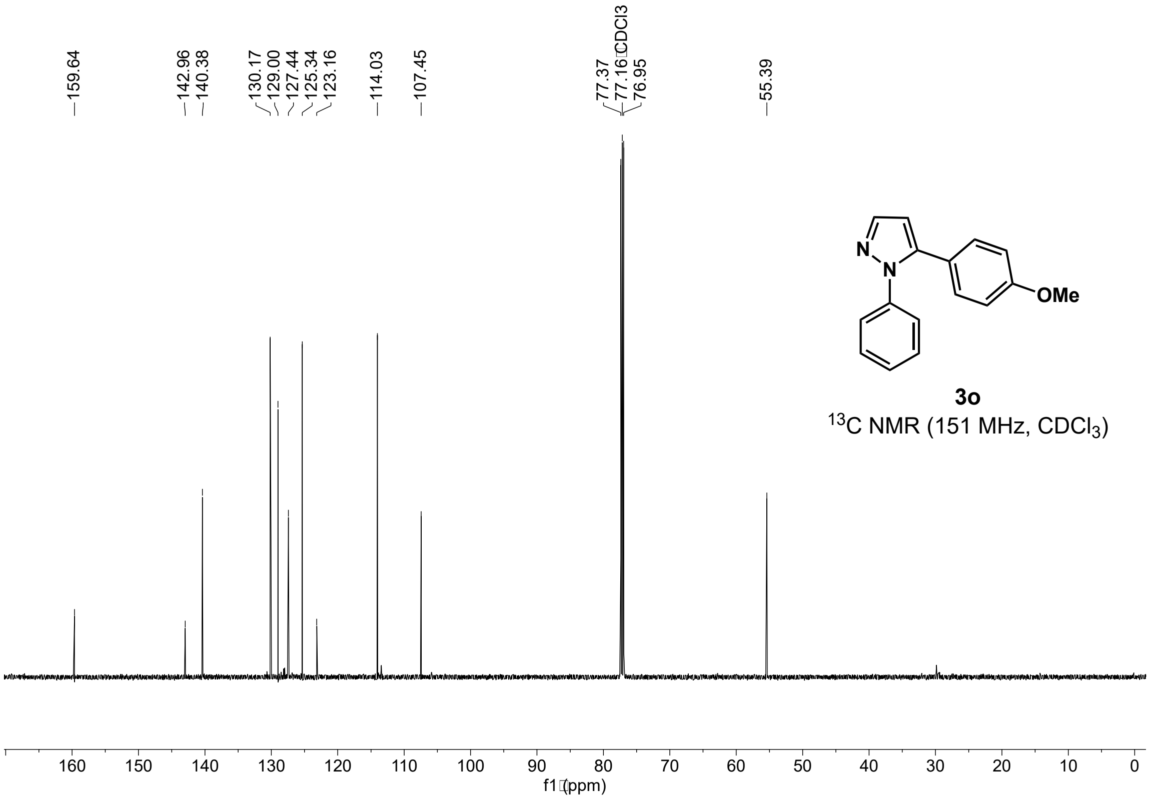

5-(4-Methoxyphenyl)-1-phenyl-1*H*-pyrazole (3p)

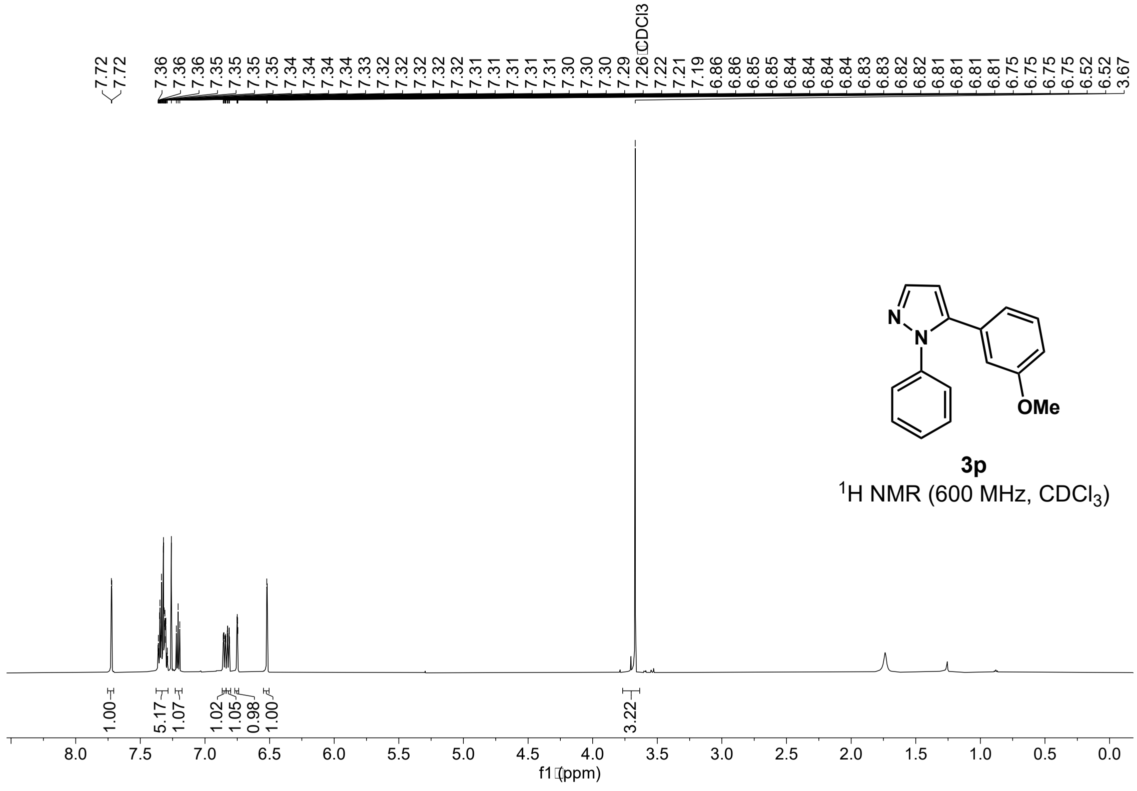

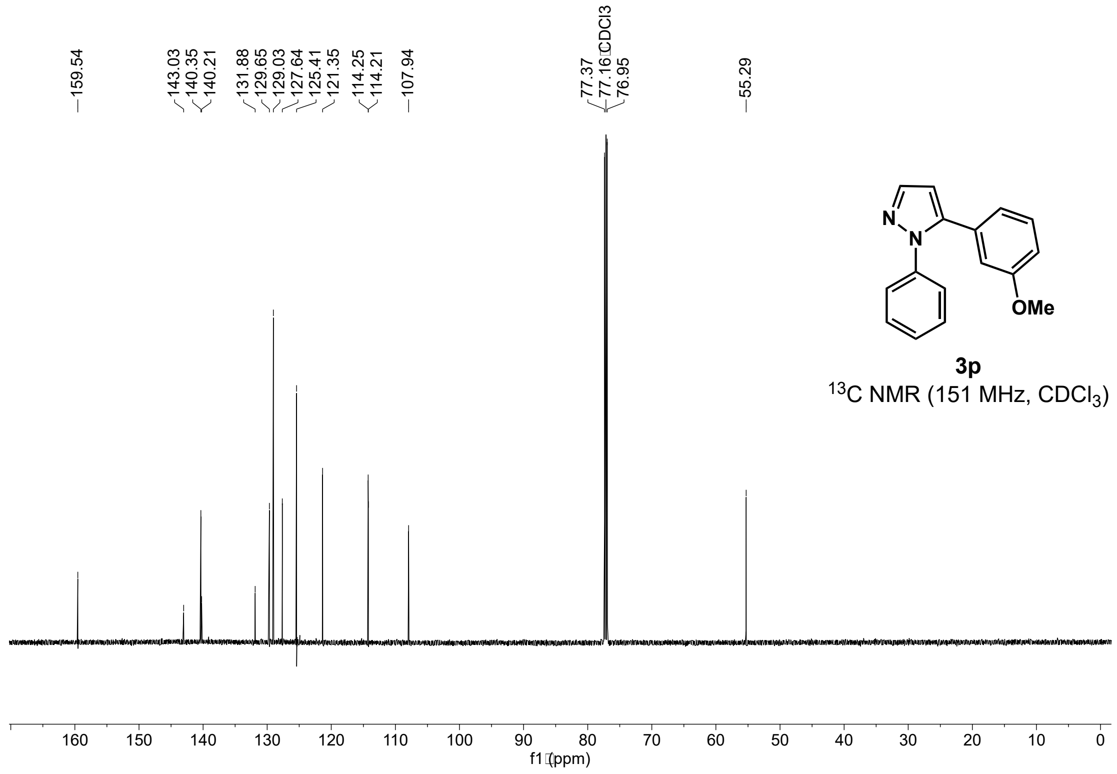

5-(Furan-2-yl)-1-phenyl-1*H*-pyrazole (3q)

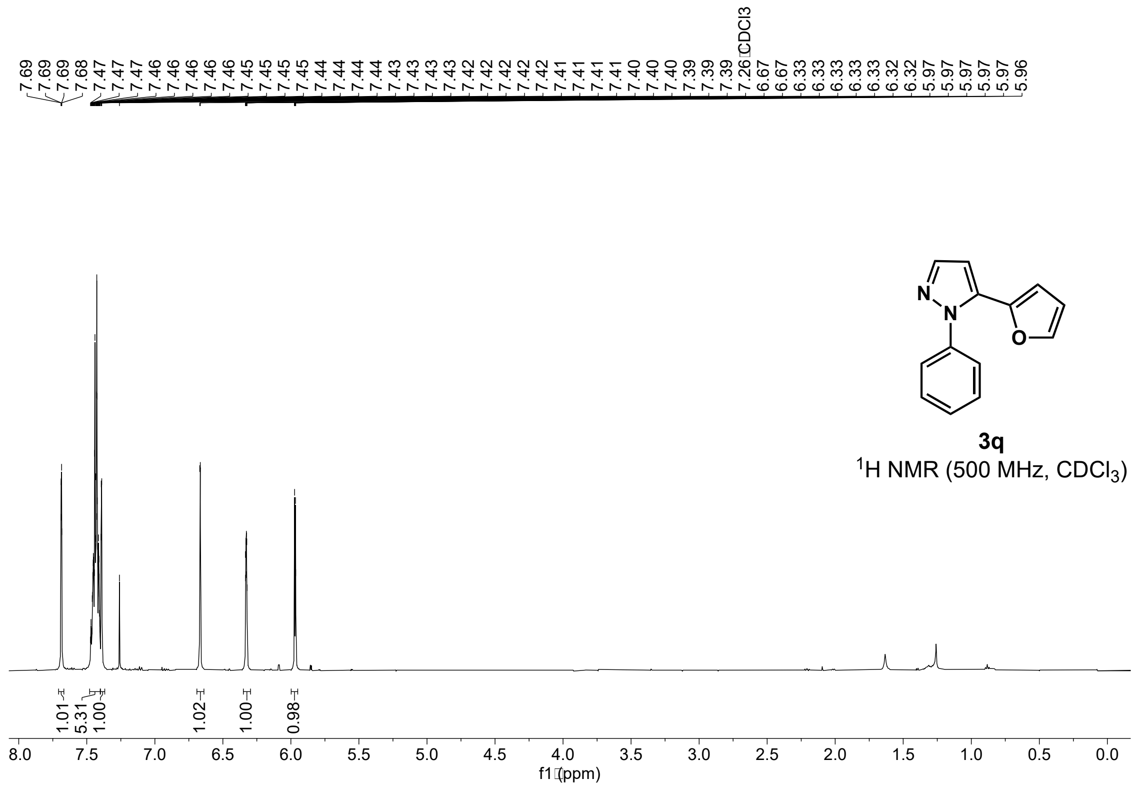

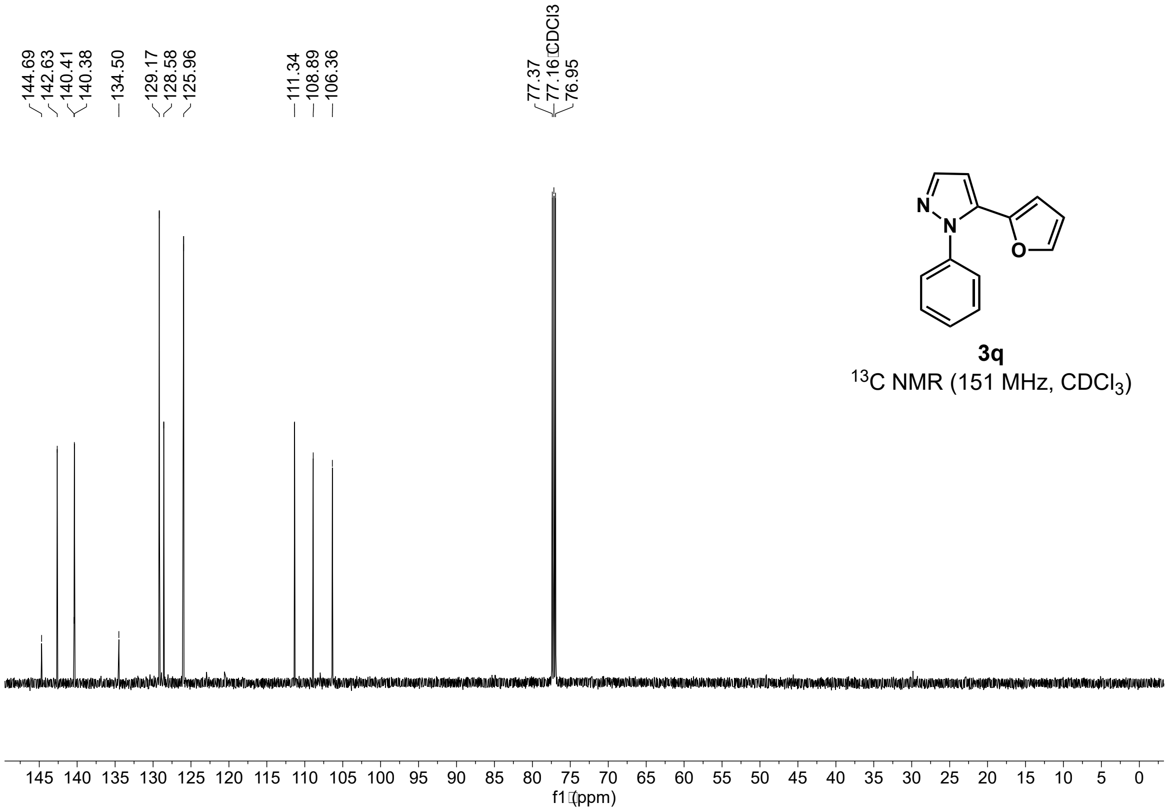

4-(1-Phenyl-1*H*-pyrazol-5-yl)pyridine (3r)

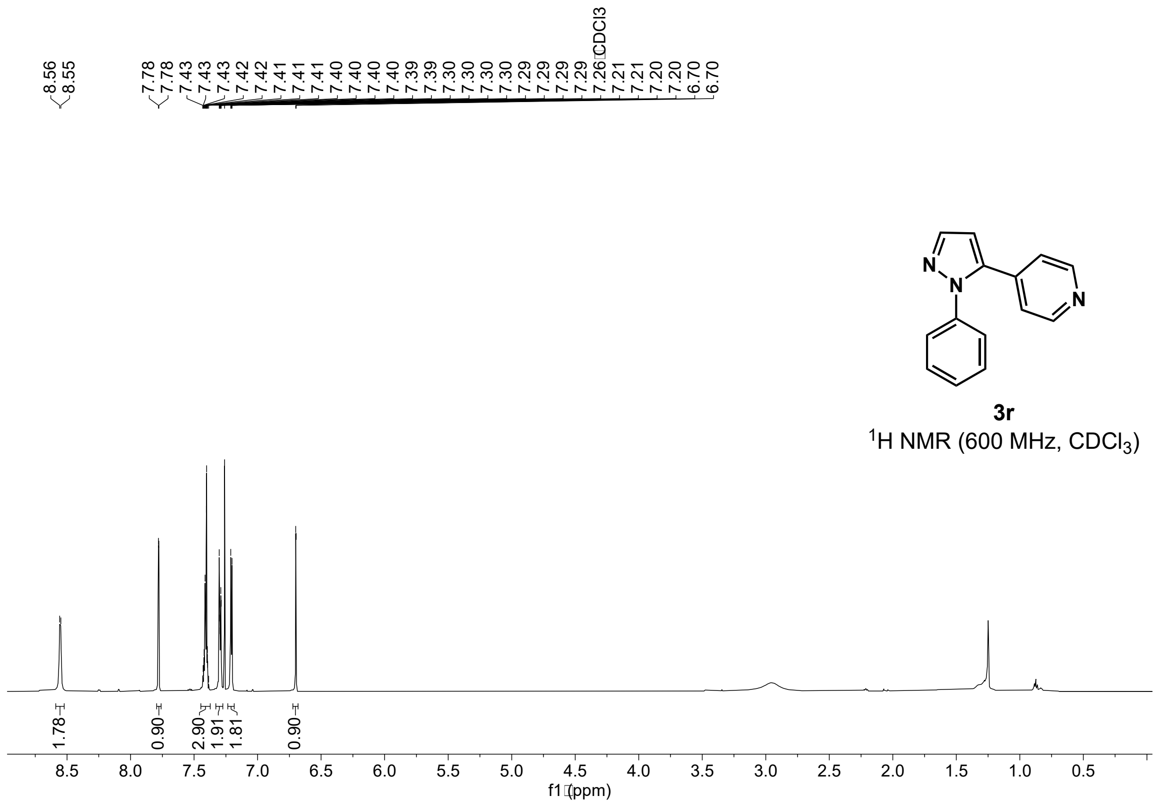

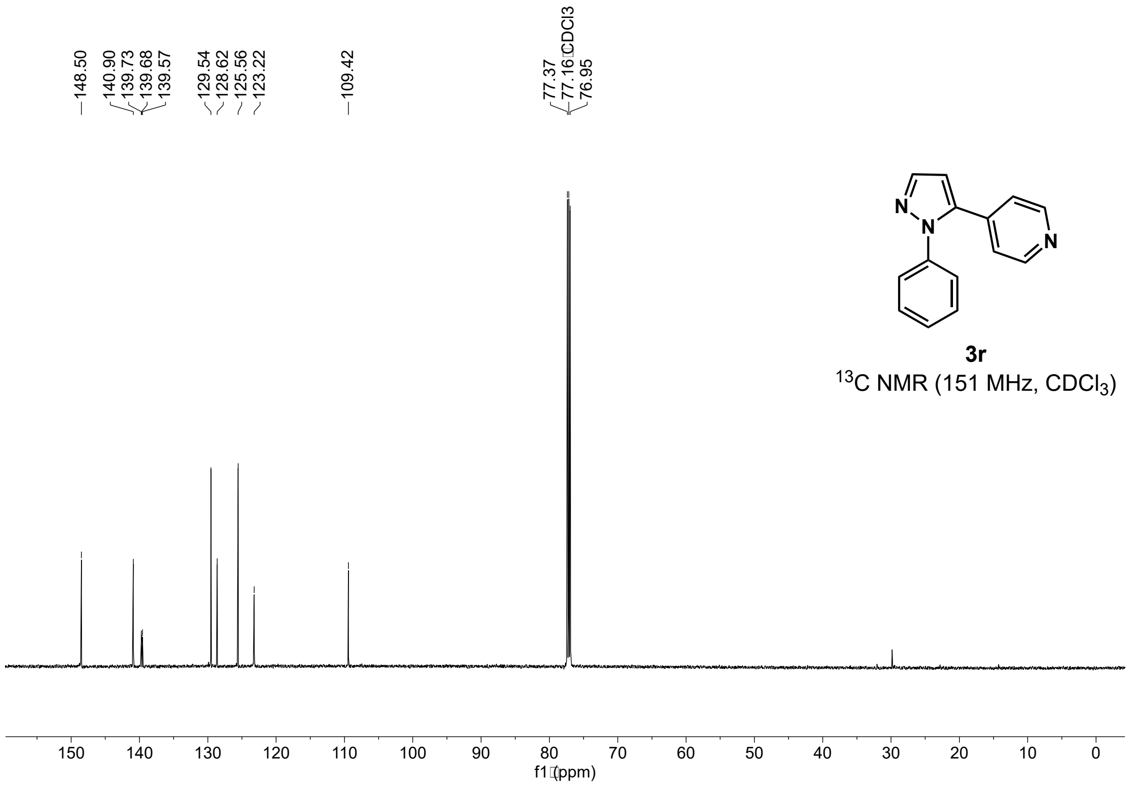

3-Methyl-1,5-diphenyl-1*H*-pyrazole (3s)

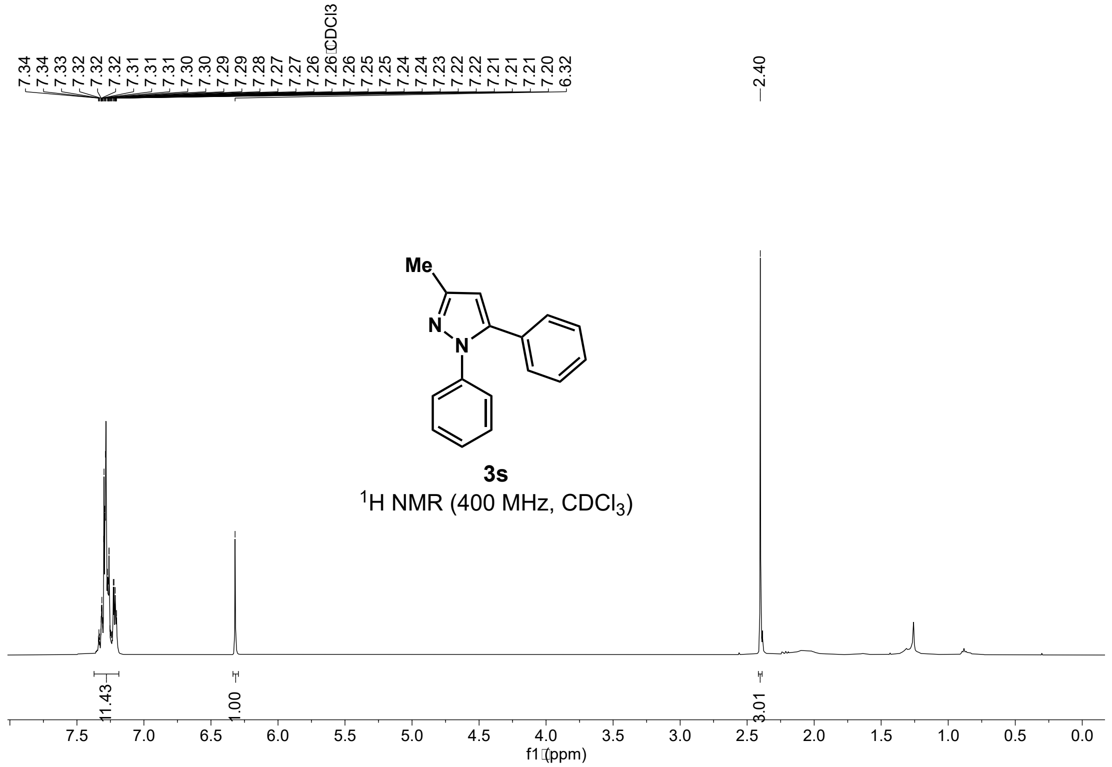

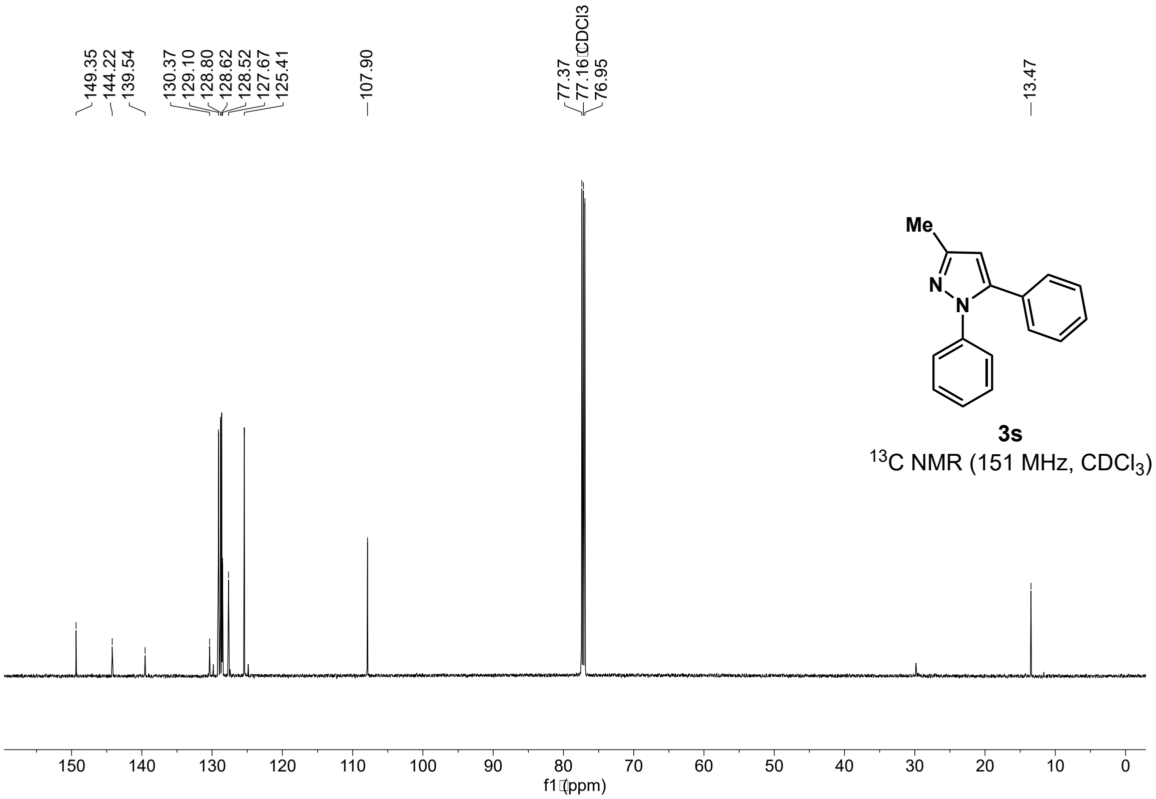

5-(Tert-butyl)-3-methyl-1-phenyl-1*H*-pyrazole (3t)

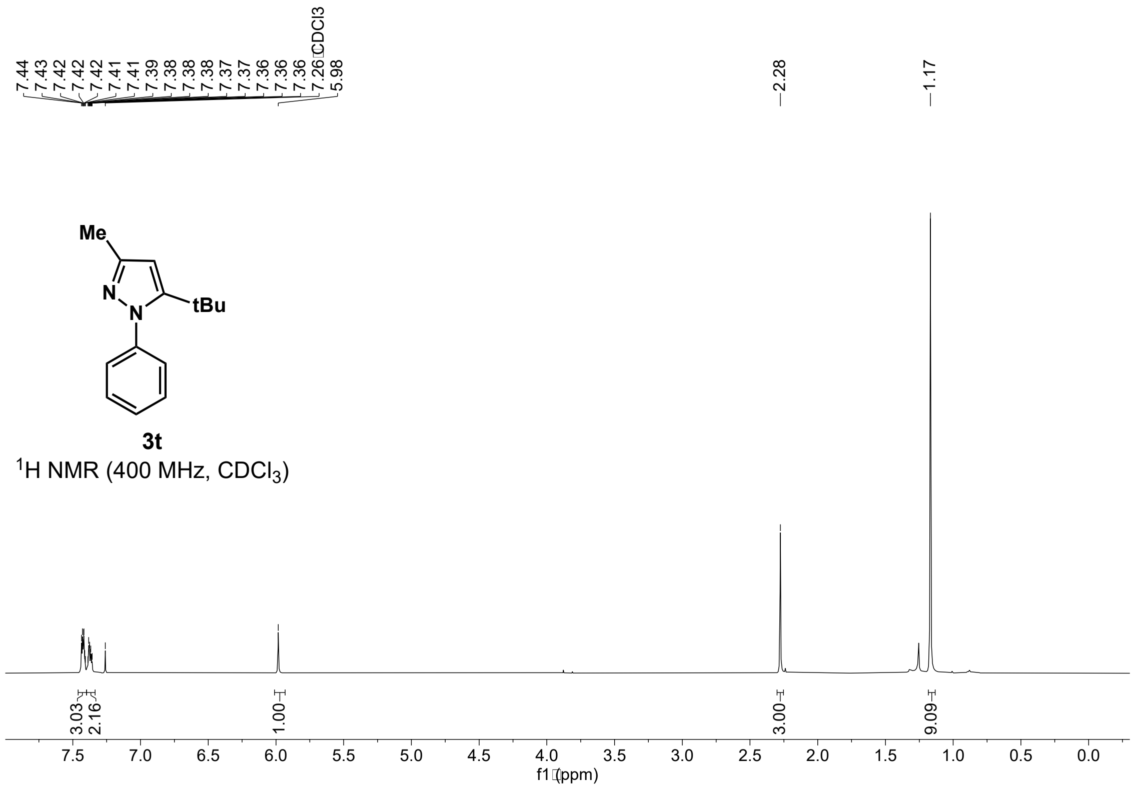

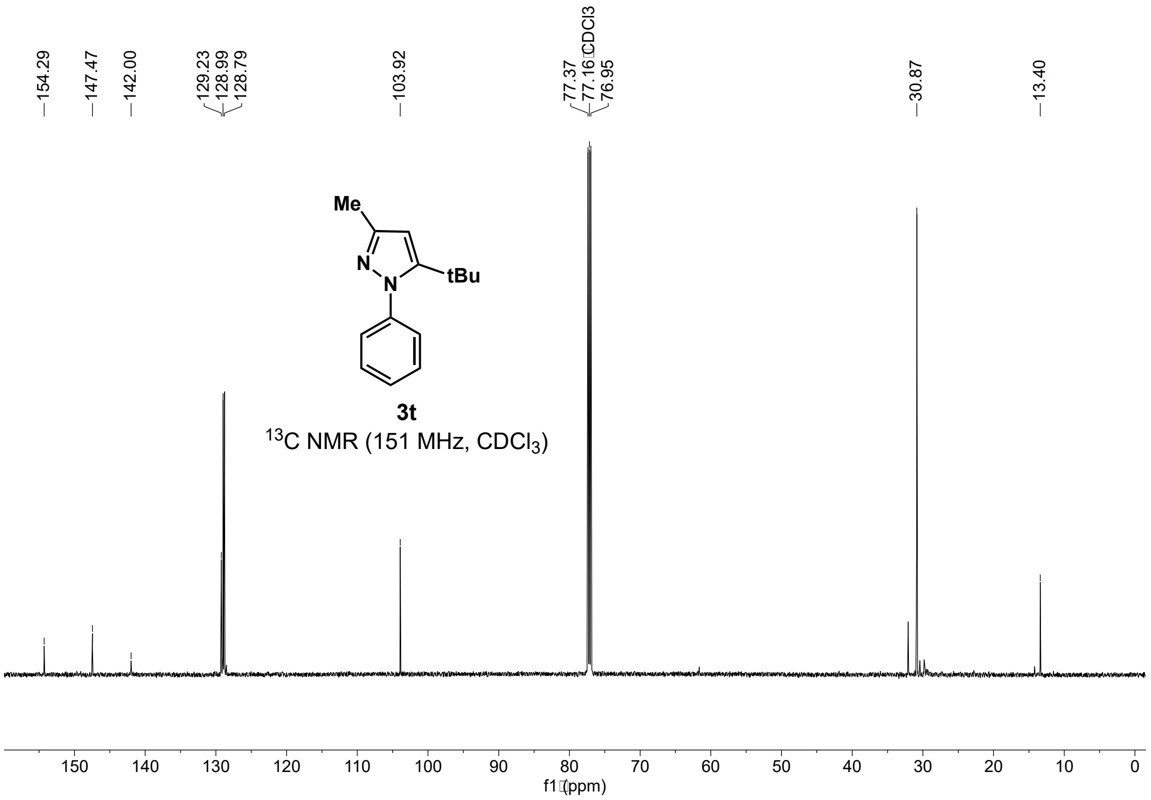

5-(Tert-butyl)-3-iodo-1-phenyl-1*H*-pyrazole (3u)

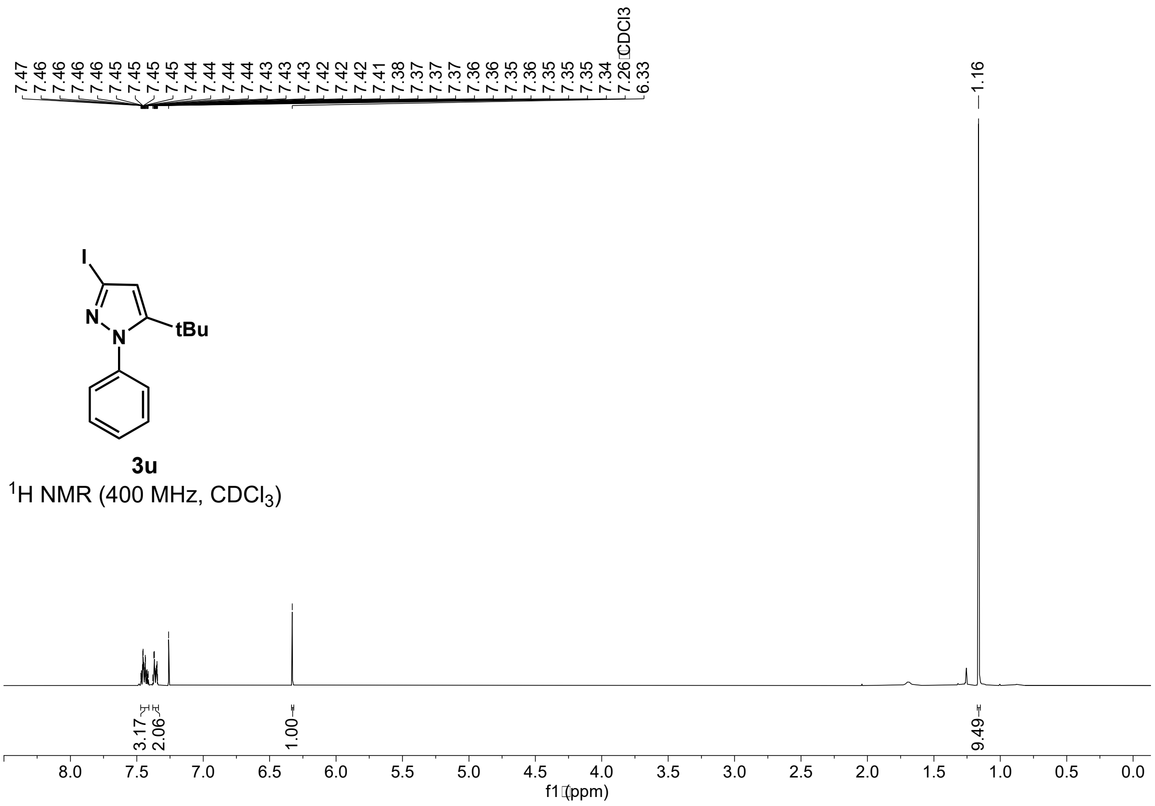

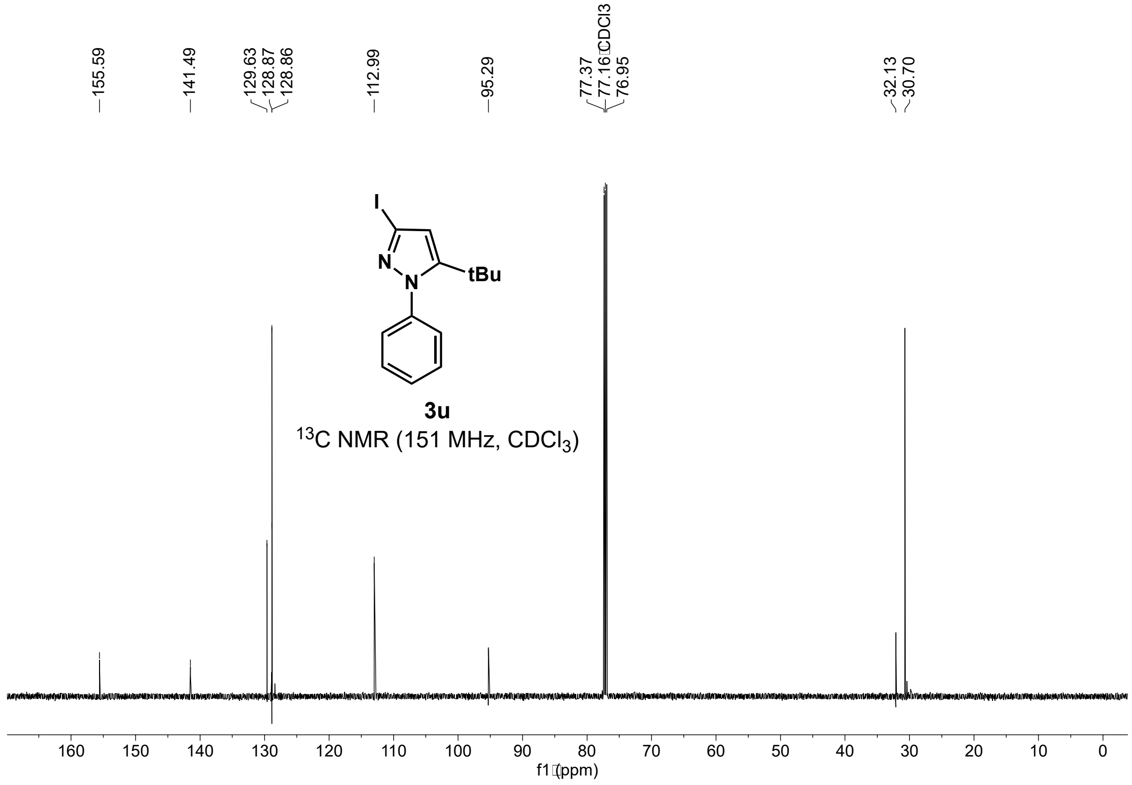

1,5-Diphenyl-1*H*-pyrazol-3-amine (3v)

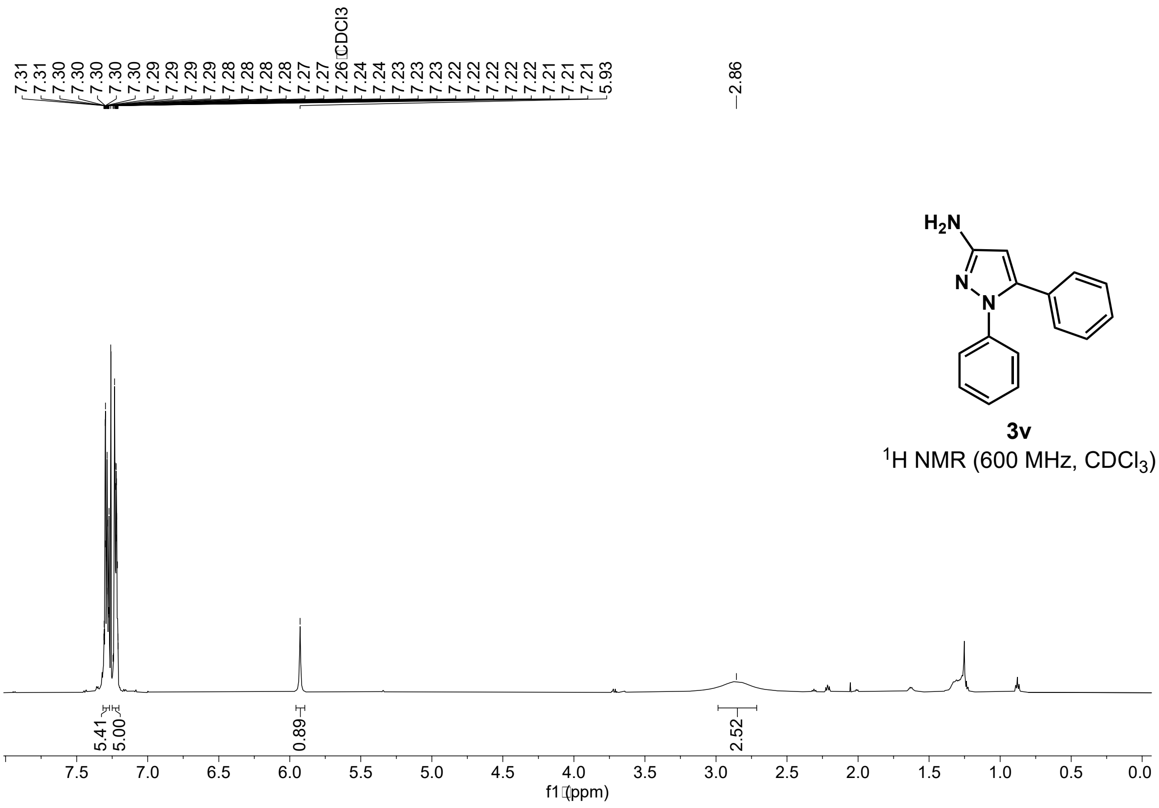

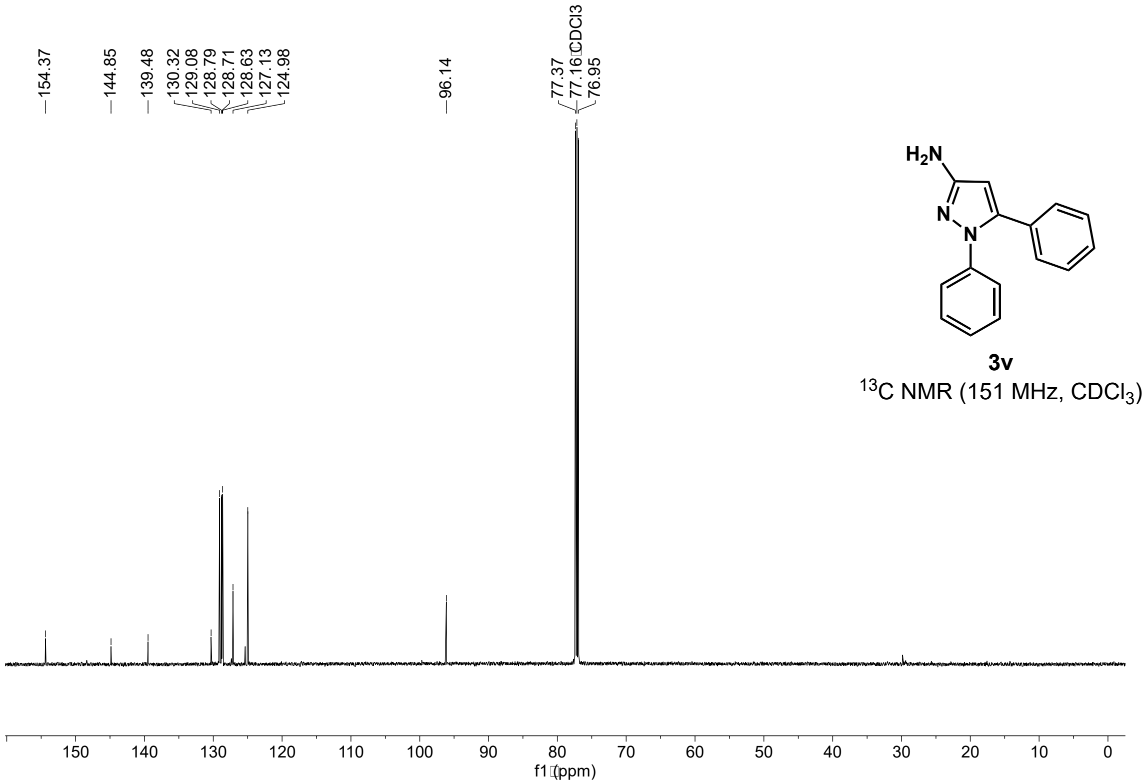

3-Cyclopropyl-1-phenyl-5-(trifluoromethyl)-1*H*-pyrazole (3w)

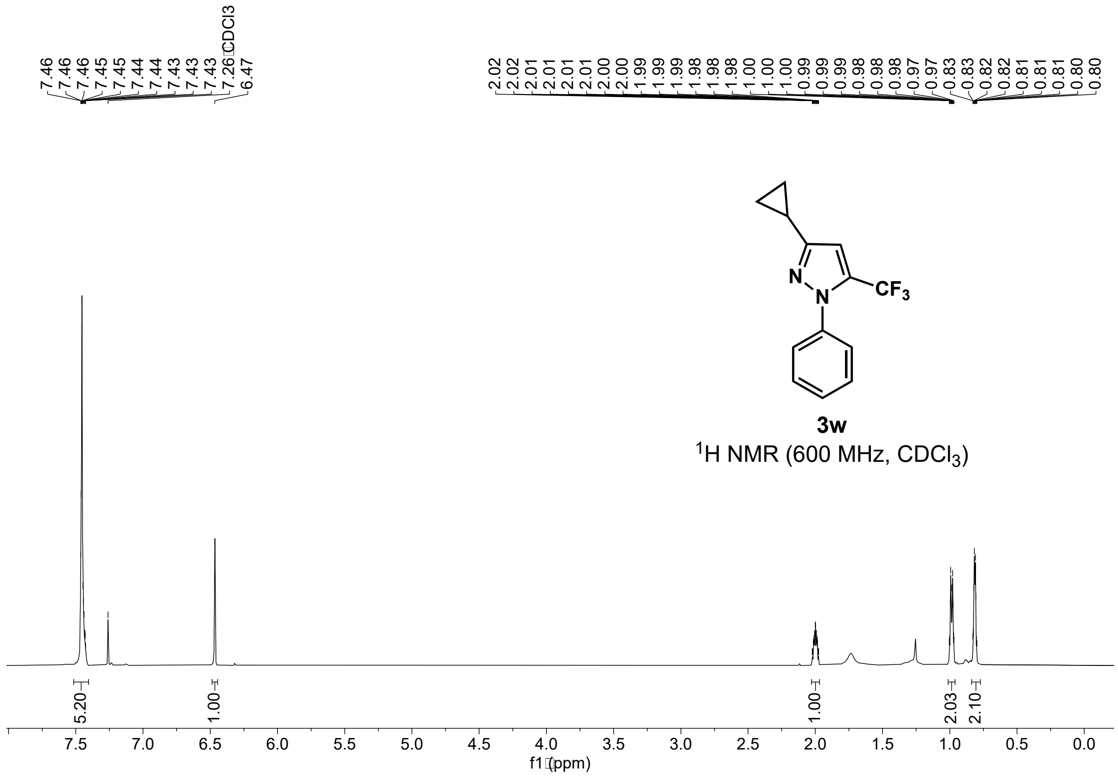

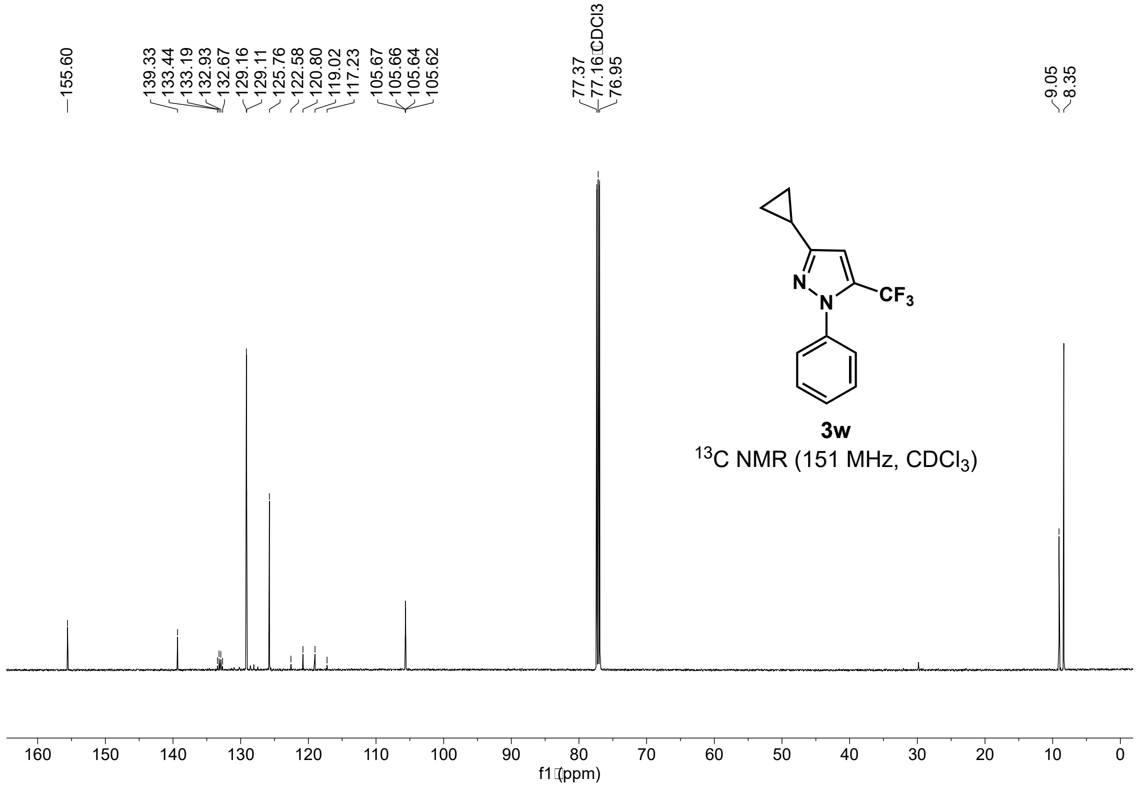

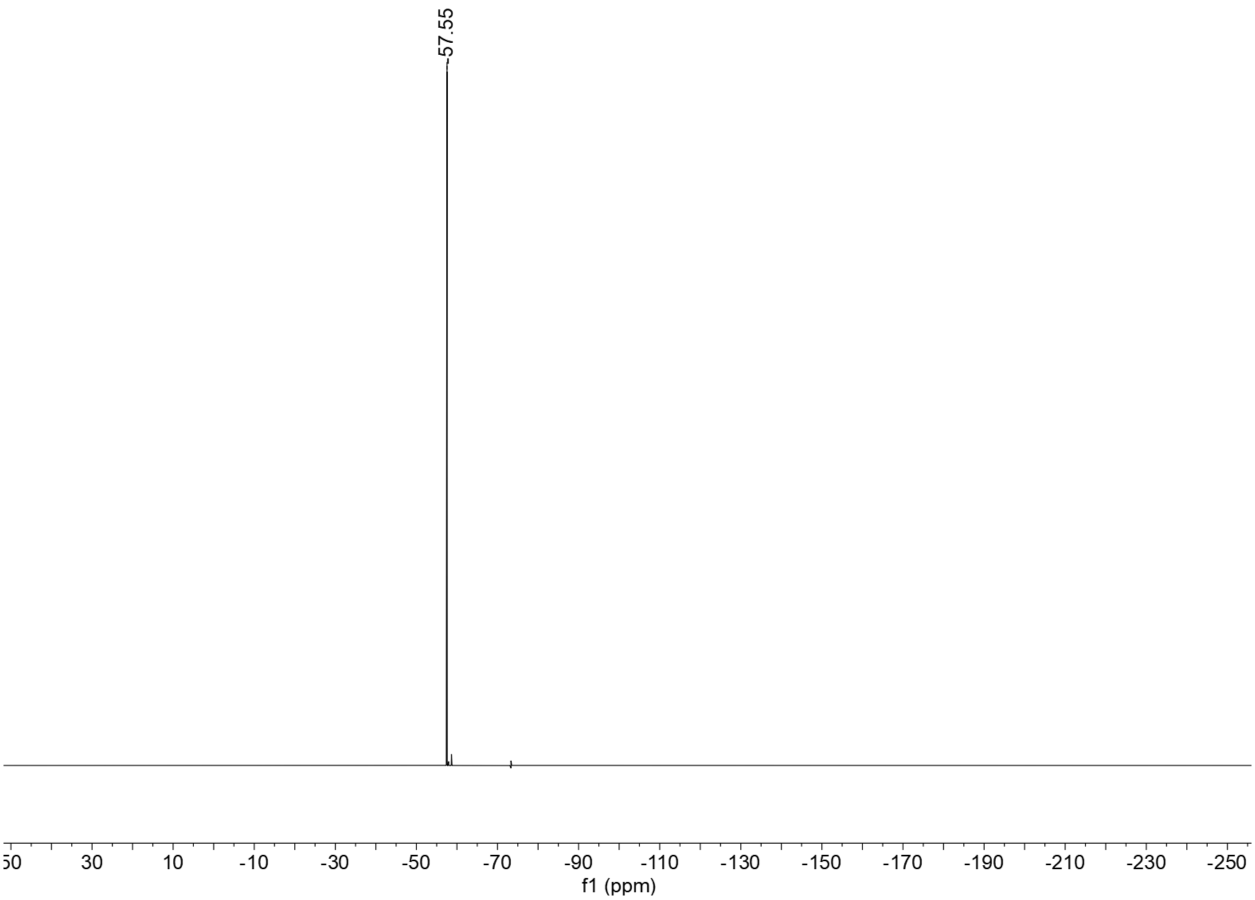
-57.55
50
30
10
-10
-30
-50
-70
-90
-110
-130
-150
-170
-190
-210
-230
-250
f1 (ppm)

Methyl 5-ethyl-1-phenyl-1*H*-pyrazole-3-carboxylate (3x)

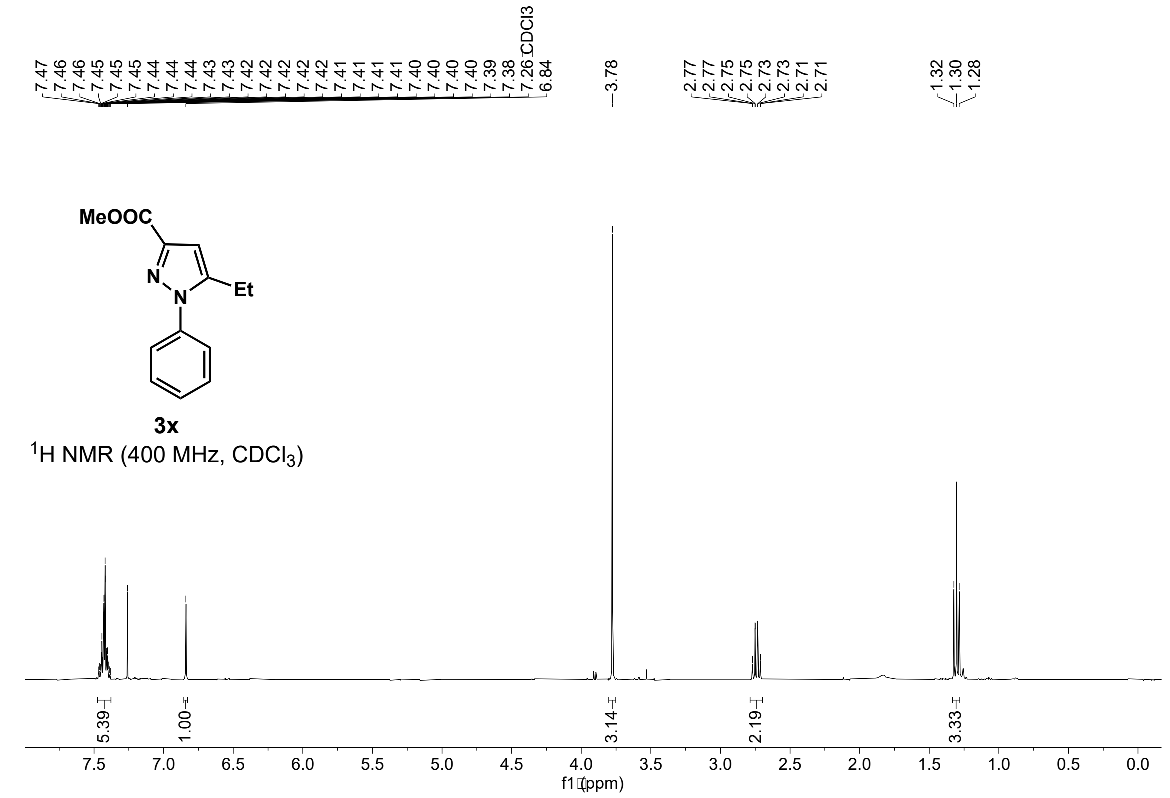

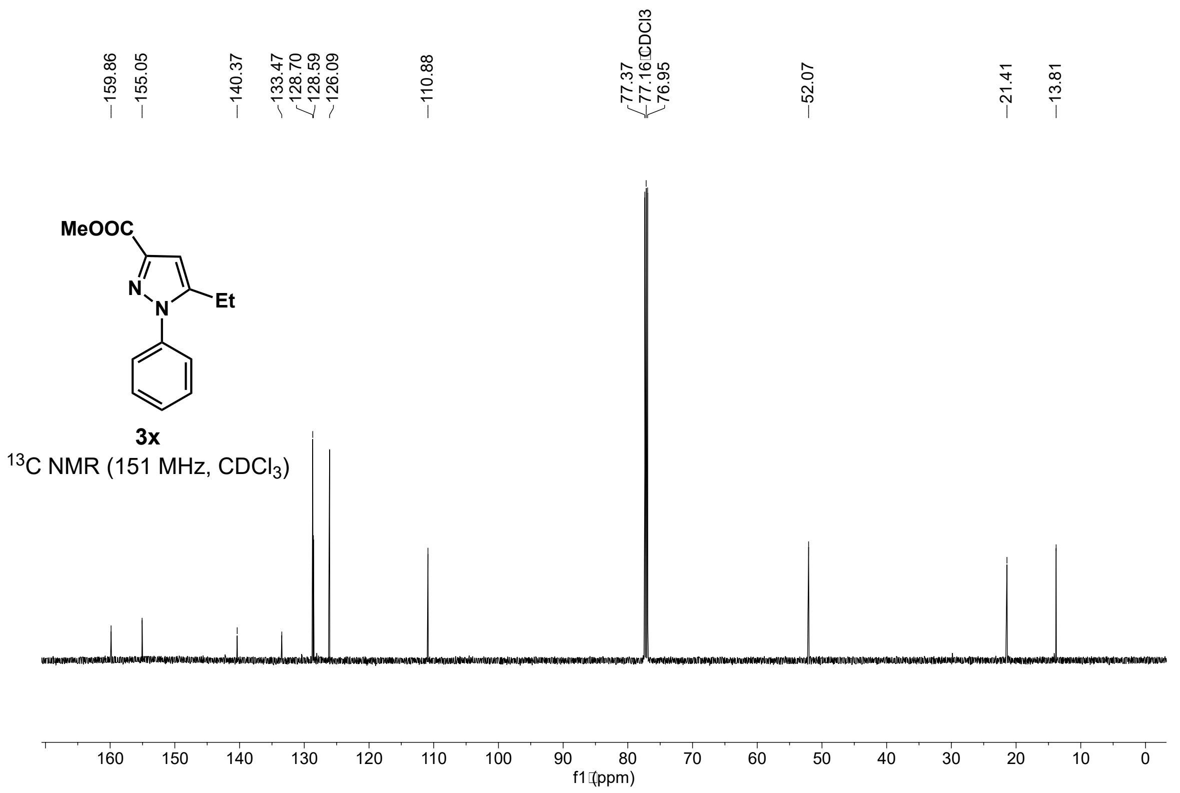

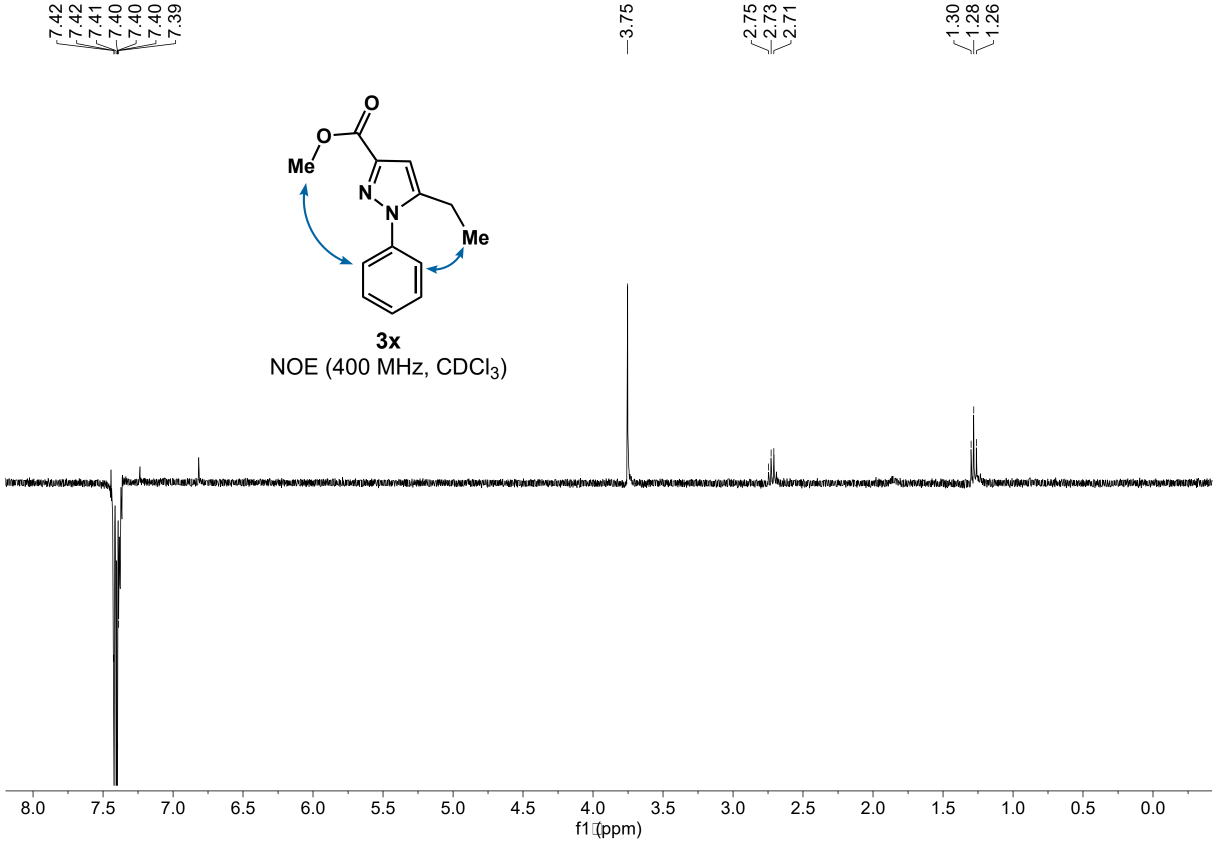
7.42
7.42
7.41
7.40
7.40
7.40
7.39
3.75
2.75
2.73
2.71
1.30
1.28
1.26
O
O
Me
N
N
Me
3x
NOE (400 MHz, CDCl3)
8.0
7.5
7.0
6.5
6.0
5.5
5.0
4.5
4.0
3.5
3.0
2.5
2.0
1.5
1.0
0.5
0.0
f1 (ppm)

Methyl 4-bromo-1-phenyl-1*H*-pyrazole-3-carboxylate (3y)

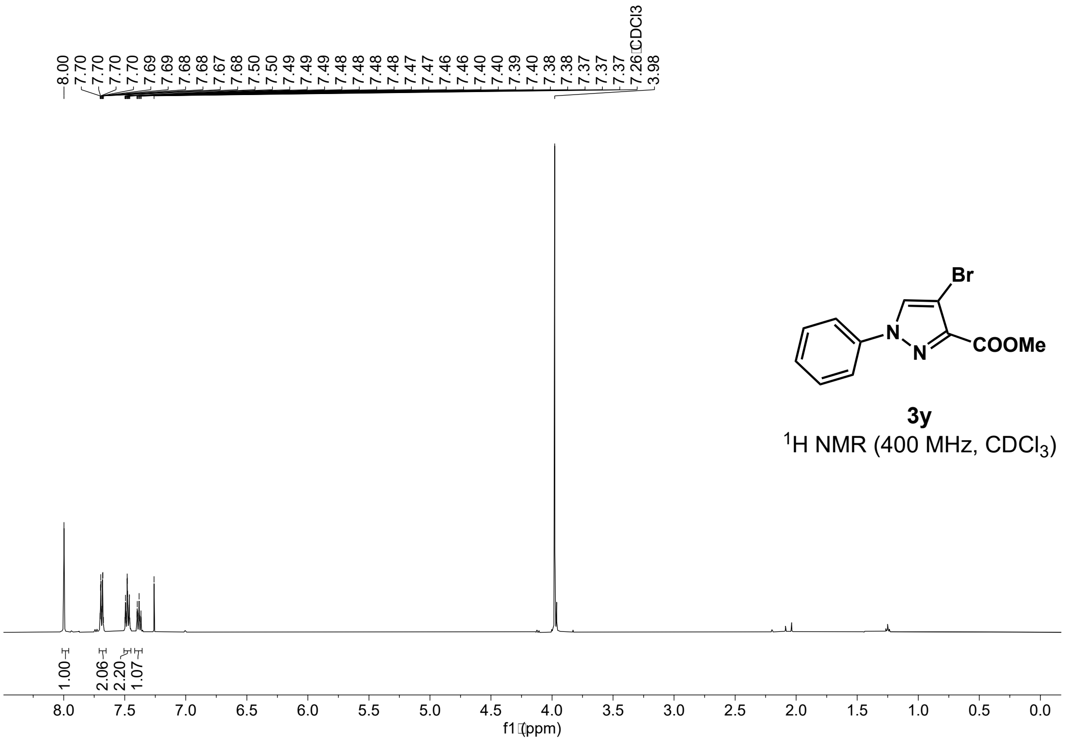

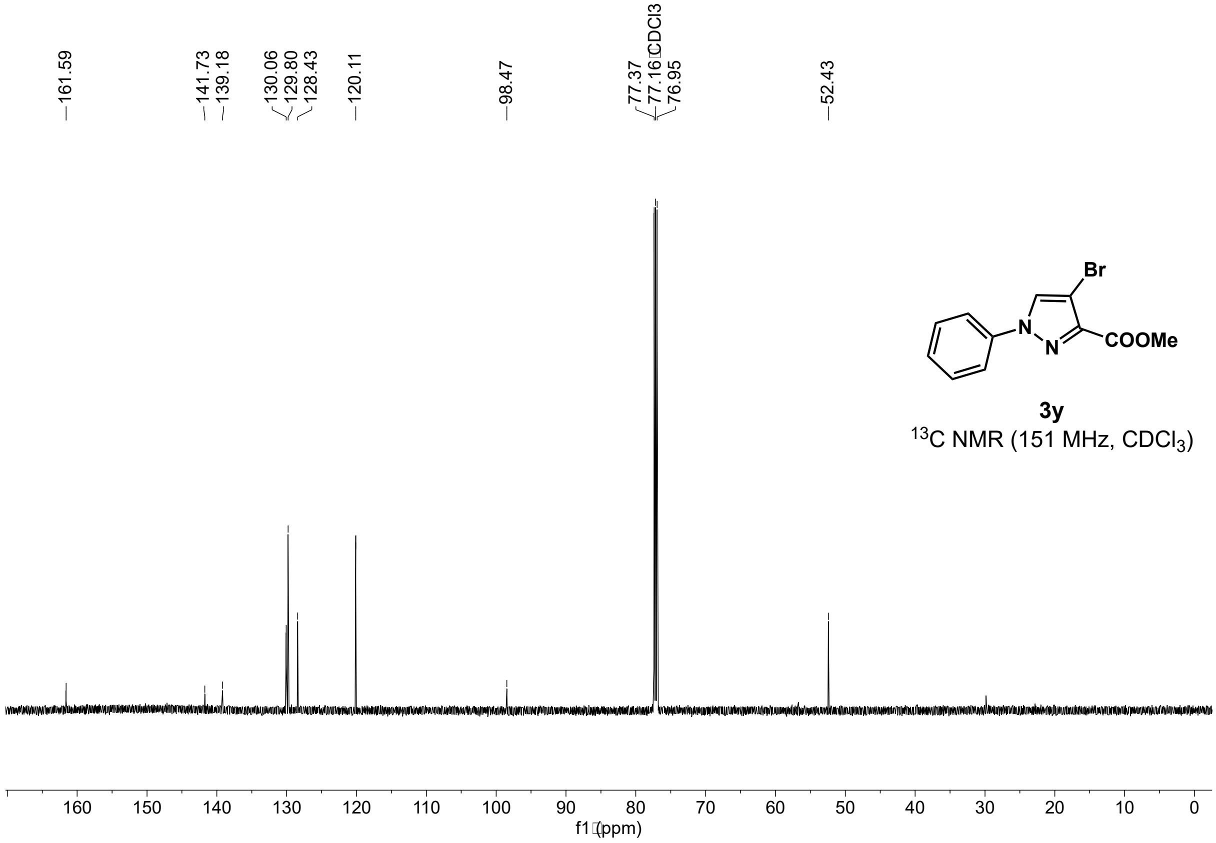

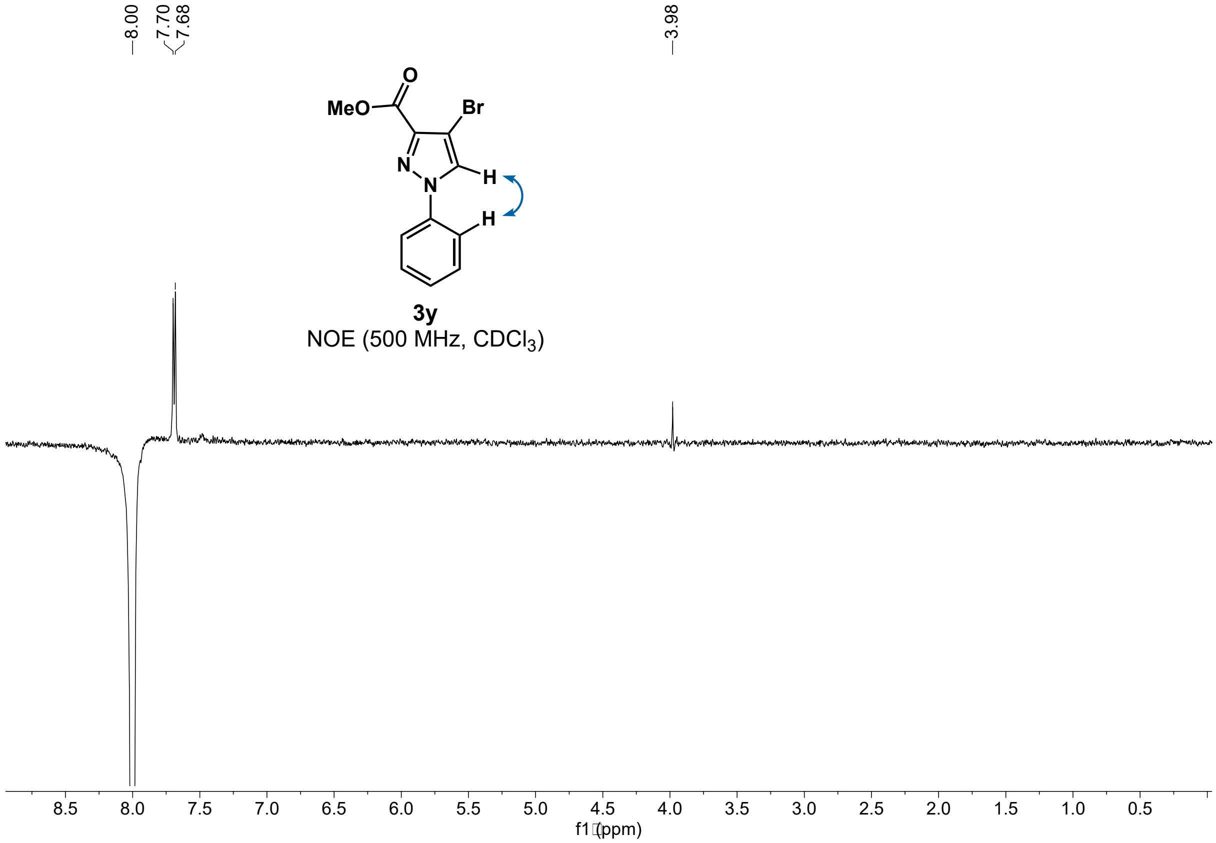
8.00
7.70
7.68
3.98
MeO
O
Br
N
N
H
H
3y
NOE (500 MHz, CDCl3)
8.5
8.0
7.5
7.0
6.5
6.0
5.5
5.0
4.5
4.0
3.5
3.0
2.5
2.0
1.5
1.0
0.5
f1 (ppm)

Ethyl 5-cyclopropyl-1-phenyl-1*H*-pyrazole-4-carboxylate (3z)

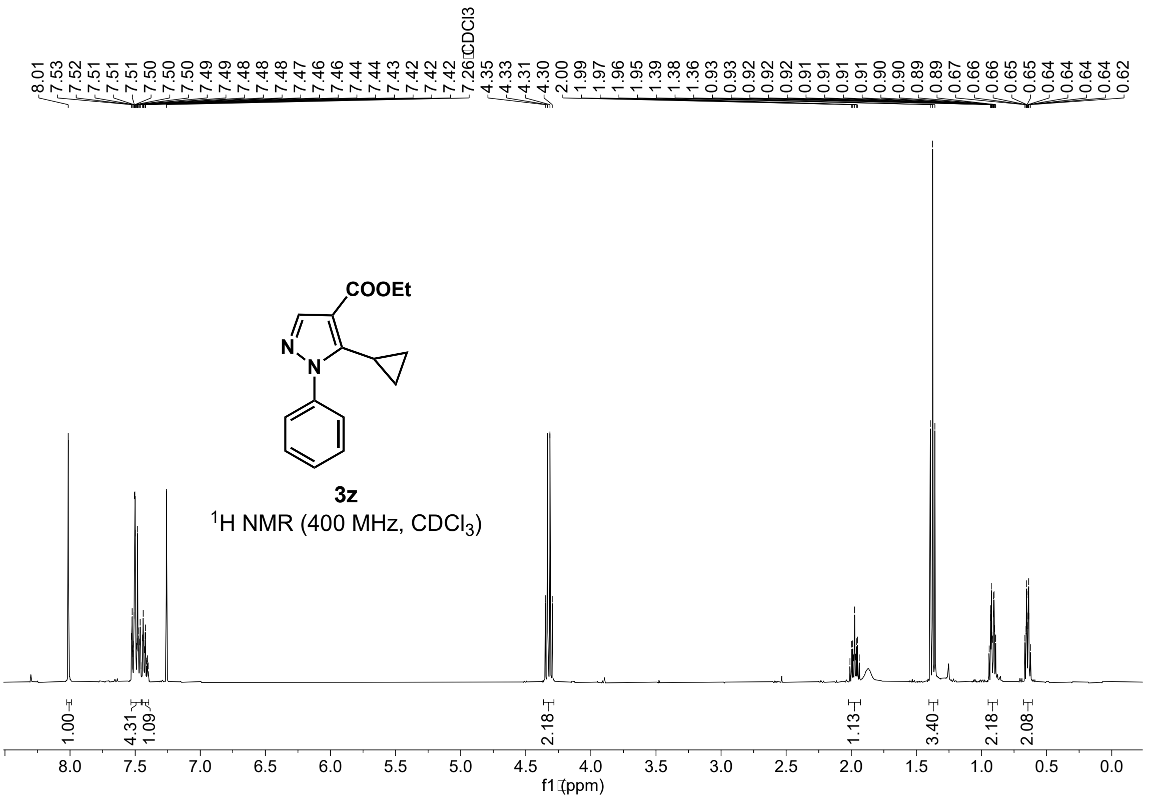

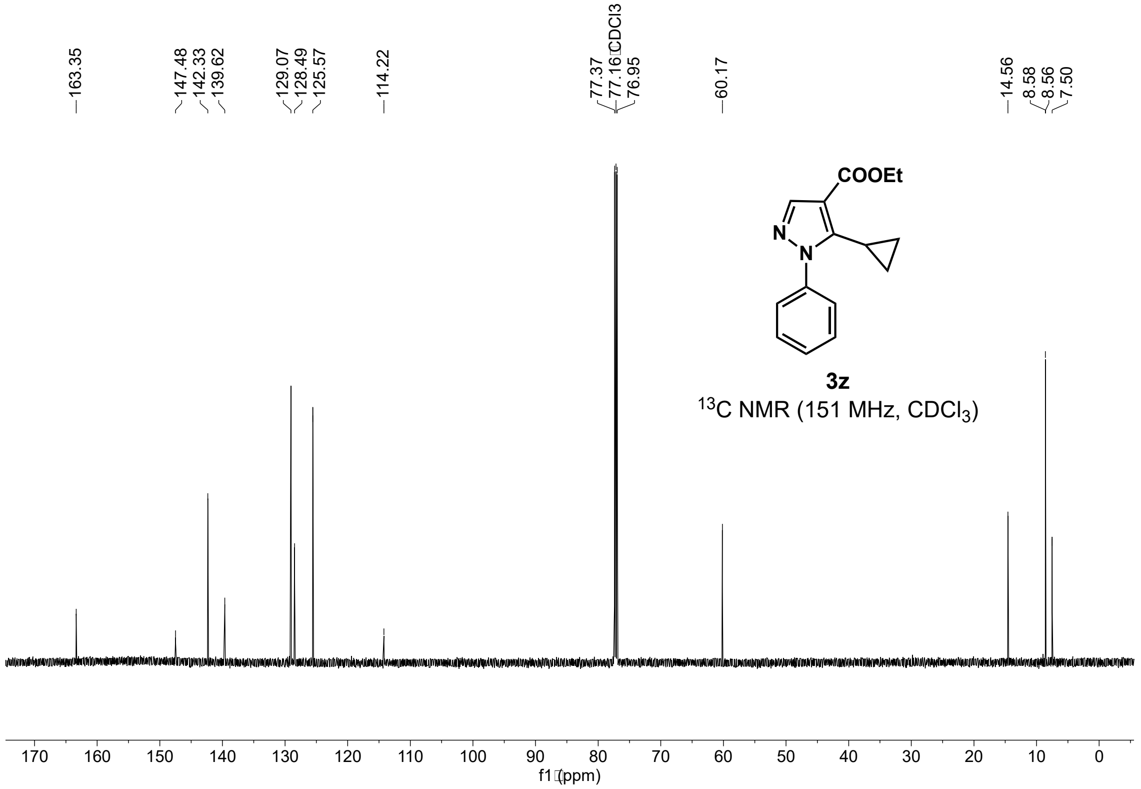

3,4-Dimethyl-1,5-diphenyl-1H-pyrazole (3aa)

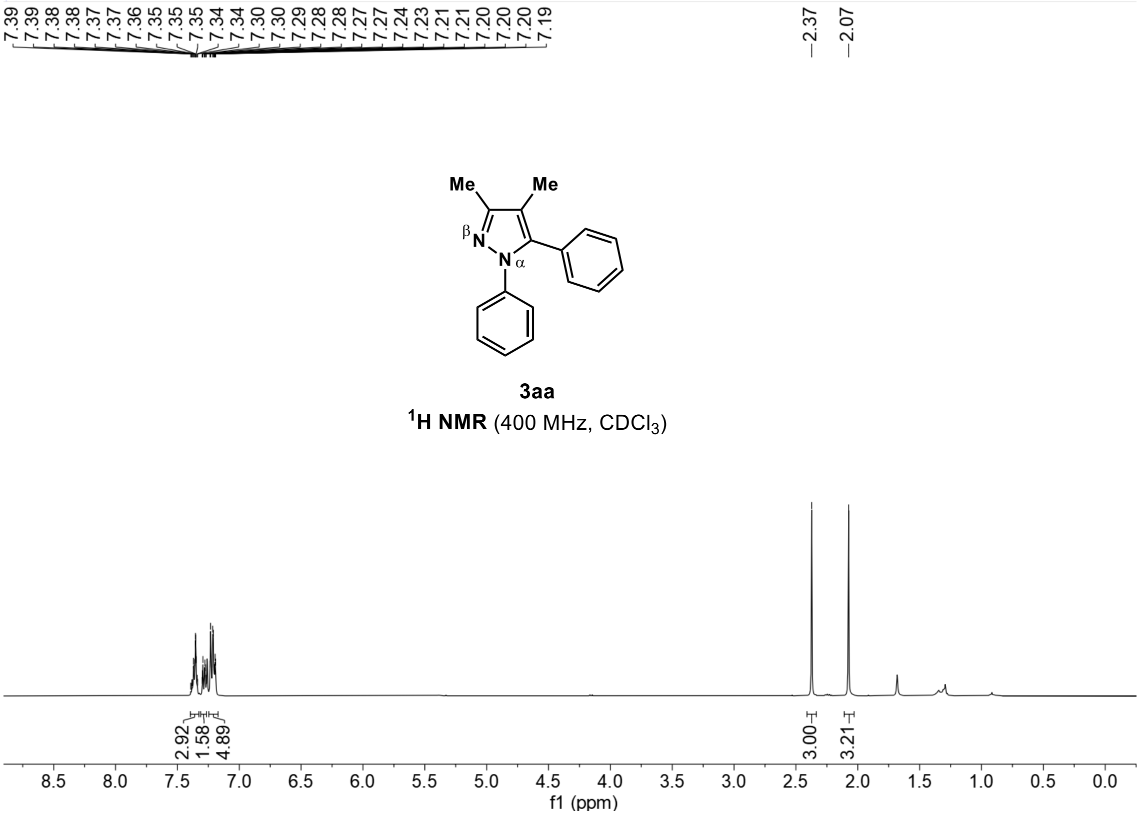

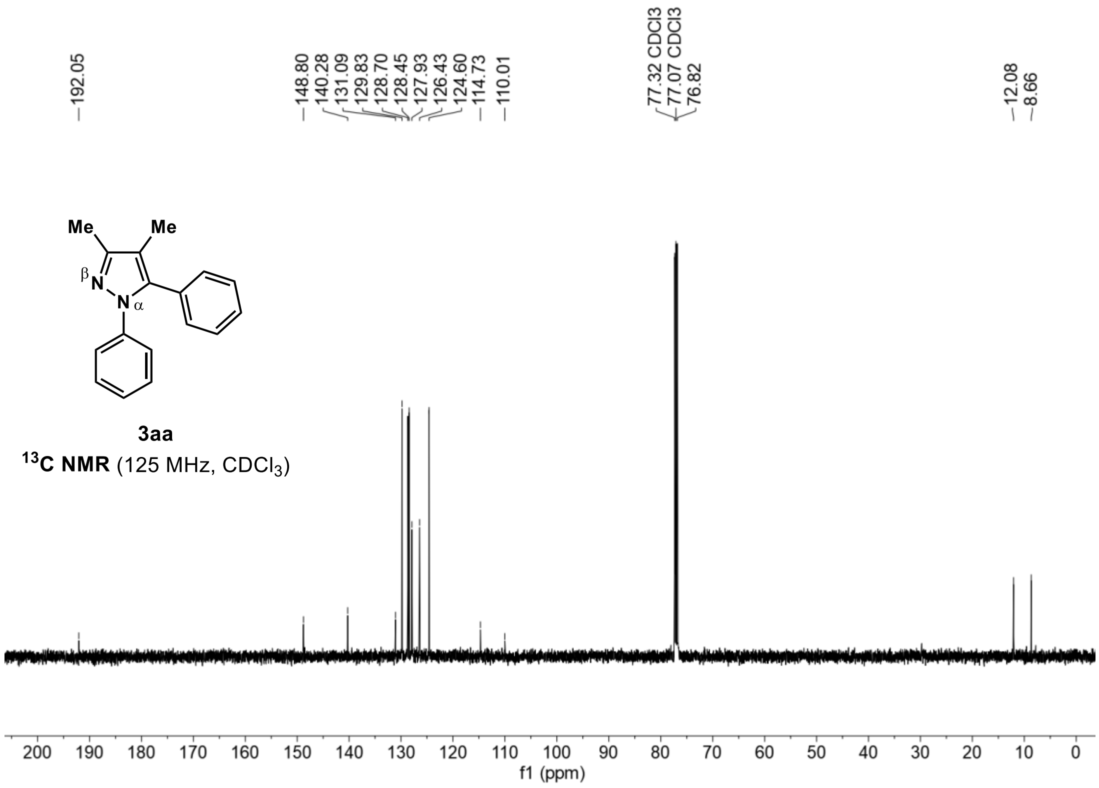

1-Phenyl-1*H*-indazole (3ab)

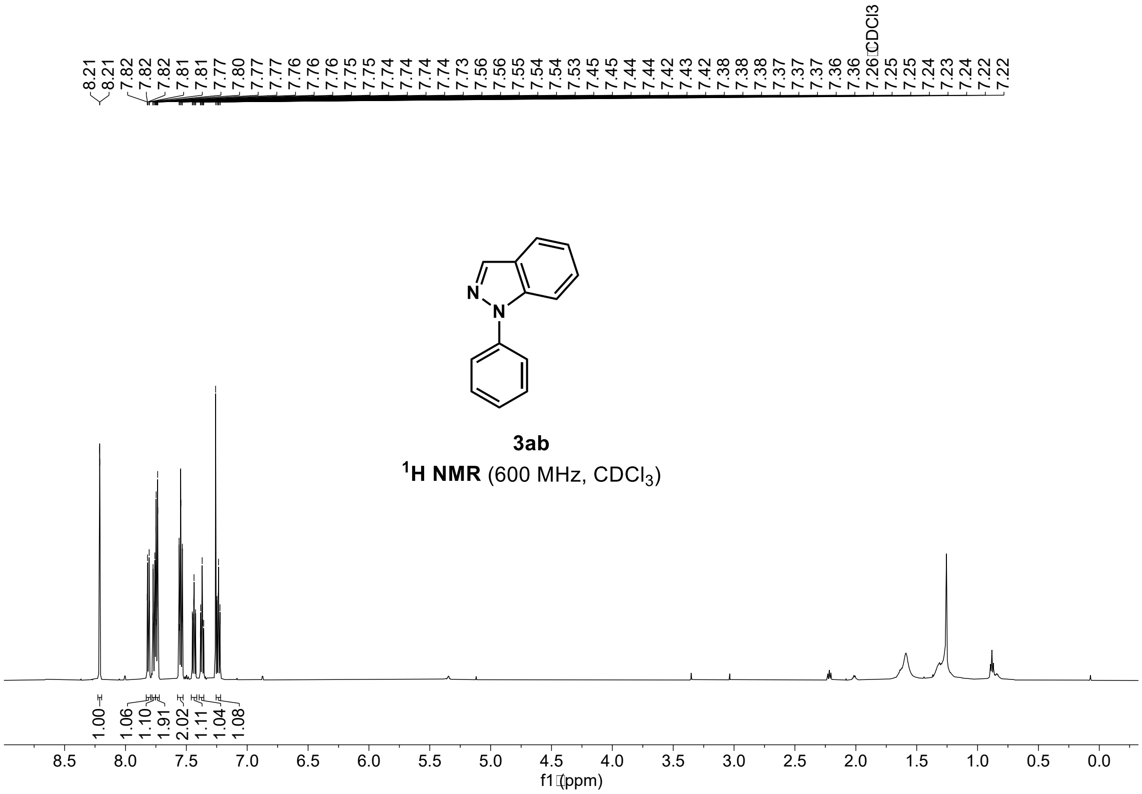

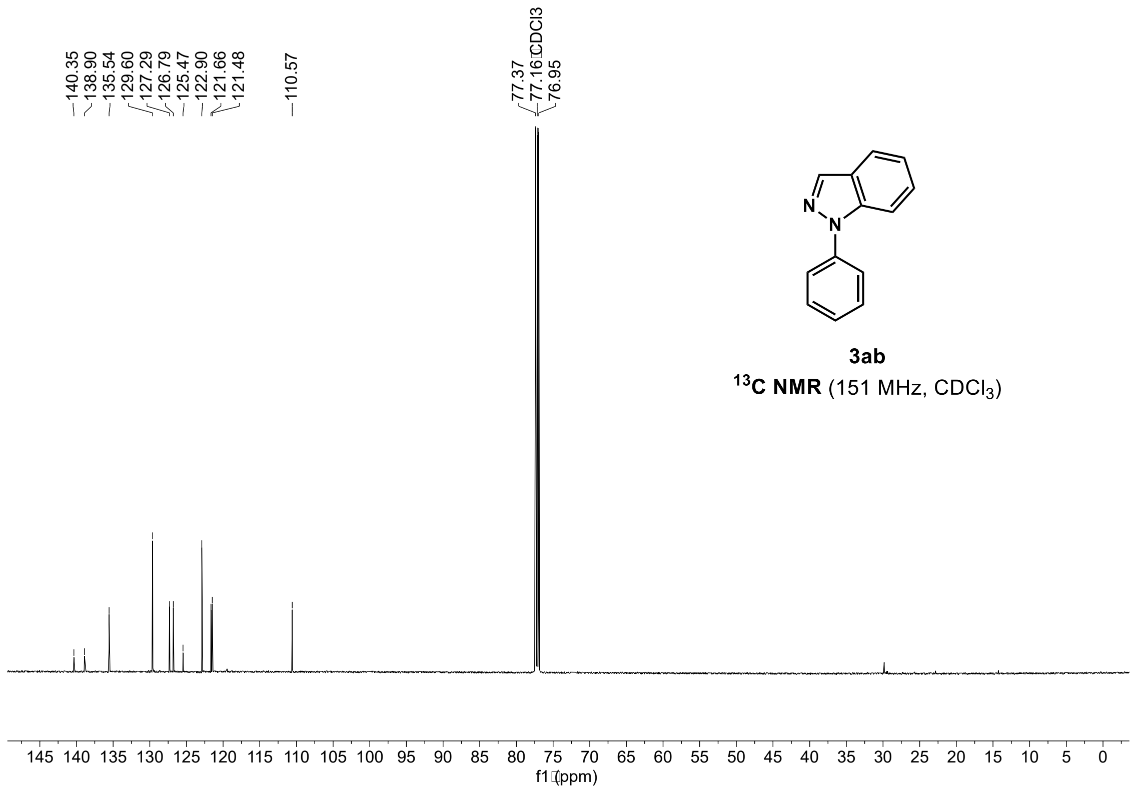

1,5-Diphenyl-1*H*-1,2,4-triazole (3ac)

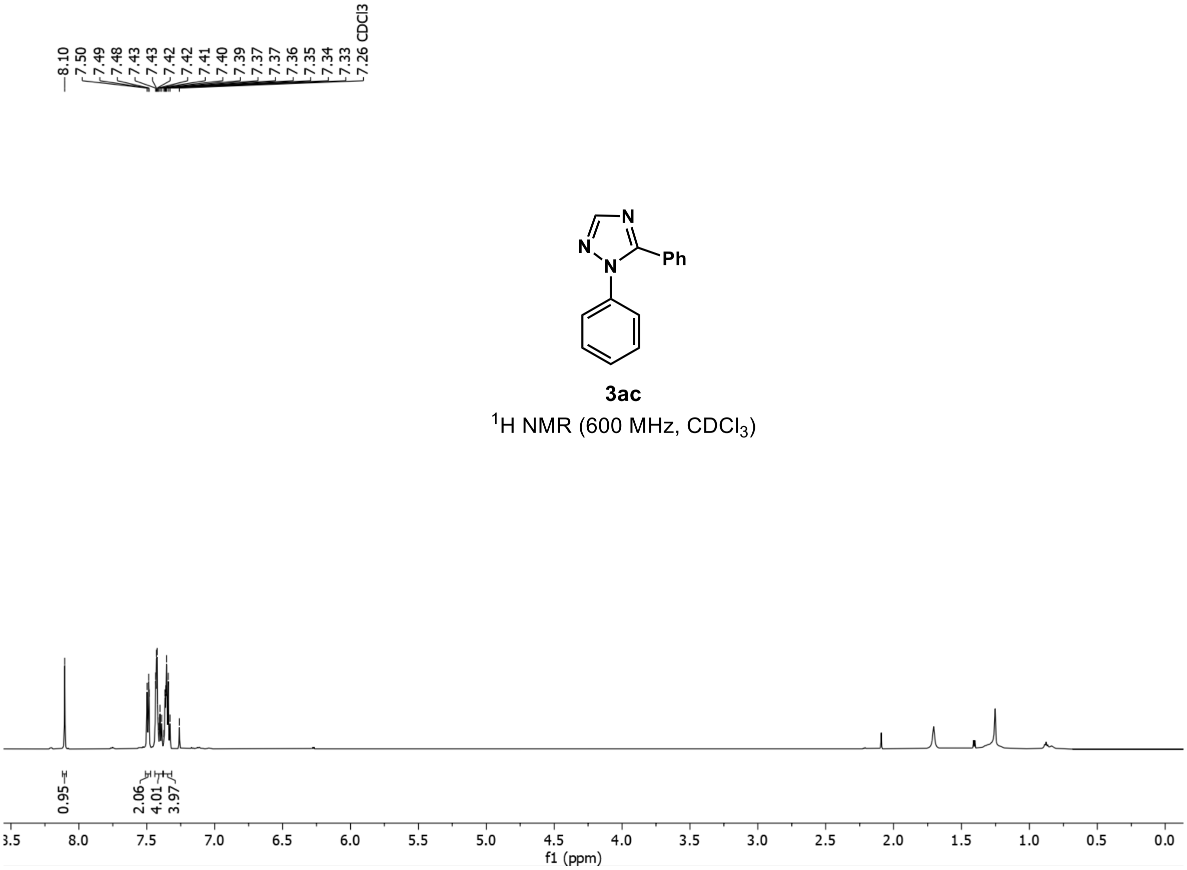

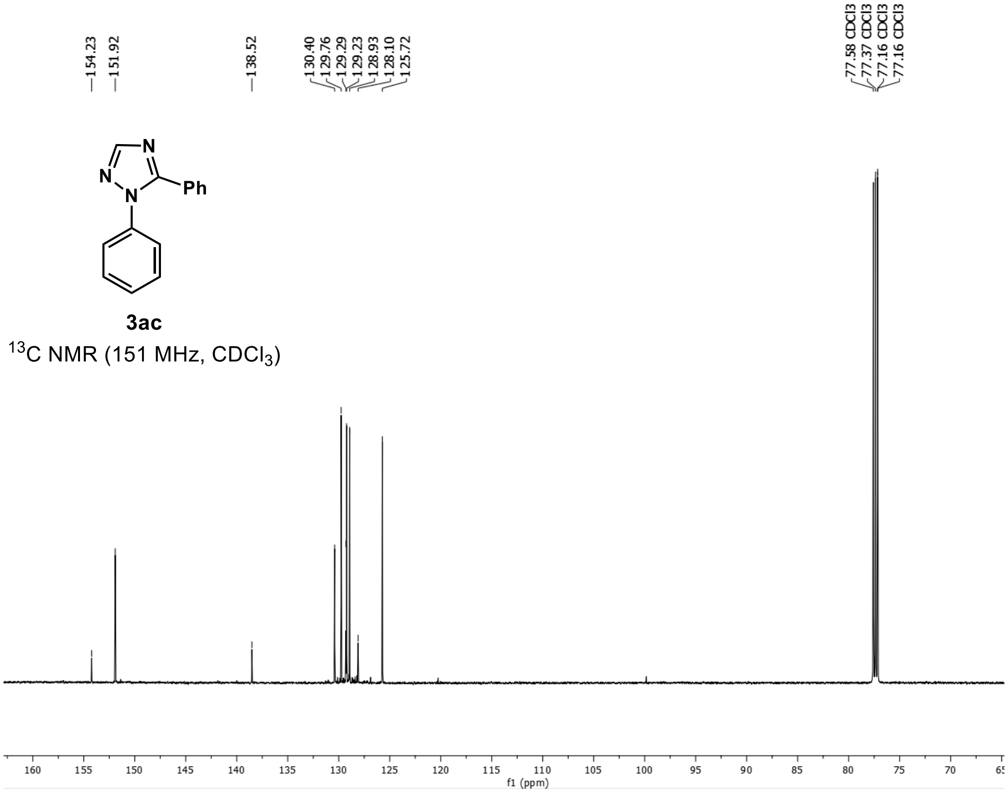

4-Bromo-2-phenyl-2*H*-1,2,3-triazole (3ad)

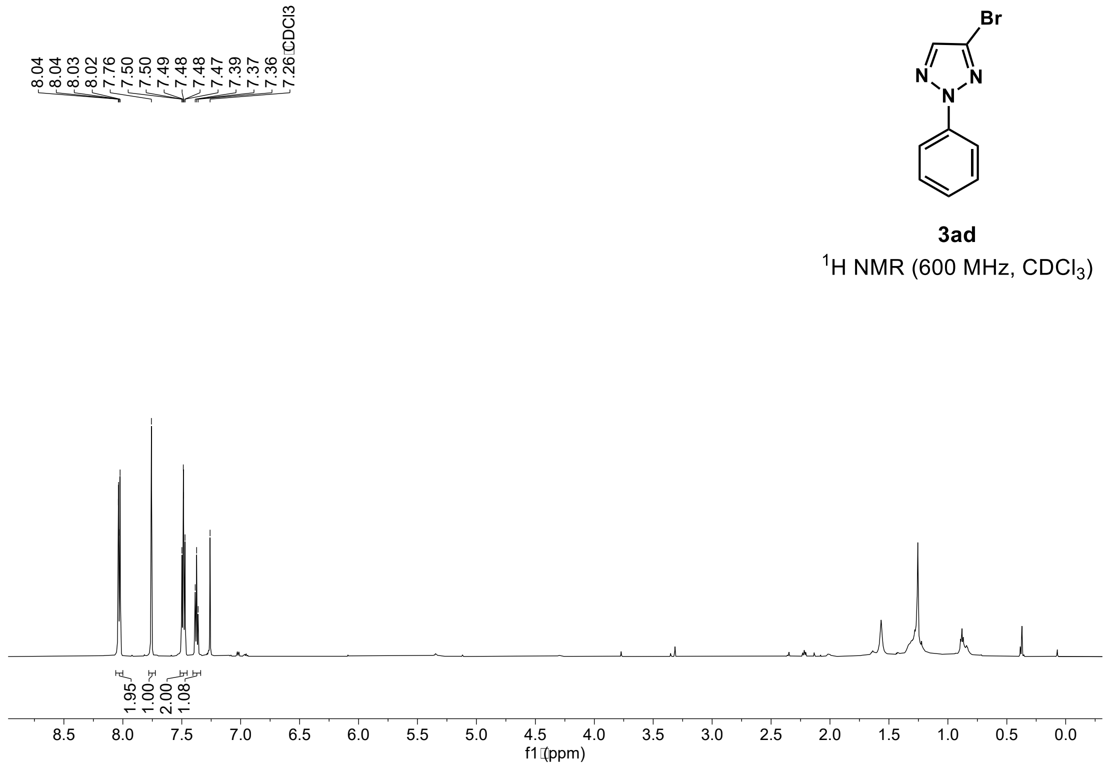

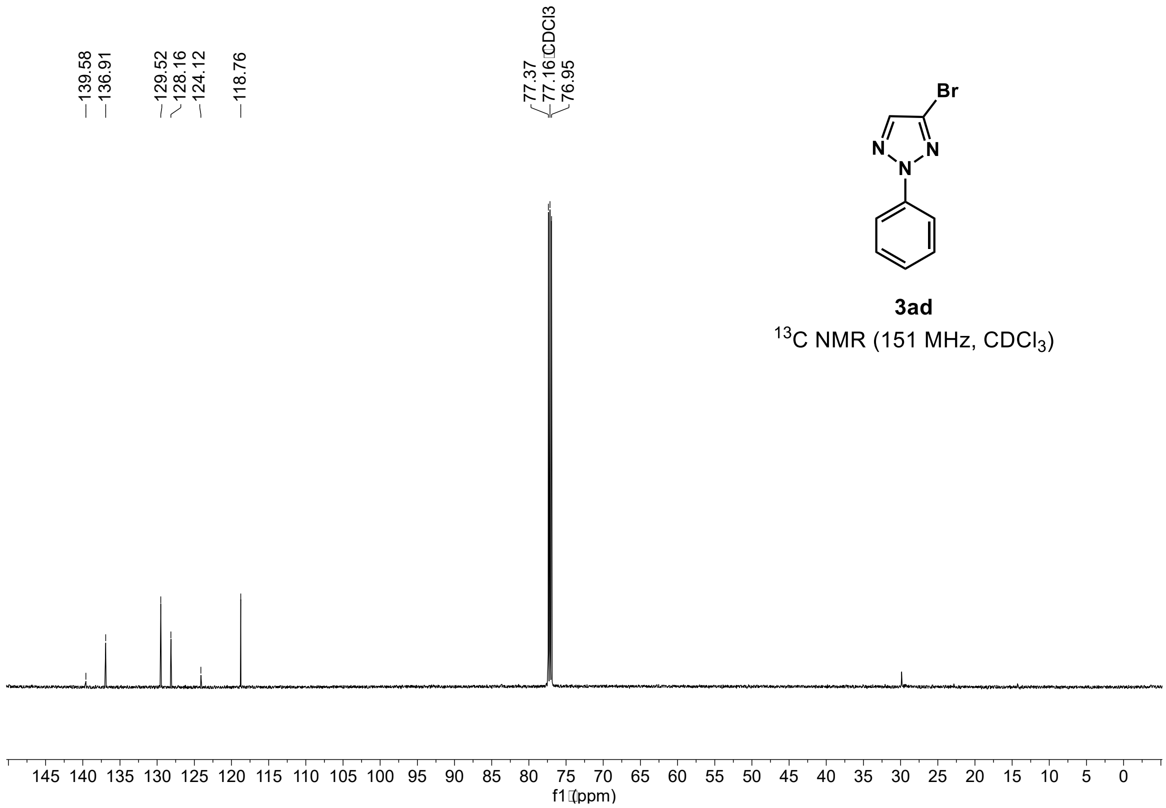

1,3-Diphenyl-1*H*-pyrazole (4a)

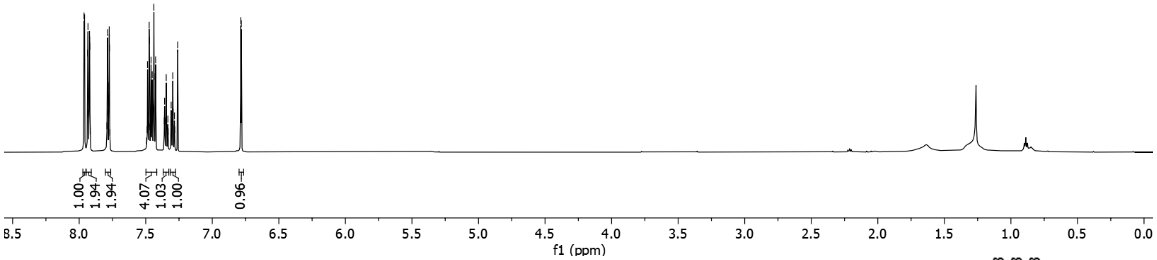

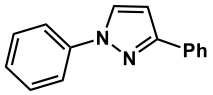

4a

^{1}H NMR (600 MHz, $CDCl_3$)

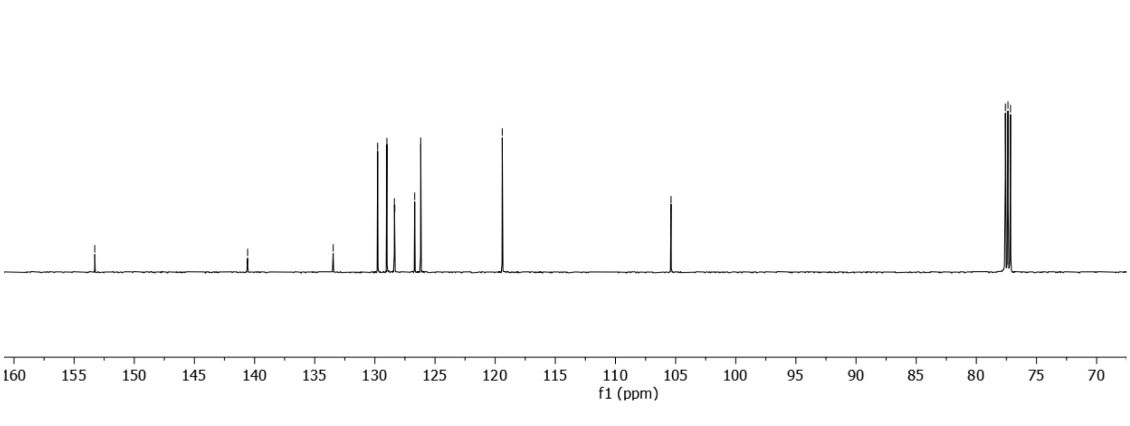

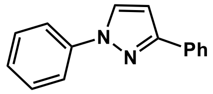

4a

^{13}C NMR (151 MHz, $CDCl_3$)

3-Methyl-1-phenyl-1*H*-pyrazole (4b)

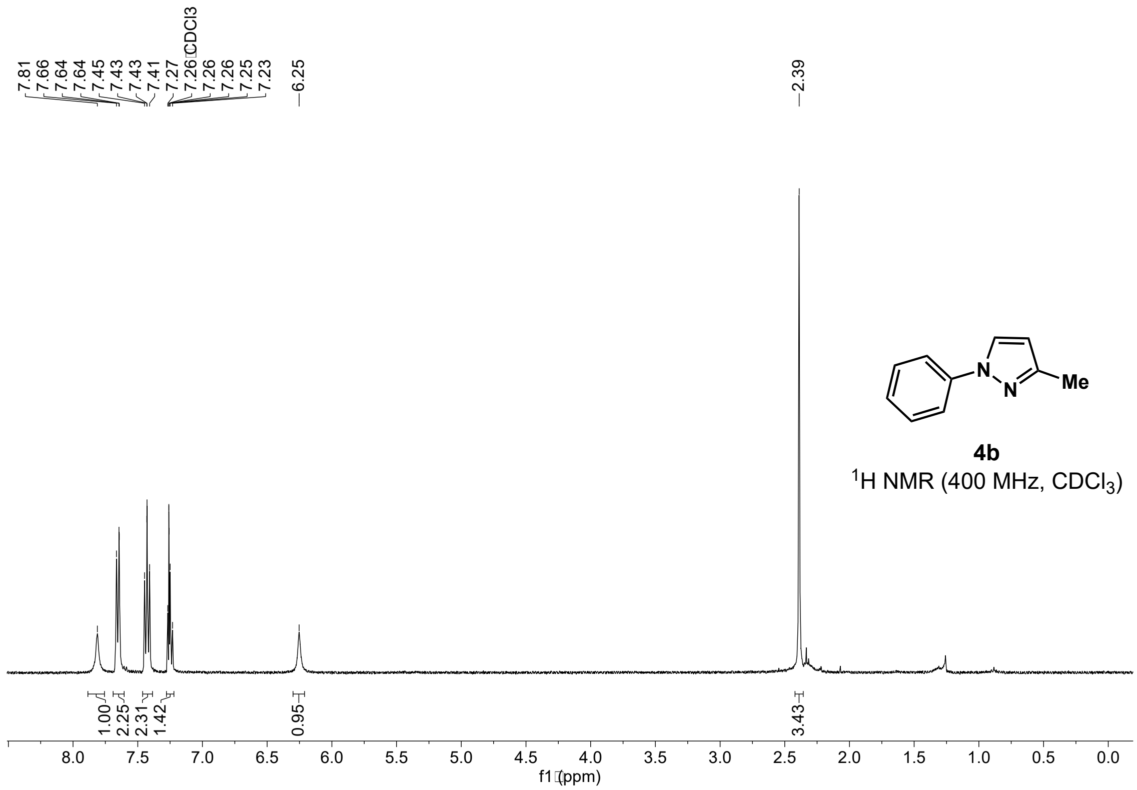

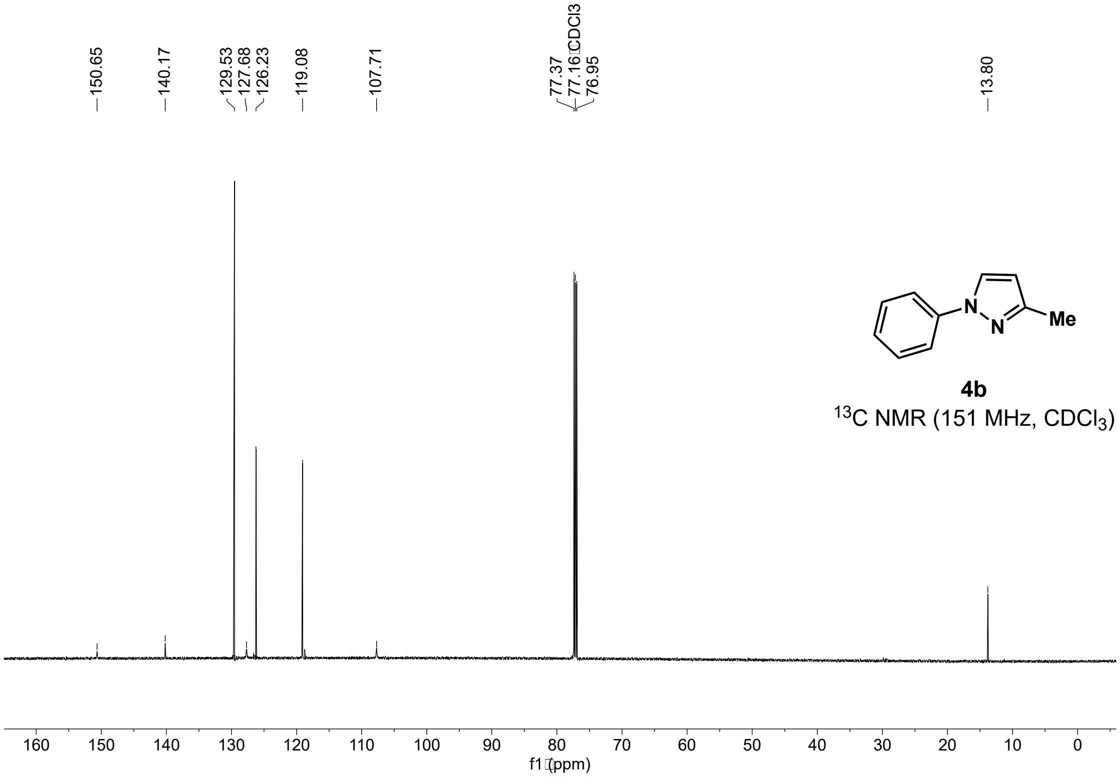

3-Cyclopropyl-1-phenyl-1*H*-pyrazole (4c)

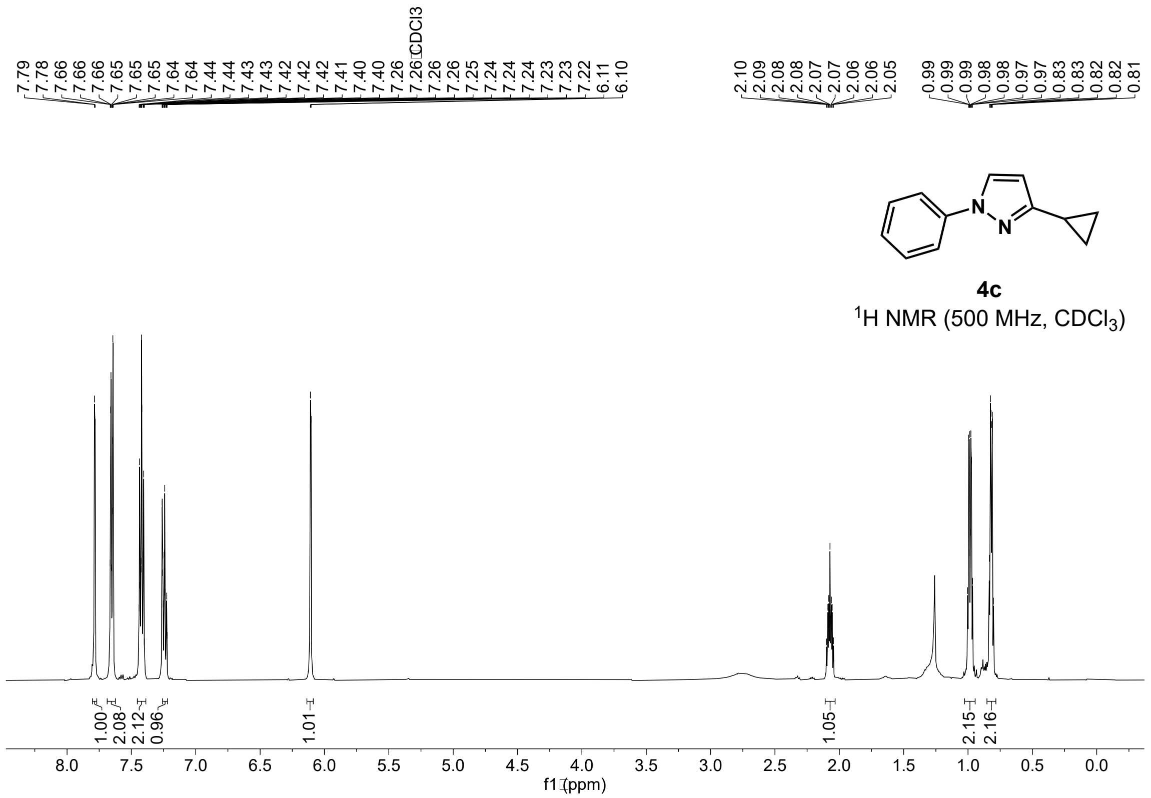

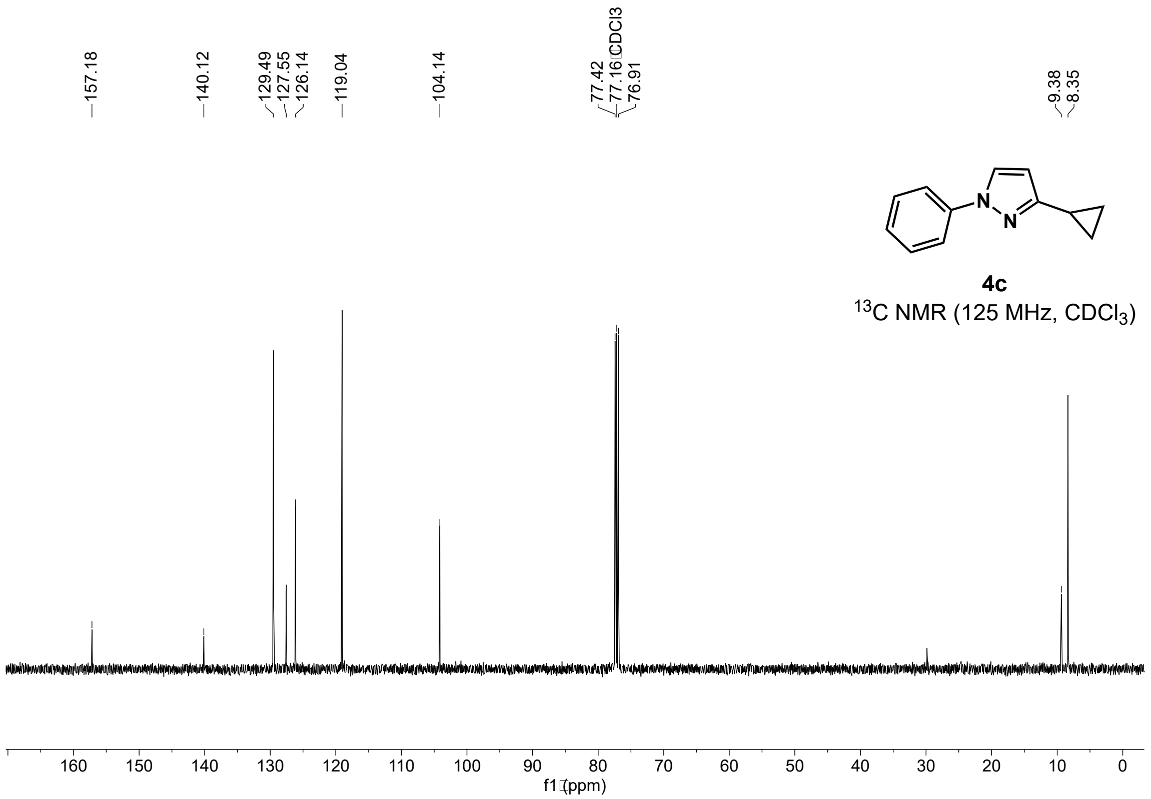

3-Isopropyl-1-phenyl-1*H*-pyrazole (4d)

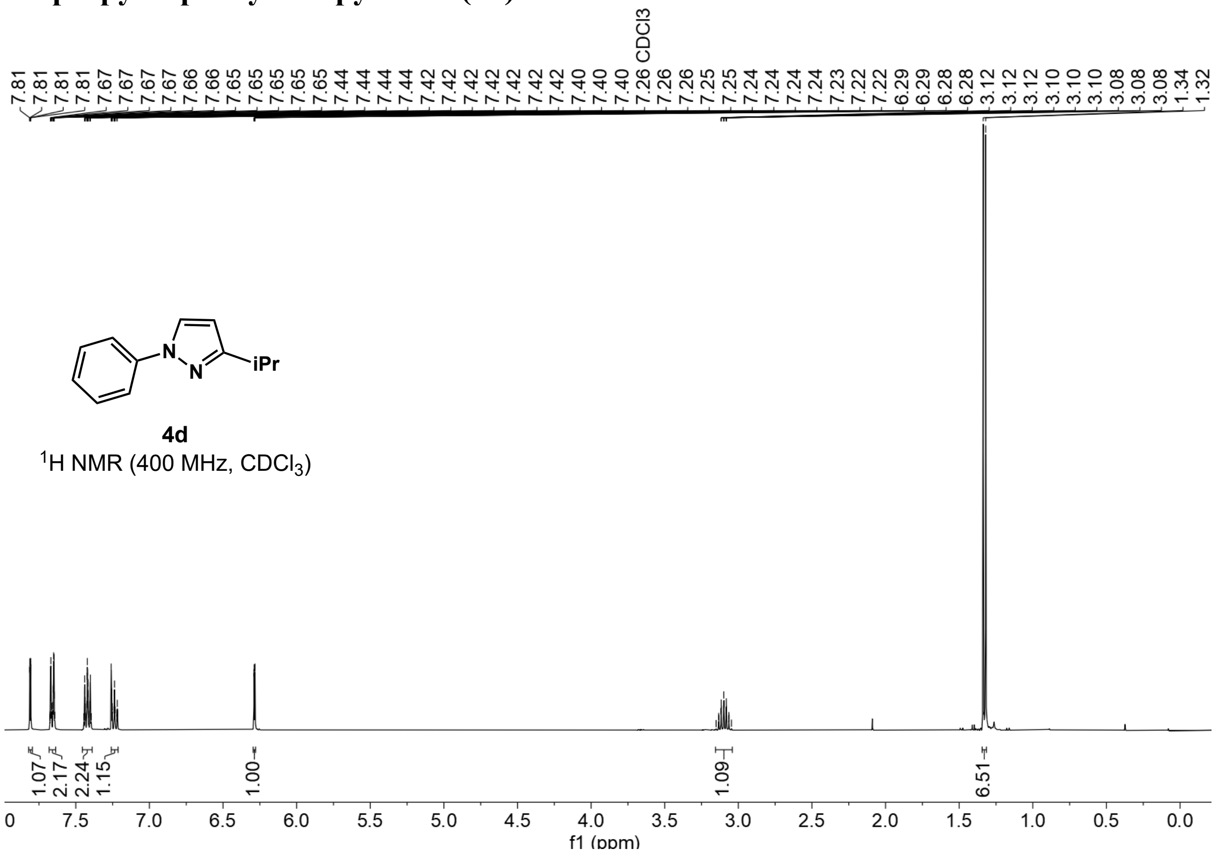

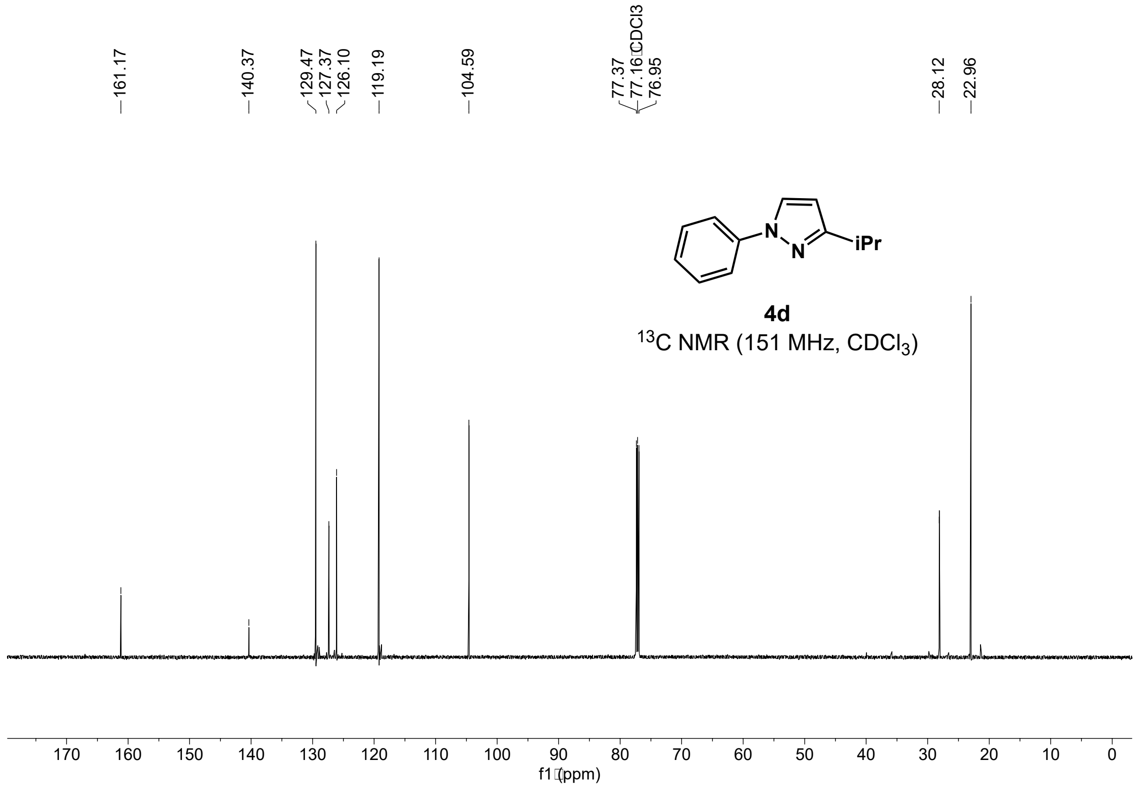

3-(Tert-butyl)-1-phenyl-1*H*-pyrazole (4e)

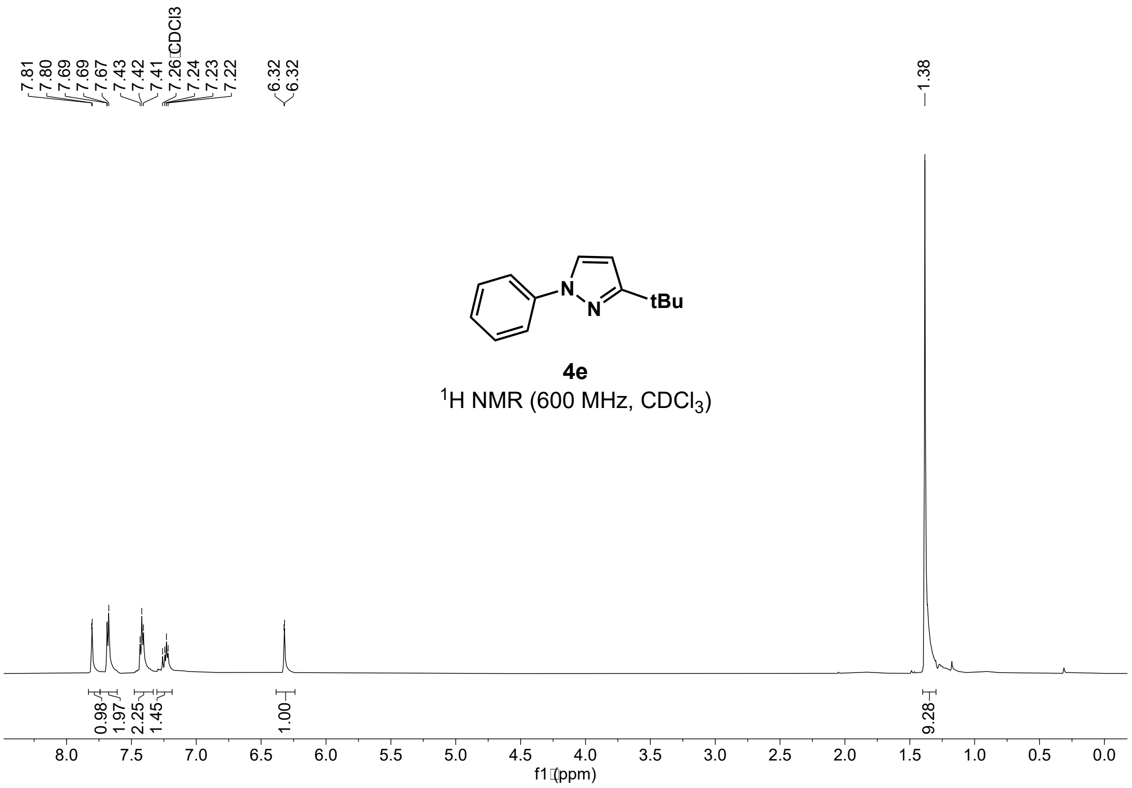

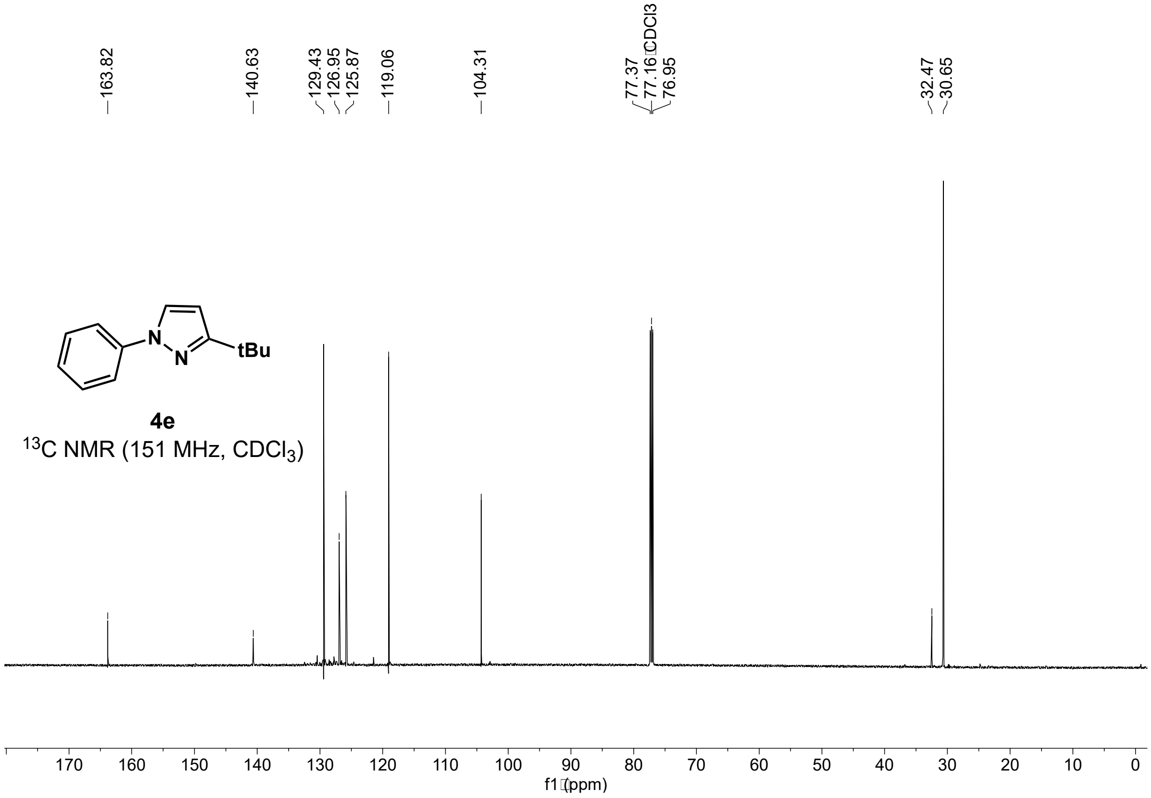

1-Phenyl-3-(o-tolyl)-1*H*-pyrazole (4f)

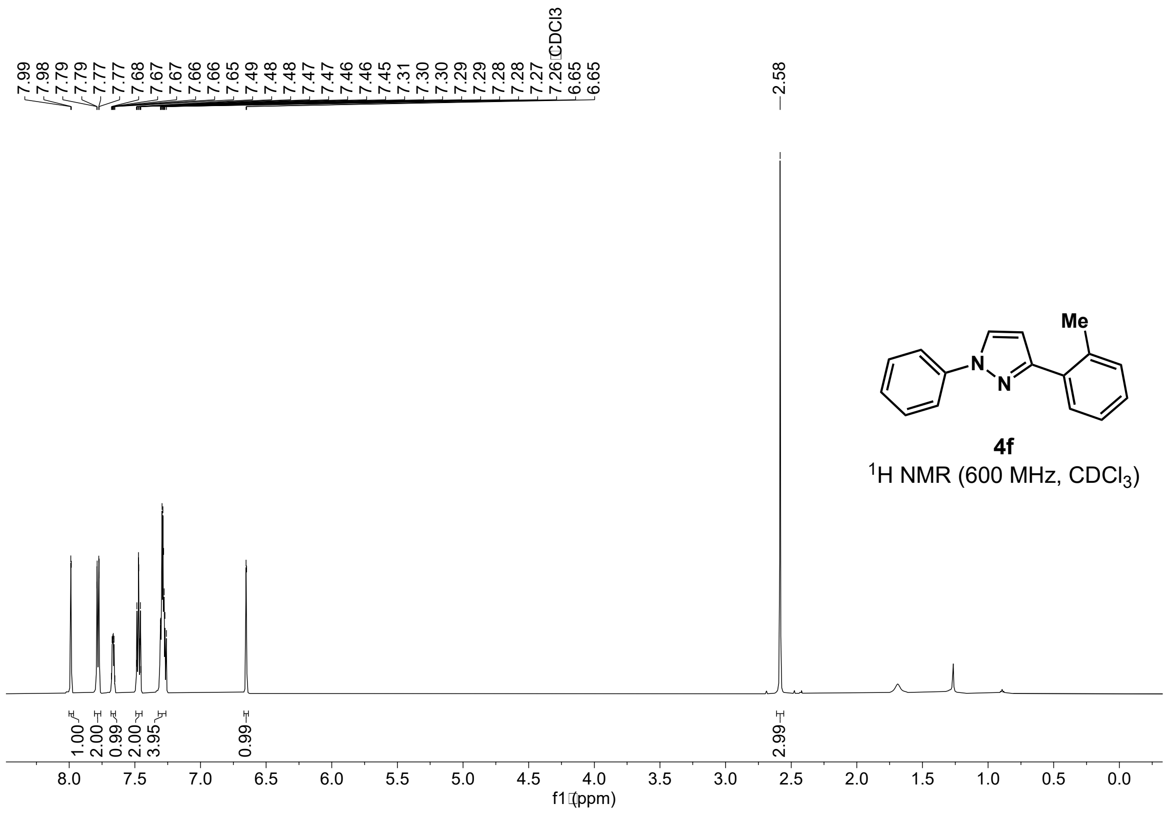

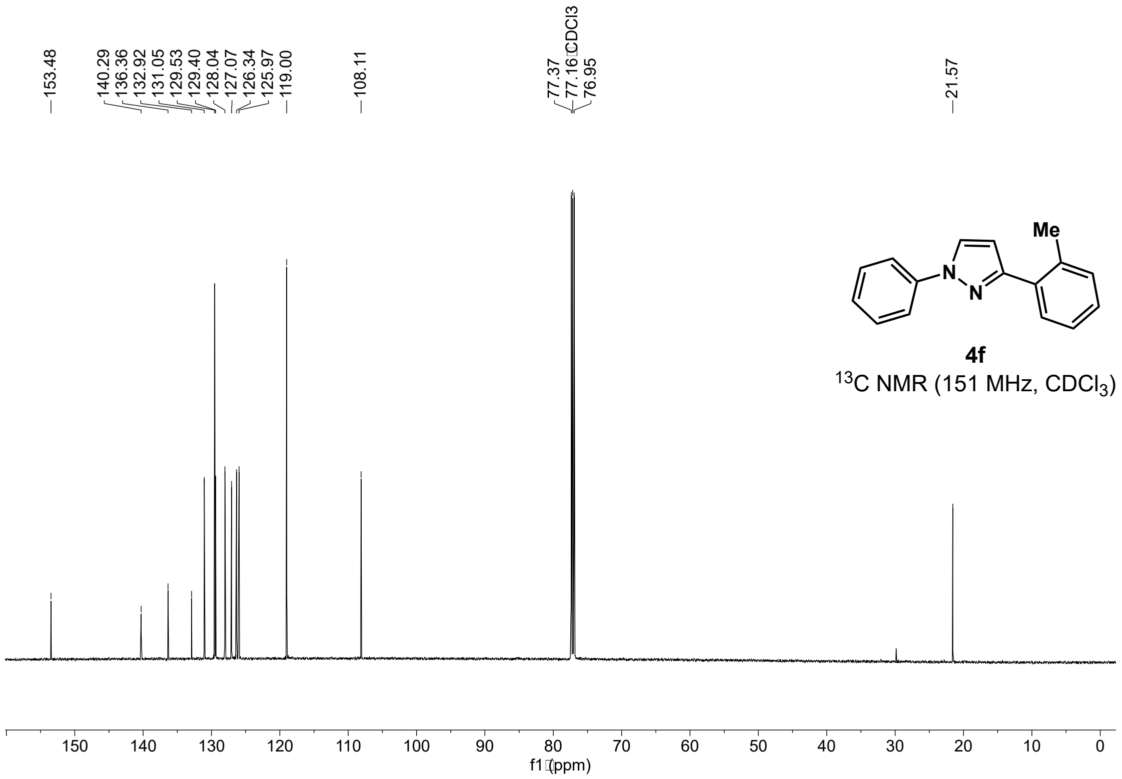

3-(2-Fluorophenyl)-1-phenyl-1*H*-pyrazole (4g)

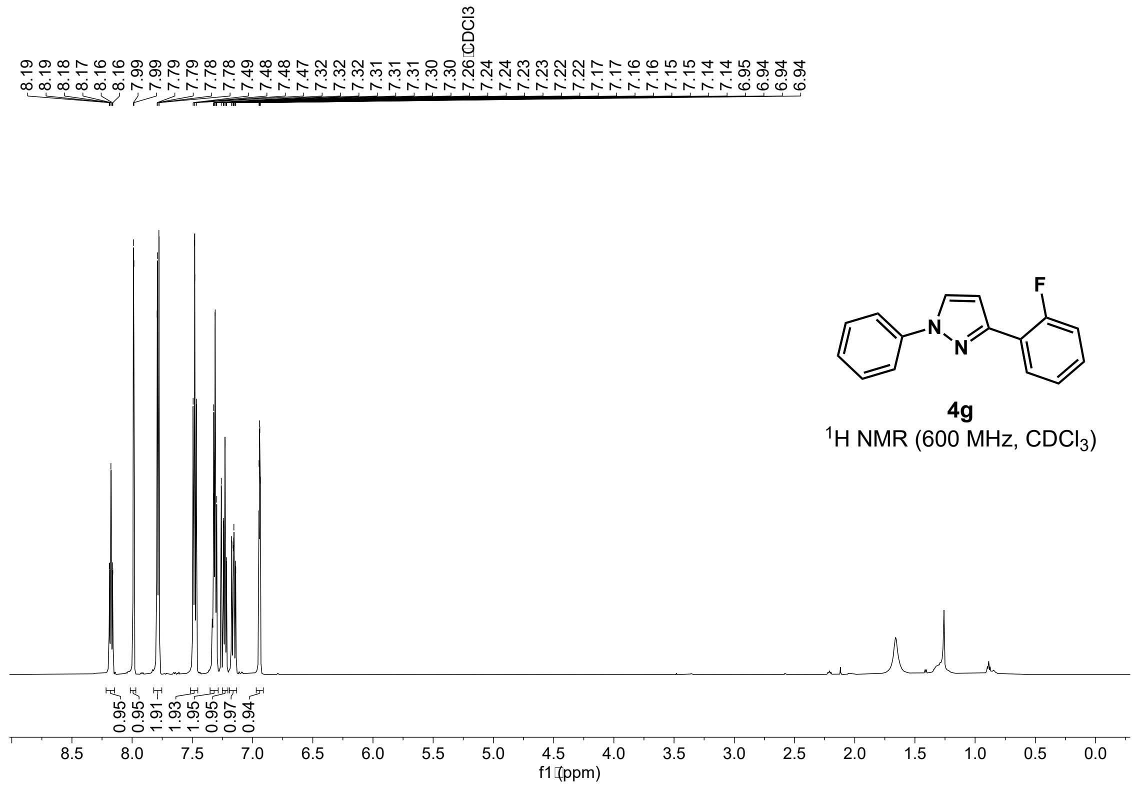

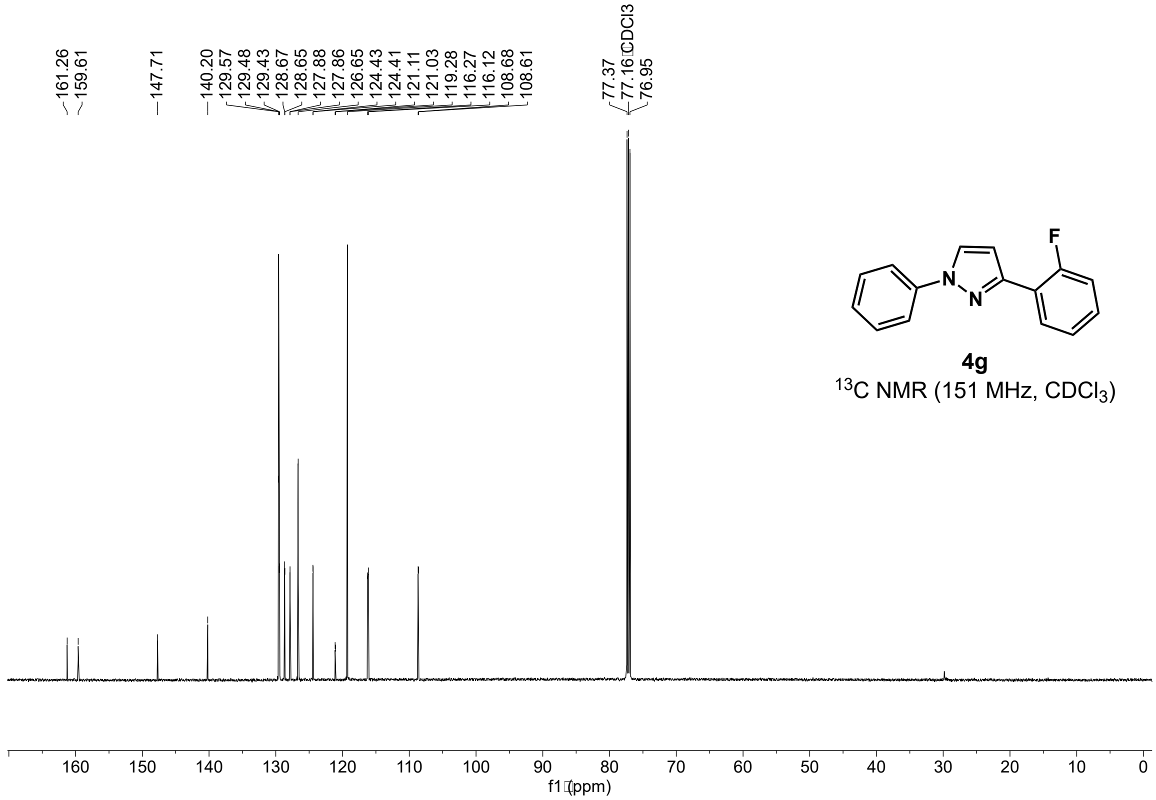

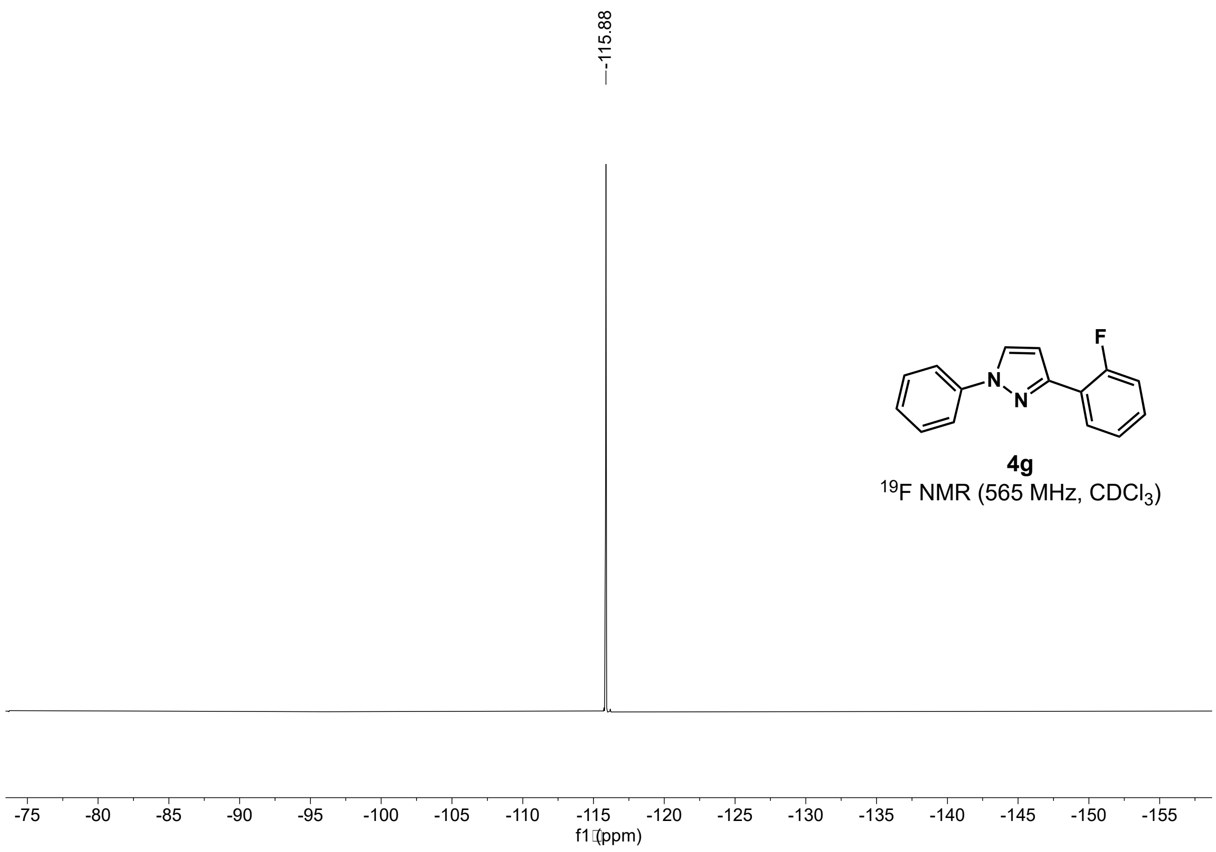
-115.88
4g
19F NMR (565 MHz, CDCl3)
-75
-80
-85
-90
-95
-100
-105
-110
-115
-120
-125
-130
-135
-140
-145
-150
-155
f1 (ppm)

3-(2-Bromophenyl)-1-phenyl-1*H*-pyrazole (4h)

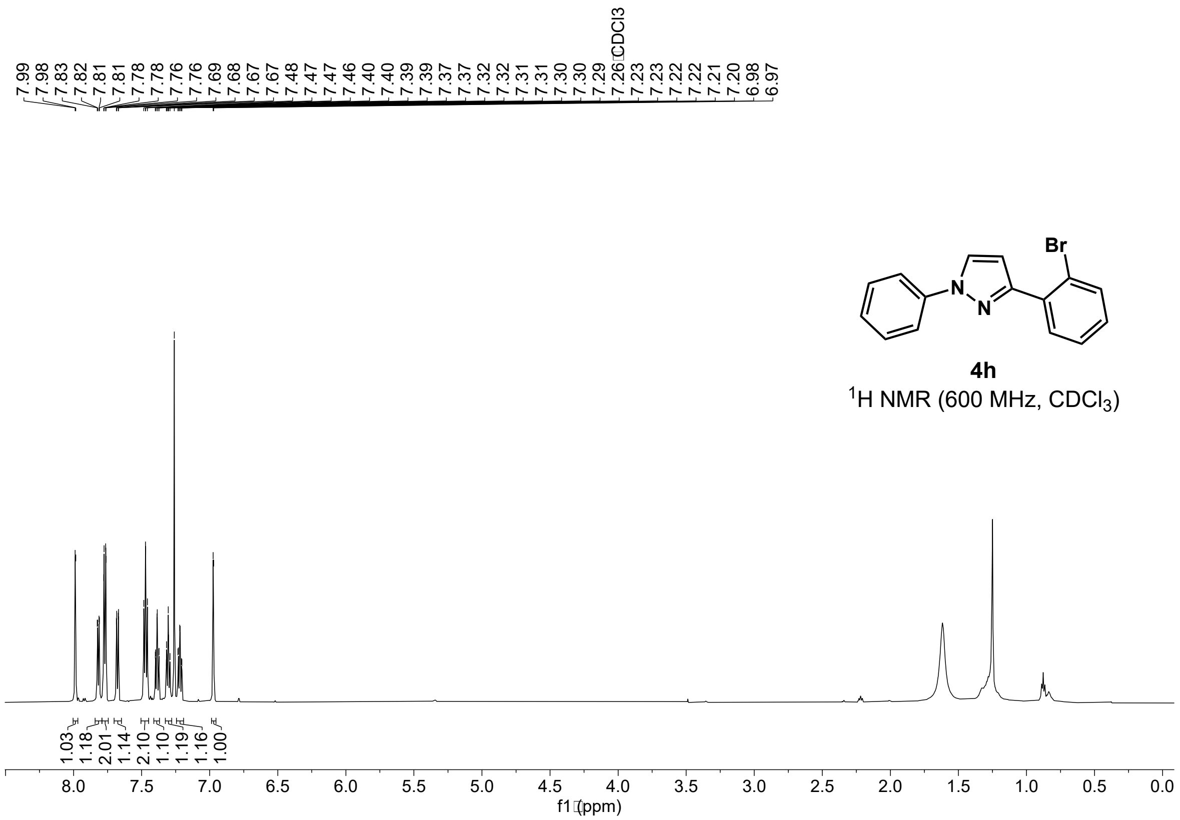

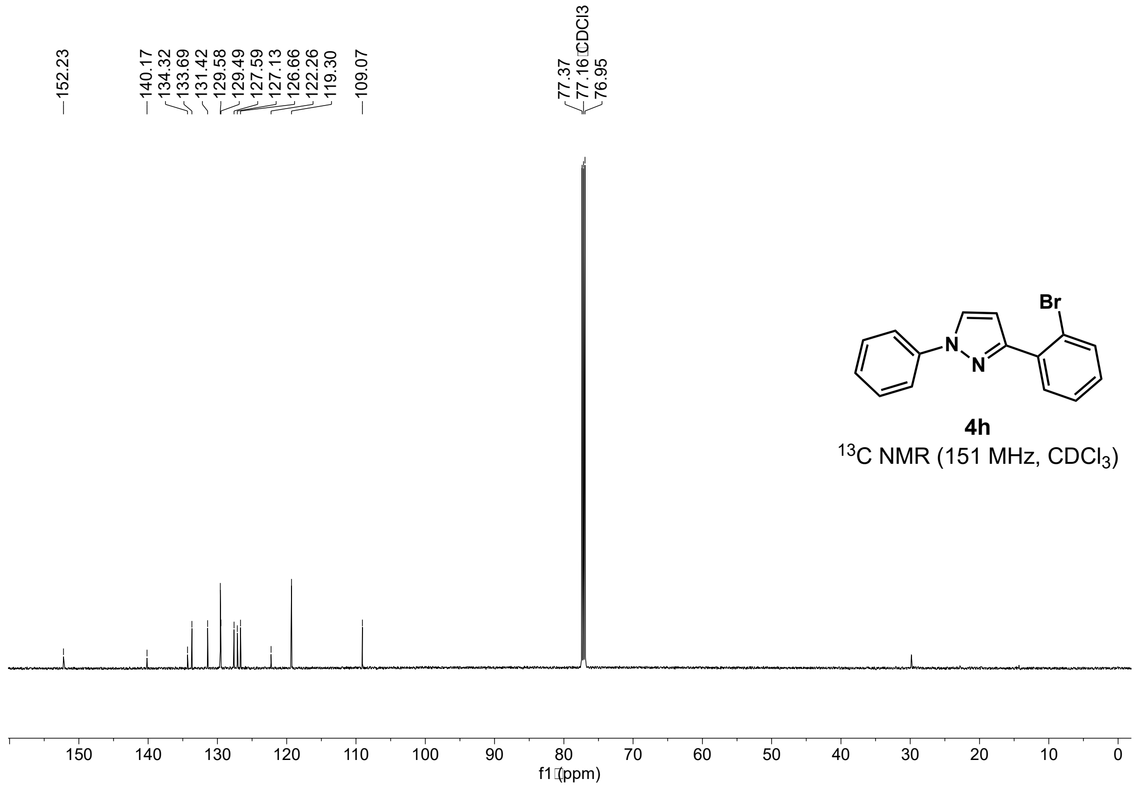

3-(2-Methoxyphenyl)-1-phenyl-1*H*-pyrazole (4i)

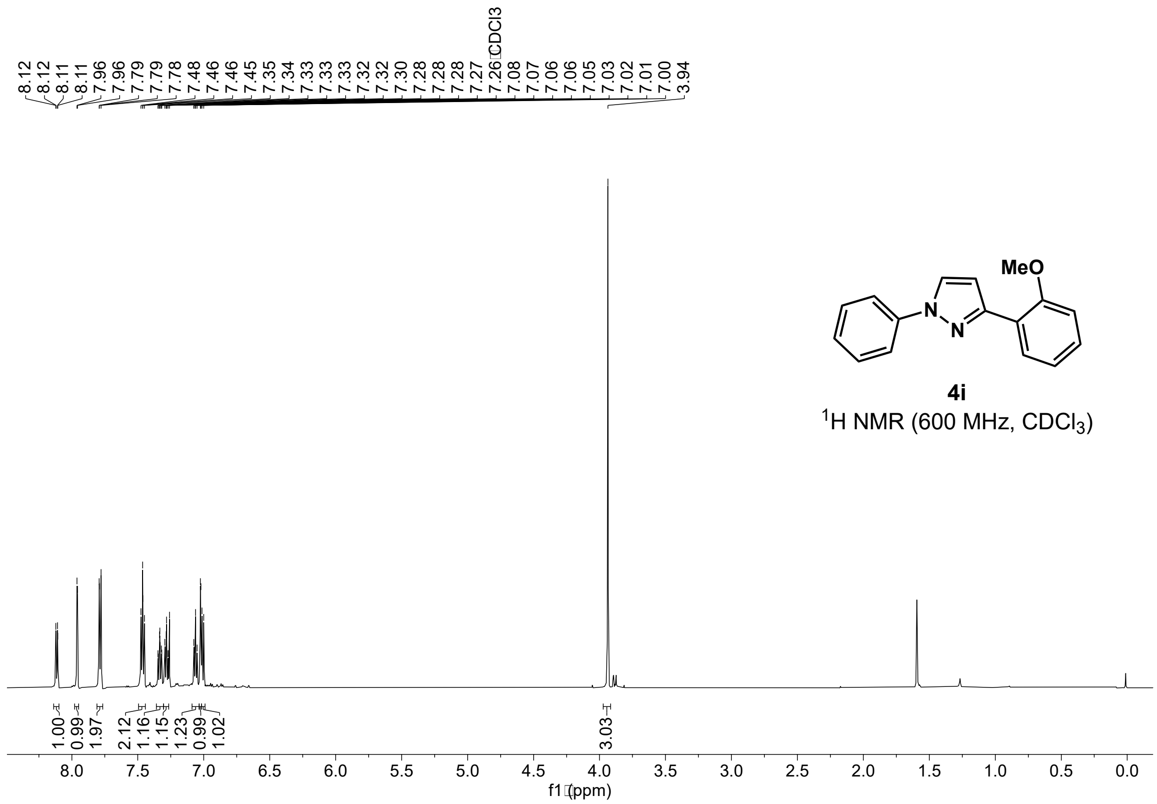

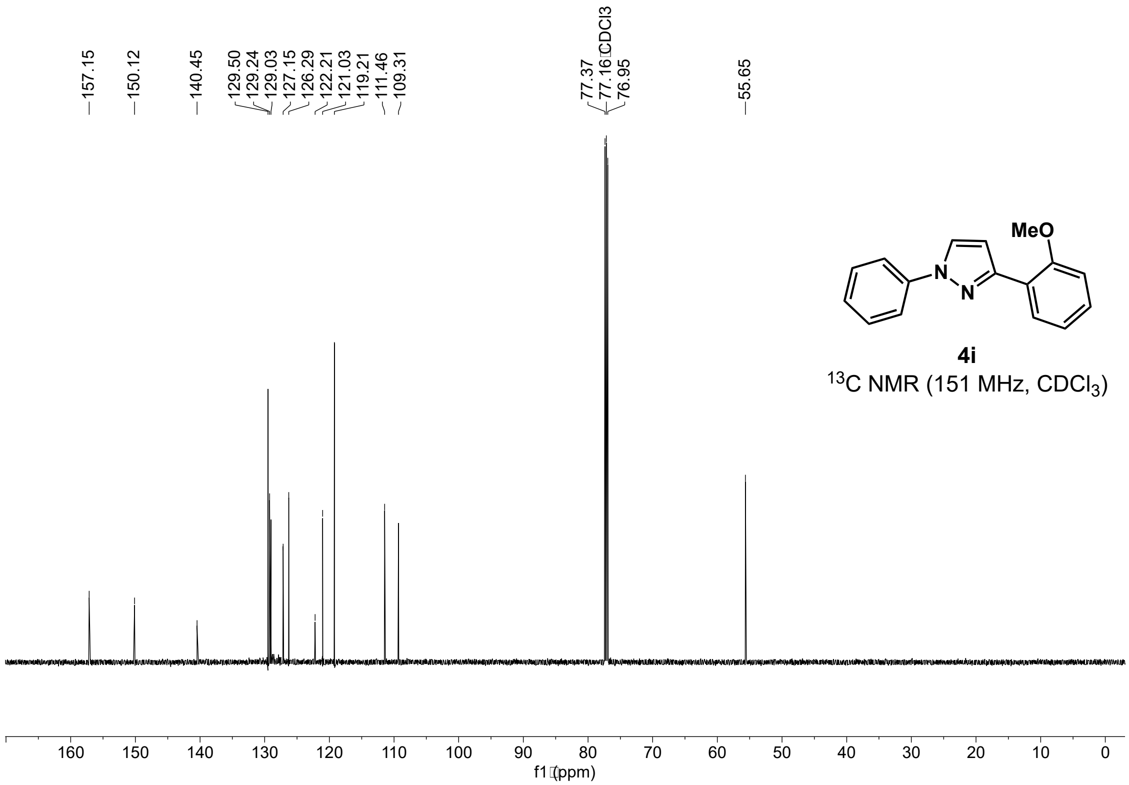

1-Phenyl-3-(p-tolyl)-1*H*-pyrazole (4j)

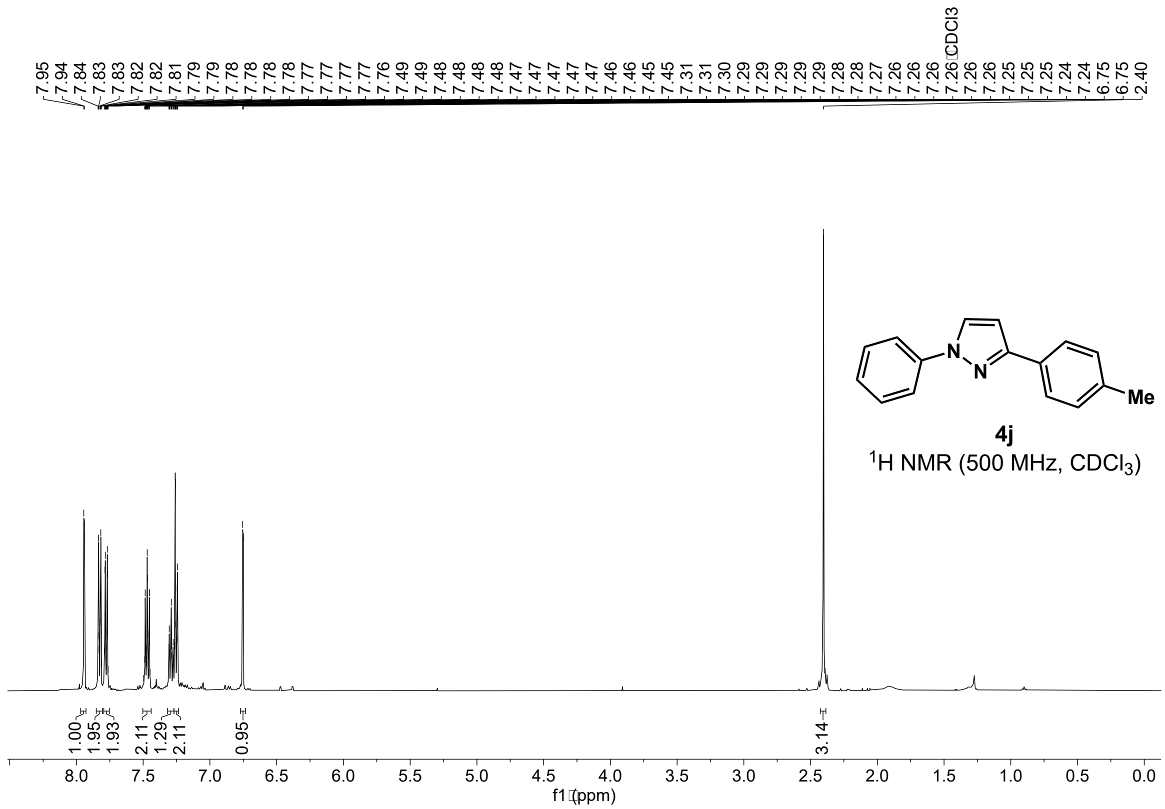

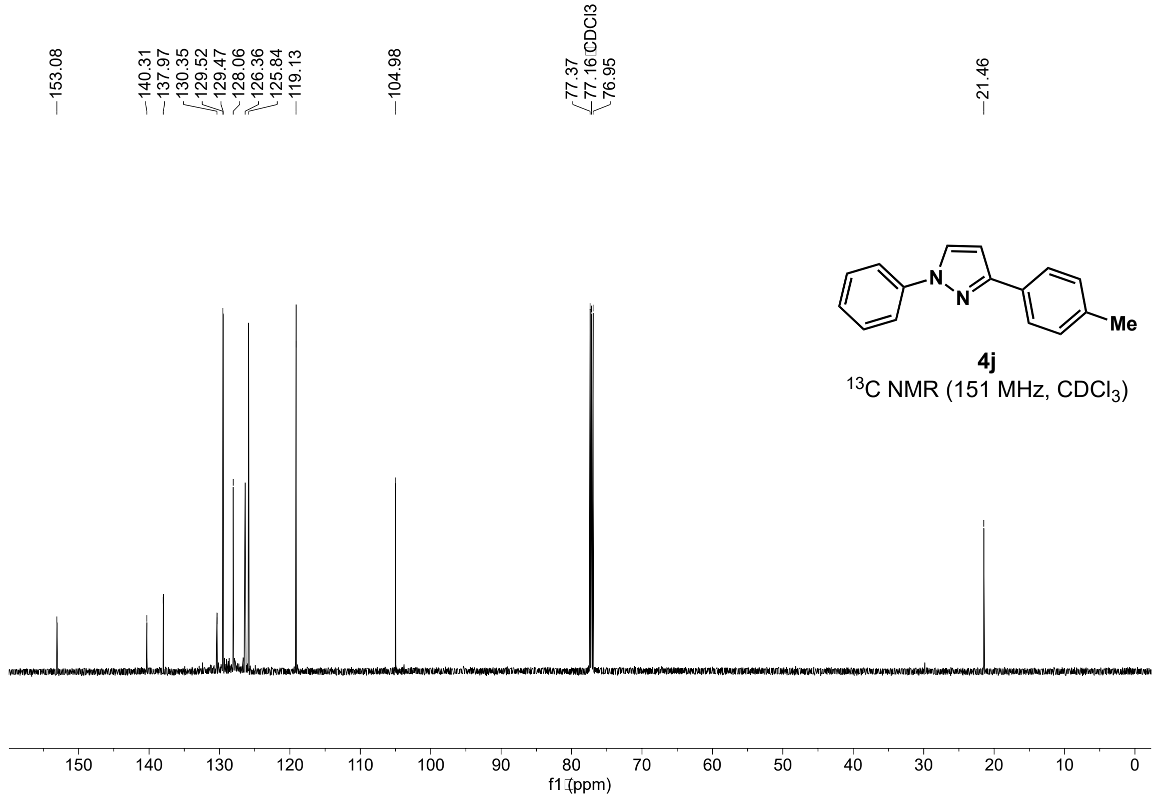

3-(4-Chlorophenyl)-1-phenyl-1*H*-pyrazole (4k)

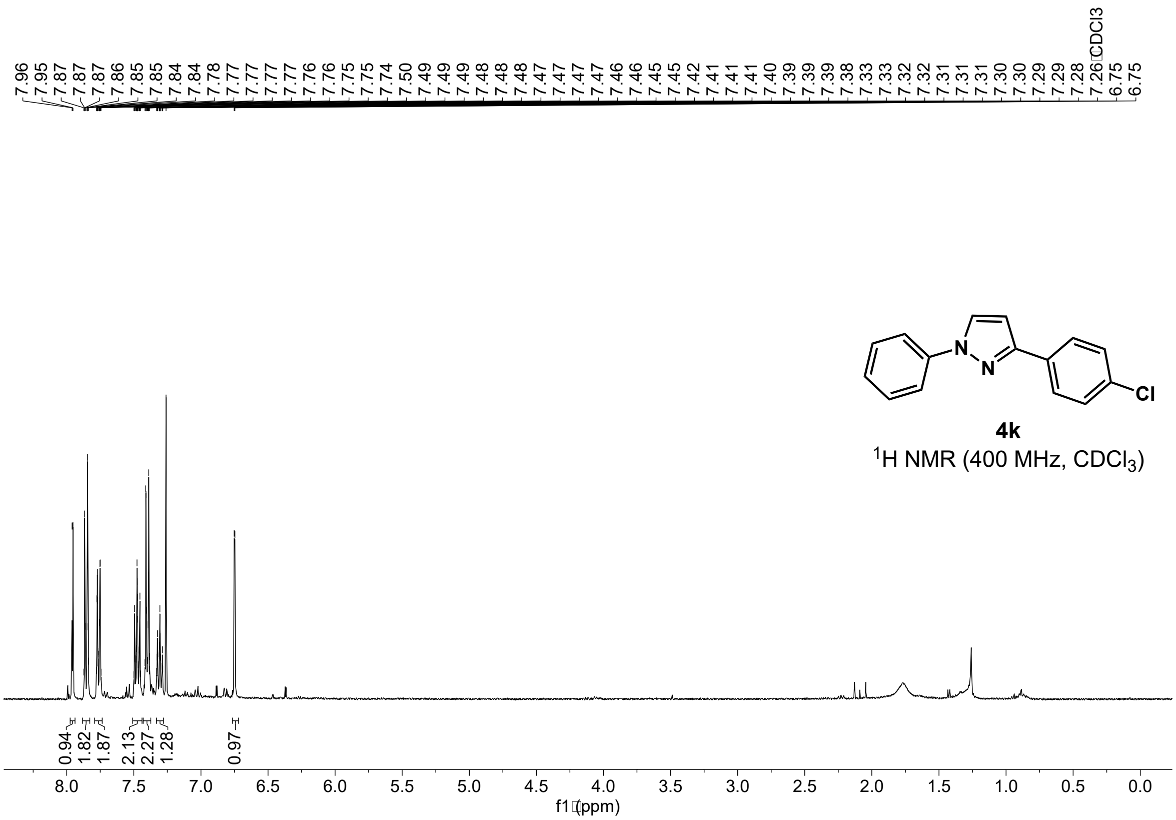

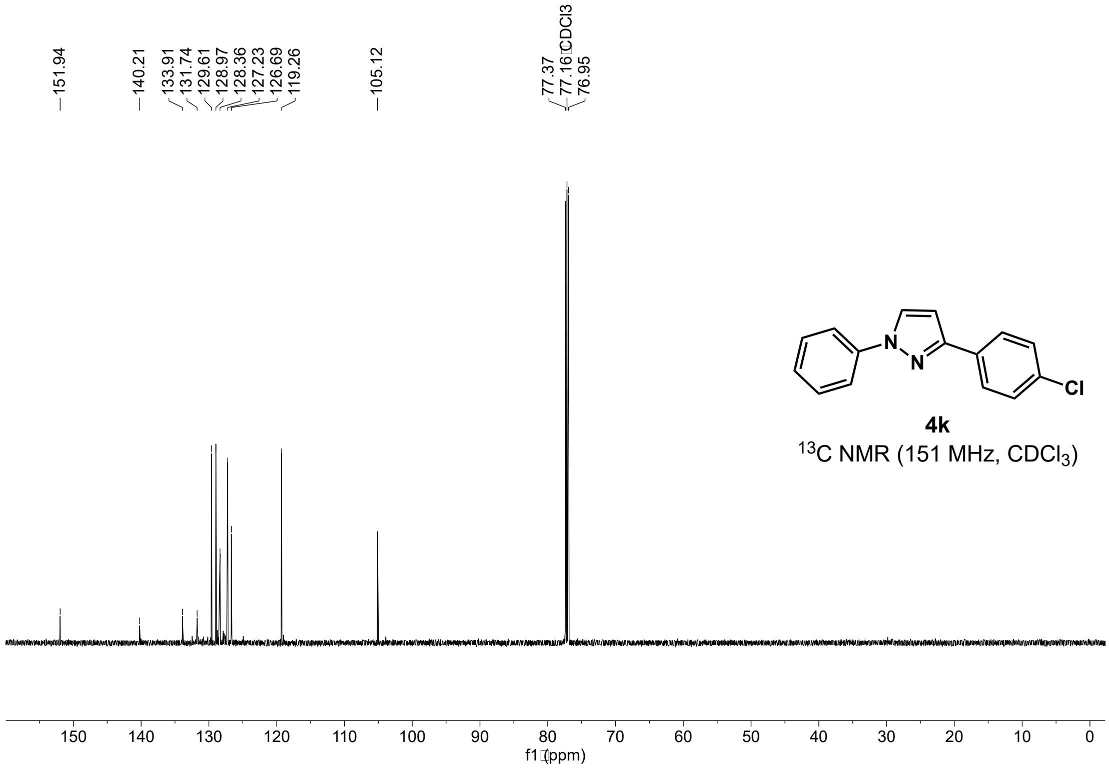

3-(4-Fluorophenyl)-1-phenyl-1*H*-pyrazole (4l)

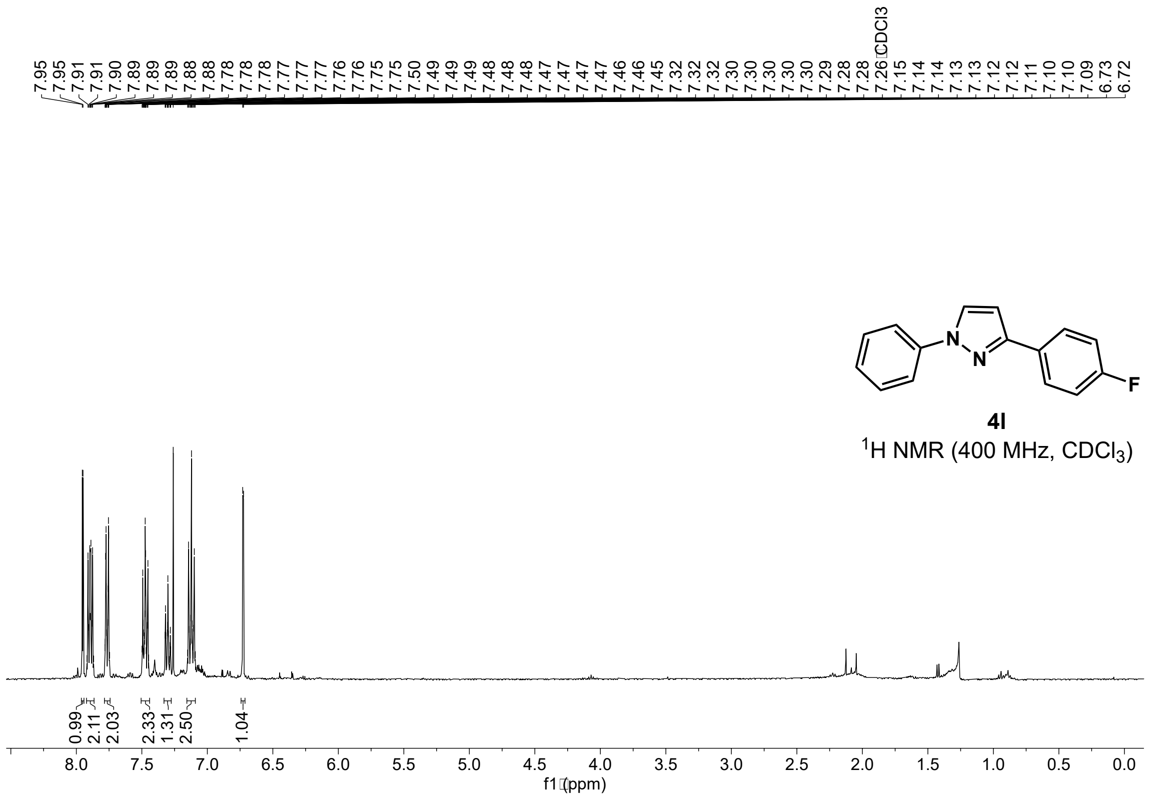

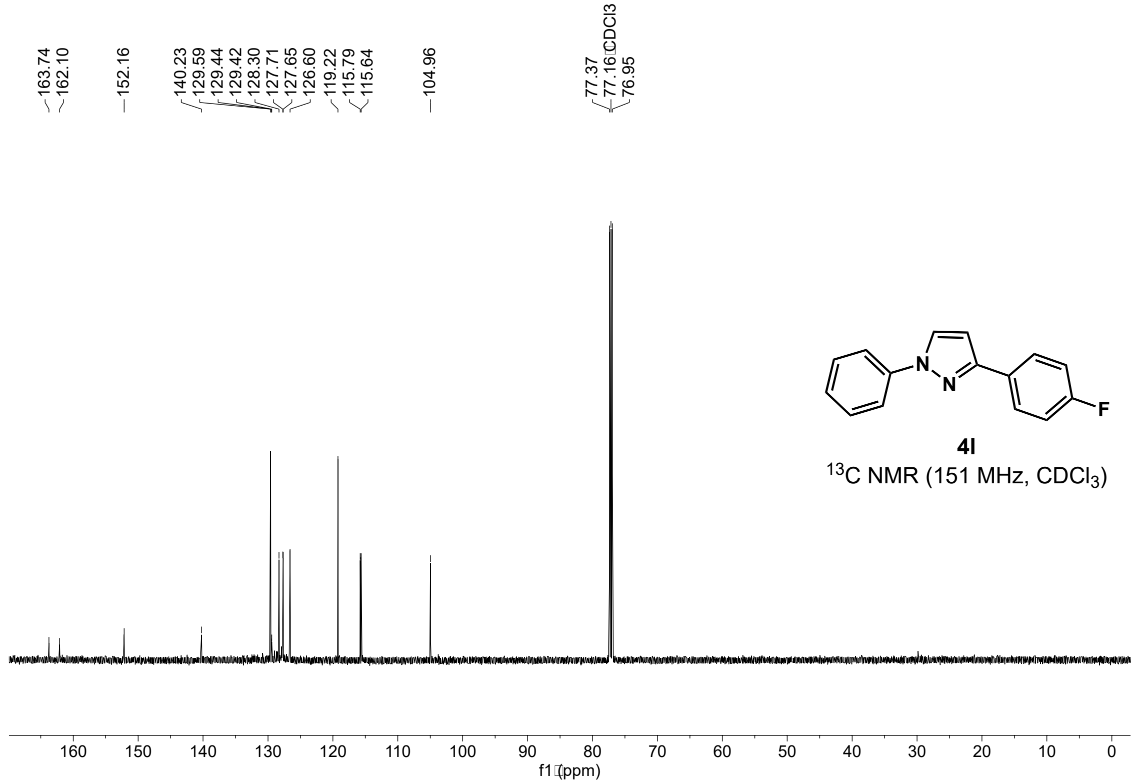

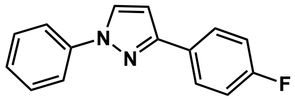

4l

^{19}F NMR (376 MHz, $CDCl_3$)

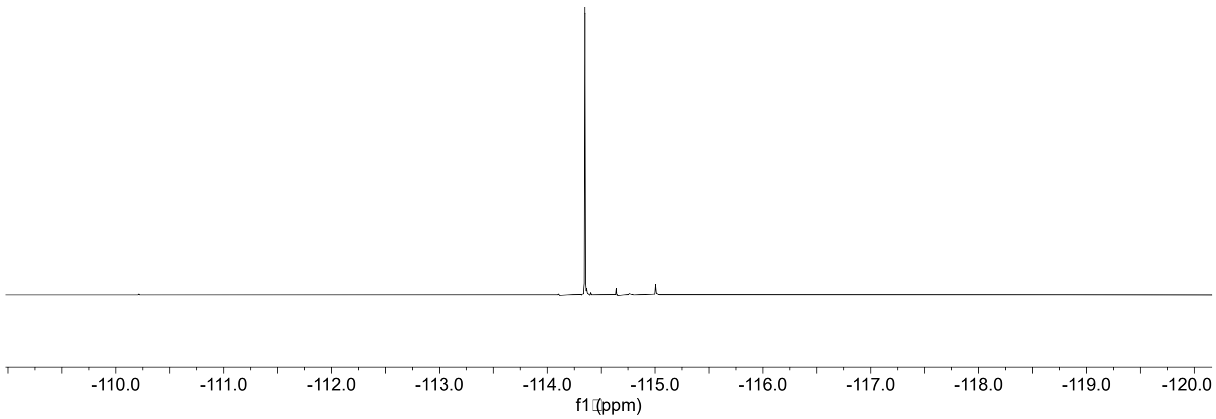

3-(4-Methoxyphenyl)-1-phenyl-1*H*-pyrazole (4m)

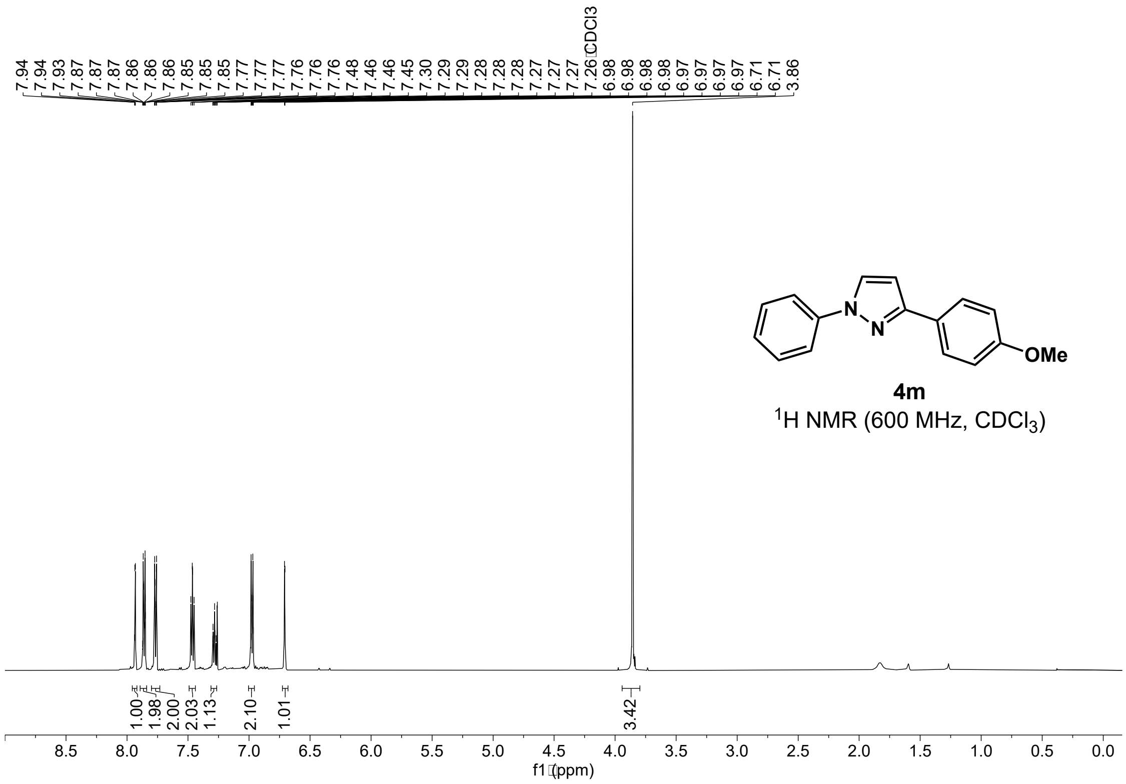

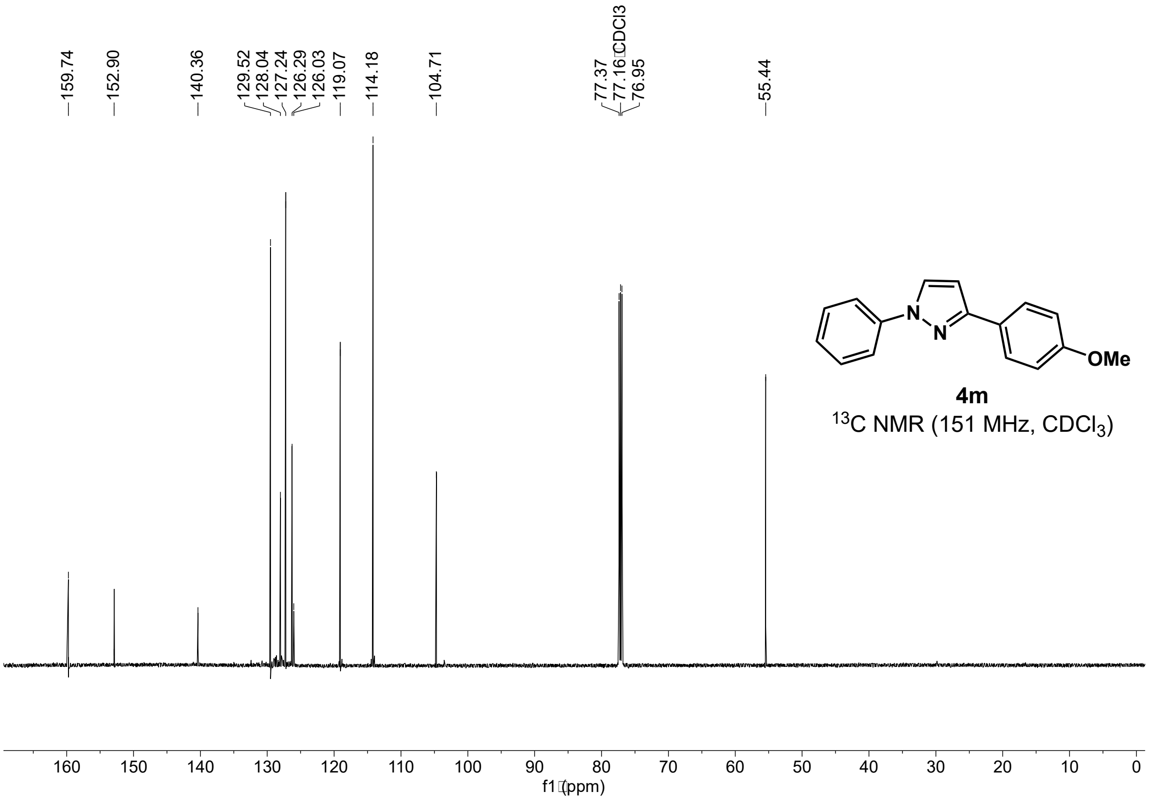

3-(3-Methoxyphenyl)-1-phenyl-1H-pyrazole (4n)

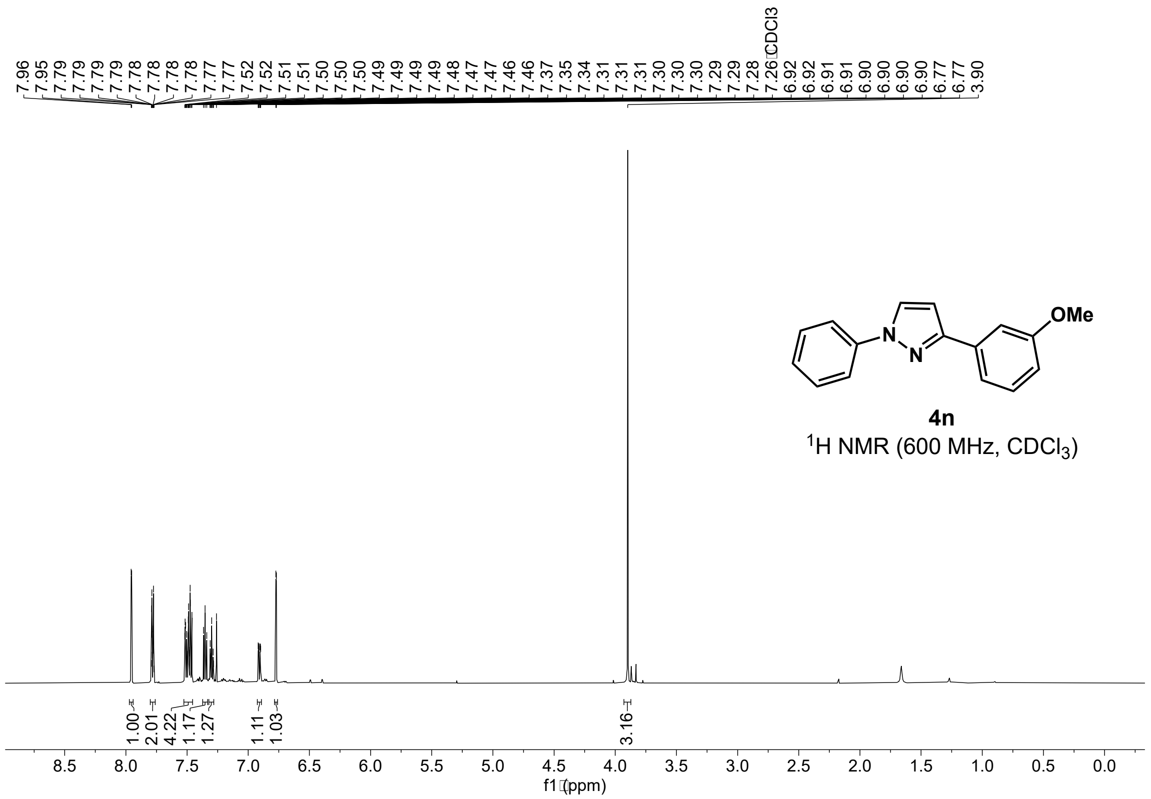

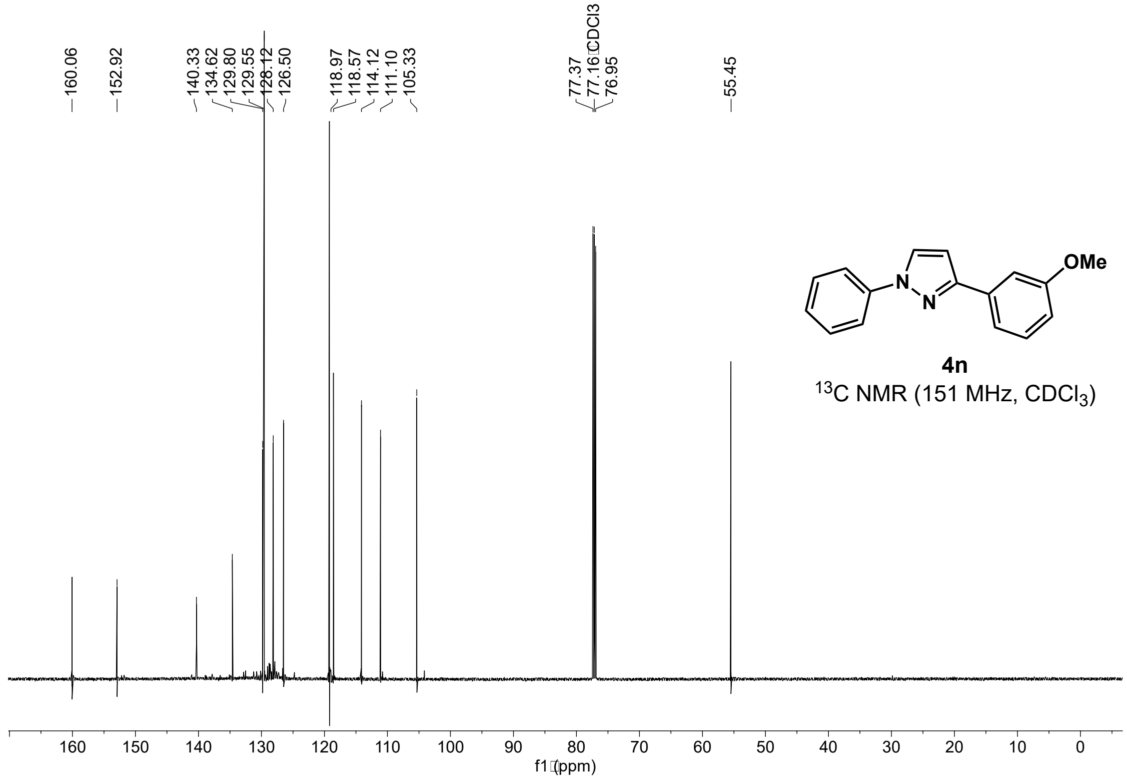

3-(Furan-2-yl)-1-phenyl-1*H*-pyrazole (4o)

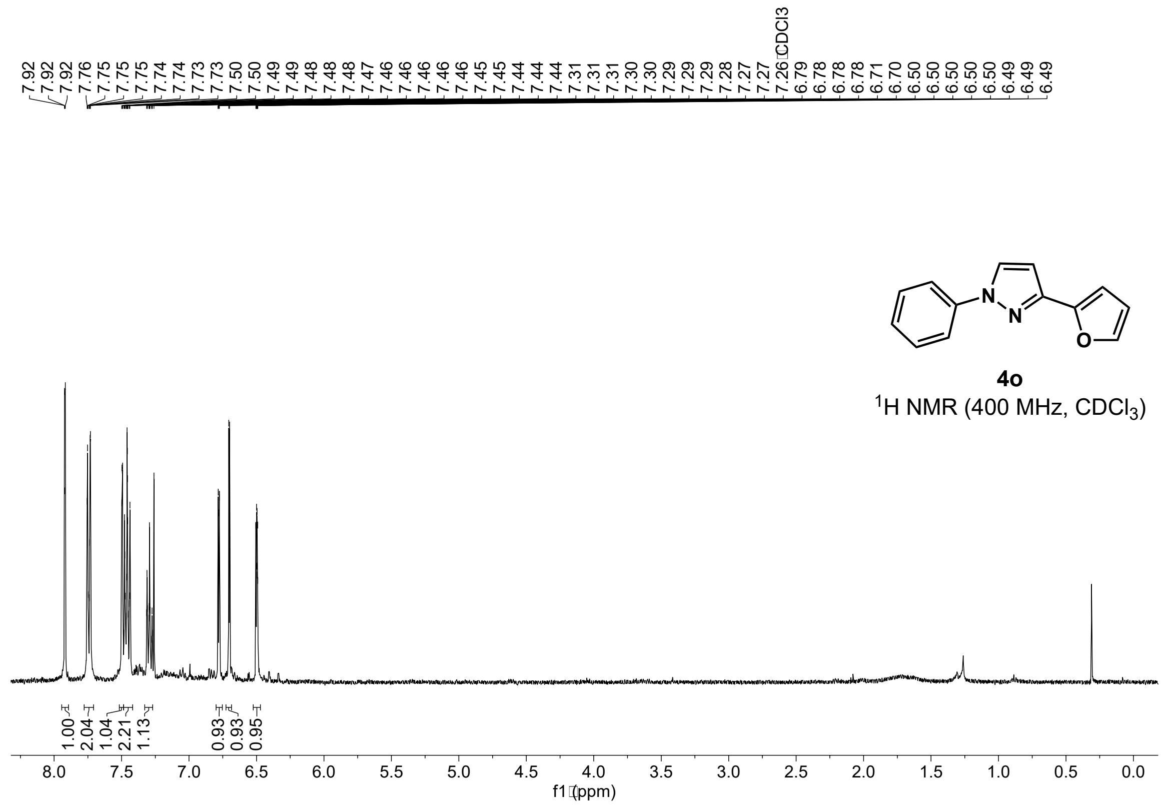

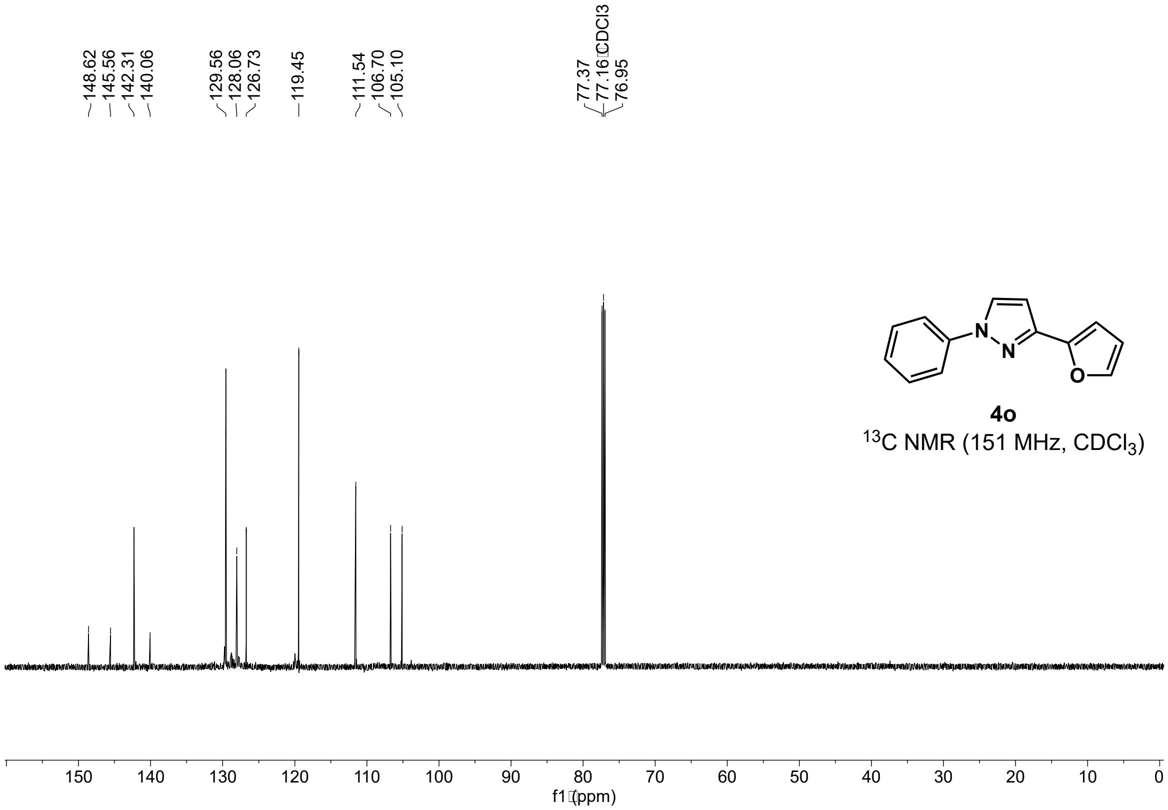

5-Methyl-1,3-diphenyl-1*H*-pyrazole (4p)

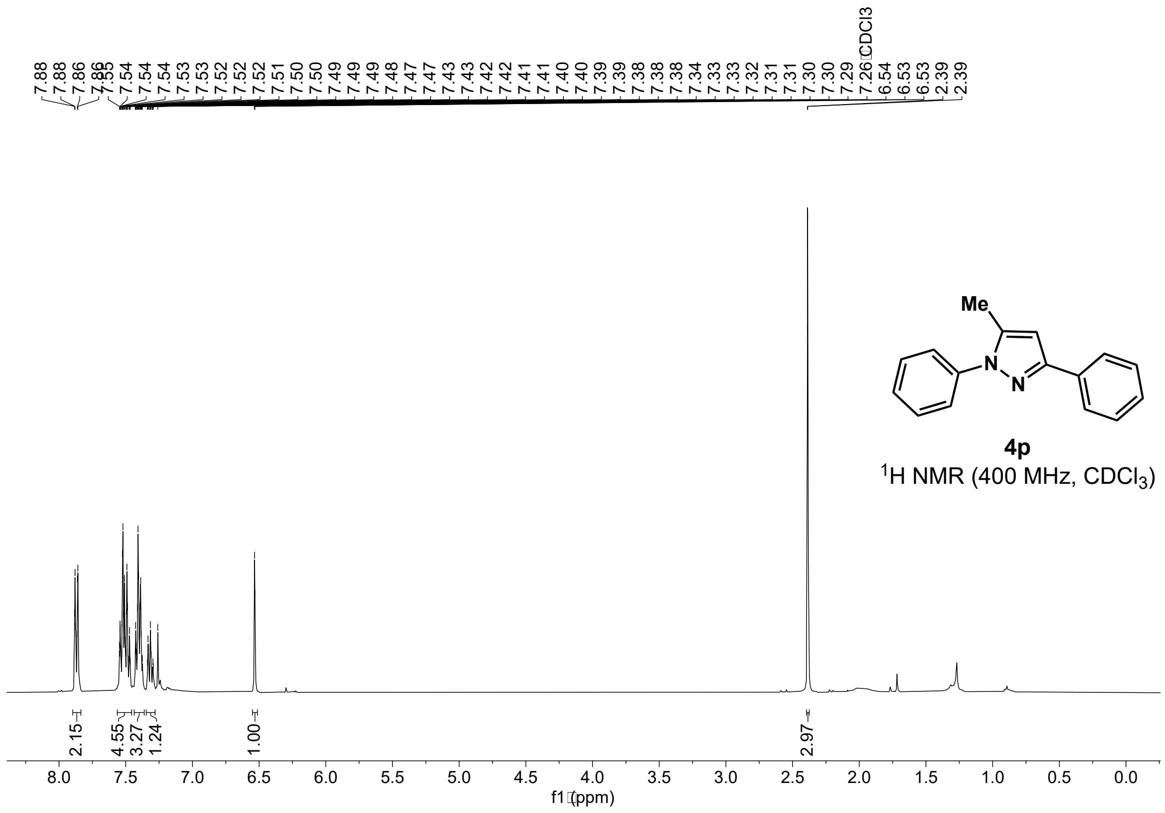

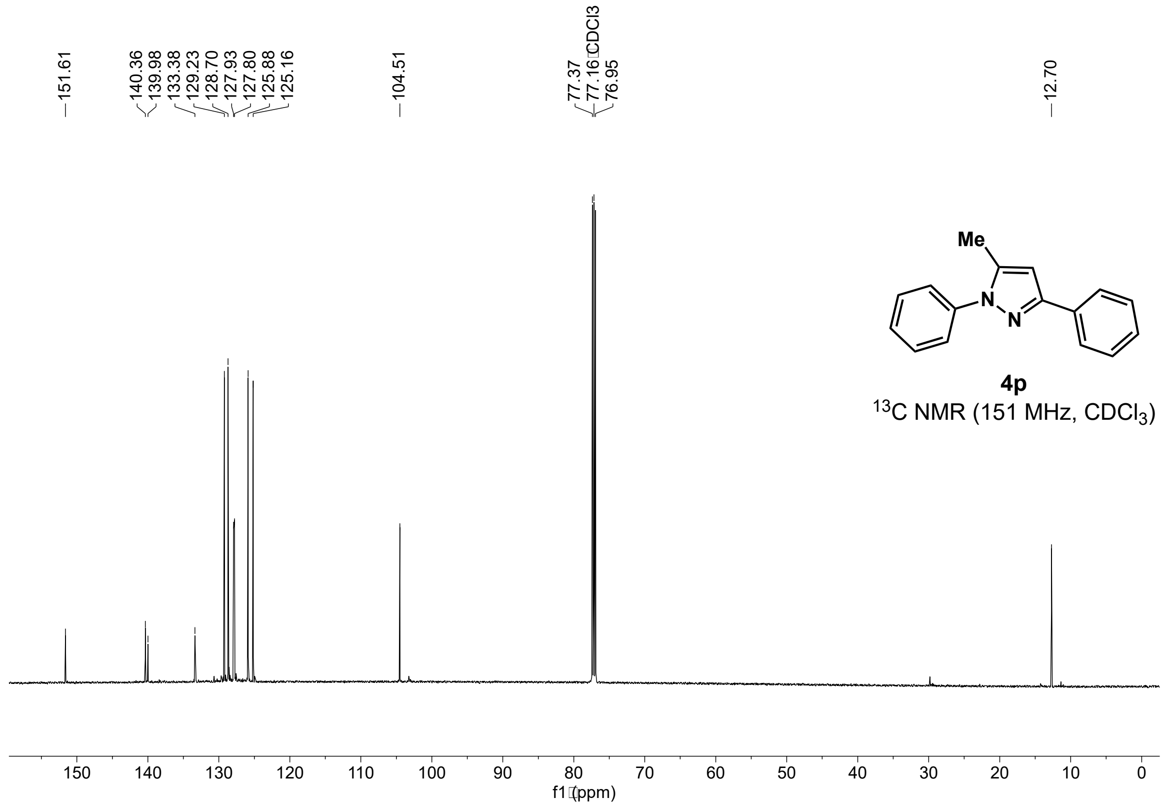

3-(Tert-butyl)-5-methyl-1-phenyl-1*H*-pyrazole (4q)

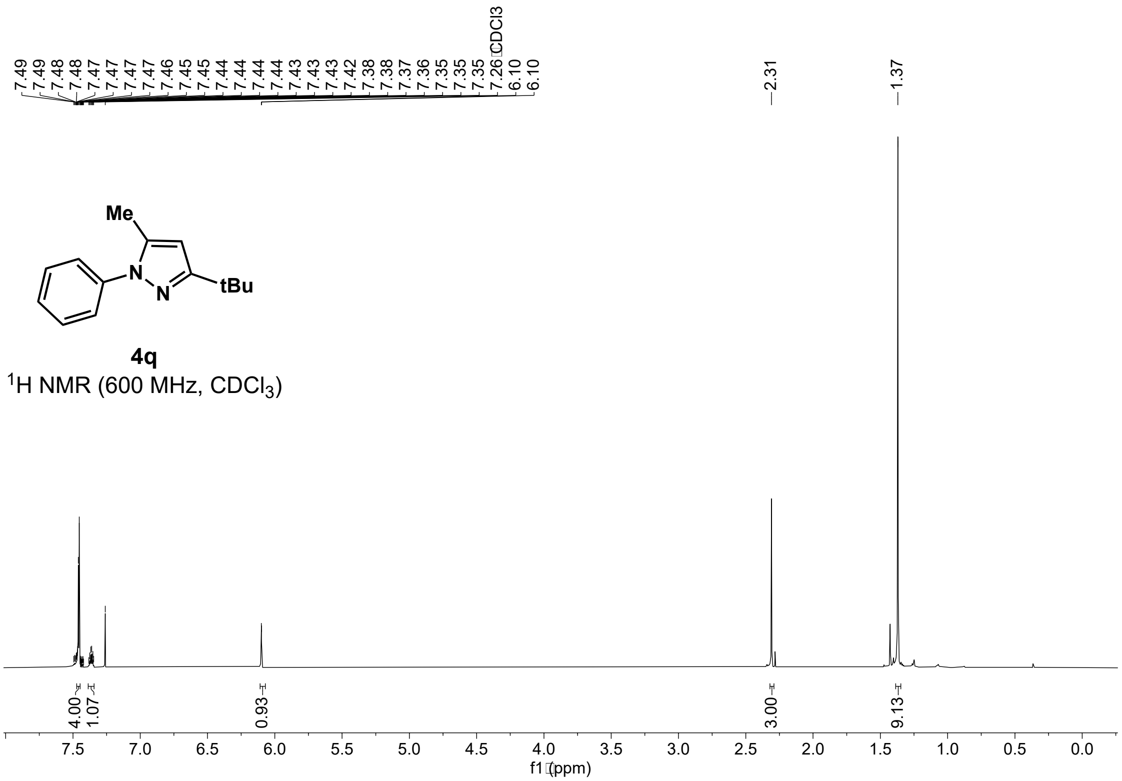

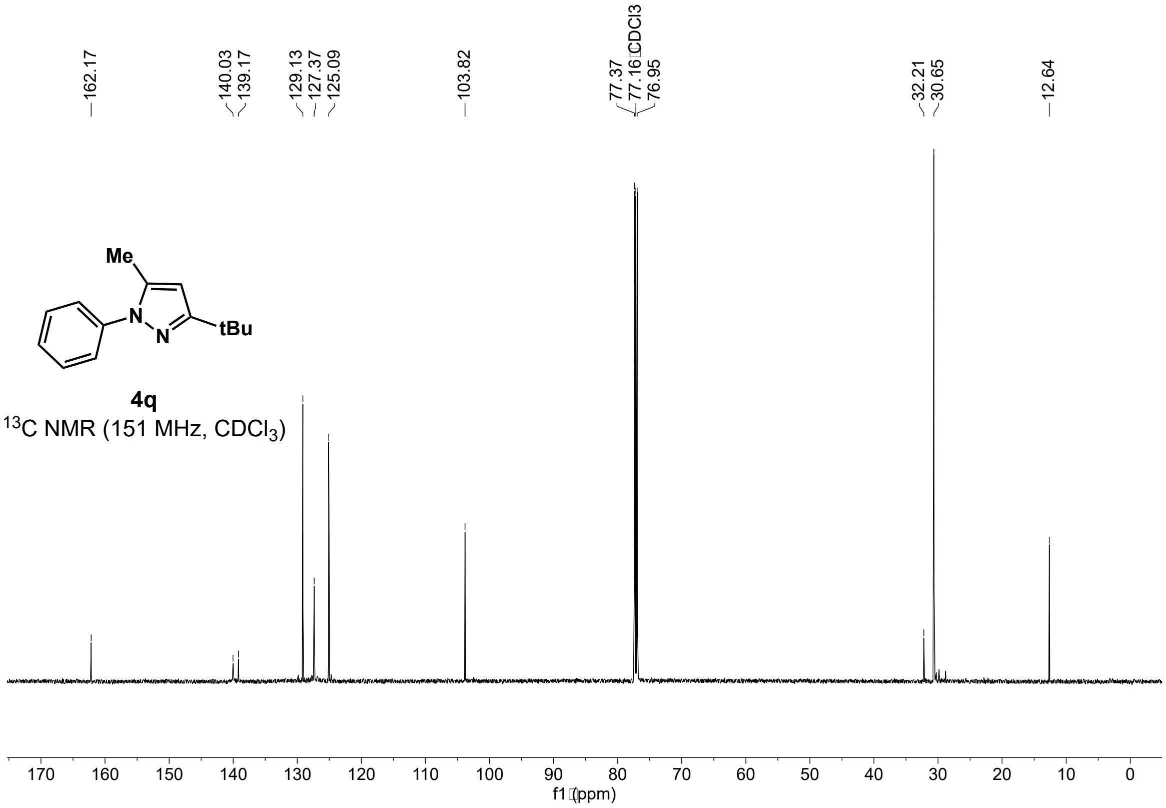

1,3-Diphenyl-1*H*-pyrazol-5-amine (4r)

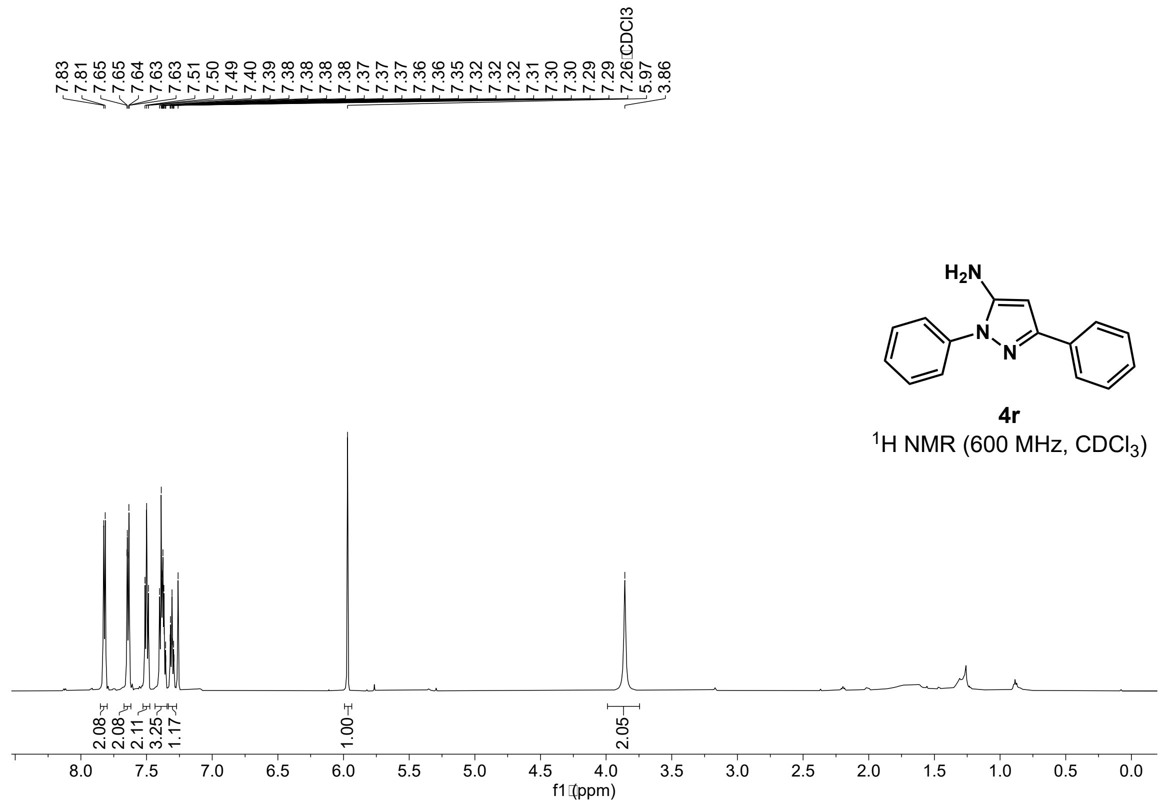

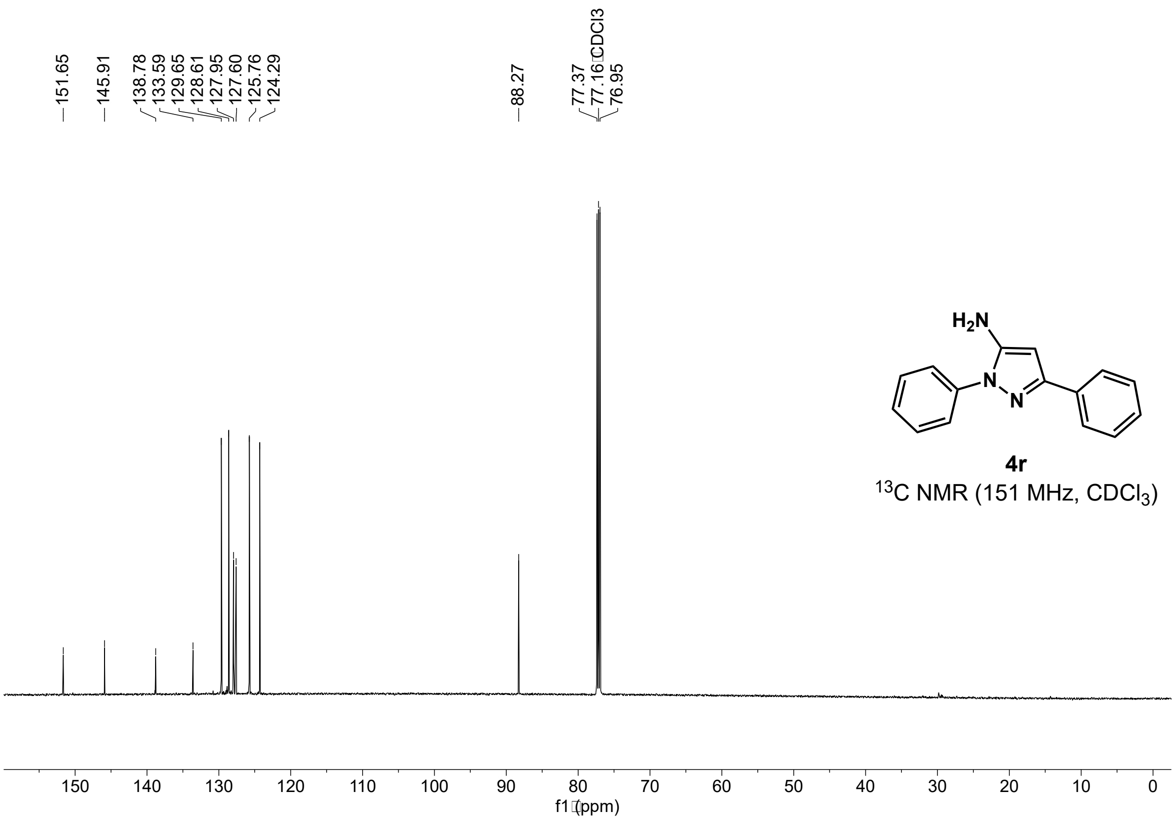

Ethyl 3-cyclopropyl-1-phenyl-1*H*-pyrazole-4-carboxylate (4s)

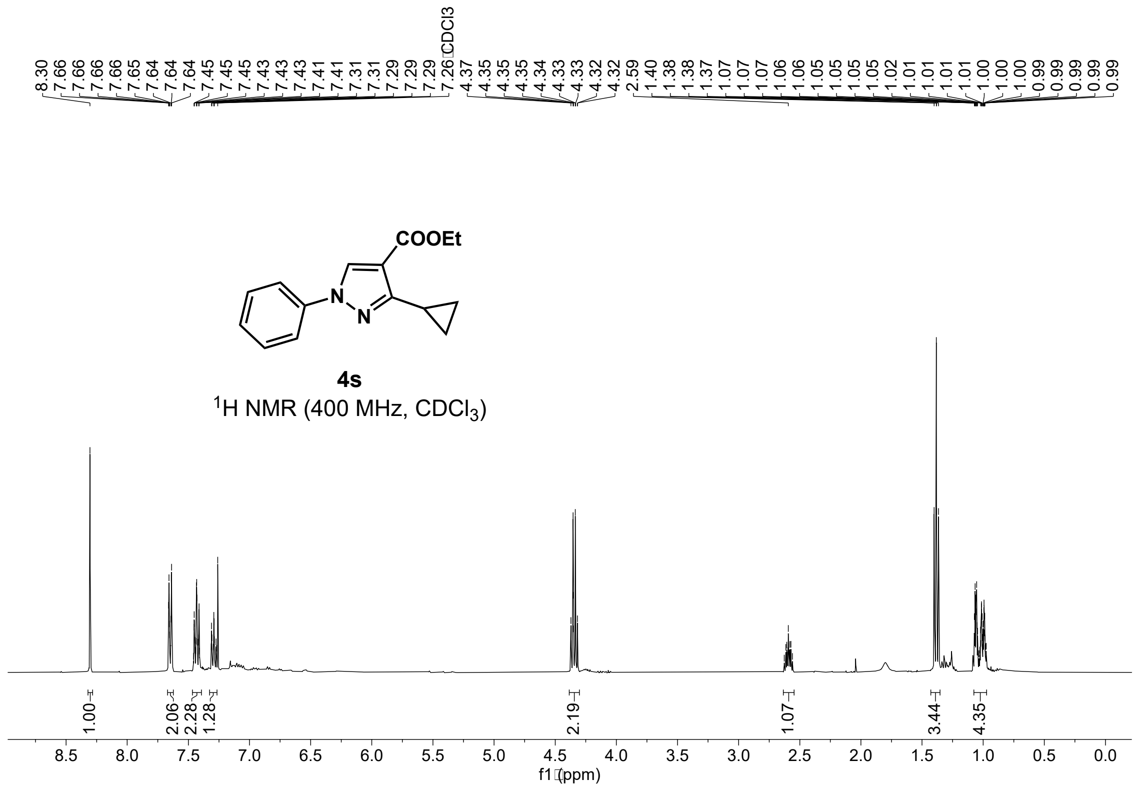

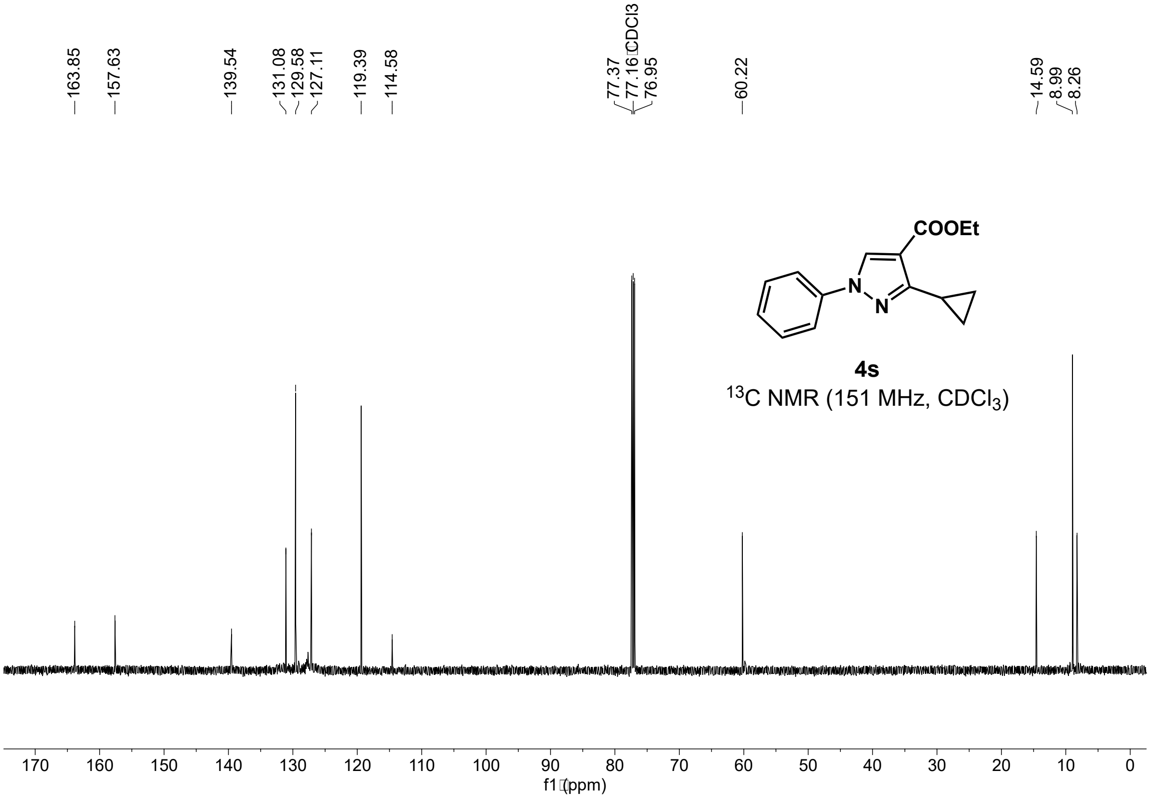

1-Phenyl-1*H*-indazole (4t)

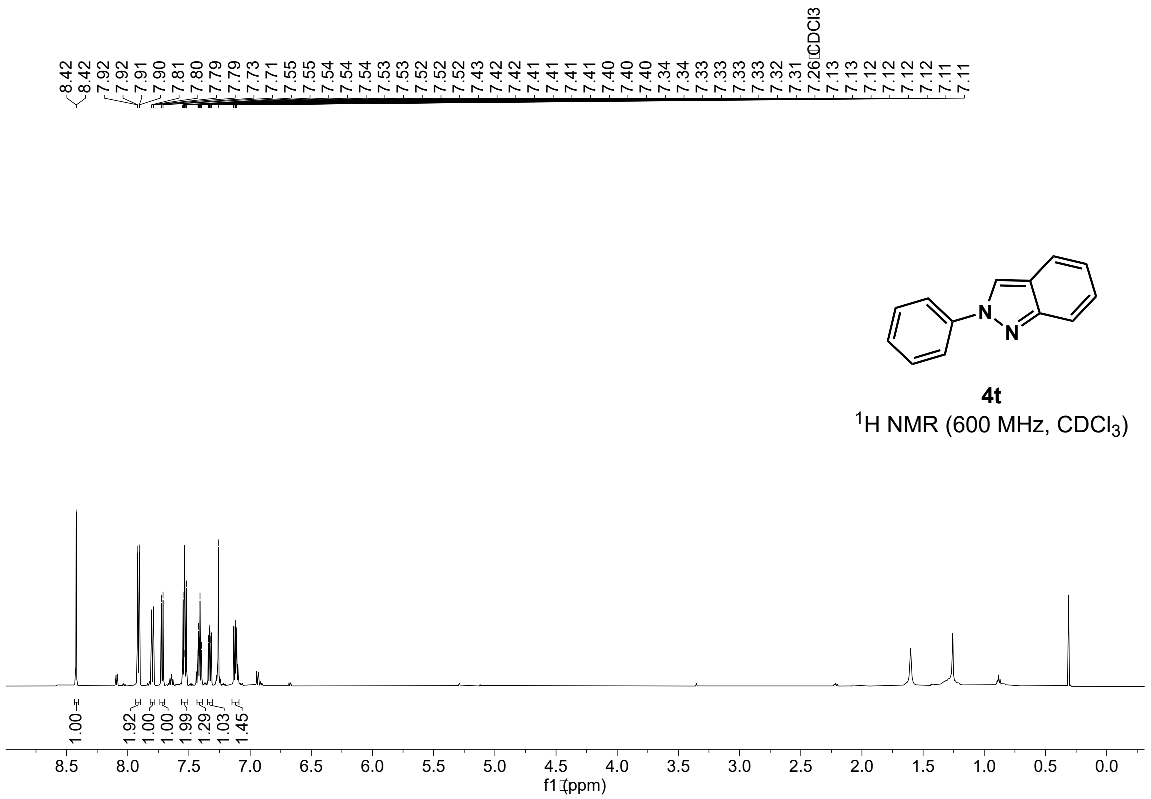

4t
^1H NMR (600 MHz, $CDCl_3$)

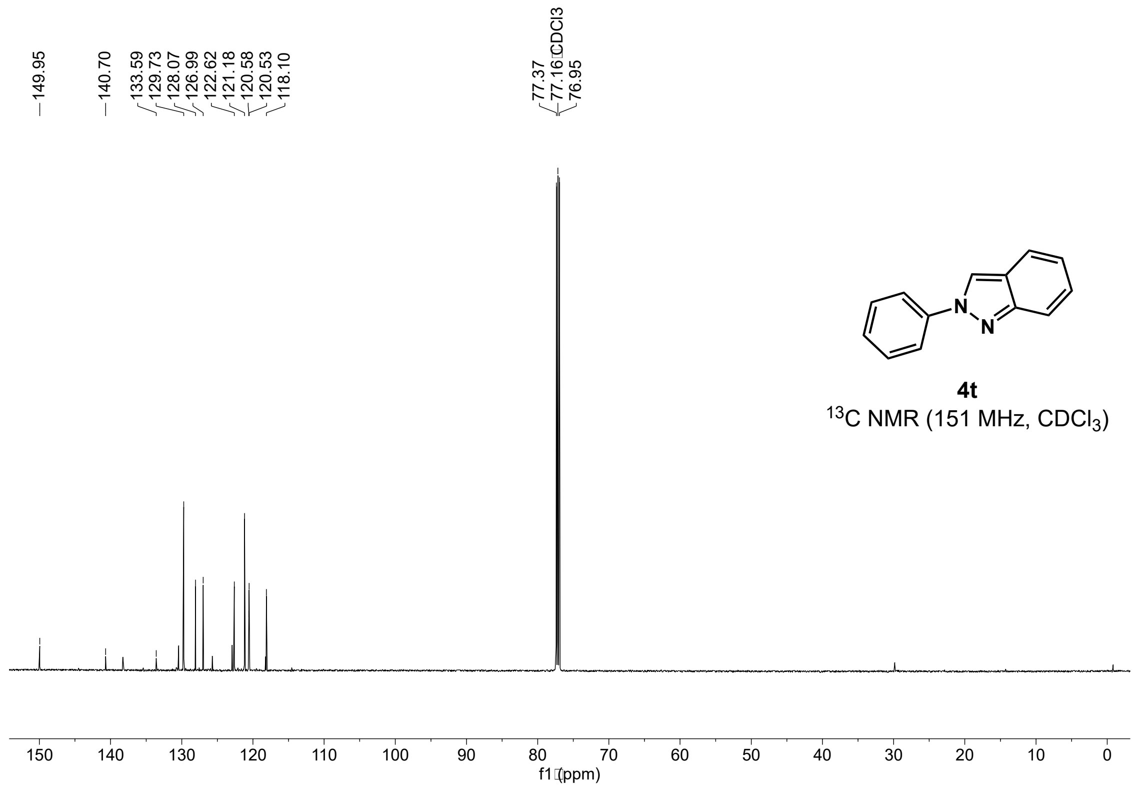

4t
^{13}C NMR (151 MHz, $CDCl_3$)

1-(3,4-Dimethylphenyl)-5-phenyl-1*H*-pyrazole (5a)

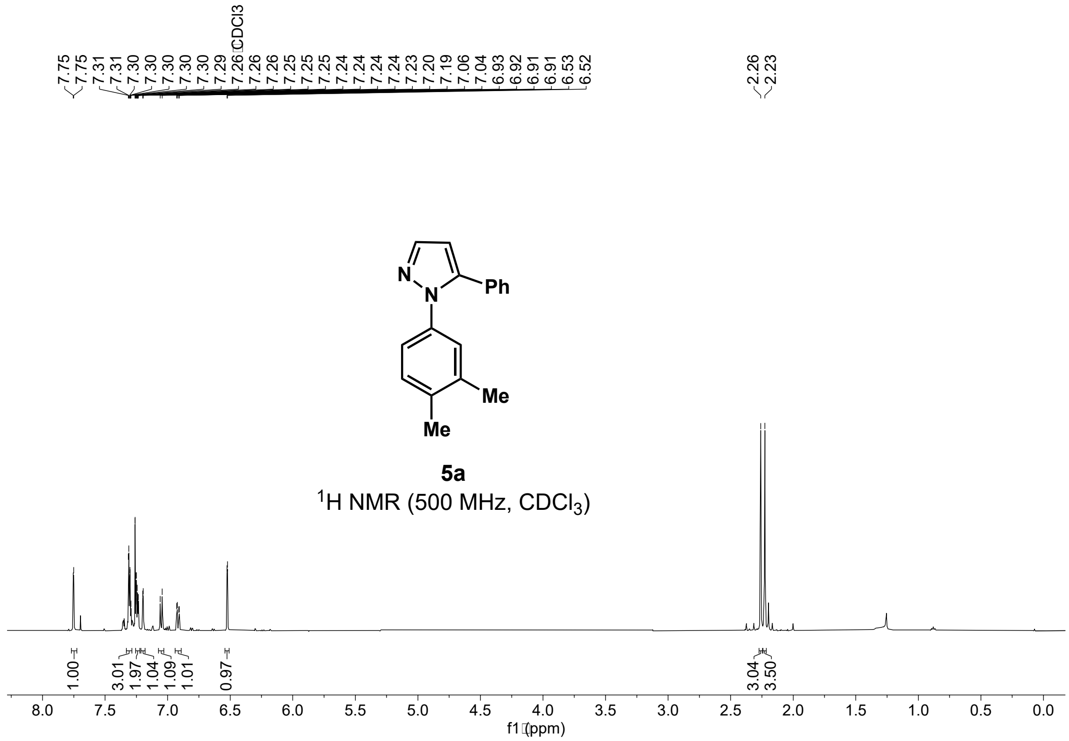

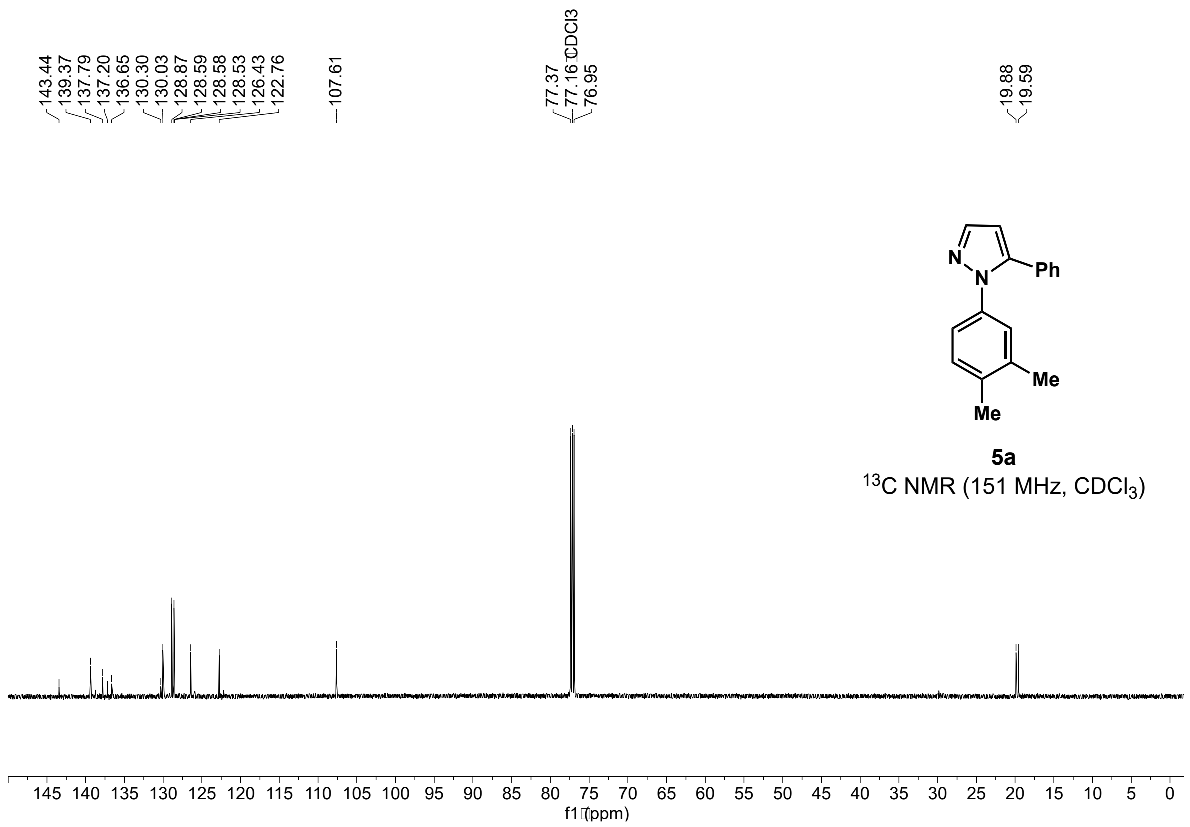

1-(3,4-Dimethoxyphenyl)-5-phenyl-1*H*-pyrazole (5b)

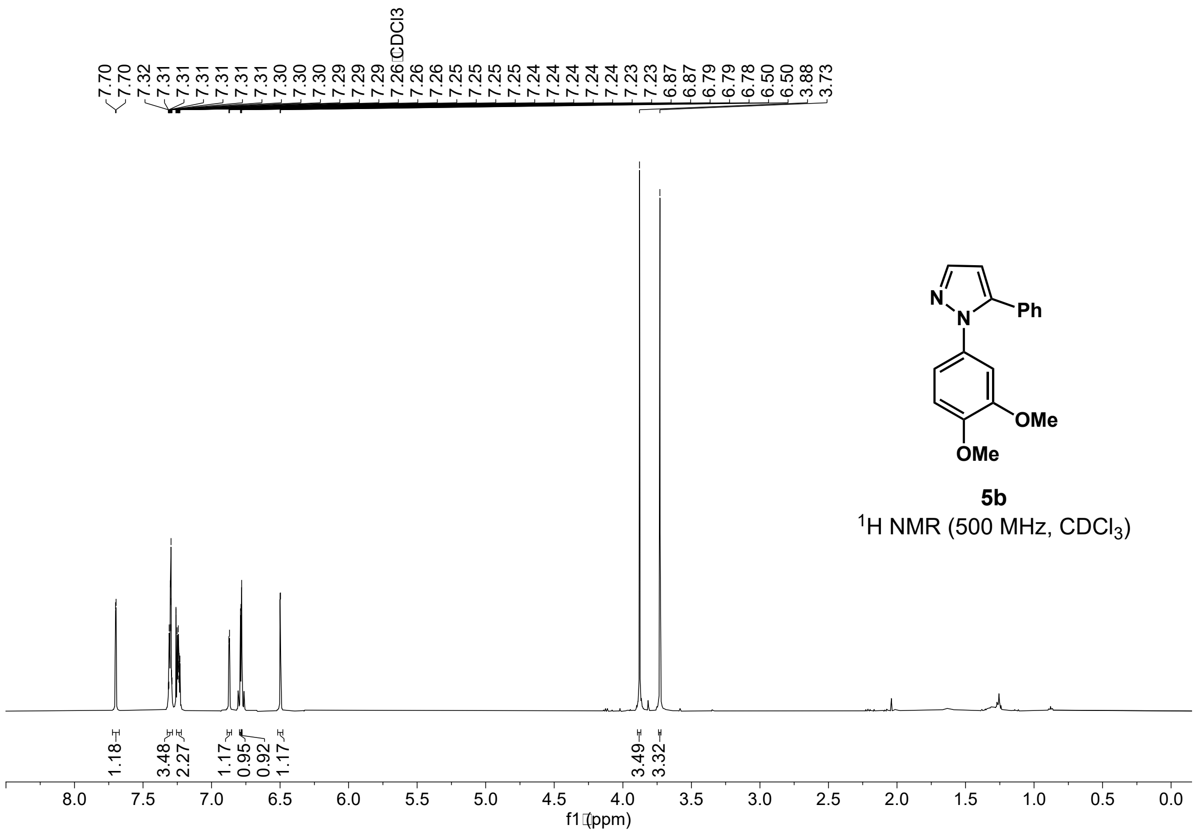

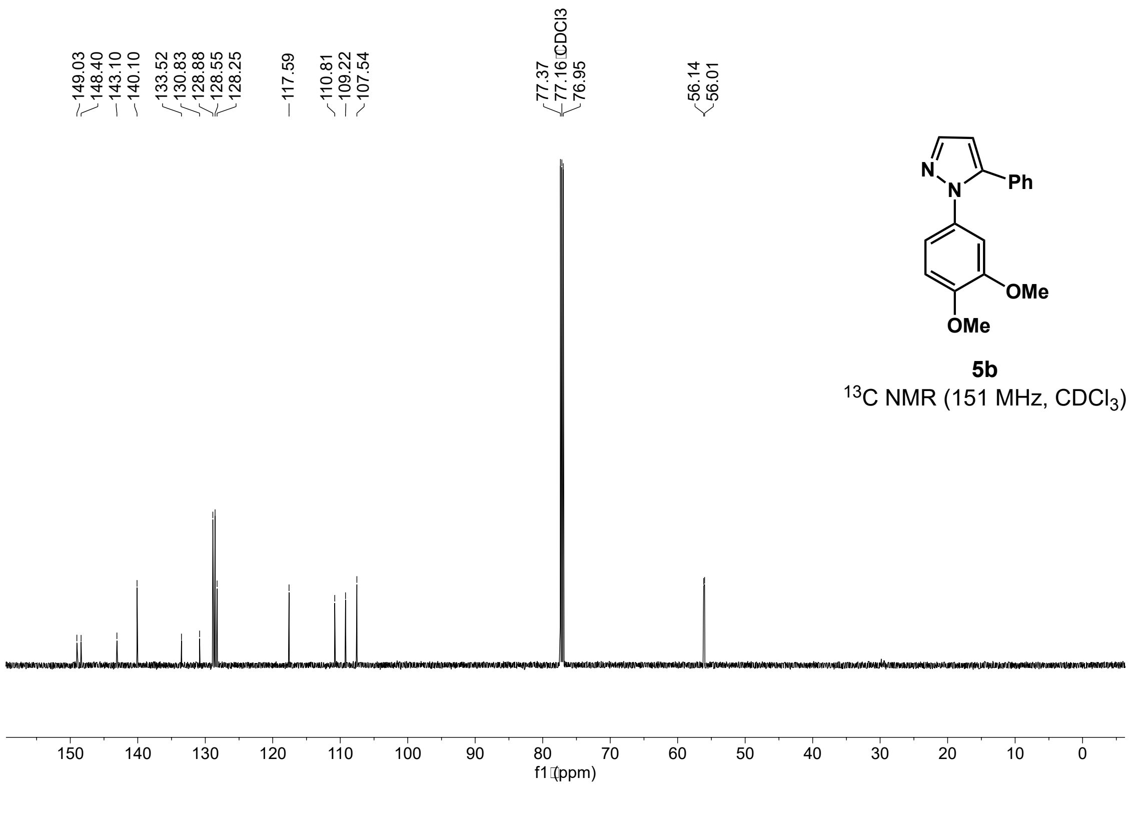

1-(Naphthalen-2-yl)-5-phenyl-1*H*-pyrazole (5c)

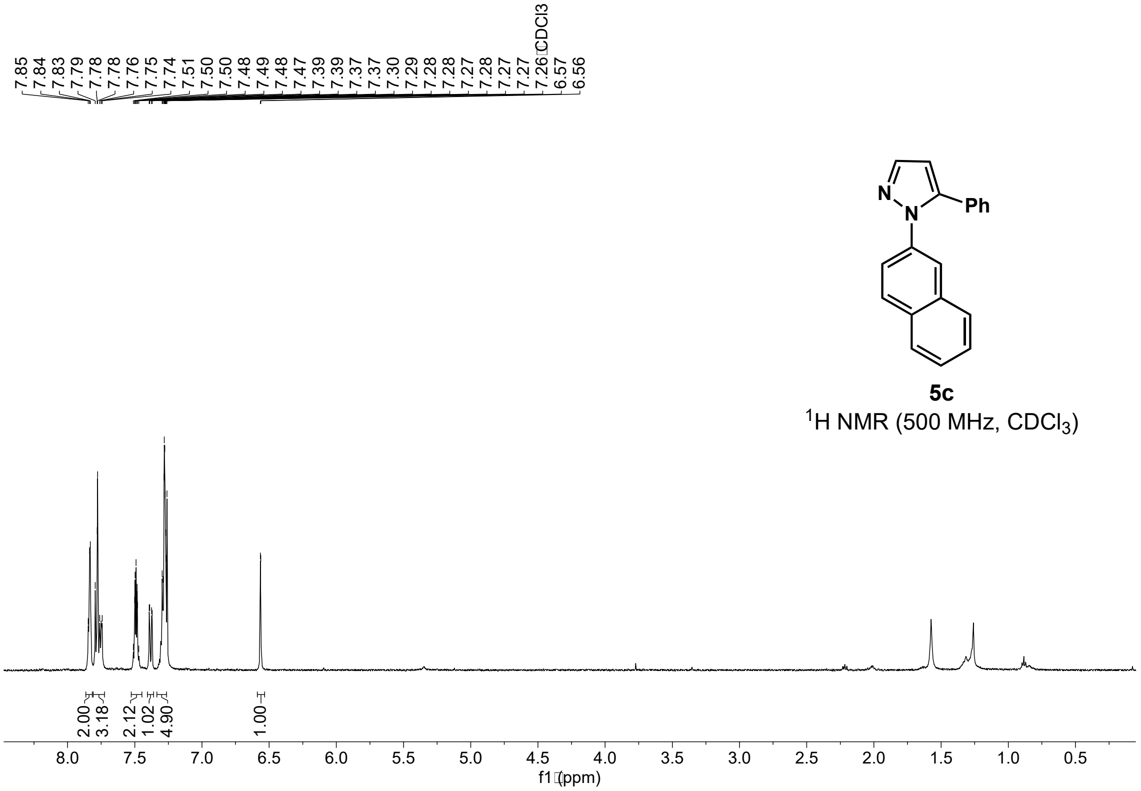

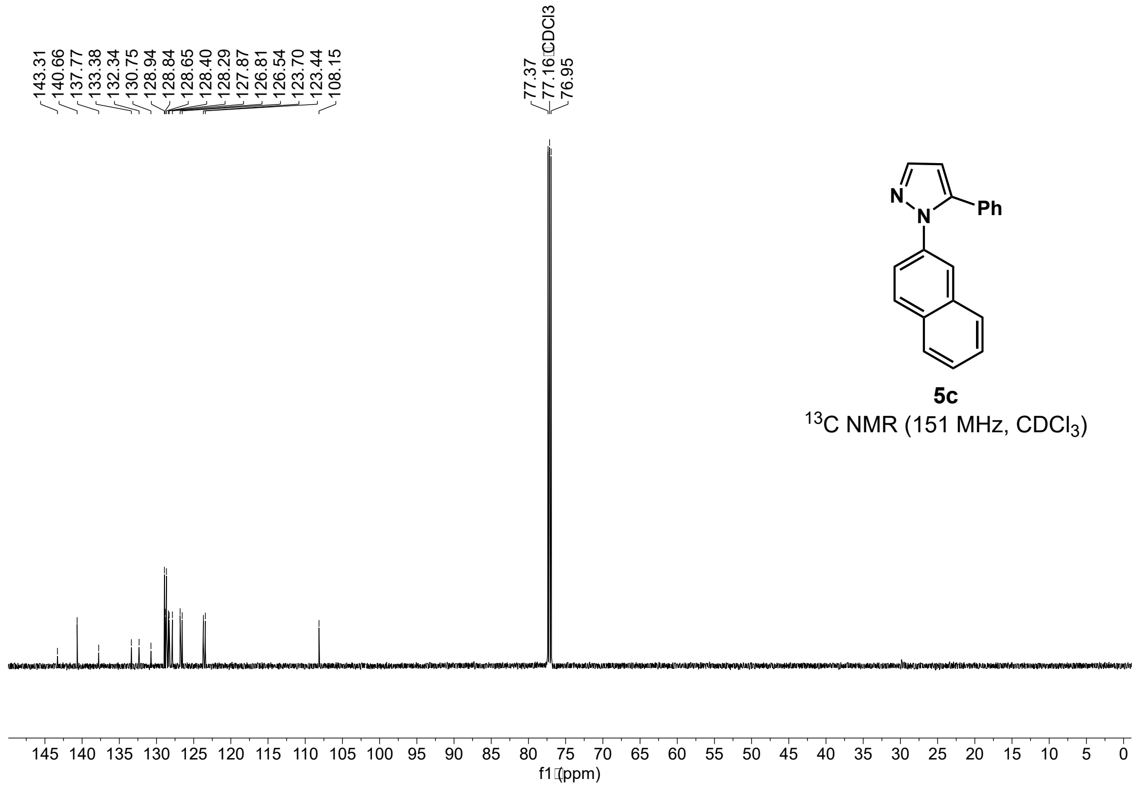

5-Phenyl-1-(p-tolyl)-1*H*-pyrazole (5d)

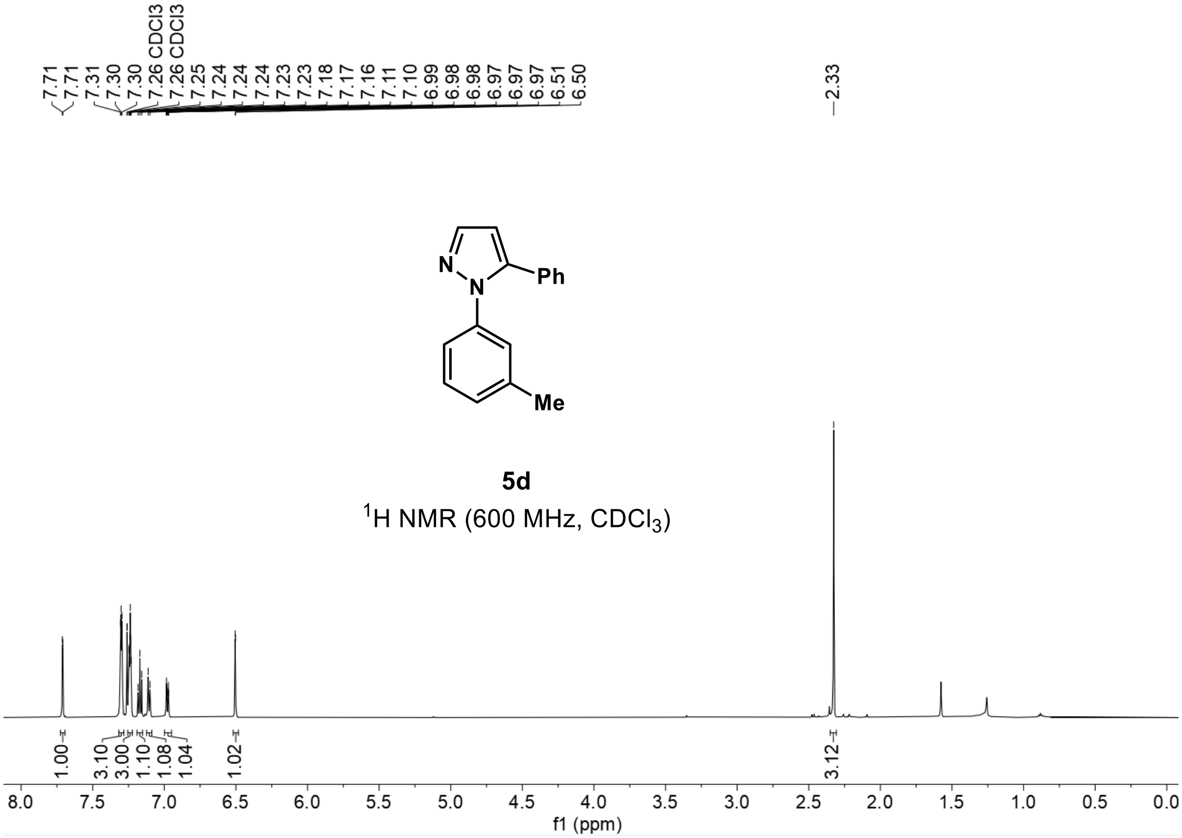

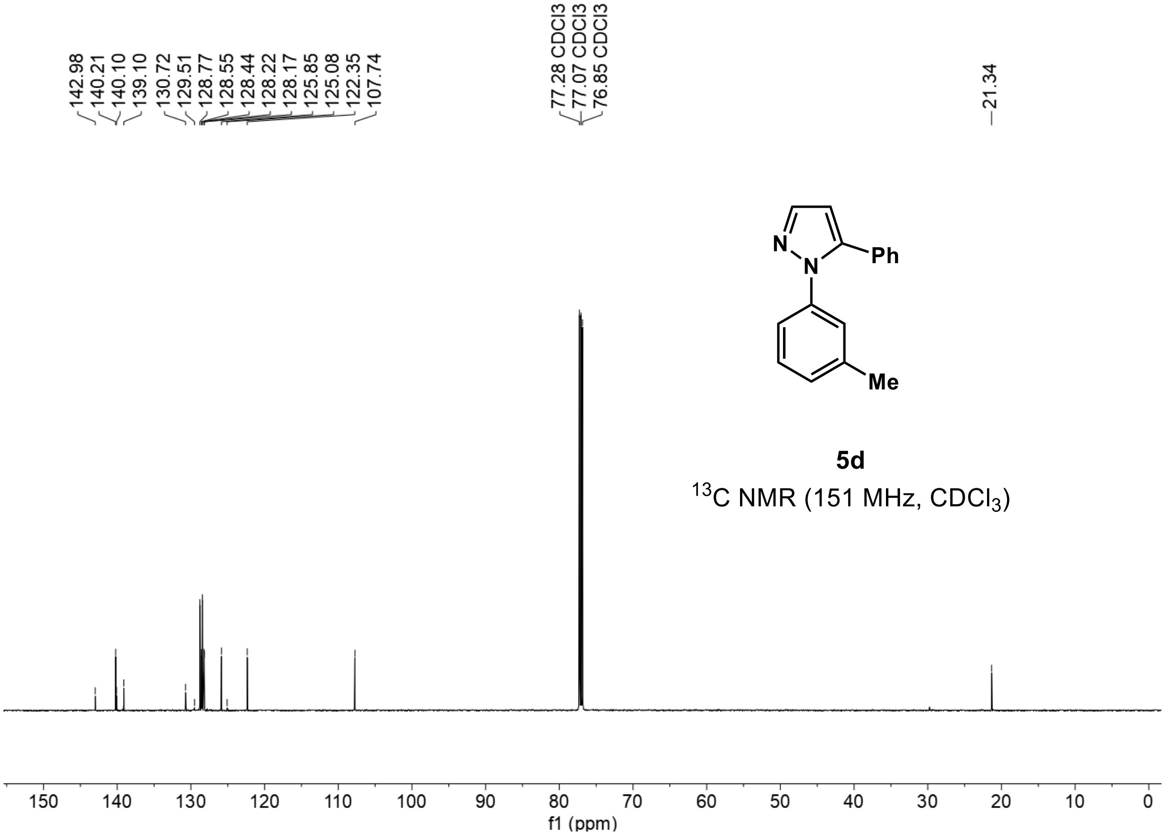

1-(4-Chlorophenyl)-5-phenyl-1H-pyrazole (5e)

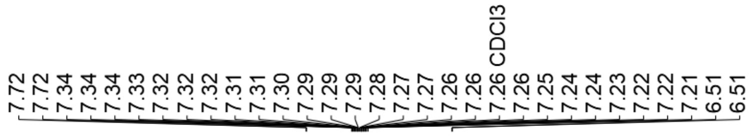

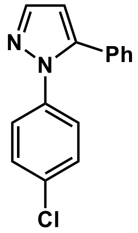

5e

^{1}H NMR (600 MHz, $CDCl_3$)

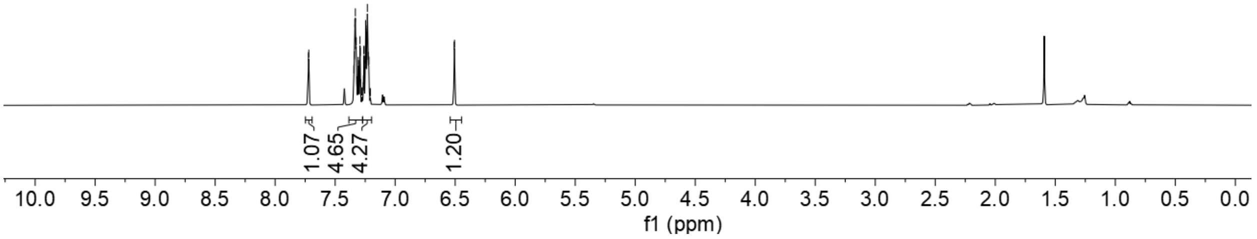

5-Phenyl-1-(m-tolyl)-1*H*-pyrazole (5f)

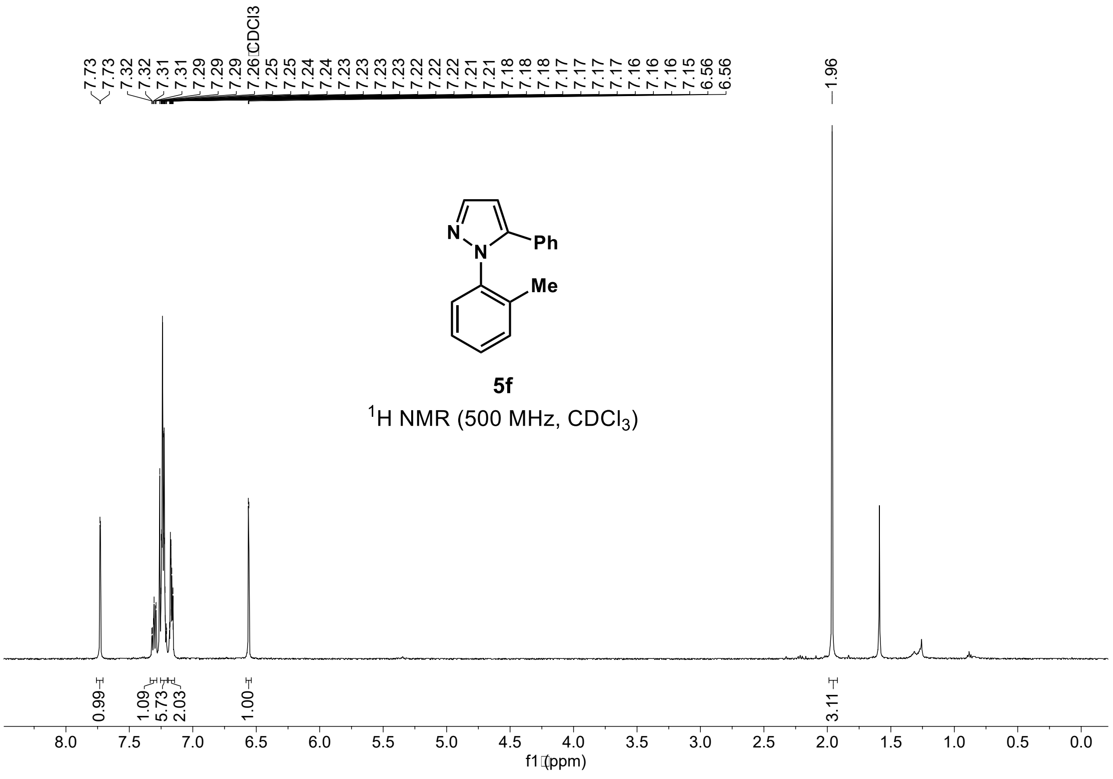

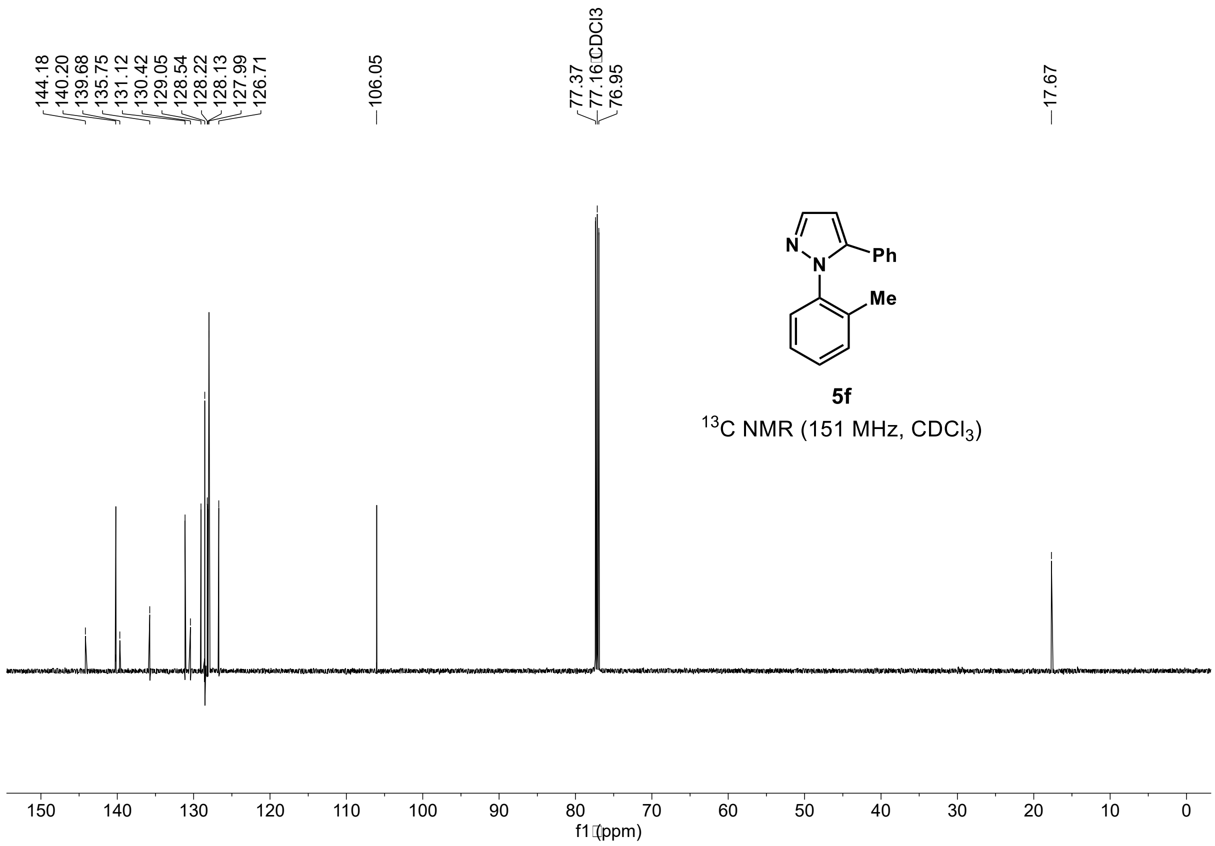

5-Phenyl-1-(2-(trimethylsilyl)phenyl)-1*H*-pyrazole (5g)

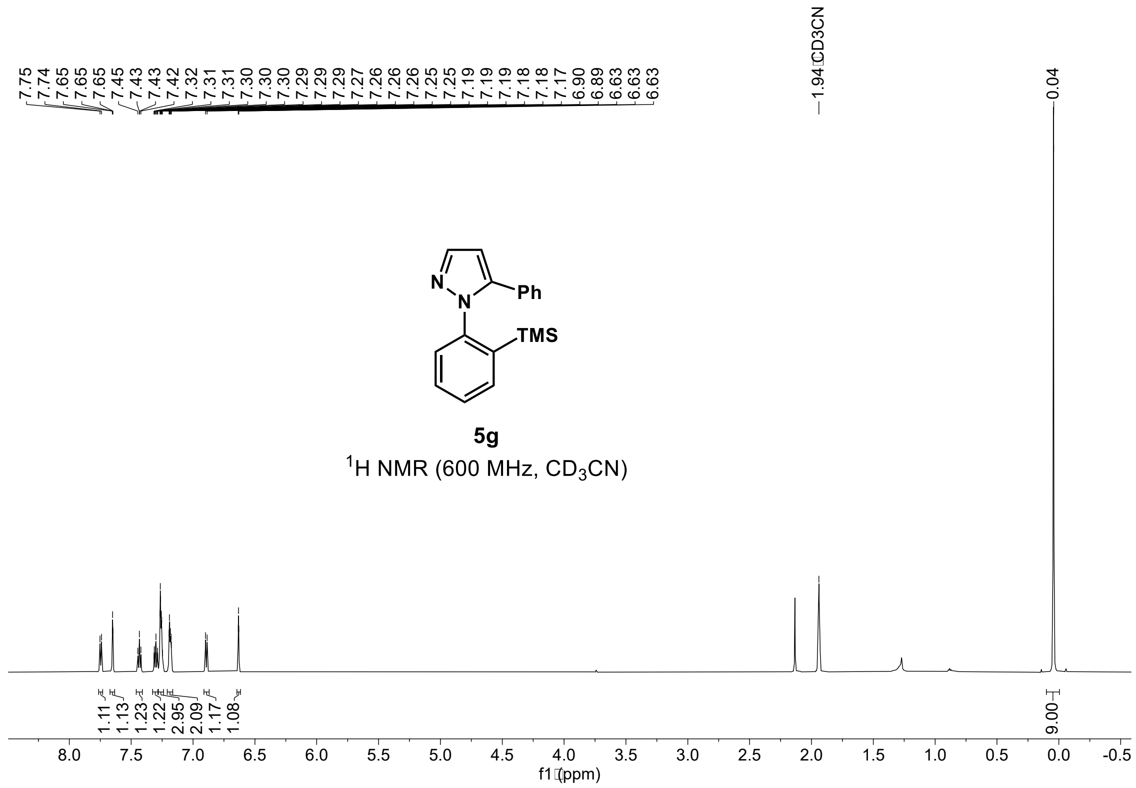

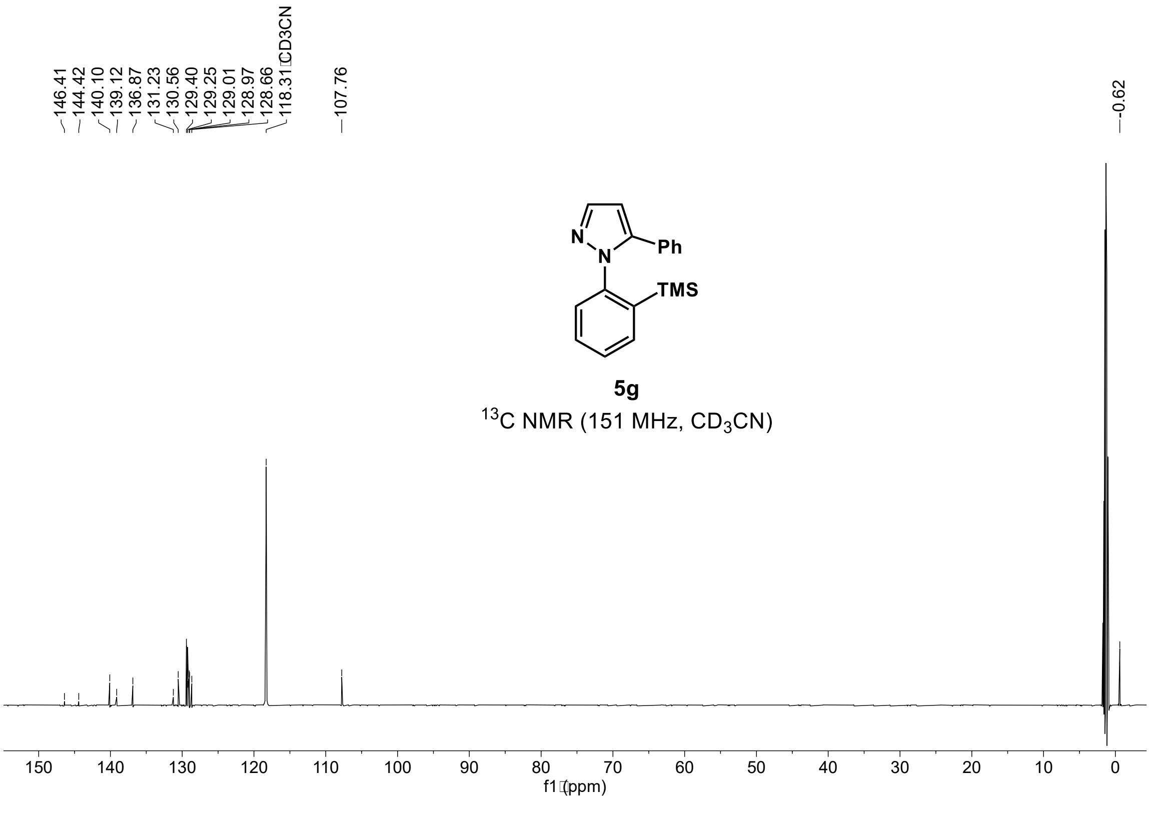

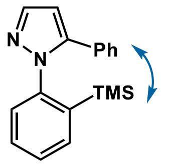

5g
NOE (600 MHz, CD_3CN)

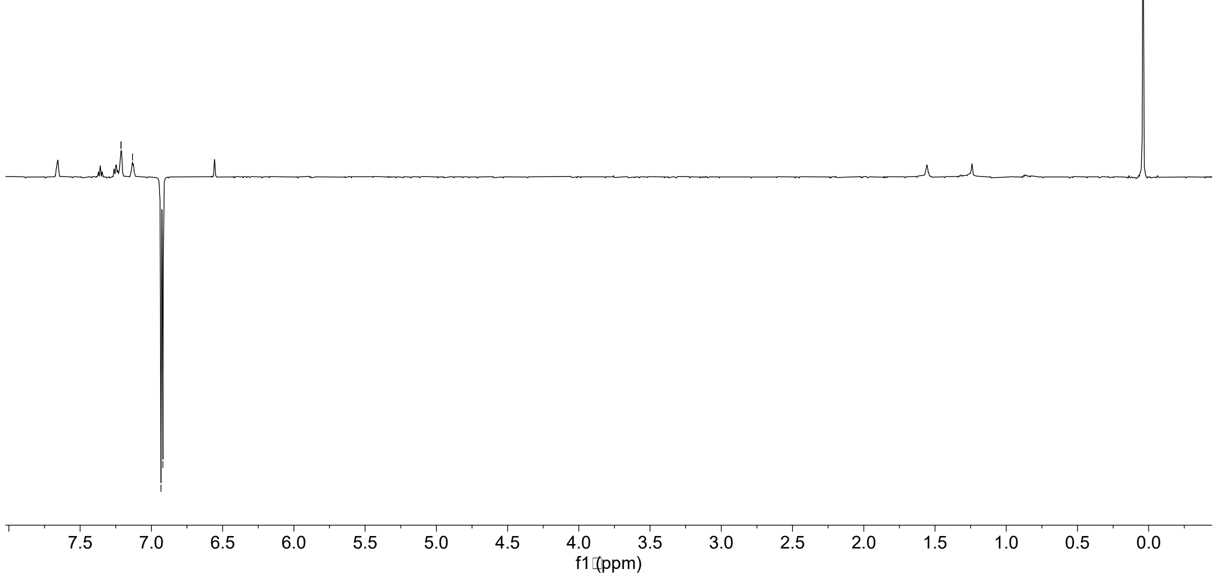

1-([1,1'-Biphenyl]-2-yl)-5-phenyl-1*H*-pyrazole (5h)

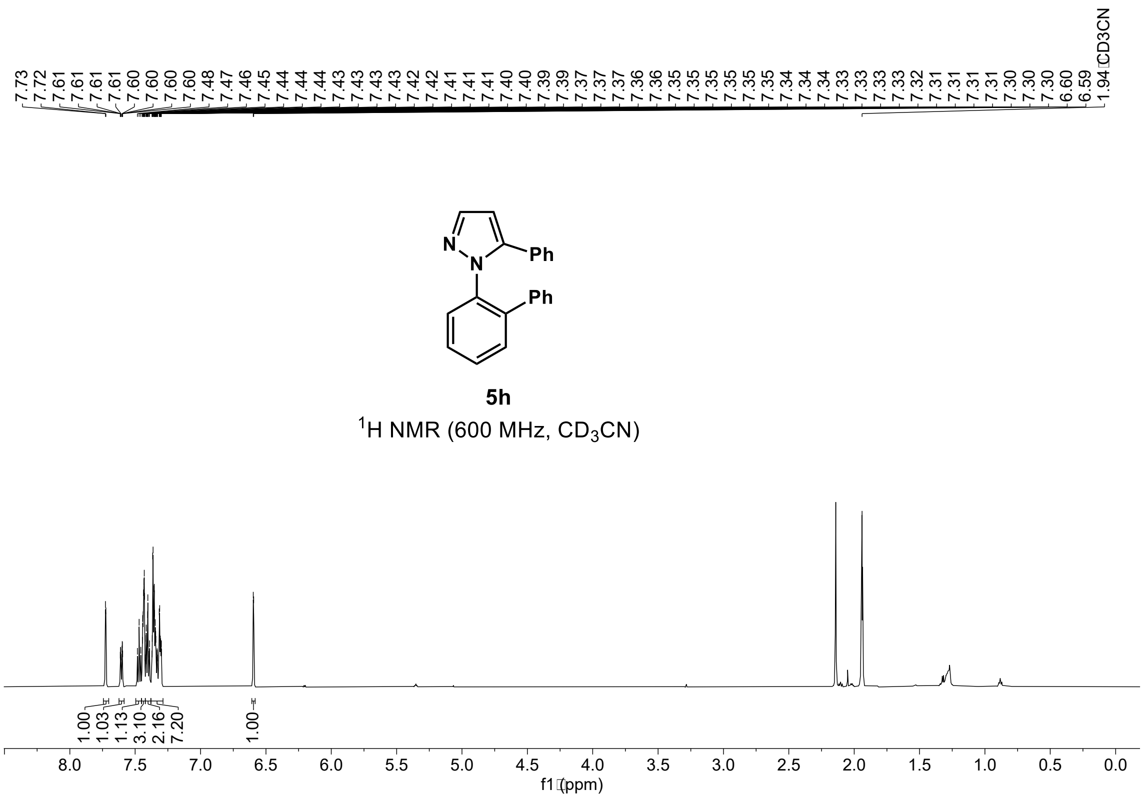

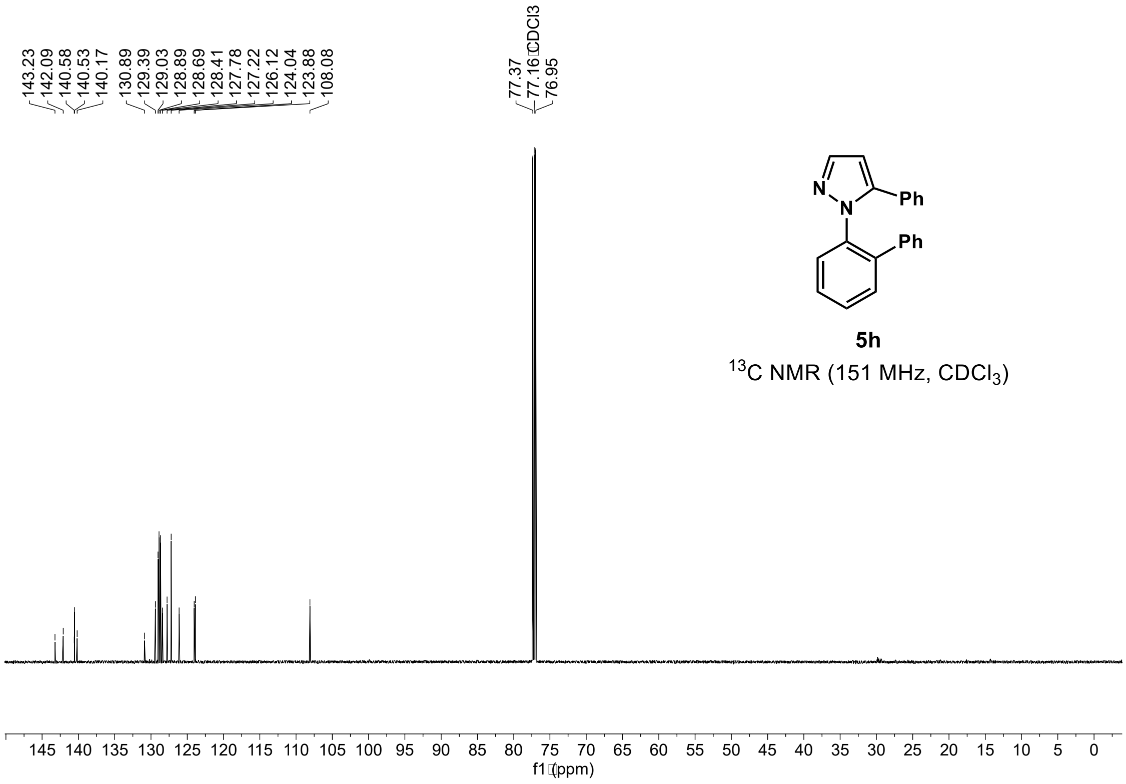

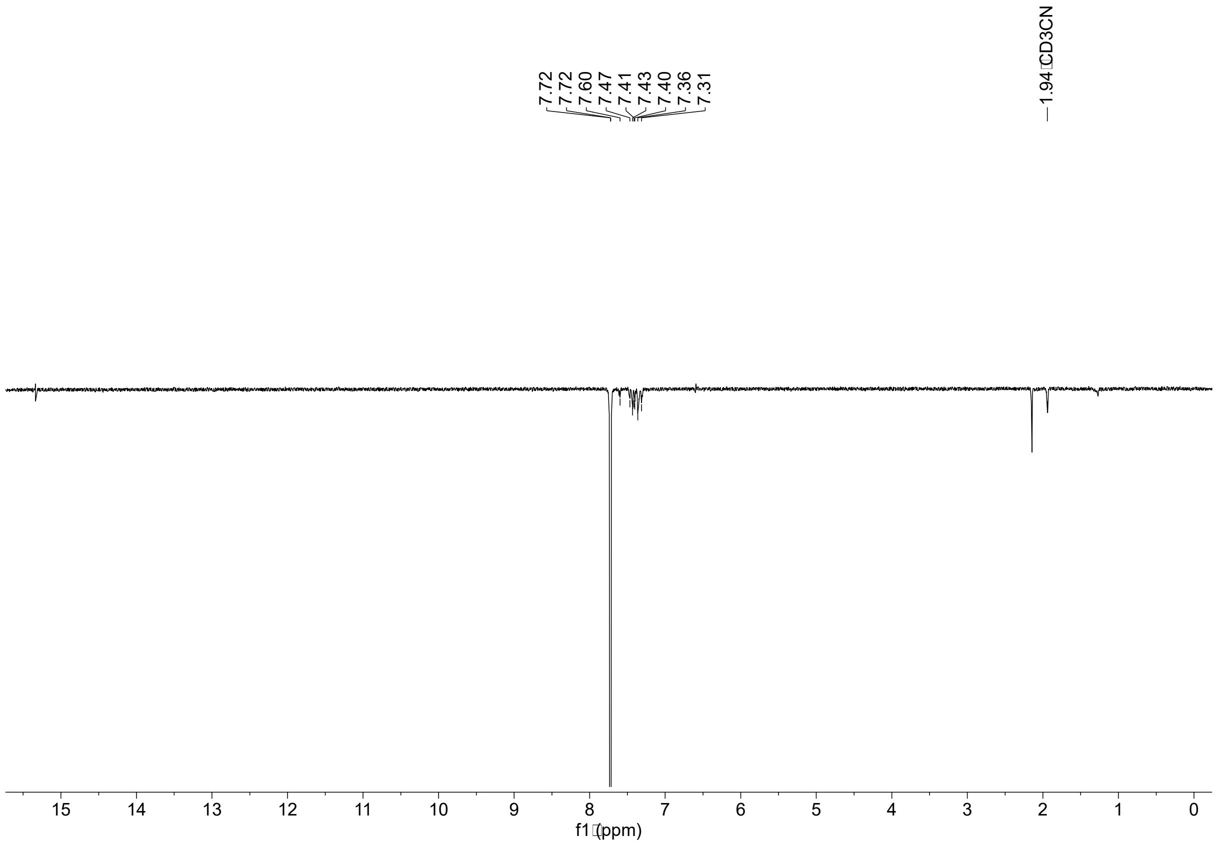

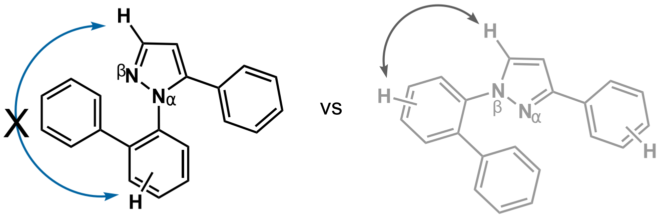

5h
NOE (600 MHz, CD_3CN)

1-(3-Nitrophenyl)-5-phenyl-1*H*-pyrazole (5i)

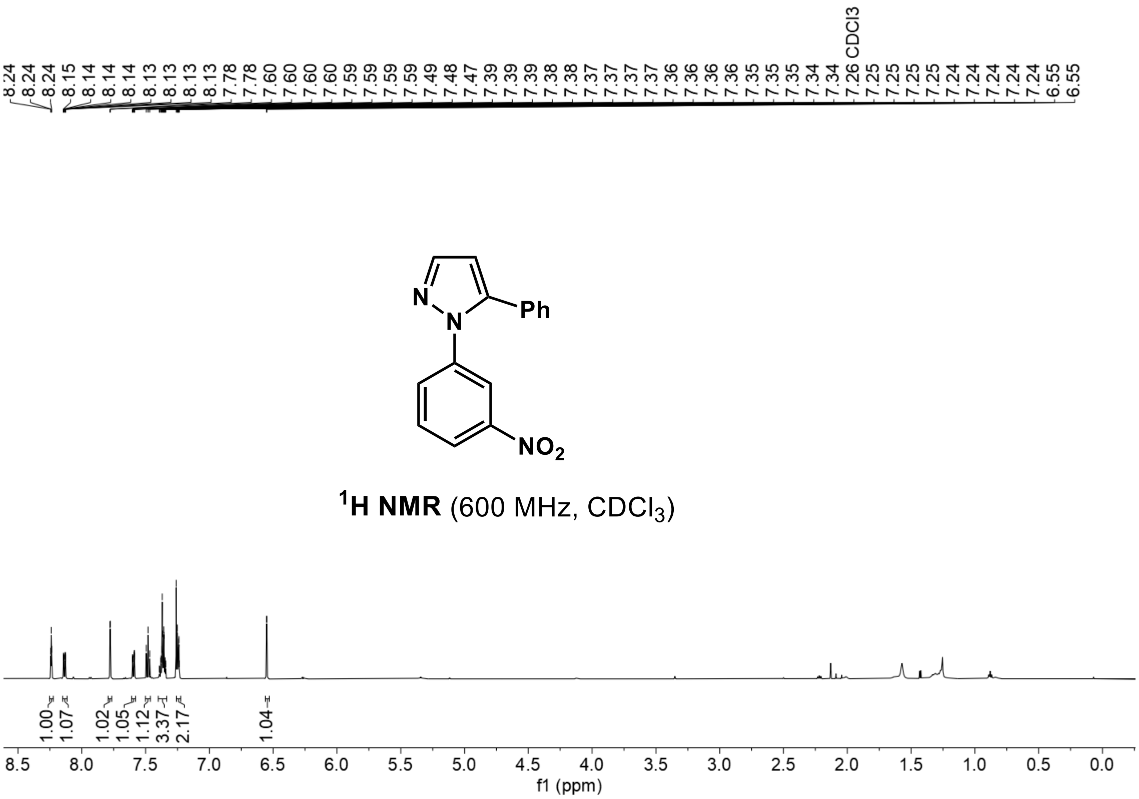

^{1}H NMR (600 MHz, $CDCl_3$)

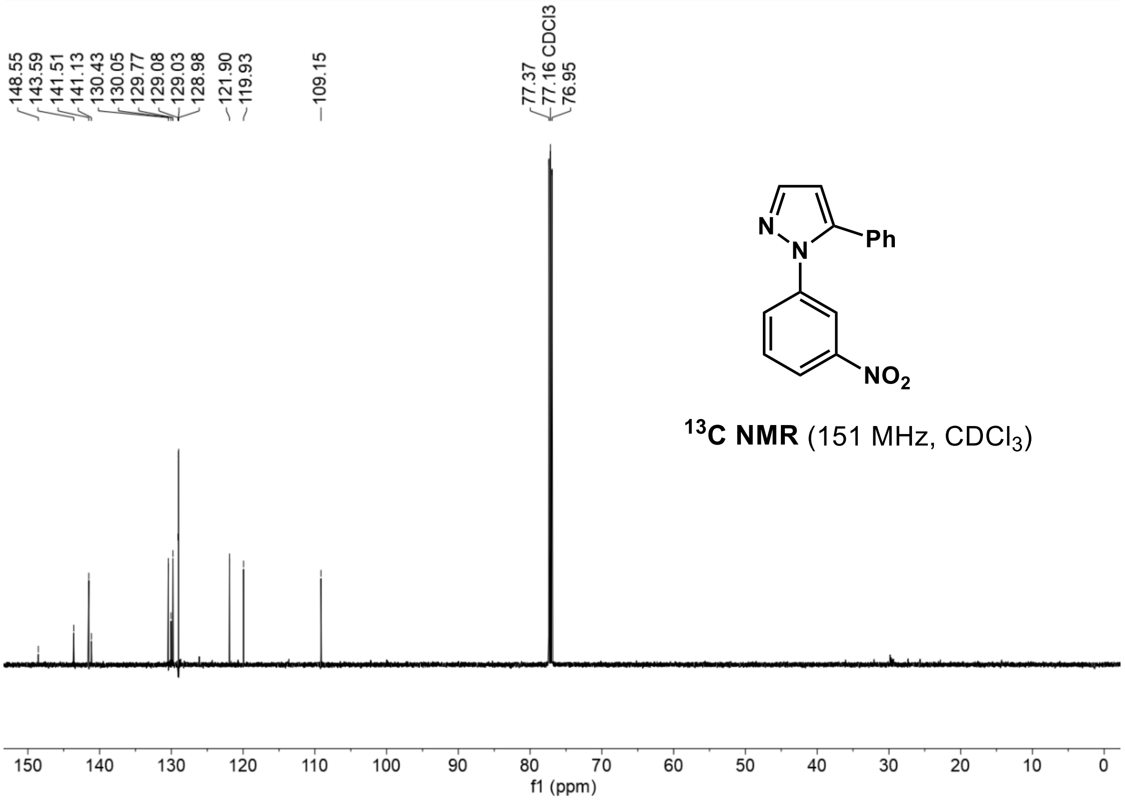

^{13}C NMR (151 MHz, $CDCl_3$)

1-(Naphthalen-1-yl)-5-phenyl-1*H*-pyrazole (5j')

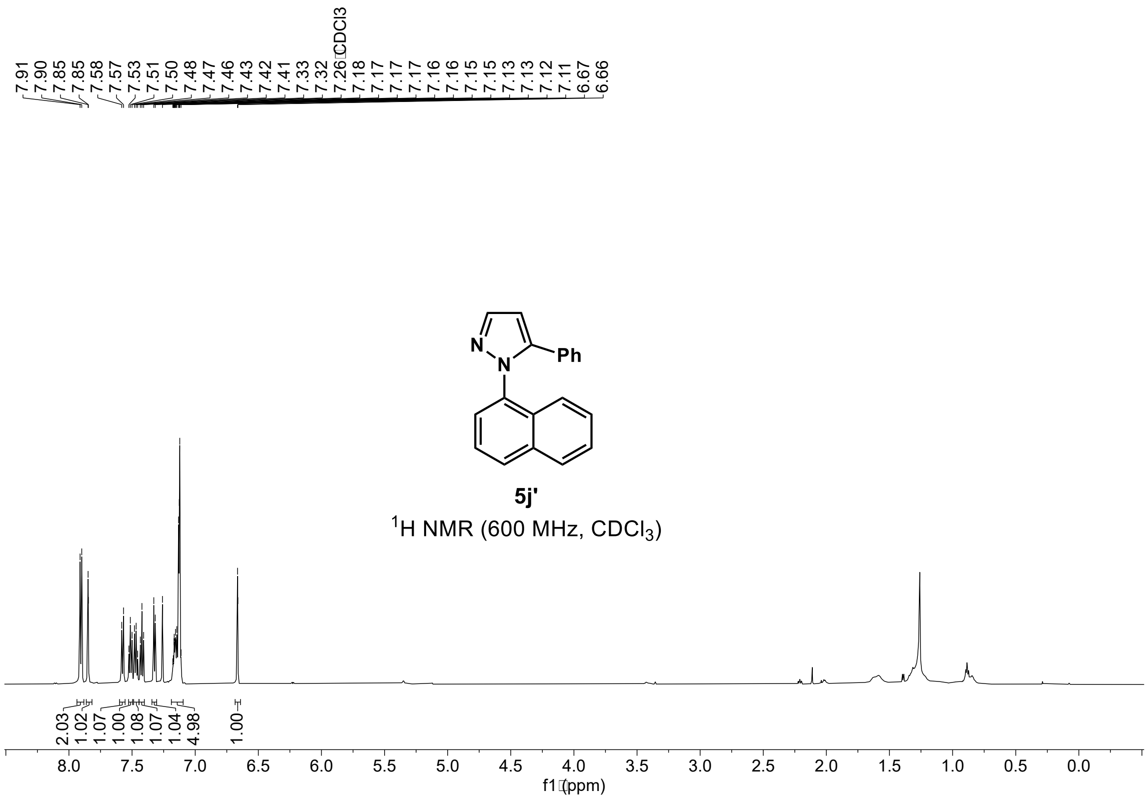

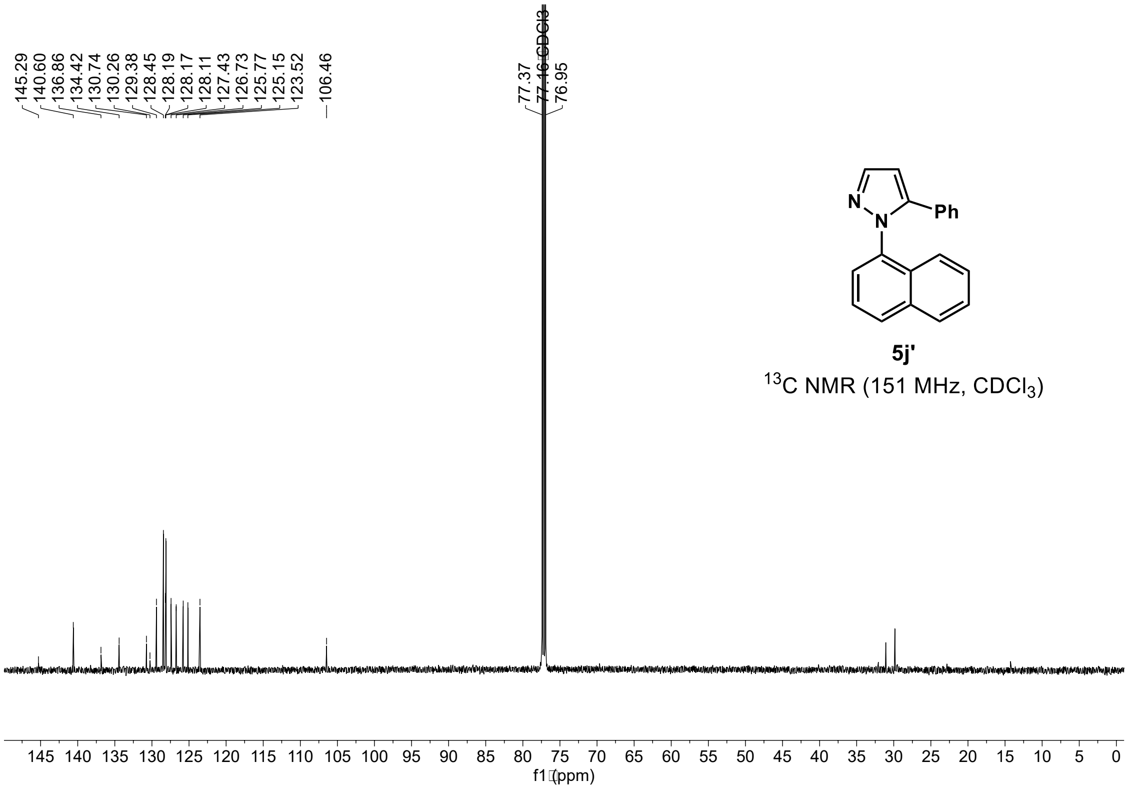

3-Cyano-*N*-(5-phenyl-1*H*-pyrazol-3-yl)benzamide (6)

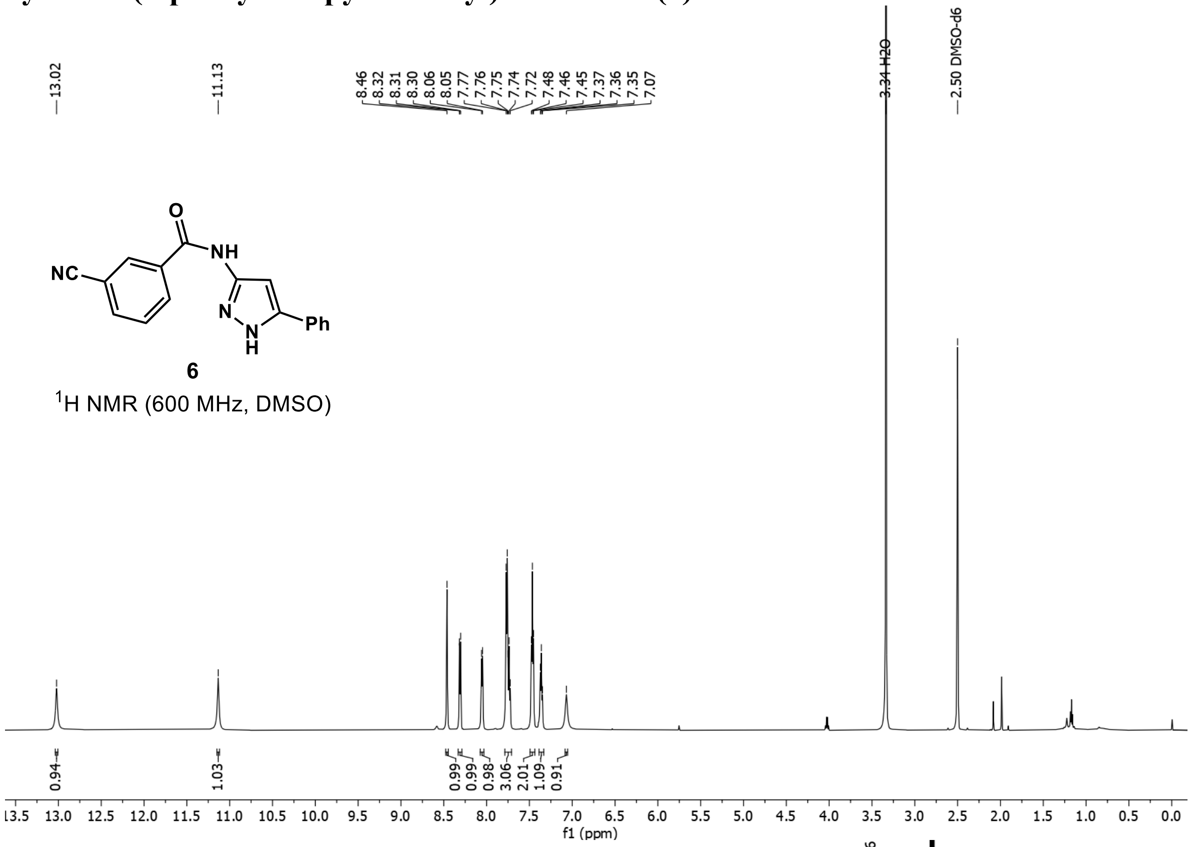

6
^{1}H NMR (600 MHz, DMSO)

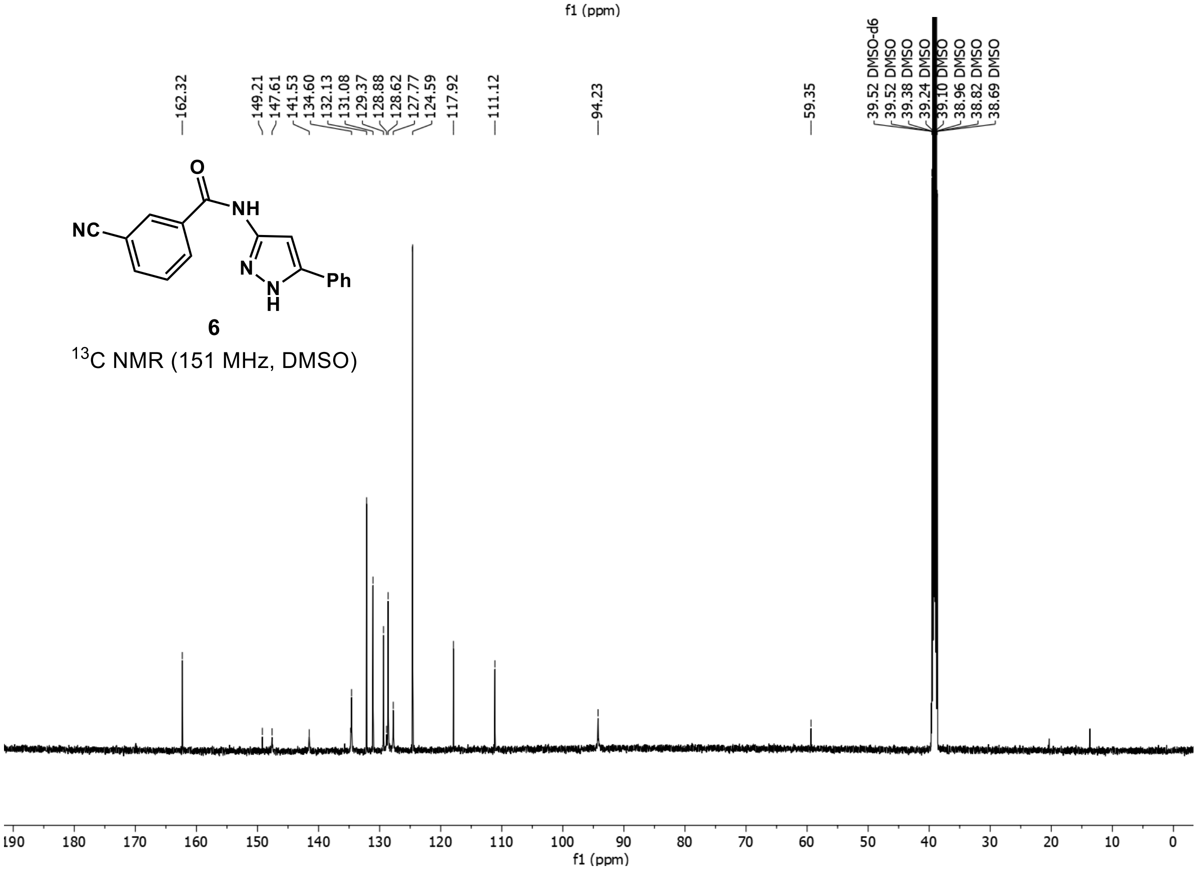

6
^{13}C NMR (151 MHz, DMSO)

3-Cyano-*N*-(1,3-diphenyl-1*H*-pyrazol-5-yl)benzamide (7, CDPPB)

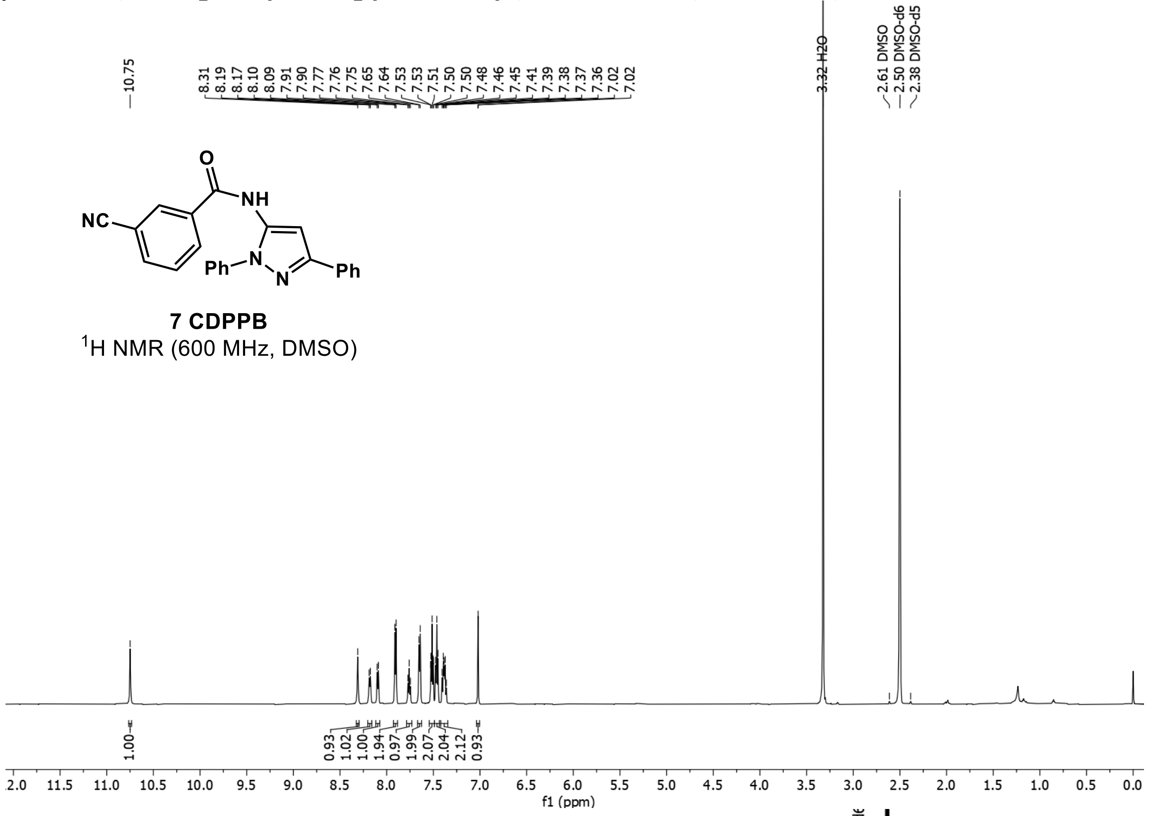

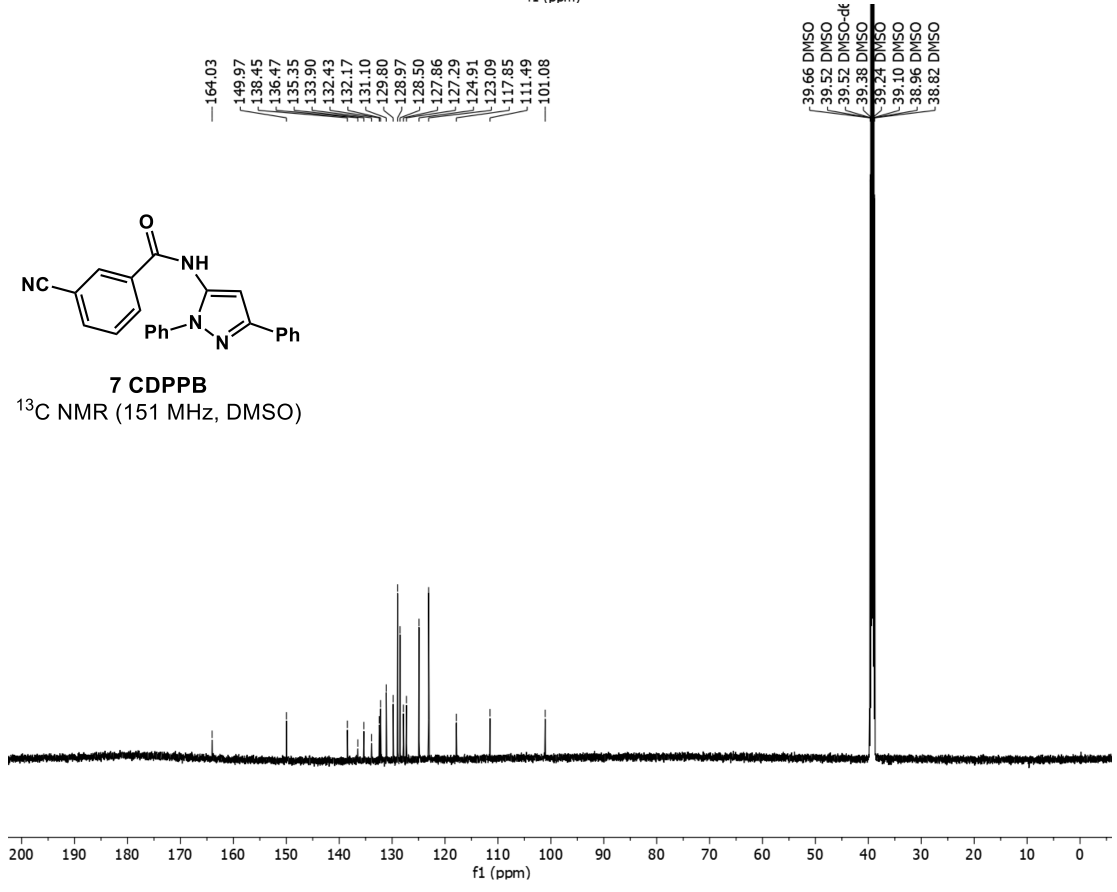

3-Cyano-*N*-(1,5-diphenyl-1*H*-pyrazol-3-yl)benzamide (8, CDPPB-*N*-Isomer)

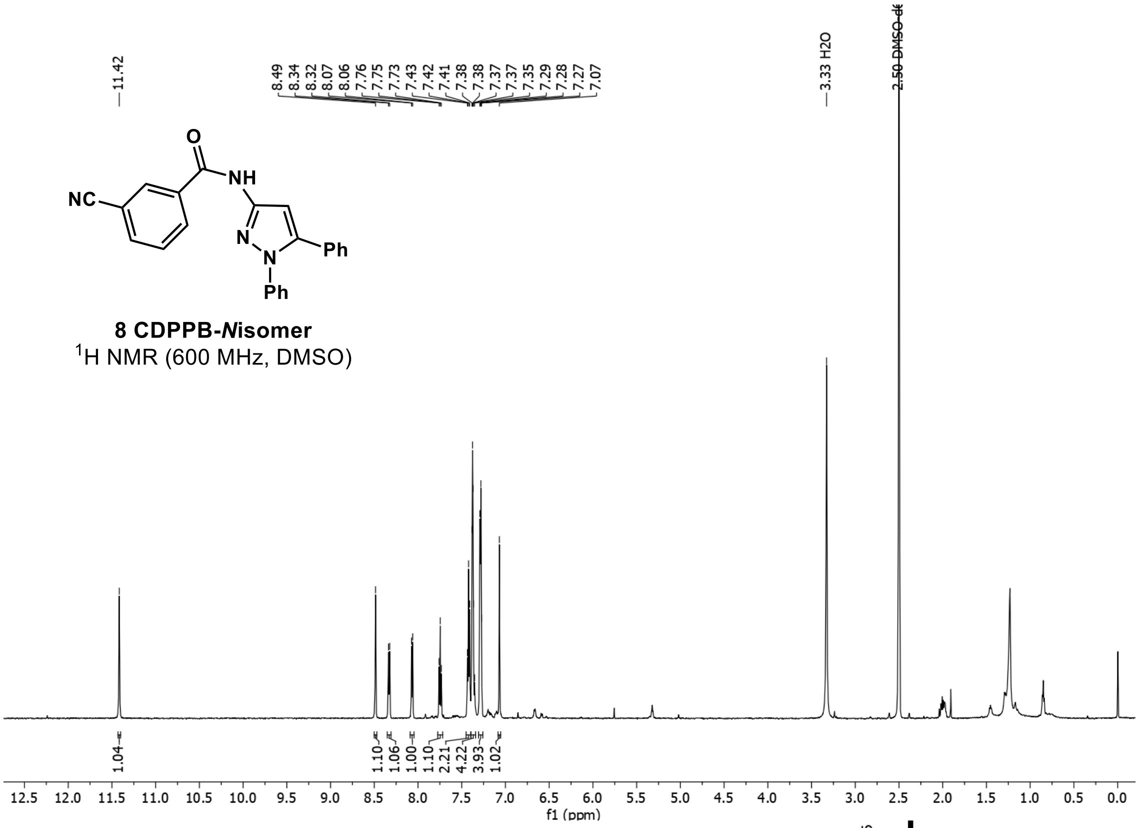

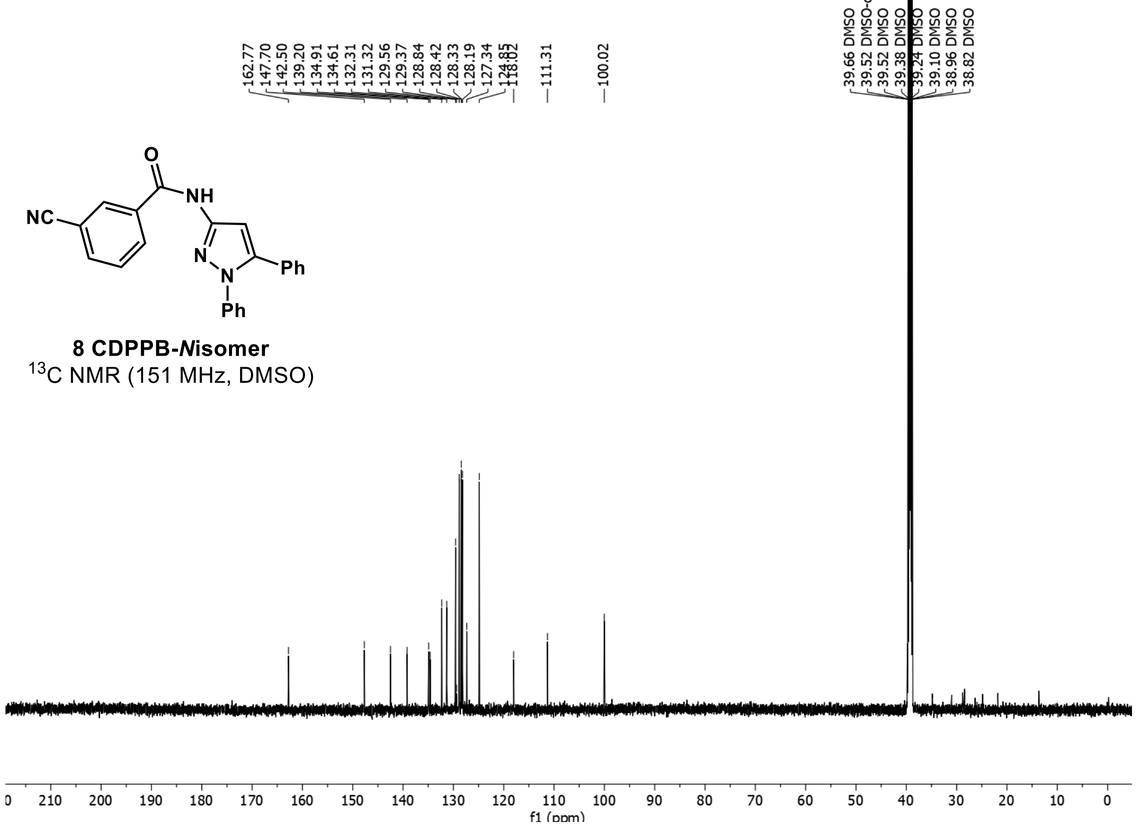

CyXant-Cu-Nα

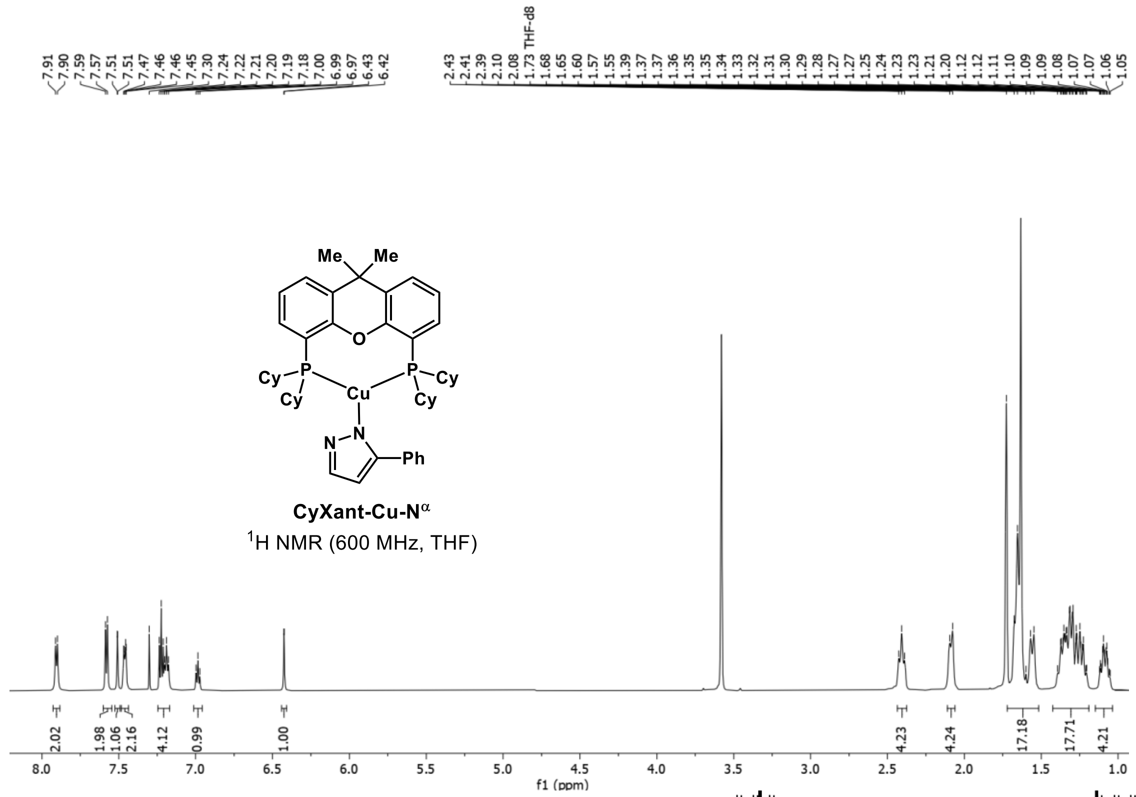
7.91
7.90
7.59
7.57
7.51
7.51
7.47
7.46
7.45
7.30
7.24
7.22
7.21
7.20
7.19
7.18
7.00
6.99
6.97
6.43
6.42
2.43
2.41
2.39
2.10
2.08
1.73 THF-d8
1.68
1.65
1.60
1.57
1.55
1.39
1.37
1.37
1.36
1.35
1.35
1.34
1.33
1.32
1.31
1.30
1.29
1.28
1.27
1.27
1.25
1.24
1.23
1.23
1.21
1.20
1.12
1.12
1.11
1.10
1.09
1.09
1.08
1.07
1.06
1.05
Me
Me
O
Cy
P
Cu
P
Cy
Cy
Cy
N
N
Ph
CyXant-Cu-Nα
1H NMR (600 MHz, THF)
2.02
1.98
1.06
2.16
4.12
0.99
1.00
4.23
4.24
17.18
17.71
4.21
8.0
7.5
7.0
6.5
6.0
5.5
5.0
4.5
4.0
3.5
3.0
2.5
2.0
1.5
1.0
f1 (ppm)

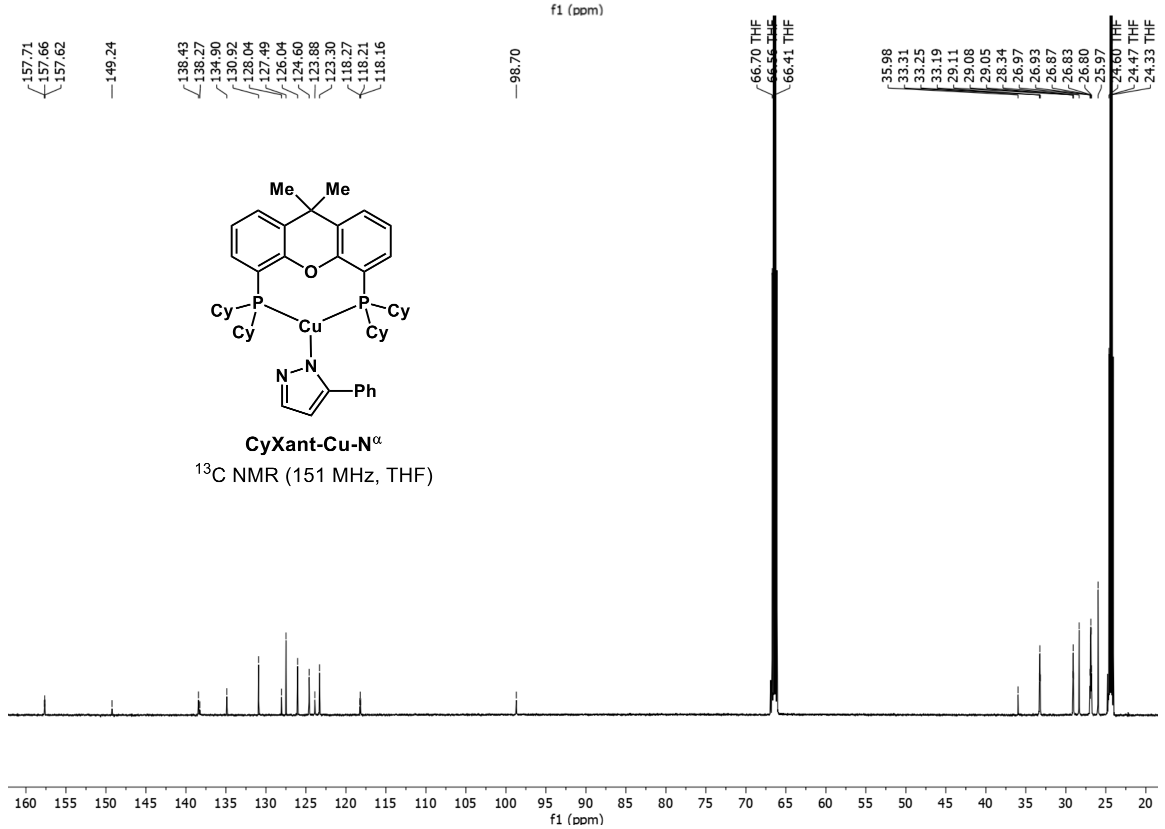
157.71
157.66
157.62
149.24
138.43
138.27
134.90
130.92
128.04
127.49
126.04
124.60
123.88
123.30
118.27
118.21
118.16
98.70
66.70 THF
66.56 THF
66.41 THF
35.98
33.31
33.25
33.19
29.11
29.08
29.05
28.34
26.97
26.93
26.87
26.83
26.80
25.97
24.60 THF
24.47 THF
24.33 THF
Me
Me
O
Cy
P
Cu
P
Cy
Cy
Cy
N
N
Ph
CyXant-Cu-Nα
13C NMR (151 MHz, THF)
160
155
150
145
140
135
130
125
120
115
110
105
100
95
90
85
80
75
70
65
60
55
50
45
40
35
30
25
20
f1 (ppm)

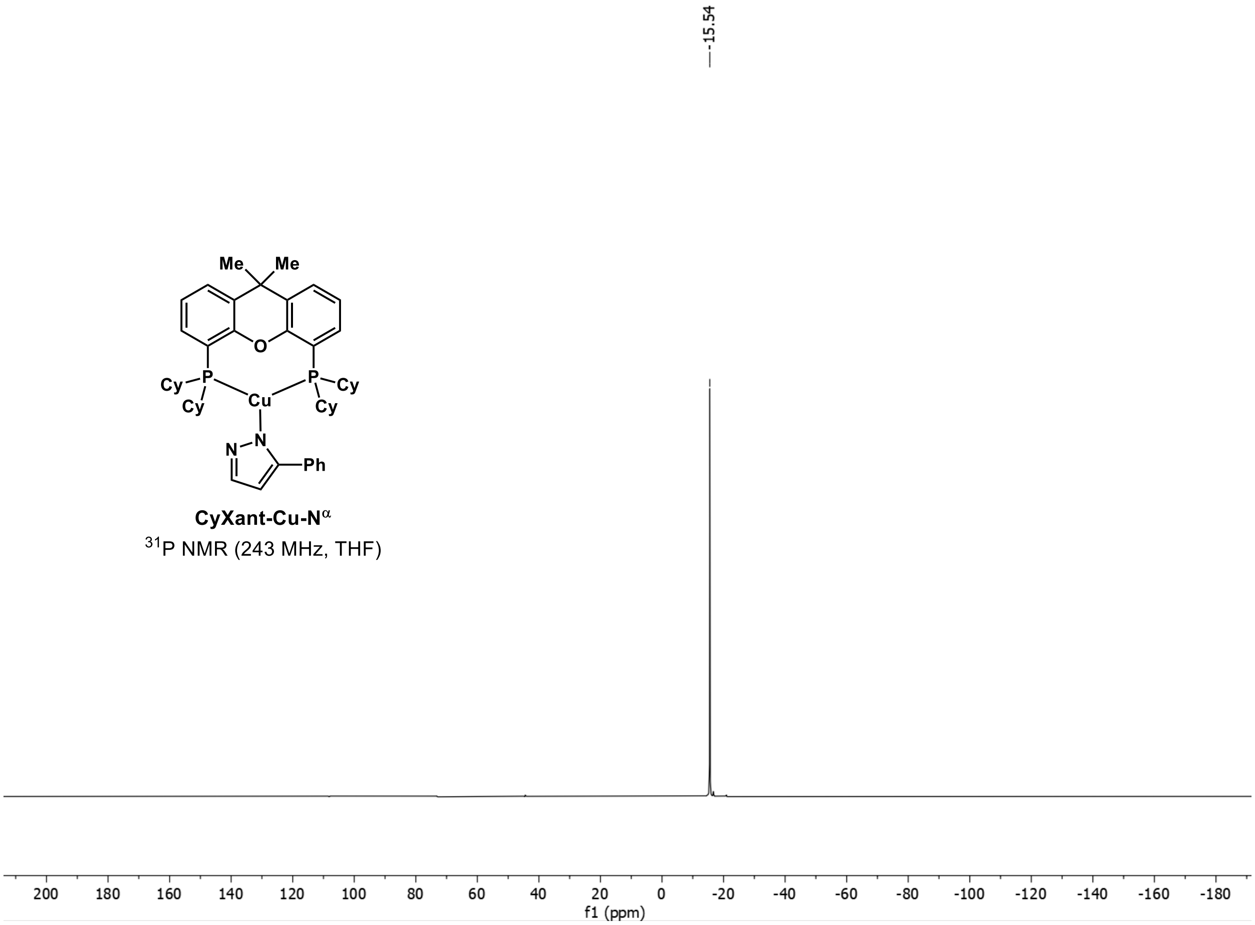

CyXant-Cu-N$^{\alpha}$

^{31}P NMR (243 MHz, THF)

13. References and Notes

63. K. Y. Lee, J. M. Kim, J. N. Kim, Regioselective synthesis of 1,3,4,5-tetrasubstituted pyrazoles from Baylis–Hillman adducts. *Tetrahedron Letters* **44**, 6737–6740 (2003).

64. G. Menozzi, L. Mosti, P. Schenone, Reaction of 2-dimethylaminomethylene-1,3-diones with dinucleophiles. VI. Synthesis of ethyl or methyl 1,5-disubstituted 1 *H* -pyrazole-4-carboxylates. *Journal of Heterocyclic Chem* **24**, 1669–1675 (1987).

65. A. Boelke, T. J. Kuczmera, E. Lork, B. J. Nachtsheim, N-Heterocyclic Iod(az)olium Salts – Potent Halogen-Bond Donors in Organocatalysis. *Chemistry A European J* **27**, 13128–13134 (2021).

66. S. Hernández, I. Moreno, R. SanMartin, G. Gómez, M. T. Herrero, E. Domínguez, Toward Safer Processes for C−C Biaryl Bond Construction: Catalytic Direct C−H Arylation and Tin-Free Radical Coupling in the Synthesis of Pyrazolophenanthridines. *J. Org. Chem.* **75**, 434–441 (2010).

67. M. Rey, S. Beaumont, Molybdenum-Mediated One-Pot Synthesis of Pyrazoles from Isoxazoles. *Synthesis* **51**, 3796–3804 (2019).

68. M. Kashiwa, Y. Kuwata, M. Sonoda, S. Tanimori, Oxone-mediated facile access to substituted pyrazoles. *Tetrahedron* **72**, 304–311 (2016).

69. R. S. Foster, H. Jakobi, J. P. A. Harrity, A General and Regioselective Synthesis of 5-Trifluoromethyl-pyrazoles. *Org. Lett.* **14**, 4858–4861 (2012).

70. G. Pai, A. P. Chattopadhyay, N-Arylation of nitrogen containing heterocycles with aryl halides using copper nanoparticle catalytic system. *Tetrahedron Letters* **57**, 3140–3145 (2016).

71. N. Yang, G. Yuan, A Multicomponent Electrosynthesis of 1,5-Disubstituted and 1-Aryl 1,2,4-Triazoles. *J. Org. Chem.* **83**, 11963–11969 (2018).

72. X. Tang, L. Huang, J. Yang, Y. Xu, W. Wu, H. Jiang, Practical synthesis of pyrazoles via a copper-catalyzed relay oxidation strategy. *Chem. Commun.* **50**, 14793–14796 (2014).

73. G. Shan, P. Liu, Y. Rao, A New Synthesis of Pyrazoles through a Lewis Acid Catalyzed Union of 3-Ethoxycyclobutanones with Monosubstituted Hydrazines. *Org. Lett.* **13**, 1746–1749 (2011).

74. F. Pünner, Y. Sohtome, M. Sodeoka, Solvent-dependent copper-catalyzed synthesis of pyrazoles under aerobic conditions. *Chem. Commun.* **52**, 14093–14096 (2016).

75. V. V. Voronin, M. S. Ledovskaya, E. G. Gordeev, K. S. Rodygin, V. P. Ananikov, [3 + 2]-Cycloaddition of *in Situ* Generated Nitrile Imines and Acetylene for Assembling of 1,3-Disubstituted Pyrazoles with Quantitative Deuterium Labeling. *J. Org. Chem.* **83**, 3819–3828 (2018).

76. G.-Q. Jin, W.-X. Gao, Y.-B. Zhou, M.-C. Liu, H.-Y. Wu, Efficient synthesis of 2-aryl-2 *H* -indazoles by base-catalyzed benzyl C–H deprotonation and cyclization. *Chem. Commun.* **56**, 14617–14620 (2020).

77. A. C. Spivey, C. M. Diaper, H. Adams, A. J. Rudge, A New Germanium-Based Linker for Solid Phase Synthesis of Aromatics: Synthesis of a Pyrazole Library. *J. Org. Chem.* **65**, 5253–5263 (2000).

78. Liu, J. *et al.* Copper-mediated tandem ring-opening/cyclization reactions of cyclopropanols with aryldiazonium salts: synthesis of *N* -arylpyrazoles. *Chem. Commun.* **56**, 2202–2205 (2020).

79. C. Dunker, L. Imberg, A. I. Siutkina, C. Erbacher, C. G. Daniliuc, U. Karst, D. V. Kalinin, Pyrazole-Based Thrombin Inhibitors with a Serine-Trapping Mechanism of Action: Synthesis and Biological Activity. *Pharmaceuticals* **15**, 1340 (2022).

80. S. Grimme. Supramolecular binding thermodynamics by dispersion-corrected density functional theory. *Chem. Eur. J.* **18**, 9955-9964 (2012).

81. C. Y. Legault. CYLview, 1.0b; Université de Sherbrooke, **2009** (http://www.cylview.org).

82. T. Lu, F. Chen. Multiwfn: A multifunctional wavefunction analyzer. *J. Comput. Chem.* **33**, 580-592 (2012).

83. W. Humphrey, A. Dalke, K. Schulten. "VMD - Visual Molecular Dynamics" *J. Molec. Graphics*, **14**, 33-38 (1996).

84. E. F. Peterson, T. D. Goddard, C. C. Huang, E. C. Meng, G. S. Couch, T. I. Croll, J. H. Morris, T. E. Ferrin. "UCSF ChemieraX: Structure visualization for researchers, educators, and developers." *Protein Sci*, **30**, 70-82 (2021).

85. A. D. Becke. Density-functional thermochemistry. III. The role of exact exchange. *J. Chem. Phys.* **98**, 5648-5652 (1993).

86. S. Grimme, J. Antony, S. Ehrlich, H. A. Krieg. Consistent and accurate ab initio parametrization of density functional dispersion correction (DFT-D) for the 94 elements H-Pu. *J. Chem. Phys.* **132**, 154104 (2010).

87. S. Grimme. Semiempirical GGA-type density functional constructed with a long-range dispersion correction. *J. Comput. Chem.* **27**, 1787-1799 (2006).

88. A. Becke. Density-Functional Thermochemistry. V. Systematic Optimization of Exchange-Correlation Functionals. *J. Chem. Phys.* **107**, 8554-8560 (1997).

89. J. D. Chai, M. Head-Gordon. Long-range corrected hybrid density functionals with damped atom-atom dispersion corrections. *Phys. Chem. Chem. Phys.* **10**, 6615-6620 (2008).

90. F. Weigend, R. Ahlrichs. Balanced basis sets of split valence, triple zeta valence and quadruple zeta valence quality for H to Rn: Design and assessment of accuracy. *Phys. Chem. Chem. Phys.* **7**, 3297-3305 (2005).

91. A. V. Marenich, C. J. Cramer, D. G. Truhlar. Universal solvation model based on solute electron density and on a continuum model of the solvent defined by the bulk dielectric constant and atomic surface tensions. *J. Phys. Chem. B* **113**, 6378-6396 (2009).

92. A. J. Schaefer, V. M. Ingman, and S. E. Wheeler, "SEQCROW: A ChimeraX Bundle to Facilitate Quantum Chemical Applications to Complex Molecular Systems" *J. Comp. Chem.* **42**, 1750 (2021).

93. V. M. Ingman, A. J. Schaefer, L. R. Andreola, and S. E. Wheeler, "QChASM: Quantum Chemistry Automation and Structure Manipulation" *WIREs Comp. Mol. Sci*. **11**, e1510 (2021).

94. S. Parsons, H. D. Flack, T. Wagner, Use of intensity quotients and differences in absolute structure refinement. *Acta Crystallogr B Struct Sci Cryst Eng Mater* **69**, 249–259 (2013).

95. B. Lakshmi, U. Wefelscheid, U. Kazmaier, Synthesis of Aryl Stannanes from Silyl Triflates via Aryne Intermediates. *Synlett* **2011**, 345–348 (2011).

96. Bickelhaupt, F. M.; Houk, K. N. Analyzing Reaction Rates with the Distortion/Interaction-Activation Strain Model. *Angew. Chem. Int. Ed.* **56**, 10070 (2017).

97. Ess, D. H.; Houk, K. N. Distortion/Interaction Energy Control of 1,3-Dipolar Cycloaddition Reactivity. *J. Am. Chem. Soc.*, **129**, 10646-10647 (2007).

98. Maji, R.; Mallojjala, S. C.; Wheeler, S. E. Chiral Phosphoric Acid Catalysis: From Numbers to Insights. *Chem. Soc. Rev.* **47**, 1142-1158 (2018).

99. Lee, K. Y., Kim, J. M. & Kim, J. N. Regioselective synthesis of 1,3,4,5-tetrasubstituted pyrazoles from Baylis–Hillman adducts. *Tetrahedron Letters* **44**, 6737–6740 (2003).

100. Bickelhaupt, F. M.; Houk, K. N. Analyzing Reactino Rates with the Distortion/Interaction Activation Strain Model. *Angew. Chem. Int. Ed. Engl.*, **56**, 10070 (2017).

101. Ess, D. H.; Houk, K. N. Distortion/Interaction Energy control of 1,3-Dipolar Cycloaddition Reactivity. *J. Am. Chem. Soc.* **129**, 10646-10647 (2007).

102. Maji, R.; Mallojjala, S. C.; Wheeler, S. E. Chiral phosphoric acid catalysis: from numbers to insights. *Chem. Soc. Rev.* **47**, 1142 (2018).
